# Supplementary material for: Strength dependency of frequency–magnitude distribution in earthquakes and implications for stress state criticality
Source: Nat Commun. 2024 Jun 11;15:4957. doi: 10.1038/s41467-024-49422-7 (PMC11166660; doi:10.1038/s41467-024-49422-7)
Supplement: Supplementary file 1 — Supplementary Information [file 41467_2024_49422_MOESM1_ESM.pdf]

Supplementary Information

Examining the strength dependency of frequency–magnitude distribution in small earthquakes: Implications for stress state criticality

Satoshi Matsumoto, Yoshihisa Iio, Shinichi Sakai, and Aitaro Kato

Correspondence: Satoshi Matsumoto; matumoto@sevo.kyushu-u.ac.jp

This supplementary information includes:

Supplementary Figures 1 to 10

Supplementary Table 1 and 2

The data for ploting Supplementary Figure 1 is available from site of Japan Meteological Agancy (https://www.data.jma.go.jp/svd/eqev/data/bulletin/hypo_e.html)

Supplementary Figures

a

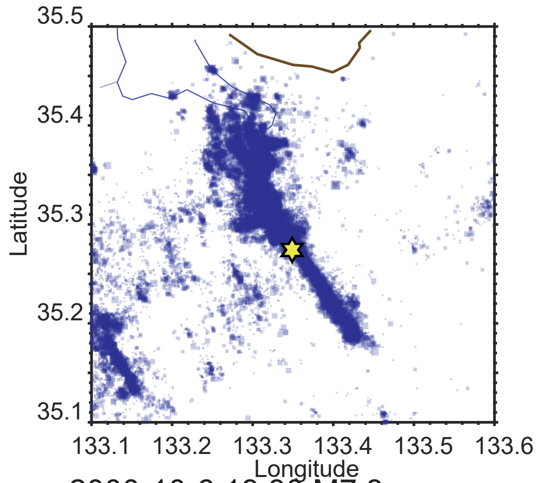
35.5
35.4
35.3
35.2
35.1
Latitude
133.1
133.2
133.3
133.4
133.5
133.6
Longitude

b

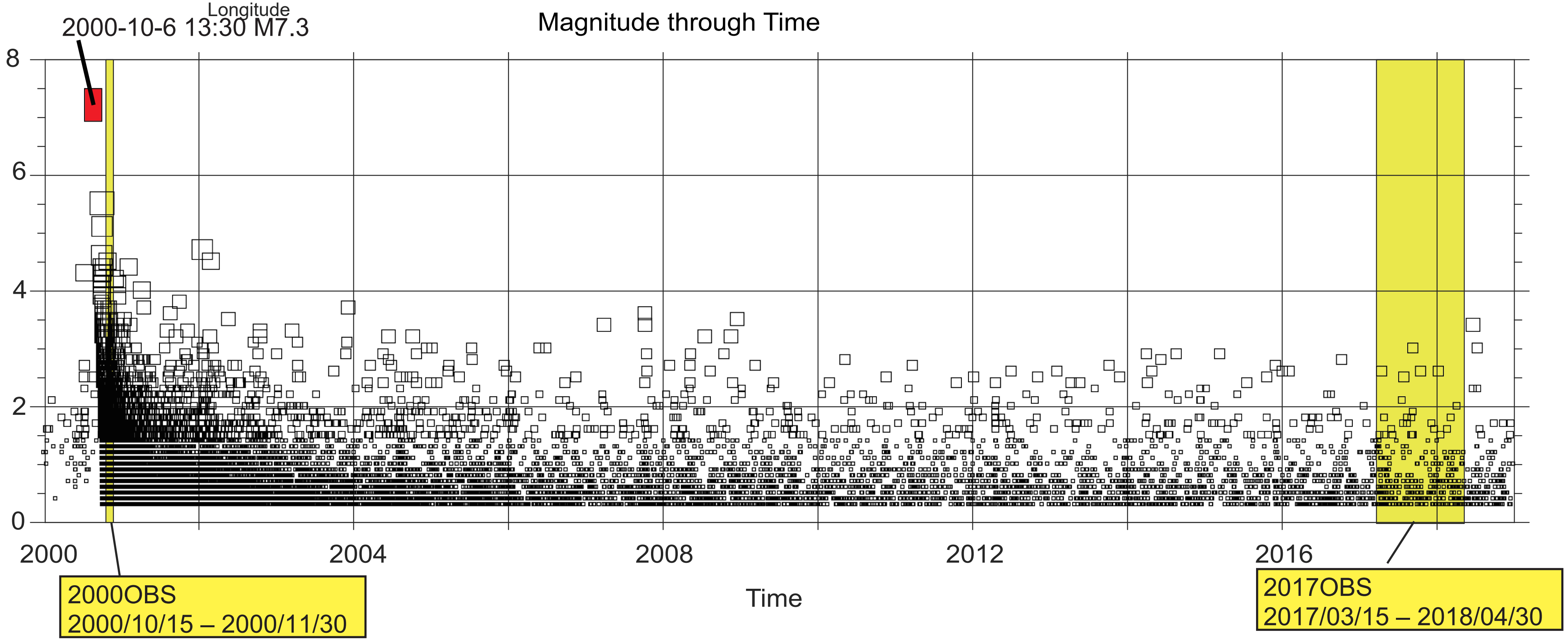
2000-10-6 13:30 M7.3
Magnitude through Time
8
6
4
2
0
2000
2004
2008
2012
2016
Time
2000OBS
2000/10/15 – 2000/11/30
2017OBS
2017/03/15 – 2018/04/30

Supplementary Fig. 1. Time sequency of the main shock (M7.3) and 2000, 2017 OBS period.

a Map showing hypocentral distribution during 2000 to 2018 Jun from Main shock from catalog by Japan Meteorological Agancy (JMA). Star indicates epicentre of the M7.3 mainshock. **b** the mainshock (red square) and observation period for 2000 and 2017 observations (yellow shade) are indicated with Magnitude – Time distribution plot from 2000 until 2018 June. The plot is as reference to demonstrate seismicity change in the target area although detectability is different from both 2000 and 2017 OBS.

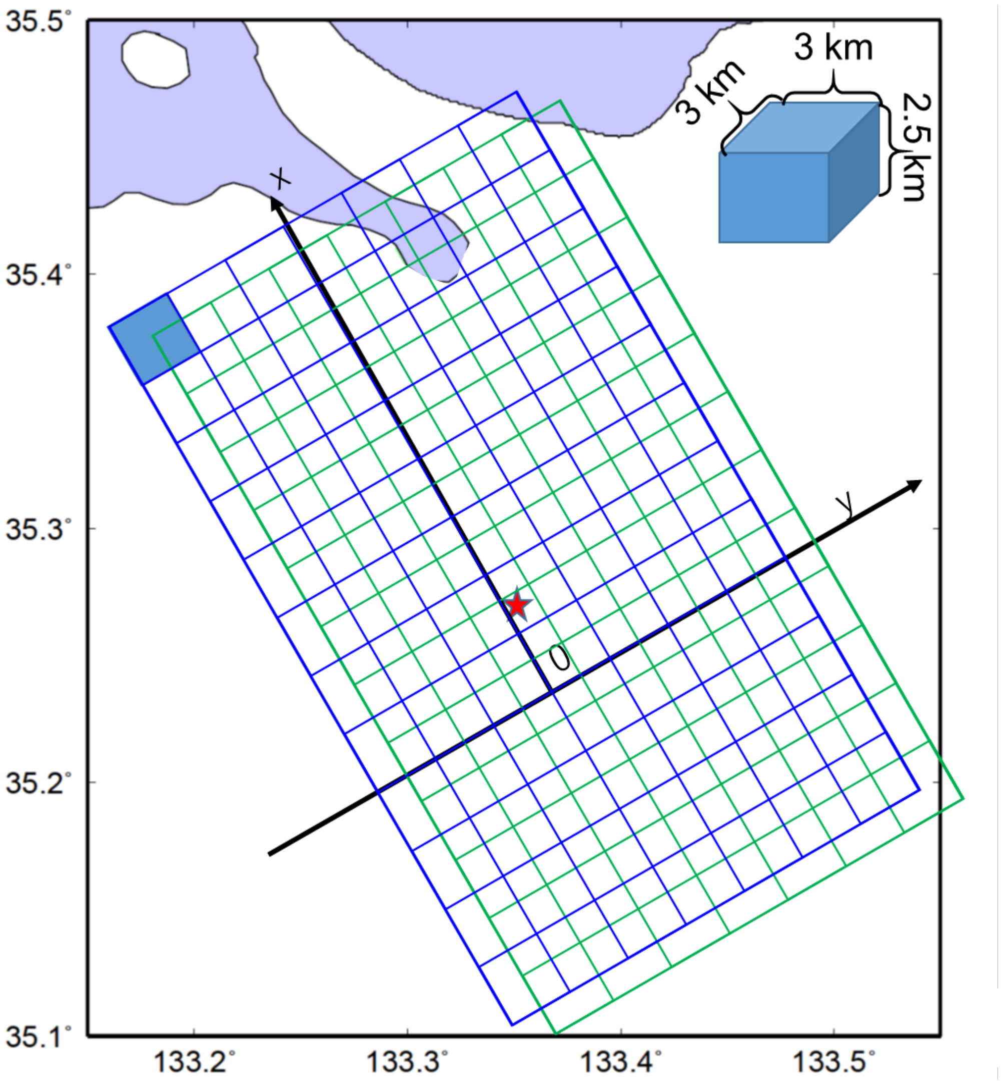

Supplementary Fig. 2. Spatial bin distribution (blue).

The size of each bin is 3 × 3 × 2.5 km. Grid distribution is shifted southwest by half a bin size (green) for smoothing Δp distribution. Δp is estimated for several events equal to or greater than 20 for each spatial bin. The red star indicates the epicentre of the 2000 M7.3 Western Tottori earthquake.

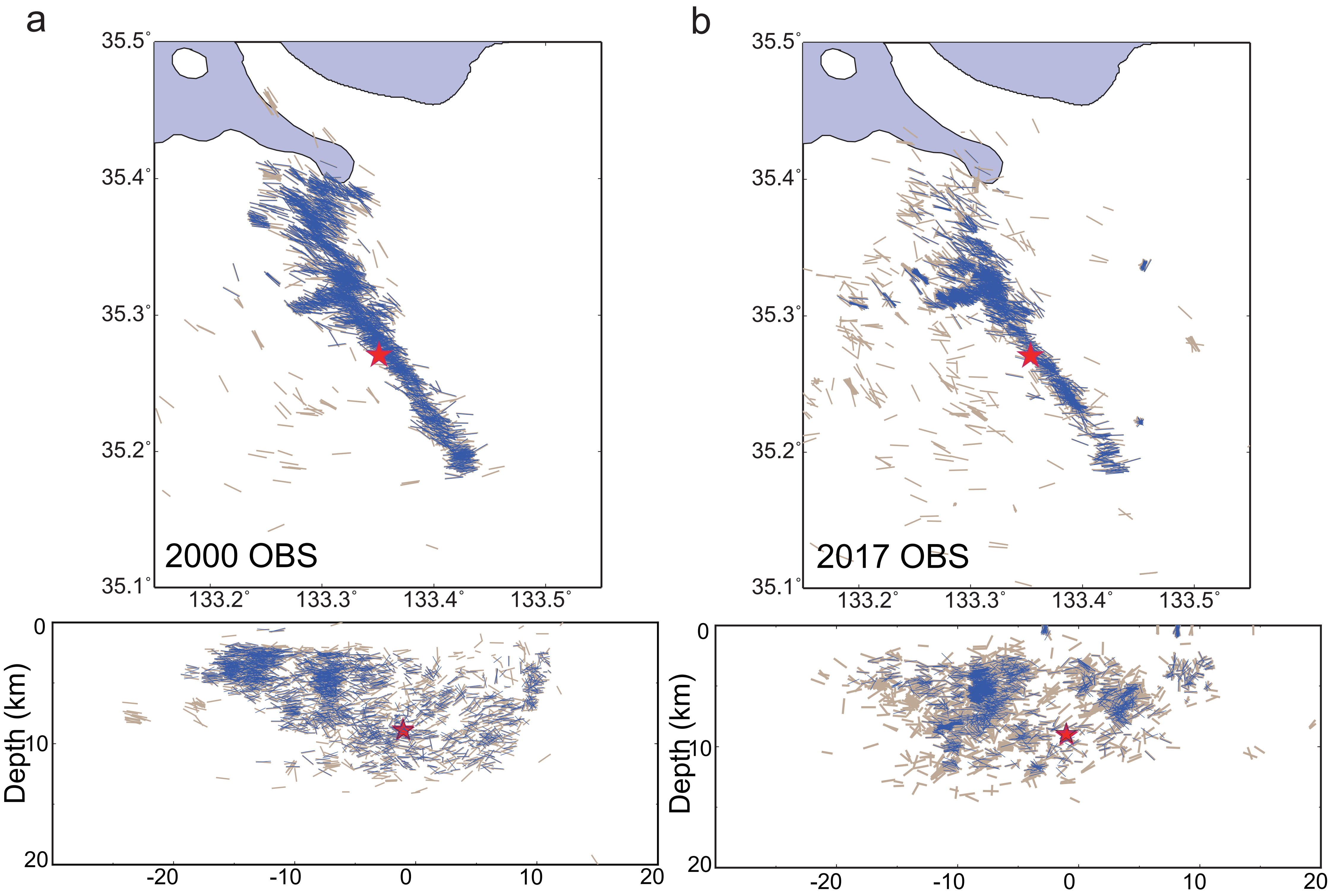

a
b
35.5°
35.4°
35.3°
35.2°
35.1°
133.2°
133.3°
133.4°
133.5°
2000 OBS
2017 OBS
Depth (km)
0
10
20
-20
-10
0
10
20

Supplementary Fig. 3. Vertical cross-section of P axis distribution of a 2000 OBS and b) 2017 OBS.

Segments are P axes in **a** 2000 OBS and **b** 2017 OBS. Map and vertical cross section in N30°W are shown in upper and lower plate, respectively. Blue segments show the P axes of focal mechanisms used in the stress estimation (see main text). The star indicates the hypocentre of the mainshock, determined by the Japan Meteorological Agency. Segments are projected on horizontal and vertical plane in N30°W.

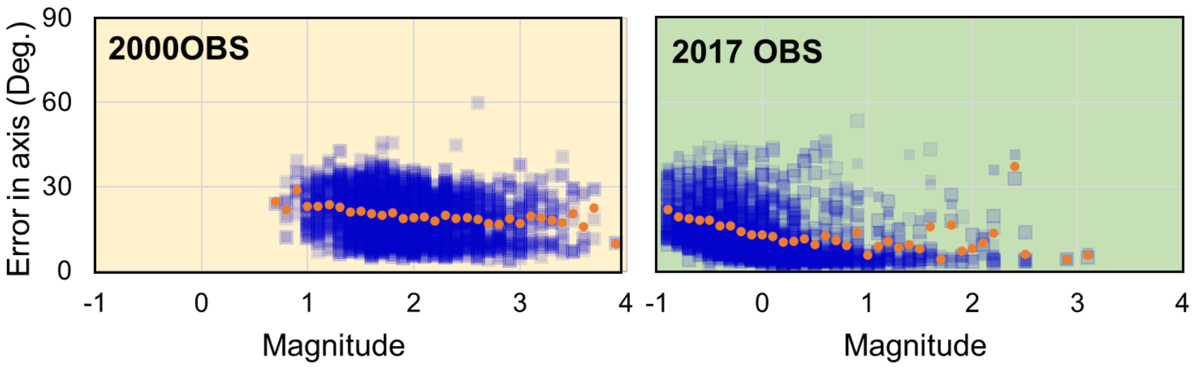

Supplementary Fig. 4. Standard errors of normal vectors of focal mechanism.

Left and right panels show values for 2000 and 2017 OBS, respectively. The blue symbol indicates the error for each normal vector. The orange symbol shows average values in the magnitude range with an interval of 0.1.

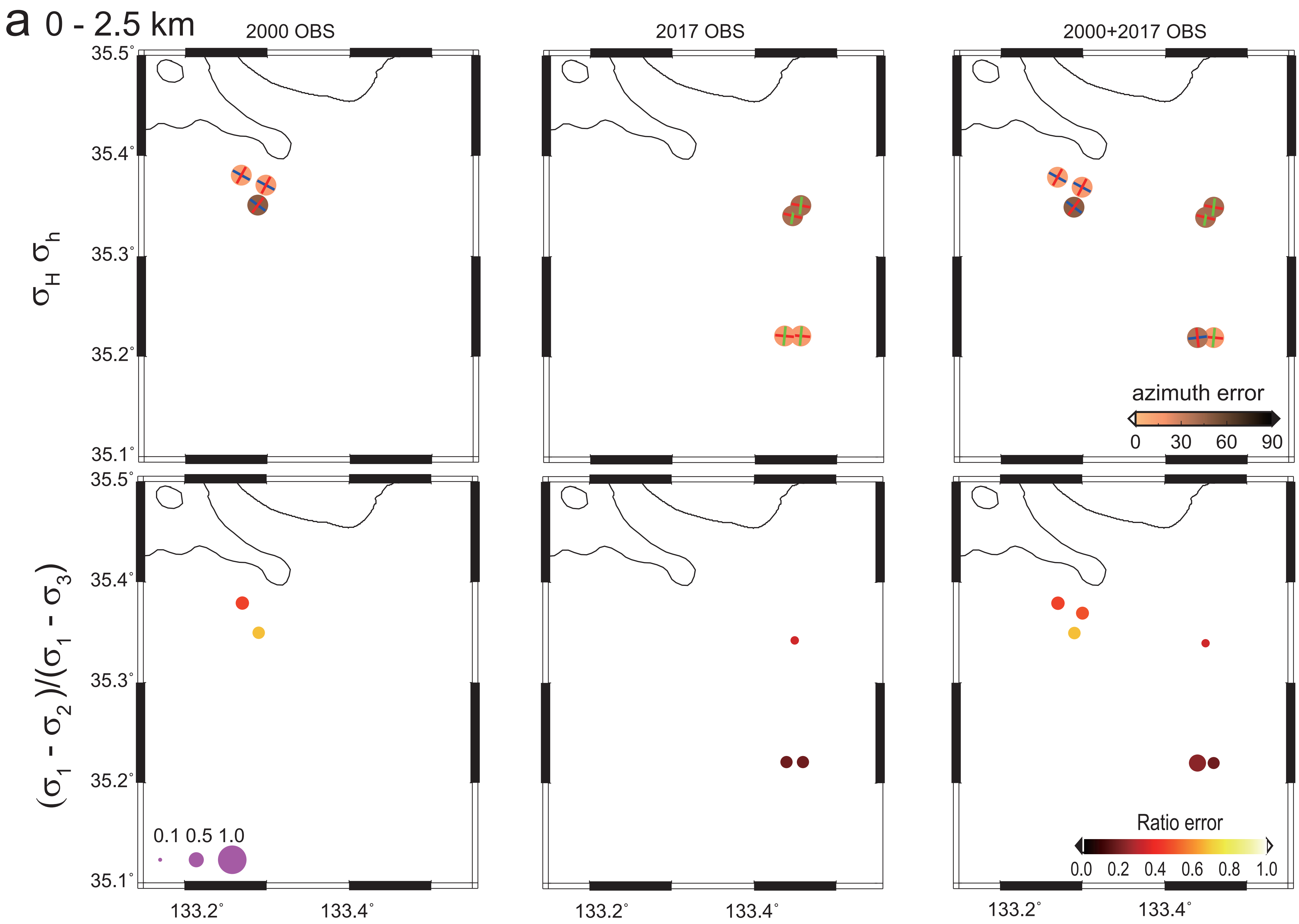

a 0 - 2.5 km
2000 OBS
2017 OBS
2000+2017 OBS
σH σh
(σ1 - σ2)/(σ1 - σ3)
35.5°
35.4°
35.3°
35.2°
35.1°
133.2°
133.4°
azimuth error
0
30
60
90
0.1 0.5 1.0
Ratio error
0.0 0.2 0.4 0.6 0.8 1.0

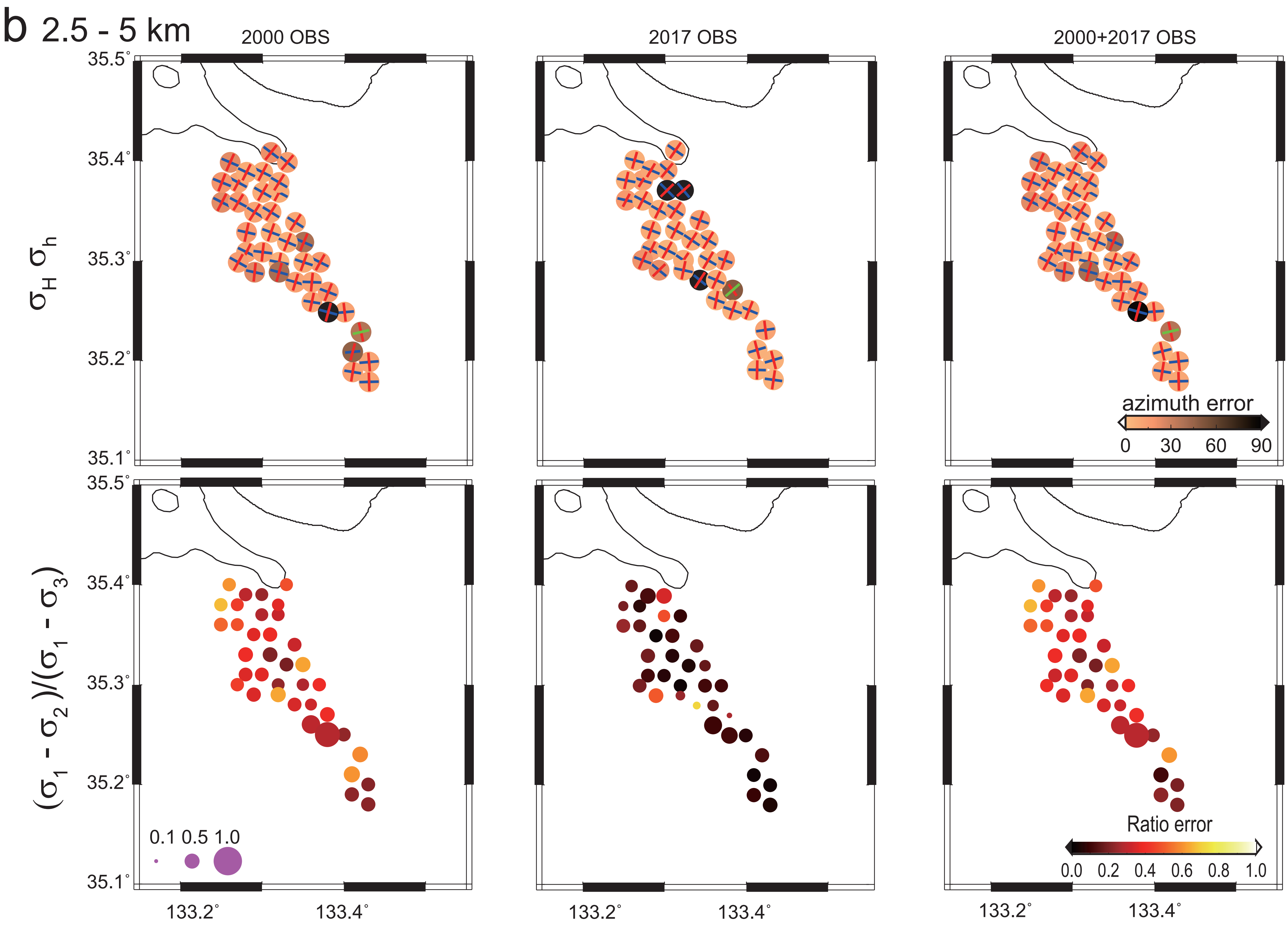

b 2.5 - 5 km
2000 OBS
2017 OBS
2000+2017 OBS
σH σh
(σ1 - σ2)/(σ1 - σ3)
35.5°
35.4°
35.3°
35.2°
35.1°
133.2°
133.4°
azimuth error
0
30
60
90
Ratio error
0.0
0.2
0.4
0.6
0.8
1.0
0.1
0.5
1.0

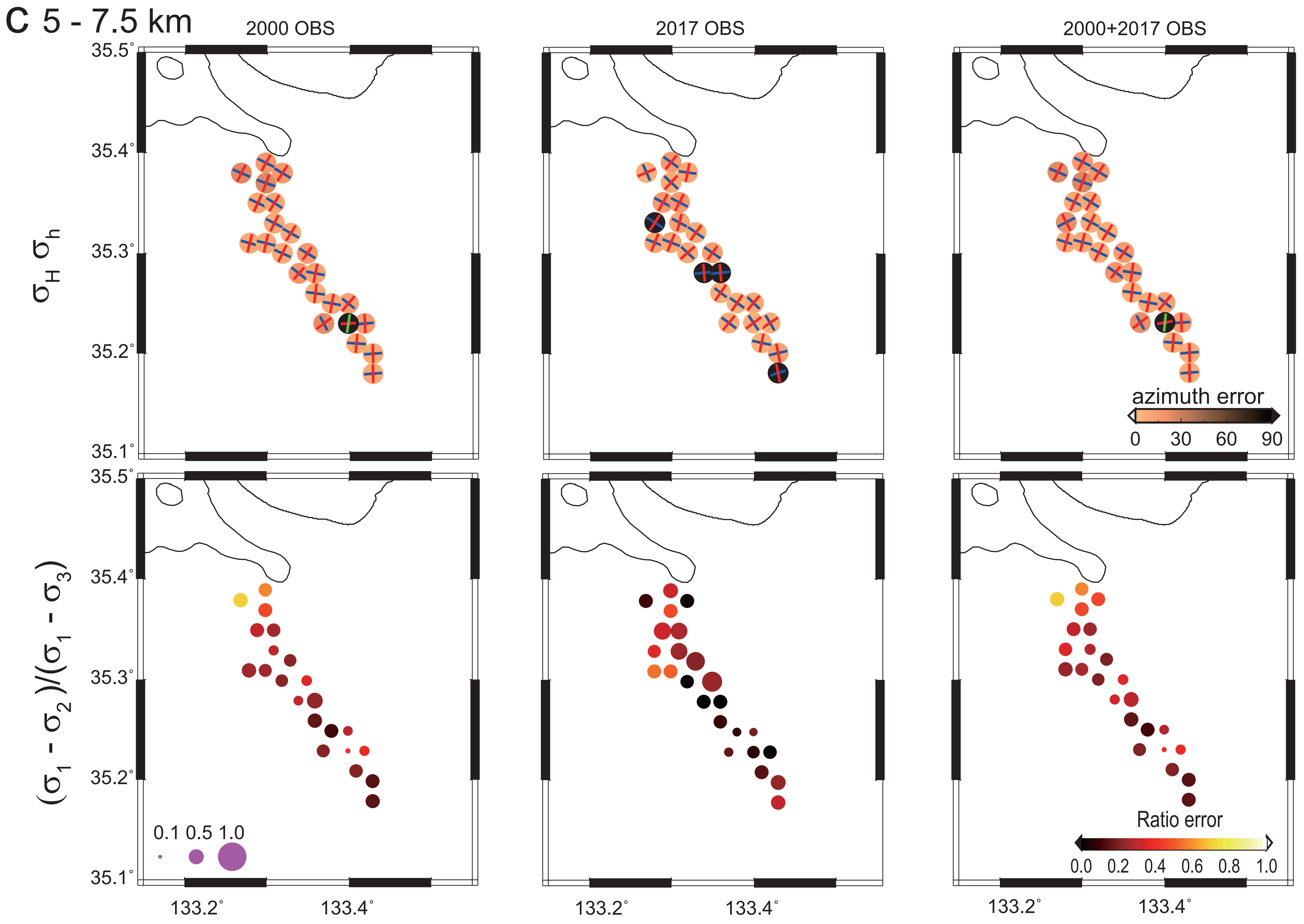

C 5 - 7.5 km
2000 OBS
2017 OBS
2000+2017 OBS
σH σh
(σ1 - σ2)/(σ1 - σ3)
35.5°
35.4°
35.3°
35.2°
35.1°
133.2°
133.4°
azimuth error
0
30
60
90
Ratio error
0.0
0.2
0.4
0.6
0.8
1.0
0.1 0.5 1.0

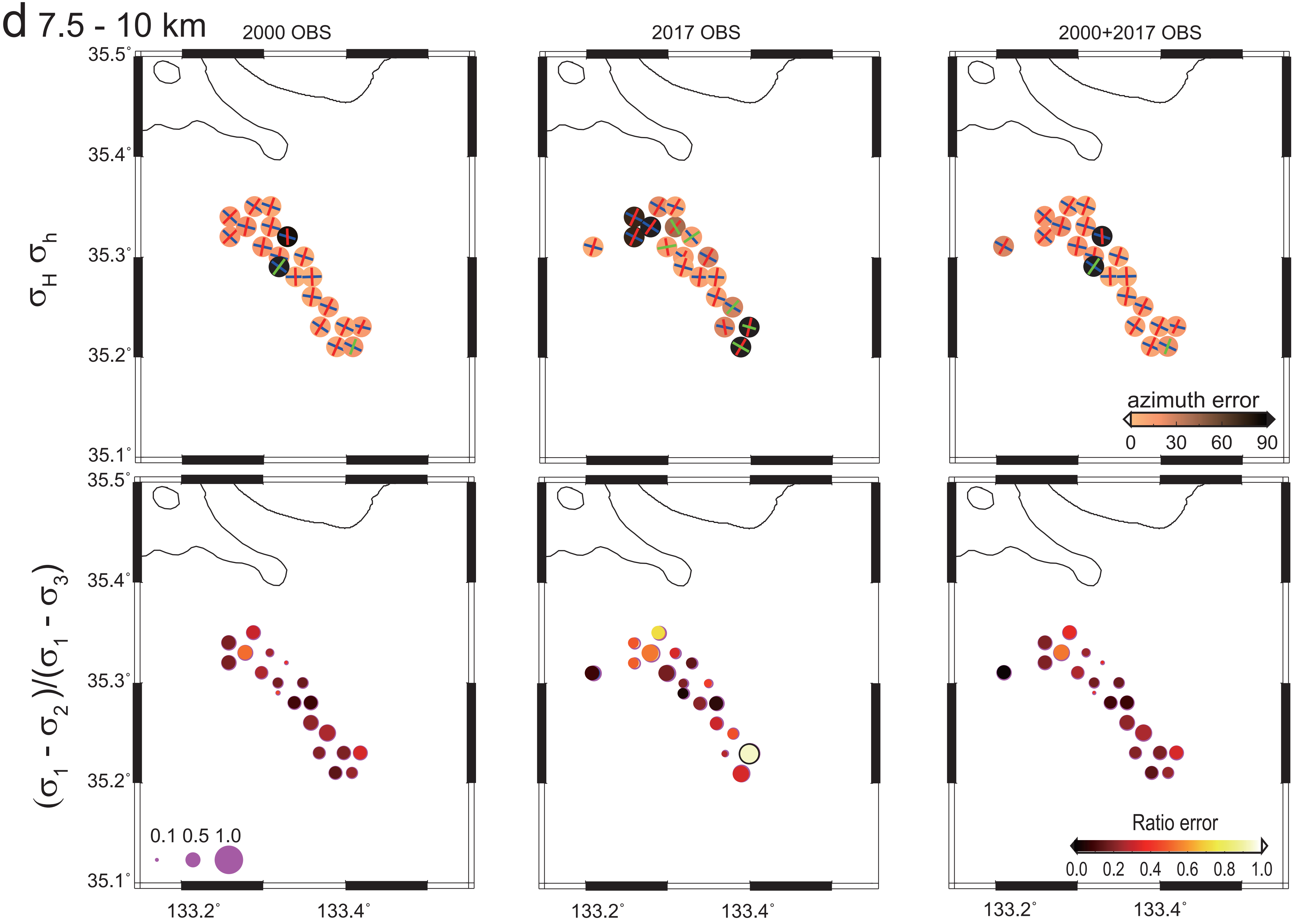
d 7.5 - 10 km
2000 OBS
2017 OBS
2000+2017 OBS
$\sigma_H\ \sigma_h$
$(\sigma_1 - \sigma_2)/(\sigma_1 - \sigma_3)$
35.5°
35.4°
35.3°
35.2°
35.1°
133.2°
133.4°
azimuth error
0 30 60 90
0.1 0.5 1.0
Ratio error
0.0 0.2 0.4 0.6 0.8 1.0

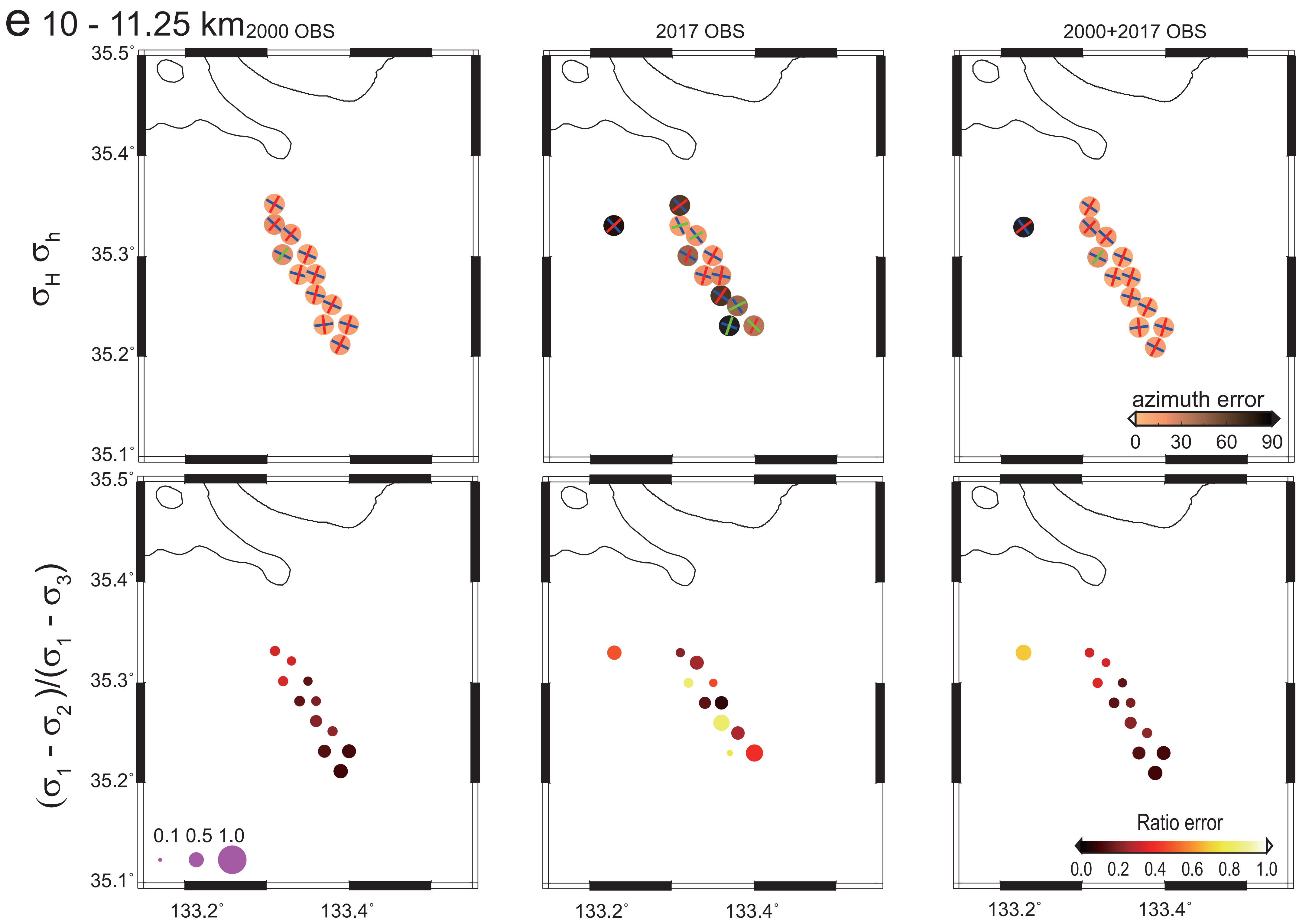

e 10 - 11.25 km
2000 OBS
2017 OBS
2000+2017 OBS
σH σh
(σ1 - σ2)/(σ1 - σ3)
35.5°
35.4°
35.3°
35.2°
35.1°
133.2°
133.4°
azimuth error
0
30
60
90
Ratio error
0.0
0.2
0.4
0.6
0.8
1.0
0.1
0.5
1.0

Supplementary Fig. 5. Estimated eigenvectors, which are two vectors close to the horizontal plane, and stress ratio (σ_2–σ_3)/(σ_1–σ_3) for each depth range.

The left, middle, and right panels show results using data from 2000 OBS, 2017 OBS, and both OBSs, respectively. In the upper panels, blue, green, and red segments correspond to the maximum, intermediate, and minimum principal axes, respectively. The brown colour covering the segment shows a 95% confidence range of the axis in the colour scale. The stress ratio value is indicated by the radius of the circle, as shown in the lower left. The colour scale at the lower right indicates the 95% confidence range. Panels **a** – **e** show results at depth range of 0 – 2.5, 2.5 – 5, 5 – 7.5, 7.5 – 10, and 10 – 12.5 km, respectively.

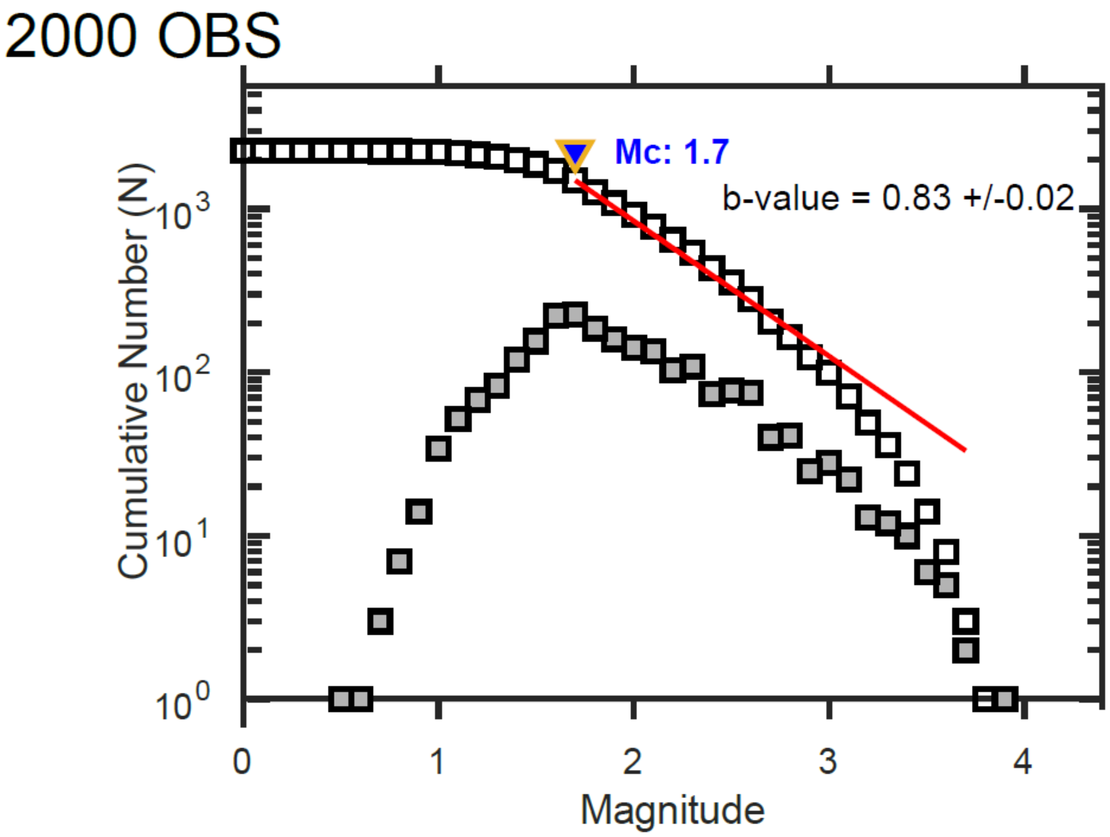

Supplementary Fig. 6. Frequency–magnitude plot for 2000 OBS data.

Mc and b-values were calculated using ZMAP. The open square shows the cumulative number from the largest magnitude range. Grey squares indicate the number of events for each magnitude range with an interval of 0.1.

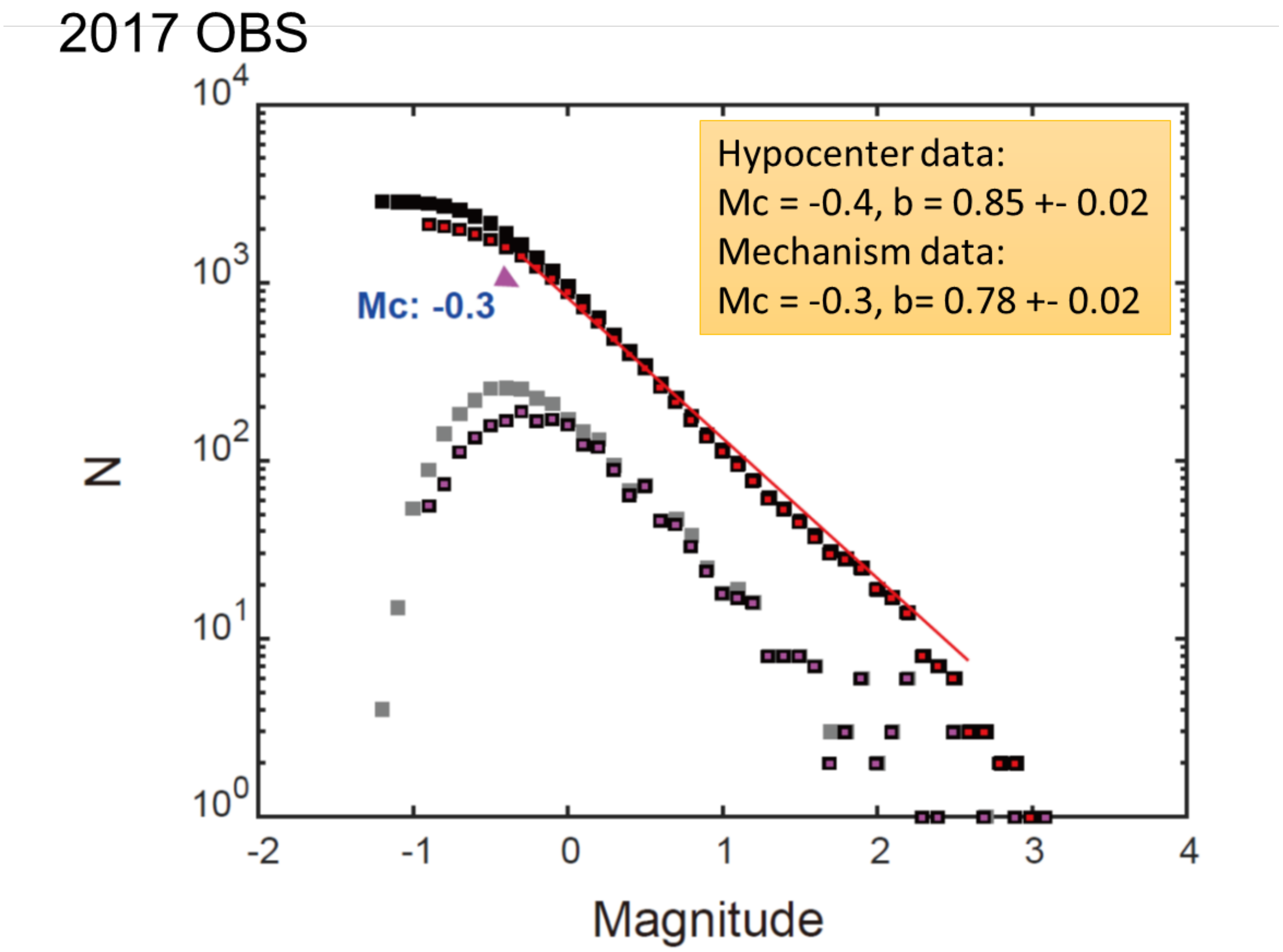

Supplementary Fig. 7. Frequency–magnitude relation for the hypocentres (black and grey) and focal mechanisms (red and pink) of 2017 OBS after event selection described in the Methods section.

Black and red symbols indicate cumulative numbers from the largest magnitude. Grey and pink show individual numbers of events within the magnitude range with an interval of 0.1. The optimum value of Mc was determined for the mechanism data using ZMAP. The red line indicates a fitting line to the mechanism data to estimate the b-value in ZMAP.

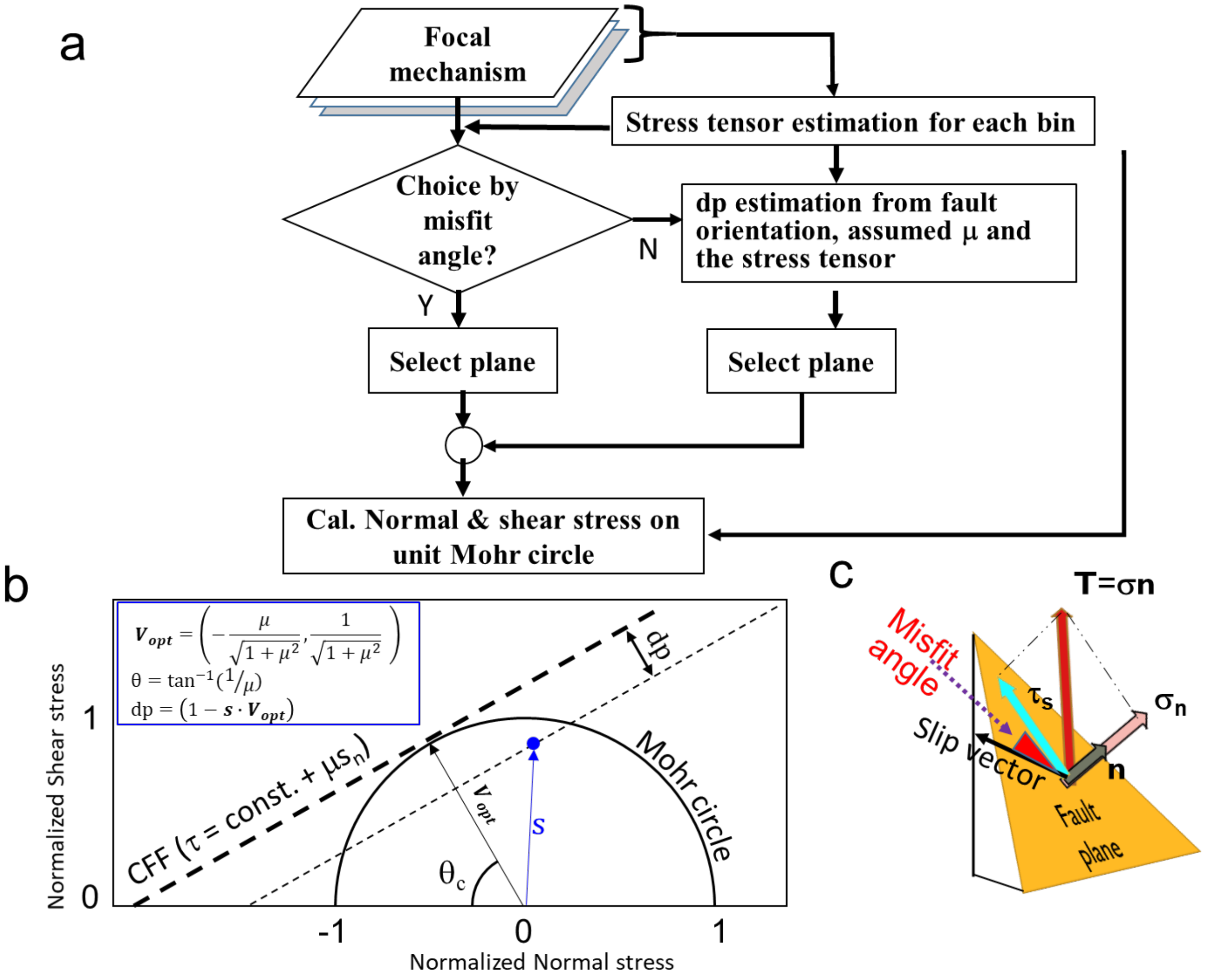

Supplementary Fig. 8. Schematic illustration of fault plane selection.

a Flow of analysis. **b** Illustration for dp estimation for an event on unit Mohr circle. **c** Misfit angle definition. A vector s in **b** can be obtained from the geometry of a fault plane and stress tensor. The optimal plane was selected based on prioritized criteria: 1) small misfit angle taking into account for estimation error, 2) small dp, where dp = (1 - S $\cdot V_{opt}$), where **S** is a vector to a point for an event's normalized normal and shear stresses from the origin, and Vopt is a vector to the touching point between unit Mohr circle and the CFF line (a vector perpendicular to the CFF line).

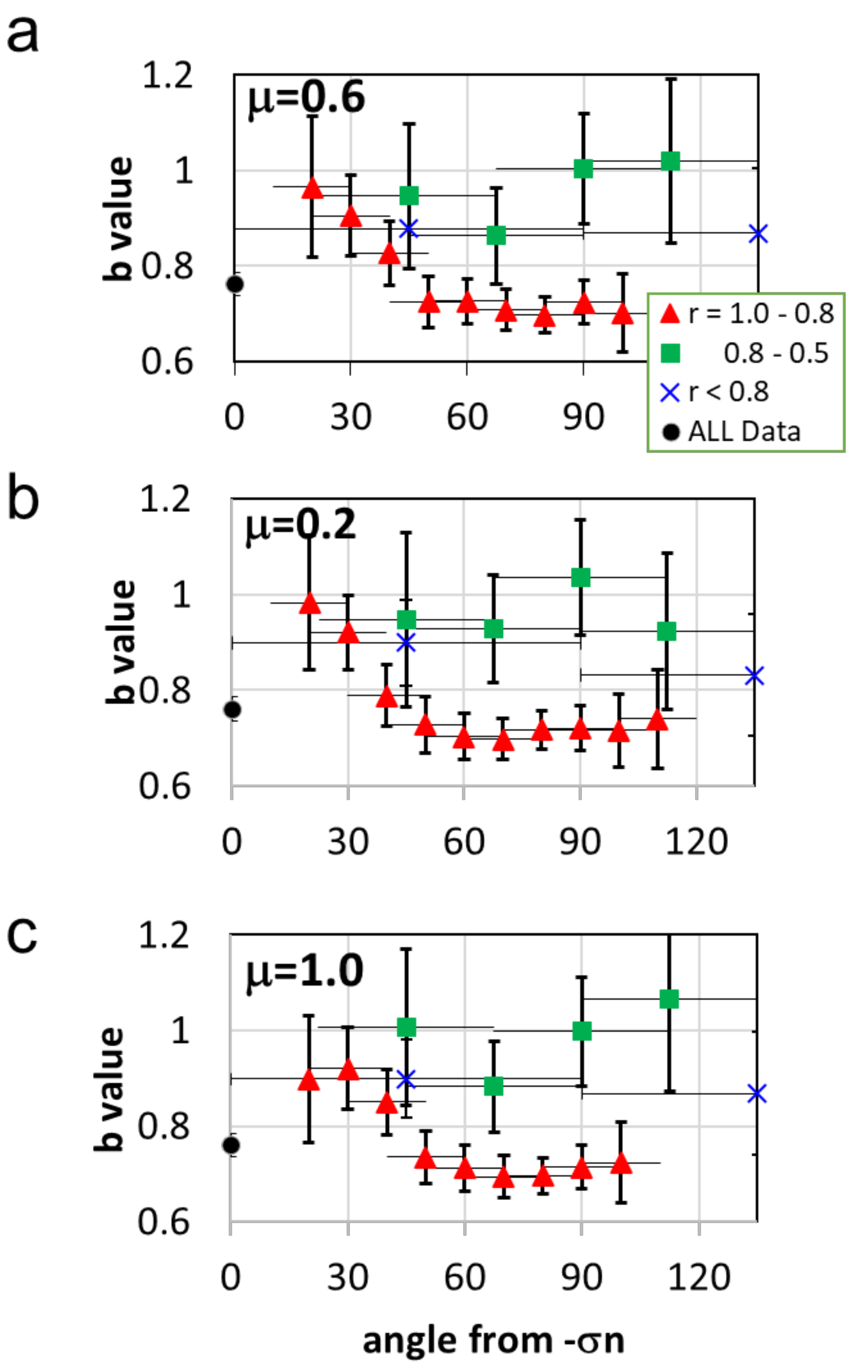

Supplementary Fig. 9. a Same as Figure 3c. **b** and **c** Same as Figure 3c but results in the cases of μ = 0.2 and μ = 1.0 for the fault plane selection in the b-value calculation, respectively. Vertical and horizontal bars indicate the standard error of the estimation and angular range on the area of the Mohr circle for estimating the b-value, respectively.

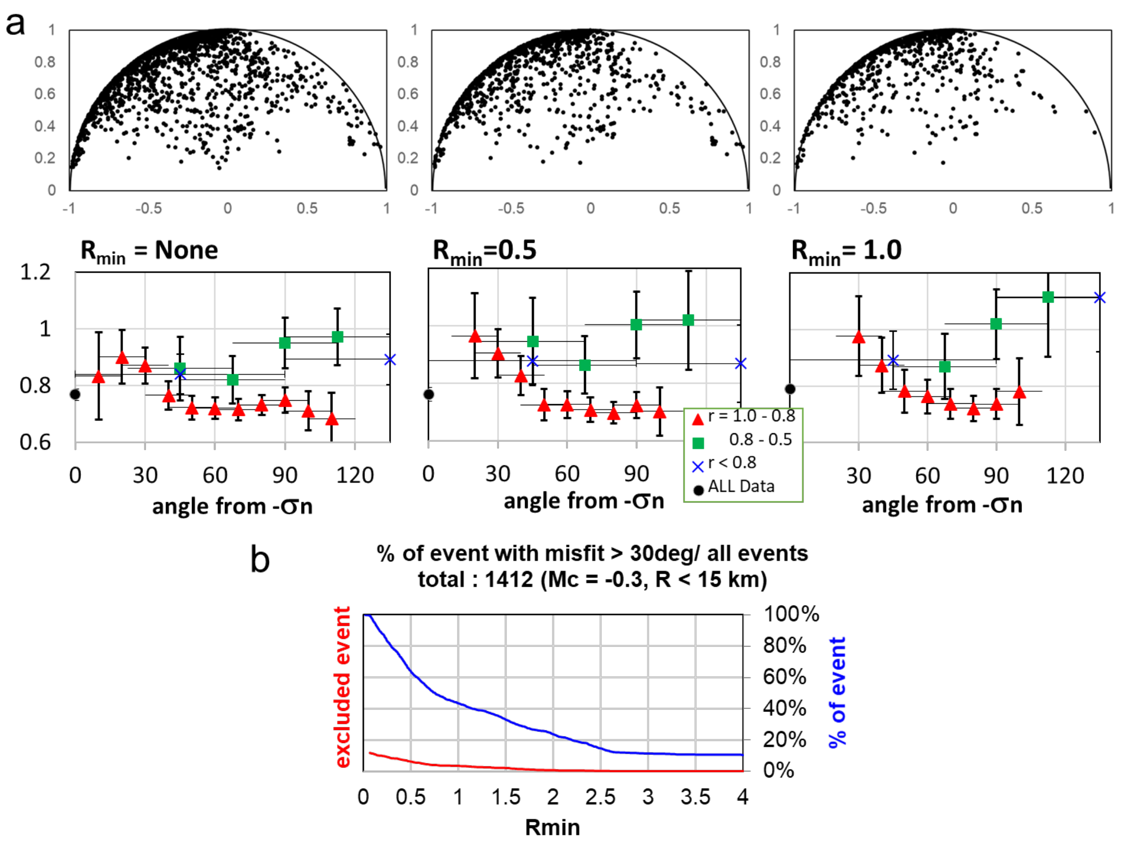

Supplementary Fig. 10. a Shear and normal stress distribution on unit Mohr circle (upper) and b-value distribution (lower) for the angle from $-\sigma_n$ (same plot as Figure 3c) under event selection based on the lower limit of distance (R_{min}) from the co-seismic fault. The left, middle, and right panels display cases for R_{min} = none, 0.5, and 1 km, respectively. The present study adopted R_{min} = 0.5 km. Vertical and horizontal bars indicate the standard error of the estimation and angular range on the area of the Mohr circle for estimating the b-value, respectively. **b** The percentages of used (blue) and excluded (red) events to the total number of events within the spatial bins, where the stress tensor was estimated. The used numbers were counted for events from R_{min} to R = 15 km. The excluded events, which were estimated with a larger misfit angle than 30° between the slip direction and the maximum shear stress

direction expected by the estimated stress tensor, were also counted from R_{min} to R = 15 km. These are plotted for R_{min} (minimum distance from fault model) change.

Supplementary Tables

Supplementary Table 1. Summary of the mainshock of the 2000 Western Tottori earthquake and two aftershock observations.

-	date/period	number of stations	Remark
Mainshock	2000/10/6	-	M7.3
2000 OBS	2000/10/15 – 11/30	59	3 compornents, for stress estimation
2017 OBS	2017/03/15 – 2018/04/30	~ 1020	1000 x UD + app. 20 x 3 components, for stress & b-value estimation

Supplementary Table 2. Average median values of error in r and q values among events containing each area in the Mohr circle. Number of events for each area is also listed. The area containing less than 50 events was not used in the analysis.

2017 OBS

r range		angle range		no. of data	Error			
r1	r2	θ1	θ2		dr_{av}	dr_{med}	$d\theta_{av}$	$d\theta_{med}$
0.8	1	0	20	56	0.035	0.022	28.977	25.55
0.8	1	10	30	151	0.037	0.021	24.969	18.53
0.8	1	20	40	297	0.051	0.028	24.252	18.72
0.8	1	30	50	393	0.057	0.035	24.382	19.89
0.8	1	40	60	457	0.059	0.038	25.22	23.23
0.8	1	50	70	550	0.057	0.036	25.06	23.305
0.8	1	60	80	638	0.056	0.04	23.055	20.485
0.8	1	70	90	722	0.058	0.041	20.549	16.79
0.8	1	80	100	465	0.07	0.05	20.511	15.87
0.8	1	90	110	128	0.129	0.114	29.044	25.04
0.8	1	100	120	51	0.163	0.141	42.243	44.13
0.5	0.8	0	45	24	0.187	0.109	21.698	16.57
0.5	0.8	22.5	67.5	102	0.311	0.301	27.796	26.62
0.5	0.8	45	90	167	0.331	0.314	28.354	26.73
0.5	0.8	67.5	112.5	175	0.282	0.235	28.763	25.1
0.5	0.8	90	135	132	0.249	0.219	29.846	26.73
0	0.8	0	90	248	0.369	0.359	31.616	27.595
0	0.8	90	180	169	0.303	0.24	32.573	28.57
