# Supplementary material for: Strength dependency of frequency–magnitude distribution in earthquakes and implications for stress state criticality
Source: Nat Commun. 2024 Jun 11;15:4957. doi: 10.1038/s41467-024-49422-7 (PMC11166660; doi:10.1038/s41467-024-49422-7)
Supplement: Supplementary file 3 — Description of Additional Supplementary Files [file 41467_2024_49422_MOESM3_ESM.pdf]

Description of Additional Supplementary Files

File Name: Supplementary Data 1
Description: Panels of Epicentre distribution. P- and T-axes distribution, Stress ratio, and Optimal directions of σ1, σ2, and σ3 for every spatial bin stress estimated.
