# Supplementary material for: Strength dependency of frequency–magnitude distribution in earthquakes and implications for stress state criticality
Source: Nat Commun. 2024 Jun 11;15:4957. doi: 10.1038/s41467-024-49422-7 (PMC11166660; doi:10.1038/s41467-024-49422-7)
Supplement: Supplementary file 4 — Supplementary Data 1 [file 41467_2024_49422_MOESM4_ESM.pdf]

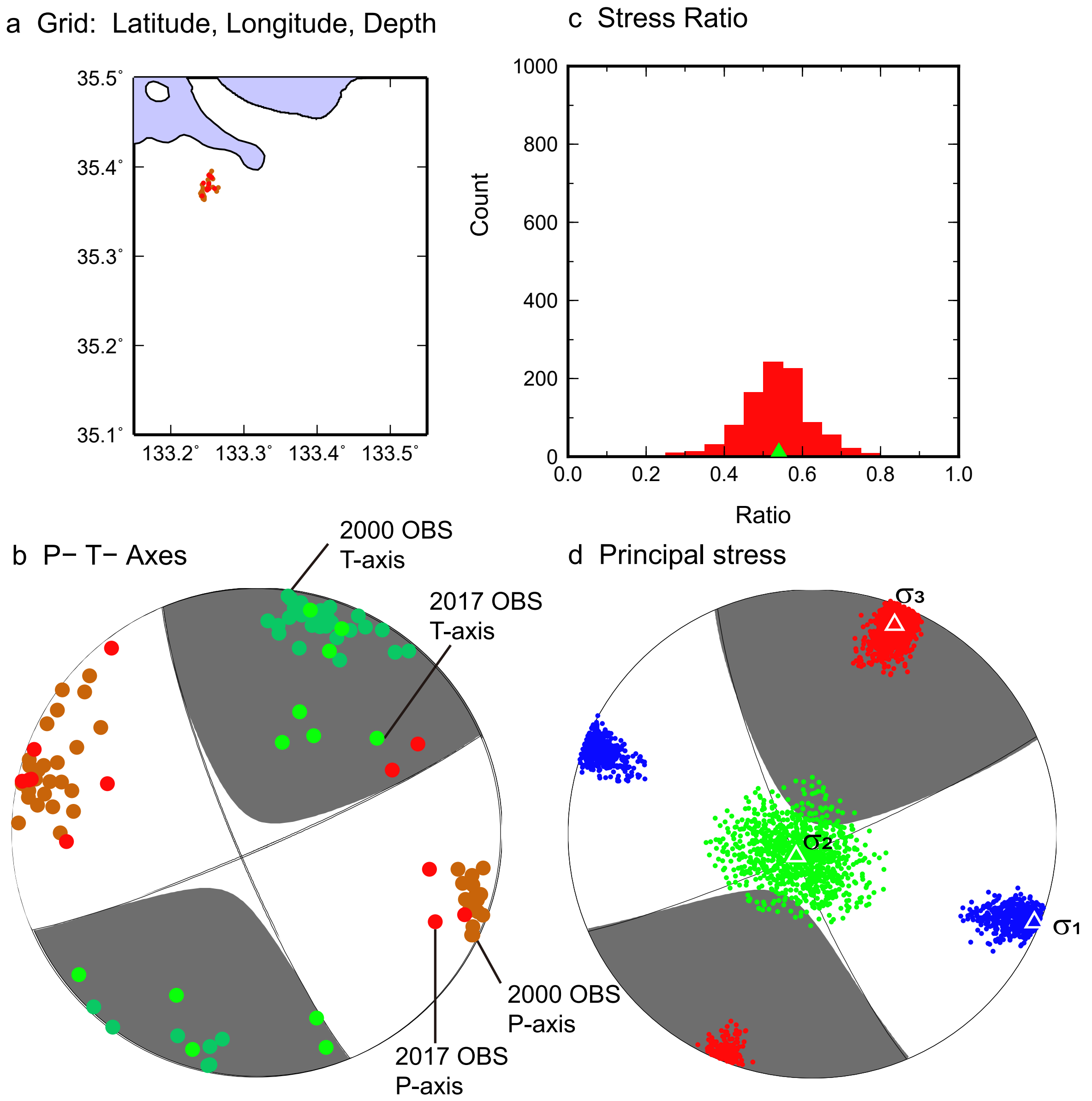

Figure legend. An example of panels in following pages. Data and parameters in stress field estimation at each spatial bin.
A) Epicentre distribution. Dark and bright colours displalocation of event in 2000 OBS and 2017 OBS, respectively.
B) P- and T- axis distribution for events plotted in lower hemisphere. Dark and Light colours indicate data in 2000 OBS and 2017 OBS, respectively. Green and Red colours correspond to P- and T- axes.
C) Stress ratio optimal value and its 95 % confidense range are indicated by triangle and red bars. Hight of the bar is frequency distribution in the ratio range with 0.02 interval.
D) optimal directions of σ1, σ2, and σ3 are plotted by blue green, and red triangles on the stress tensor plotted in lower hemisphere. Shaded area indicates tension. 95% confidense ranges for σ1,σ2, and σ3 are shown by coloured dots.
Following pages are plots for every spatial bins stress estimated.

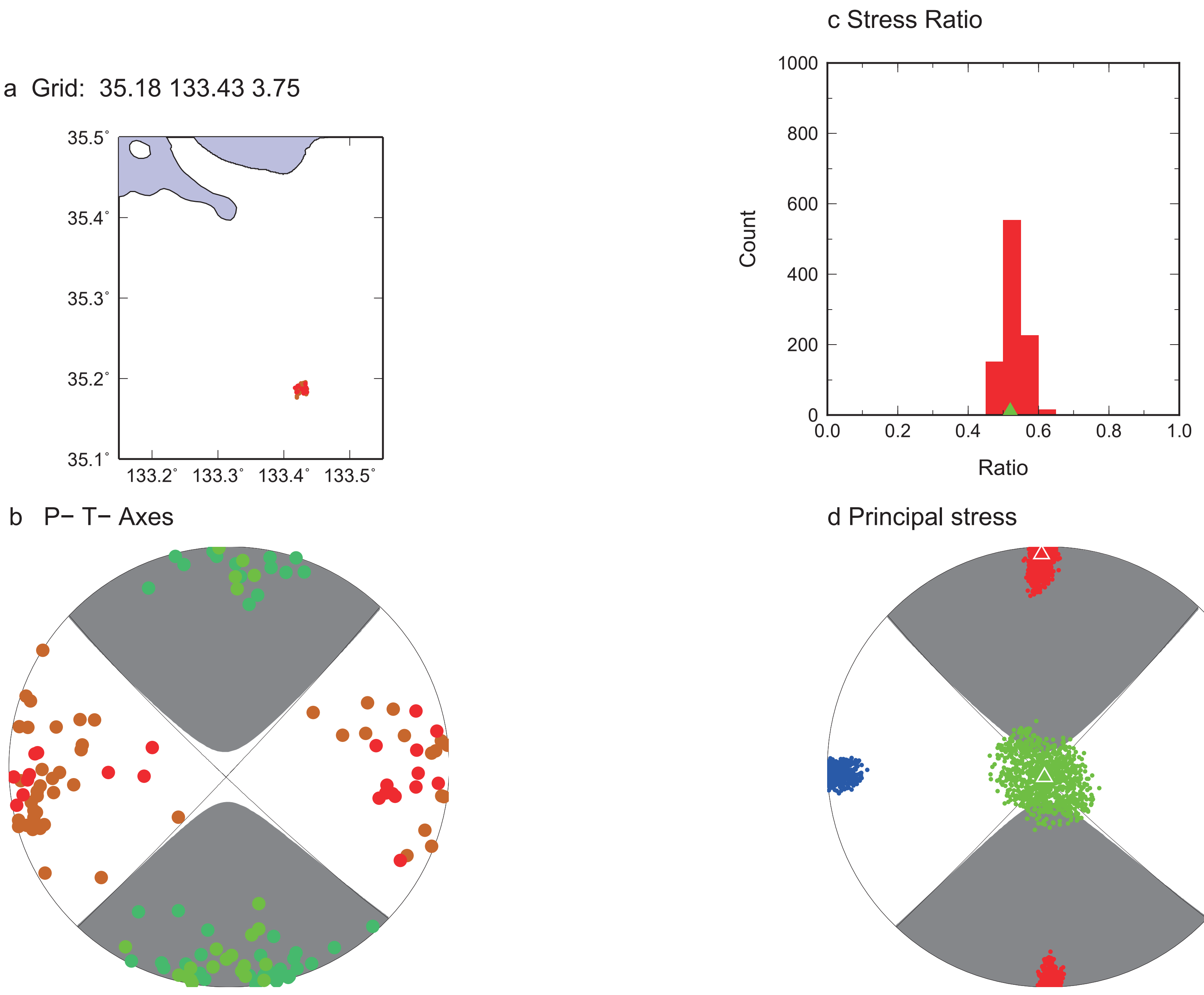

a Grid: 35.18 133.43 3.75
35.5˚
35.4˚
35.3˚
35.2˚
35.1˚
133.2˚
133.3˚
133.4˚
133.5˚
b P− T− Axes
c Stress Ratio
Count
1000
800
600
400
200
0
0.0
0.2
0.4
0.6
0.8
1.0
Ratio
d Principal stress

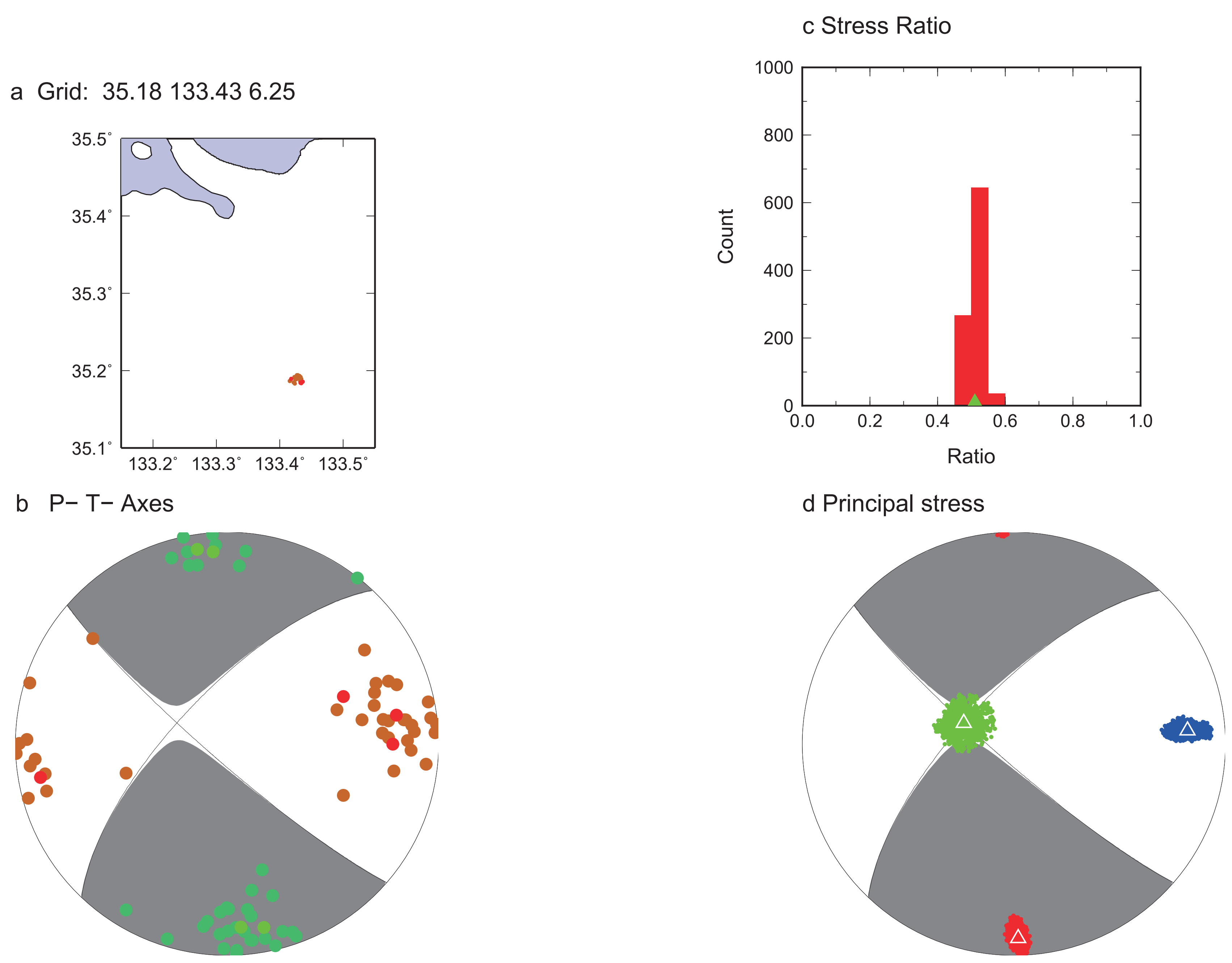
a Grid: 35.18 133.43 6.25
35.5˚
35.4˚
35.3˚
35.2˚
35.1˚
133.2˚
133.3˚
133.4˚
133.5˚
c Stress Ratio
Count
1000
800
600
400
200
0
0.0
0.2
0.4
0.6
0.8
1.0
Ratio
b P− T− Axes
d Principal stress

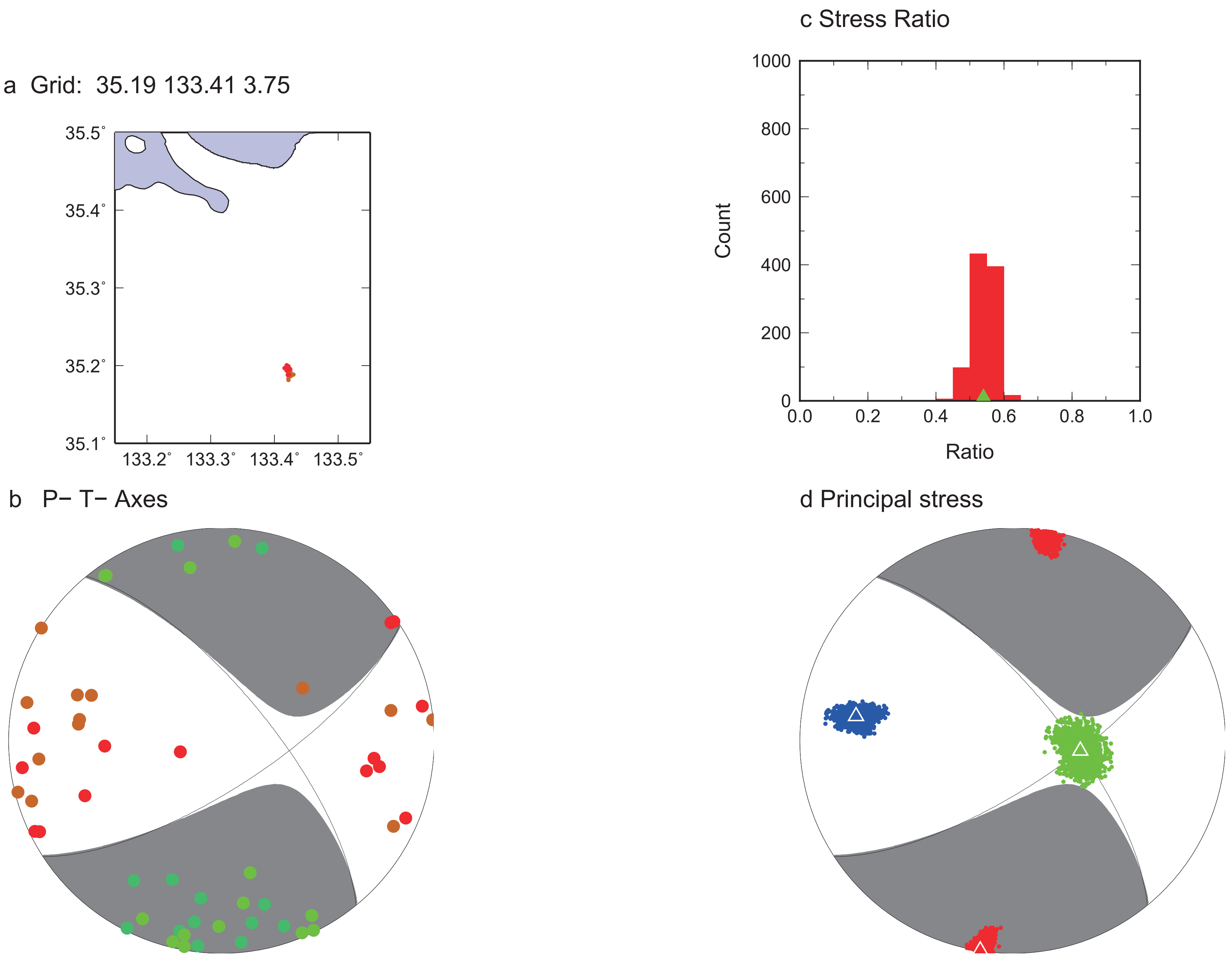

a Grid: 35.19 133.41 3.75
35.5˚
35.4˚
35.3˚
35.2˚
35.1˚
133.2˚
133.3˚
133.4˚
133.5˚
c Stress Ratio
Count
1000
800
600
400
200
0
0.0
0.2
0.4
0.6
0.8
1.0
Ratio
b P− T− Axes
d Principal stress

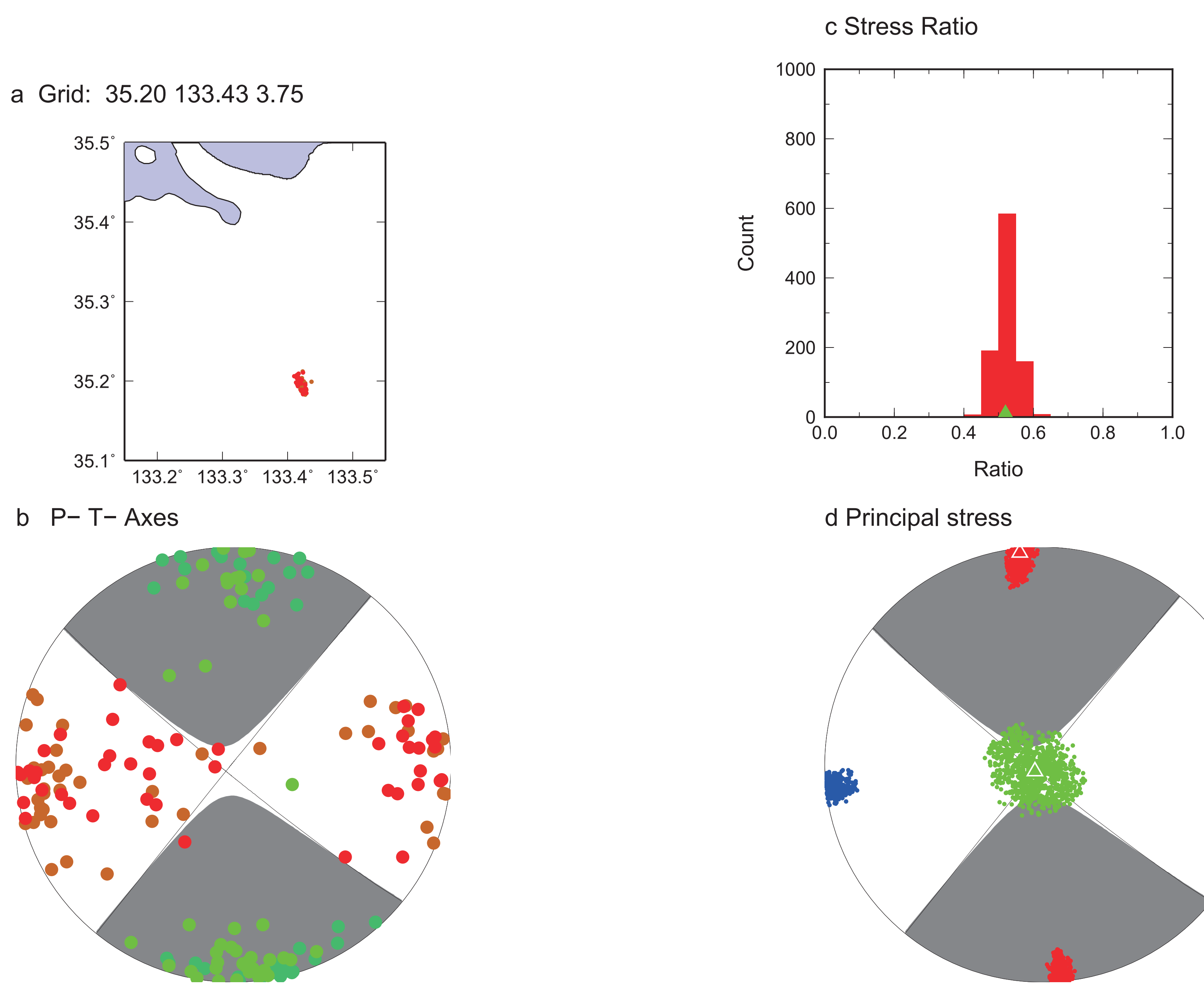

a Grid: 35.20 133.43 3.75
35.5˚
35.4˚
35.3˚
35.2˚
35.1˚
133.2˚
133.3˚
133.4˚
133.5˚
c Stress Ratio
Count
1000
800
600
400
200
0
0.0
0.2
0.4
0.6
0.8
1.0
Ratio
b P− T− Axes
d Principal stress

a Grid: 35.20 133.43 6.25

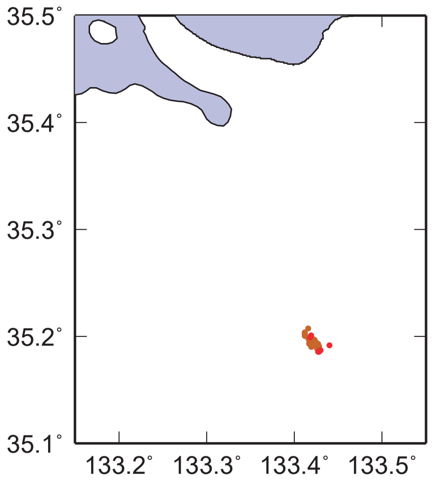

c Stress Ratio

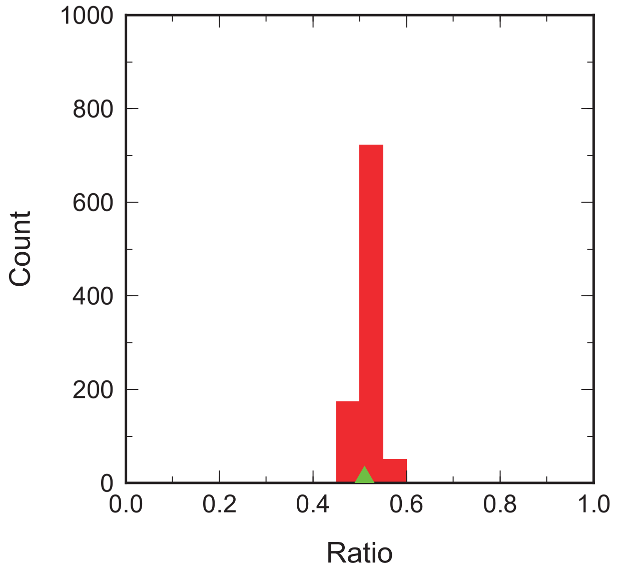

b P− T− Axes

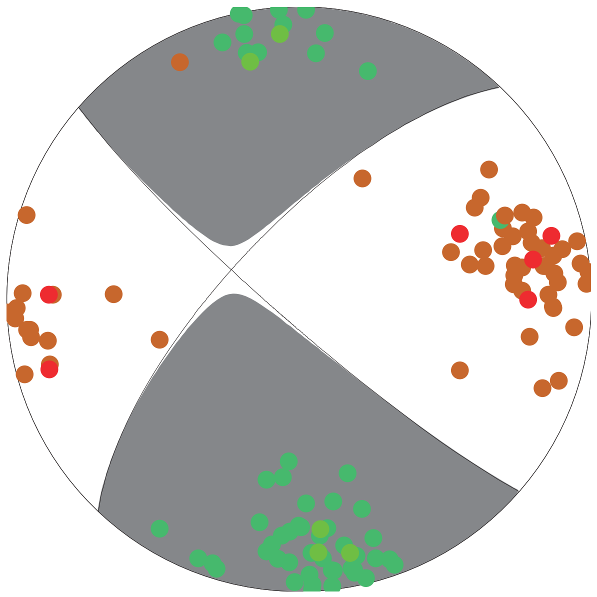

d Principal stress

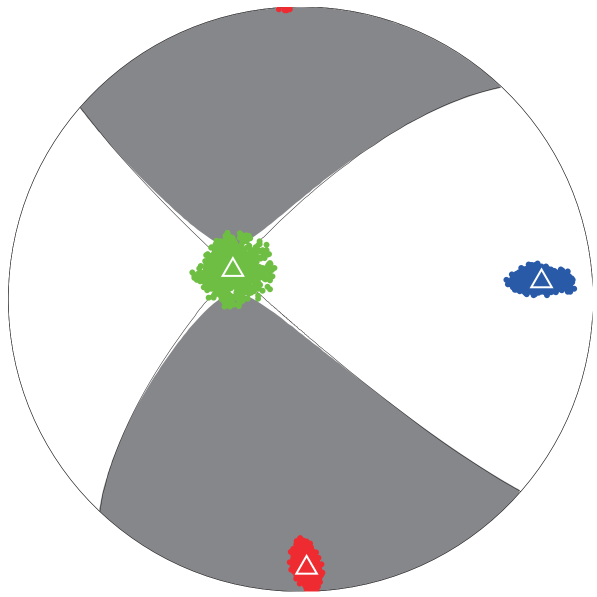

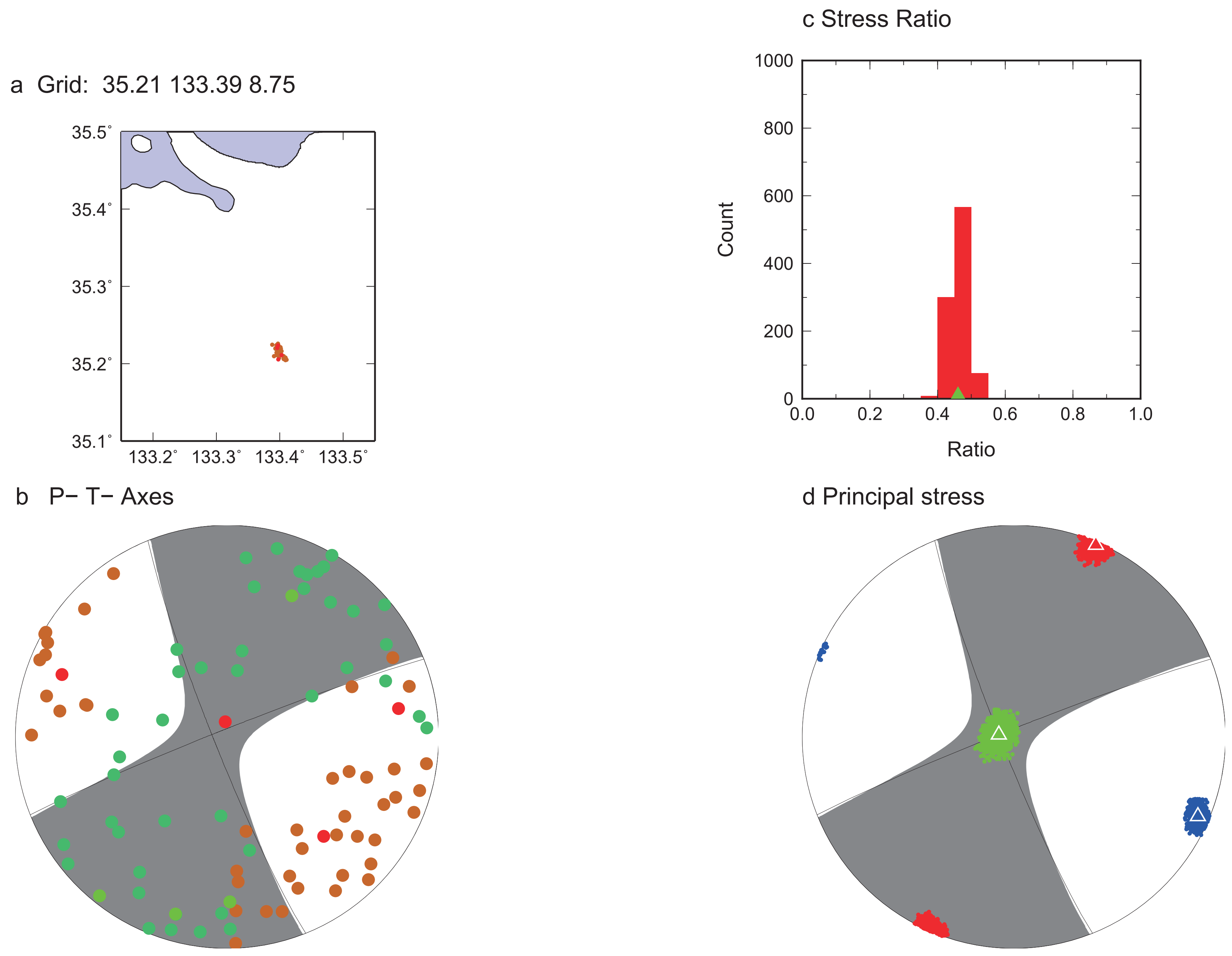
a Grid: 35.21 133.39 8.75
35.5˚
35.4˚
35.3˚
35.2˚
35.1˚
133.2˚
133.3˚
133.4˚
133.5˚
b P− T− Axes
c Stress Ratio
1000
800
600
400
200
0
Count
0.0
0.2
0.4
0.6
0.8
1.0
Ratio
d Principal stress

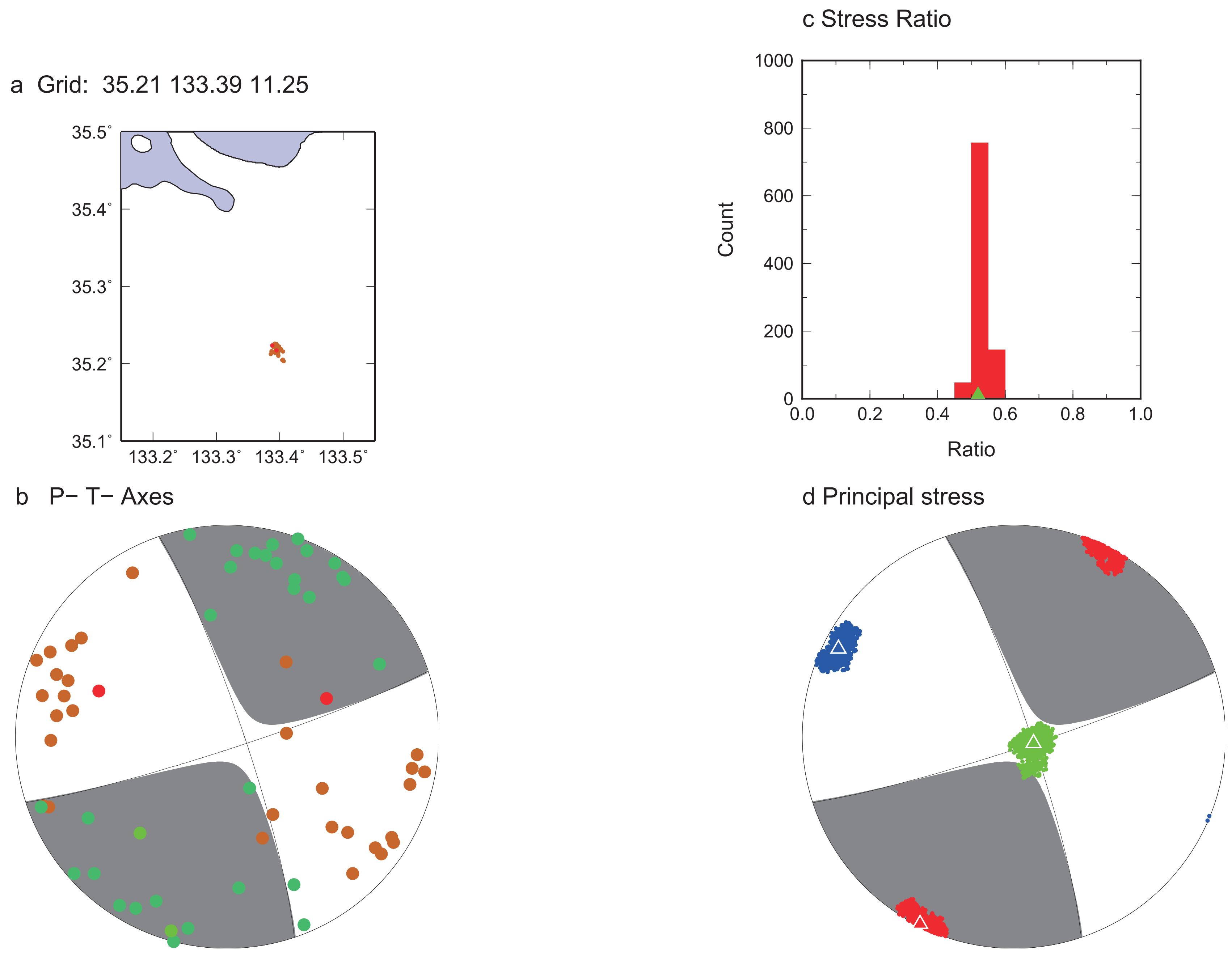

a Grid: 35.21 133.39 11.25
35.5˚
35.4˚
35.3˚
35.2˚
35.1˚
133.2˚
133.3˚
133.4˚
133.5˚
b P− T− Axes
c Stress Ratio
Count
1000
800
600
400
200
0
0.0
0.2
0.4
0.6
0.8
1.0
Ratio
d Principal stress

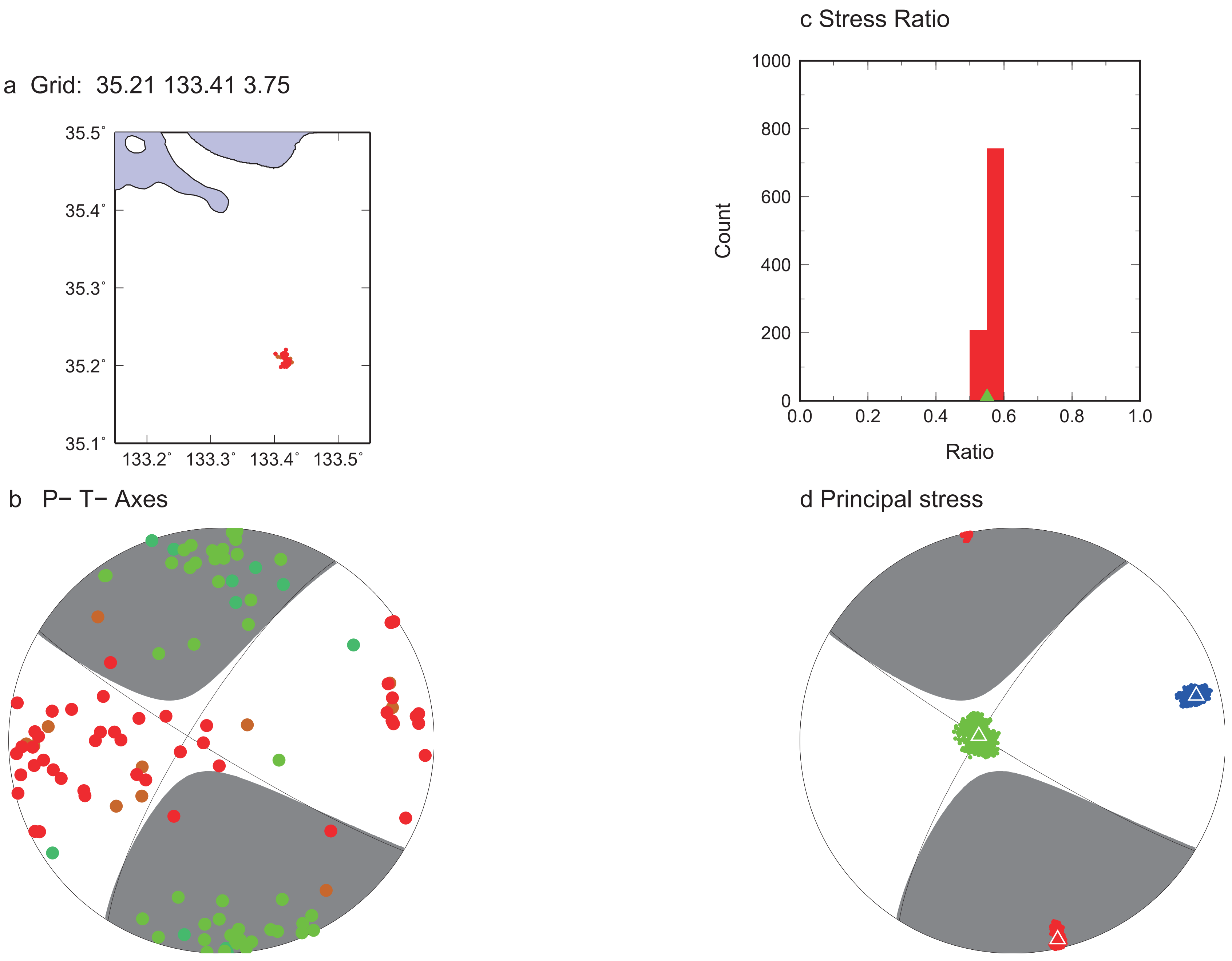
a Grid: 35.21 133.41 3.75
35.5˚
35.4˚
35.3˚
35.2˚
35.1˚
133.2˚ 133.3˚ 133.4˚ 133.5˚
c Stress Ratio
1000
800
600
400
200
0
Count
0.0 0.2 0.4 0.6 0.8 1.0
Ratio
b P− T− Axes
d Principal stress

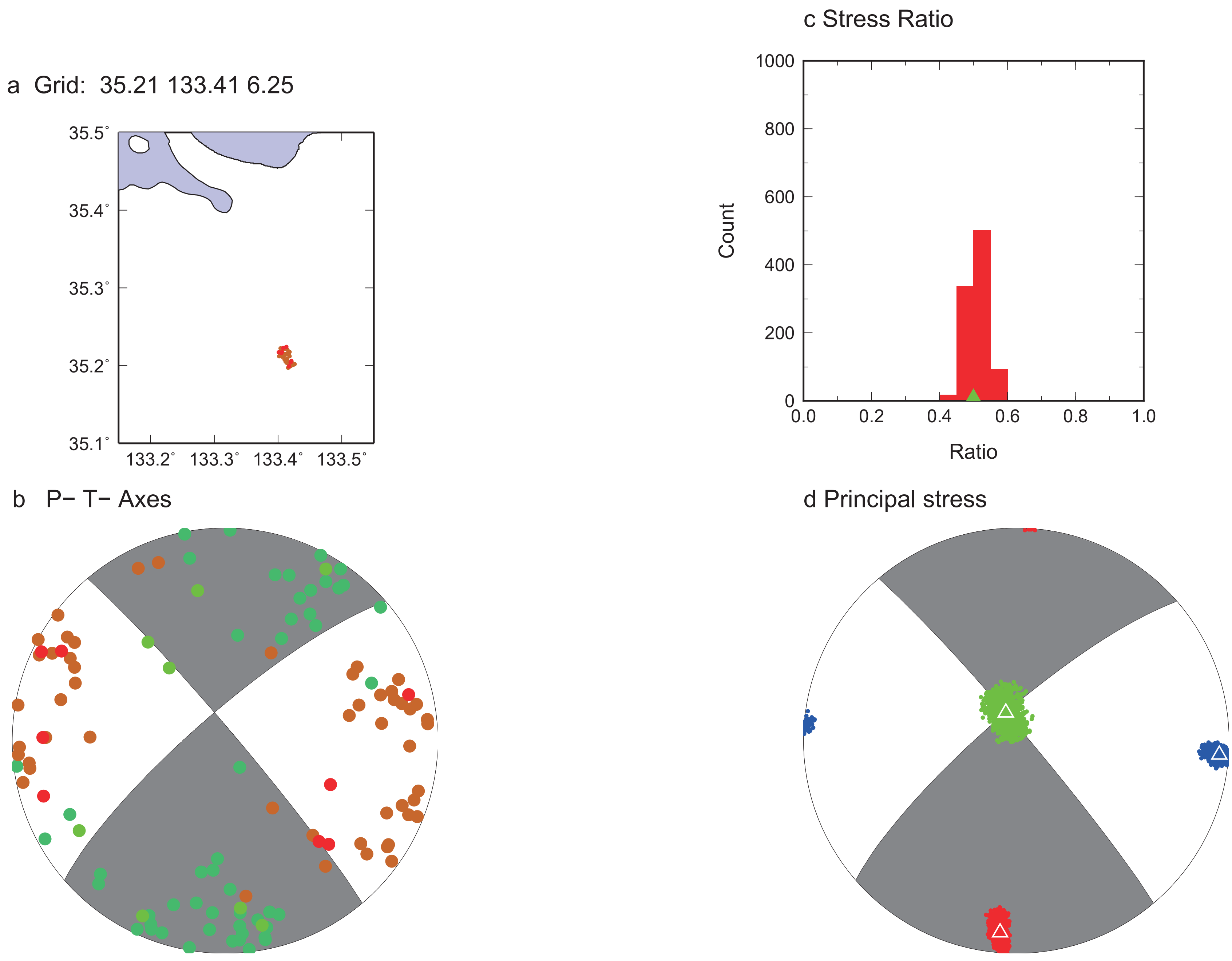
a Grid: 35.21 133.41 6.25
35.5°
35.4°
35.3°
35.2°
35.1°
133.2°
133.3°
133.4°
133.5°
b P− T− Axes
c Stress Ratio
1000
800
600
400
200
0
Count
0.0
0.2
0.4
0.6
0.8
1.0
Ratio
d Principal stress

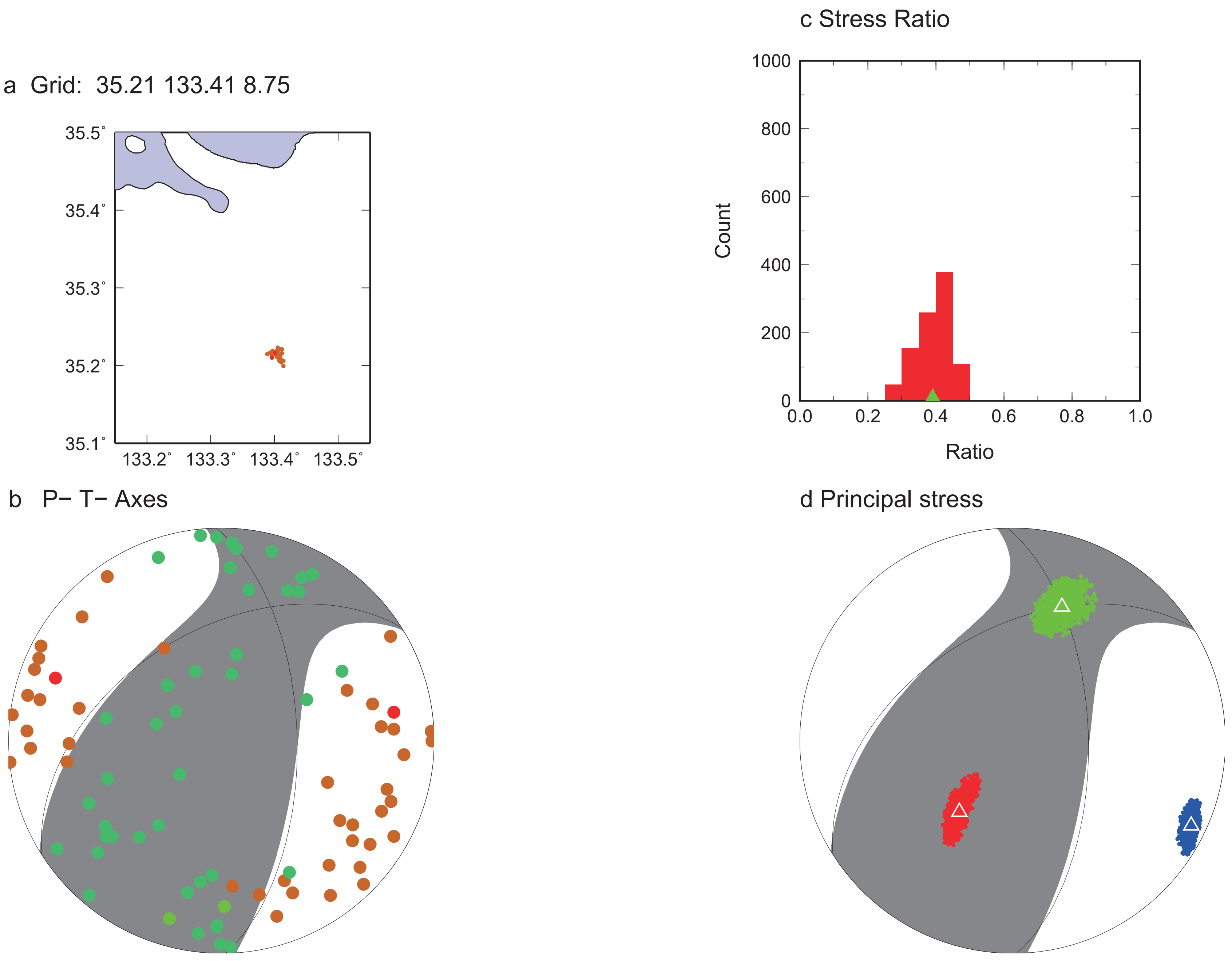

a Grid: 35.21 133.41 8.75
35.5˚
35.4˚
35.3˚
35.2˚
35.1˚
133.2˚
133.3˚
133.4˚
133.5˚
b P− T− Axes
c Stress Ratio
Count
1000
800
600
400
200
0
0.0
0.2
0.4
0.6
0.8
1.0
Ratio
d Principal stress

a Grid: 35.22 133.44 1.25

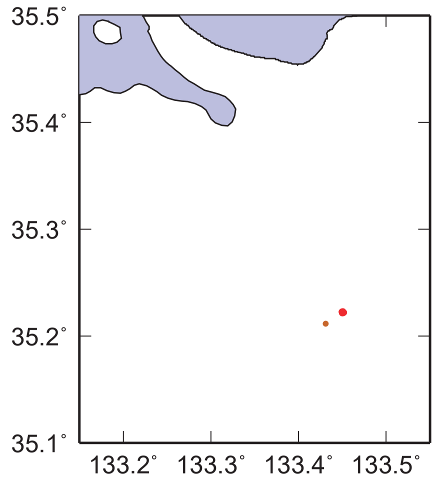

c Stress Ratio

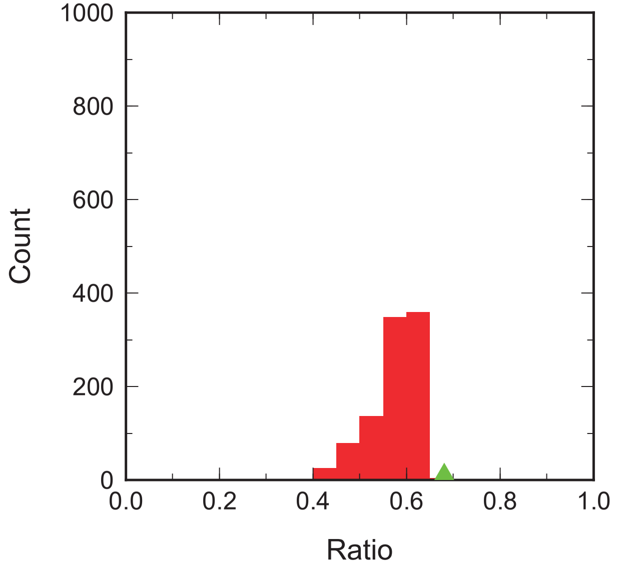

b P– T– Axes

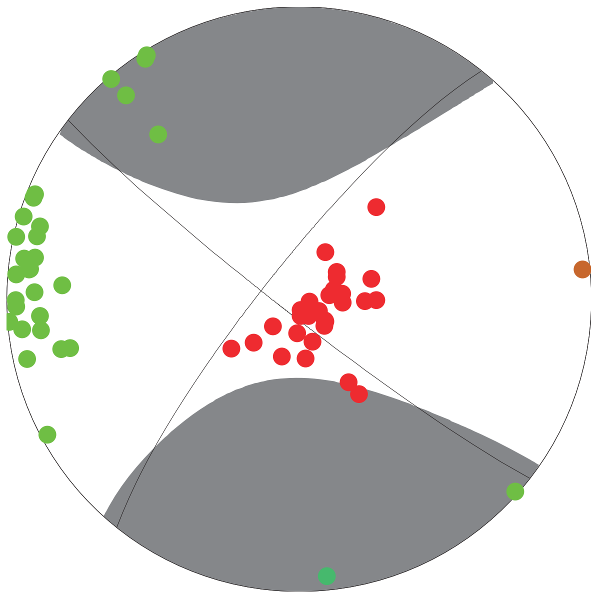

d Principal stress

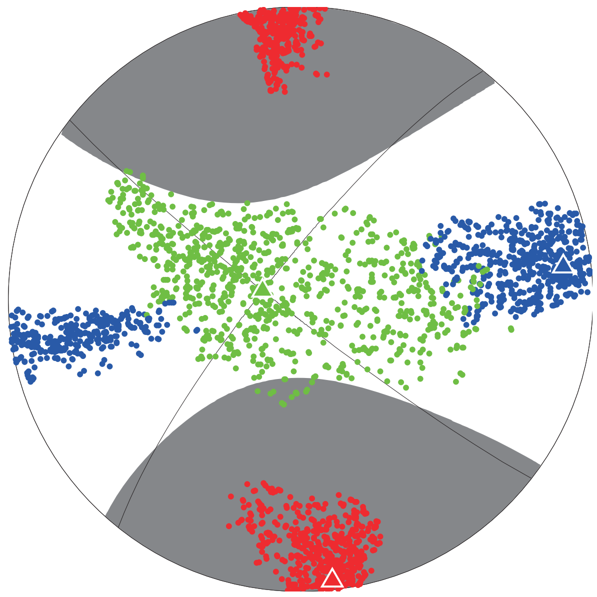

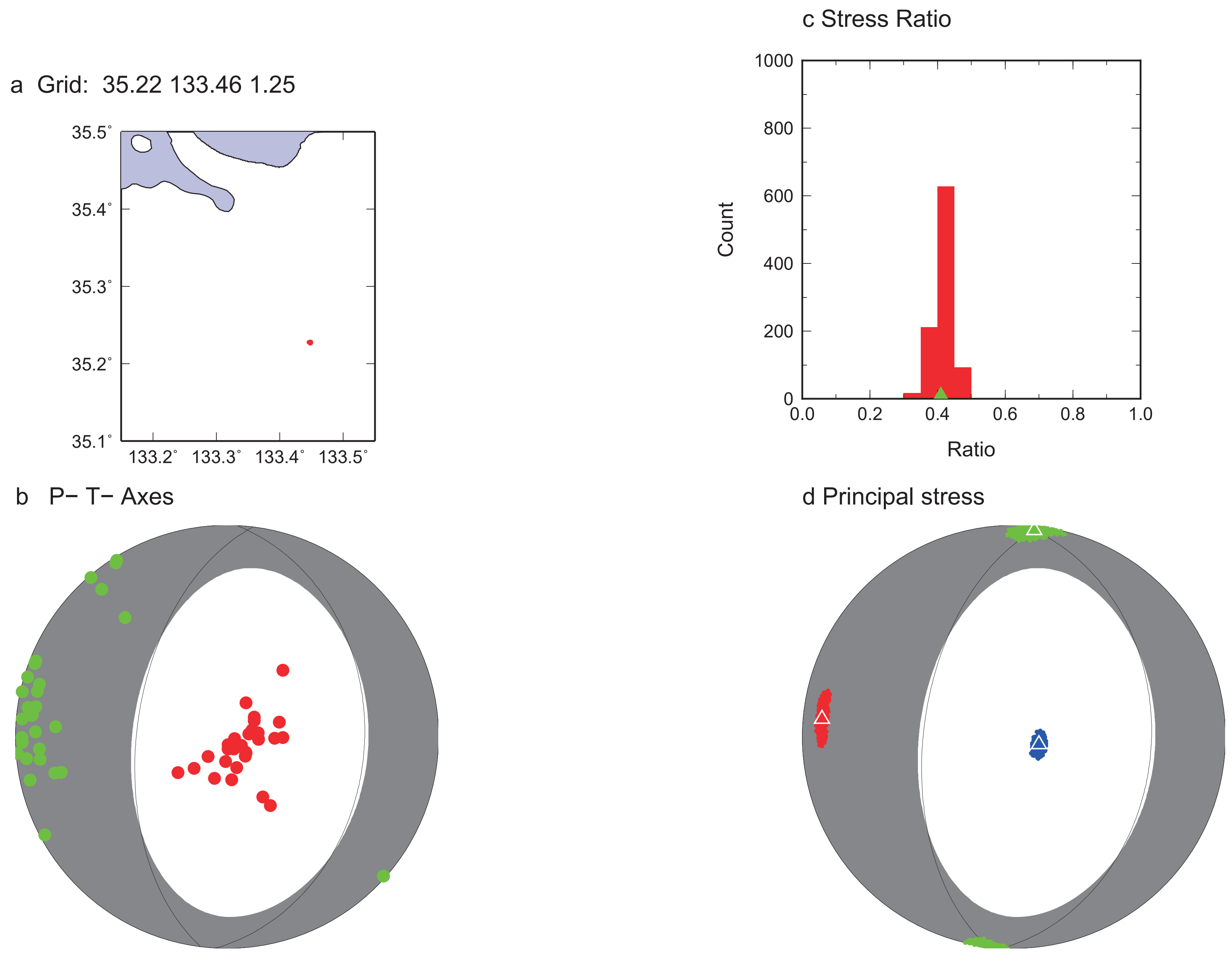
a Grid: 35.22 133.46 1.25
35.5˚
35.4˚
35.3˚
35.2˚
35.1˚
133.2˚
133.3˚
133.4˚
133.5˚
b P− T− Axes
c Stress Ratio
Count
1000
800
600
400
200
0
0.0
0.2
0.4
0.6
0.8
1.0
Ratio
d Principal stress

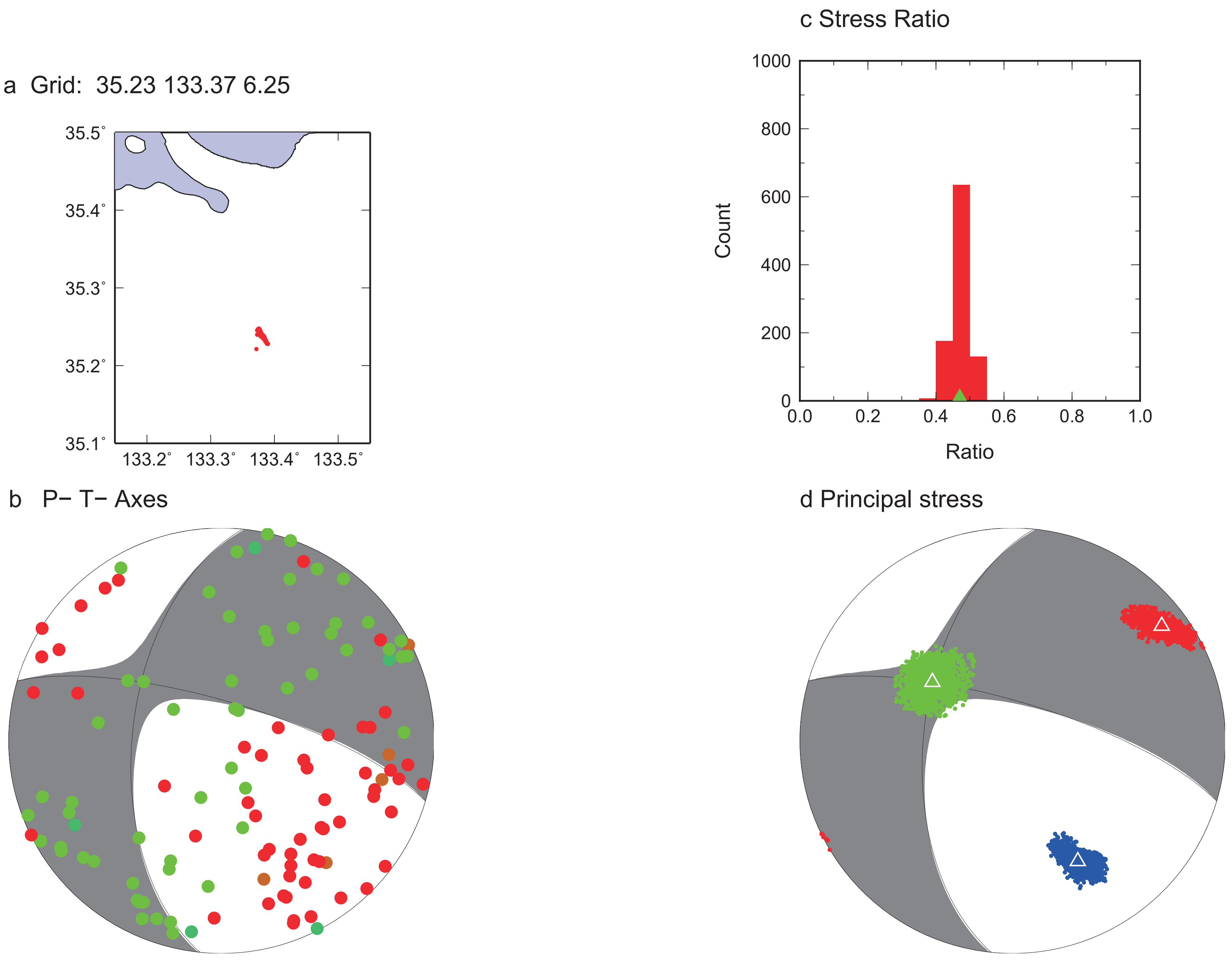
a Grid: 35.23 133.37 6.25
35.5˚
35.4˚
35.3˚
35.2˚
35.1˚
133.2˚ 133.3˚ 133.4˚ 133.5˚
b P− T− Axes
c Stress Ratio
1000
800
600
400
200
0
Count
0.0 0.2 0.4 0.6 0.8 1.0
Ratio
d Principal stress

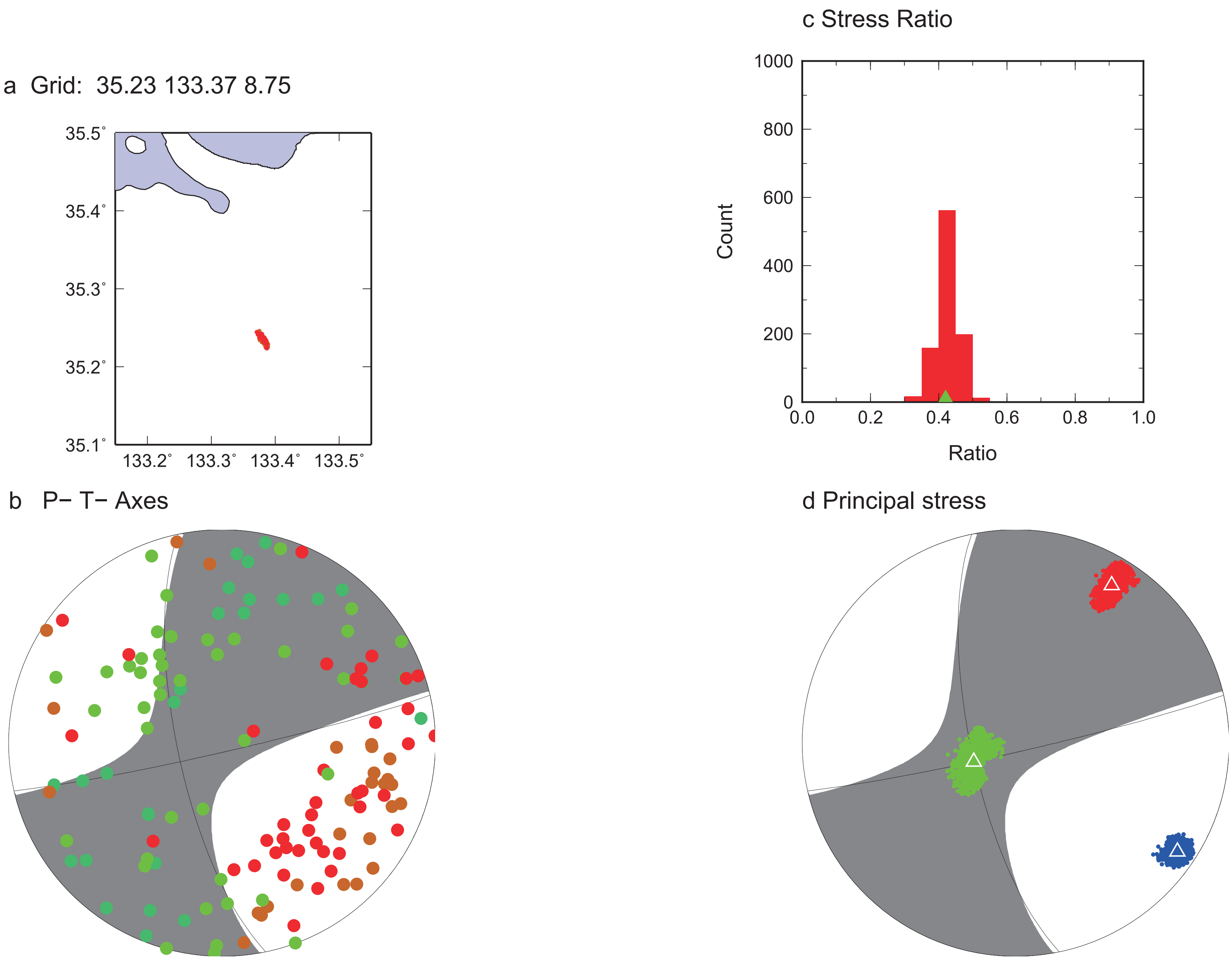
a Grid: 35.23 133.37 8.75
35.5˚
35.4˚
35.3˚
35.2˚
35.1˚
133.2˚
133.3˚
133.4˚
133.5˚
b P− T− Axes
c Stress Ratio
1000
800
600
400
200
0
Count
0.0
0.2
0.4
0.6
0.8
1.0
Ratio
d Principal stress

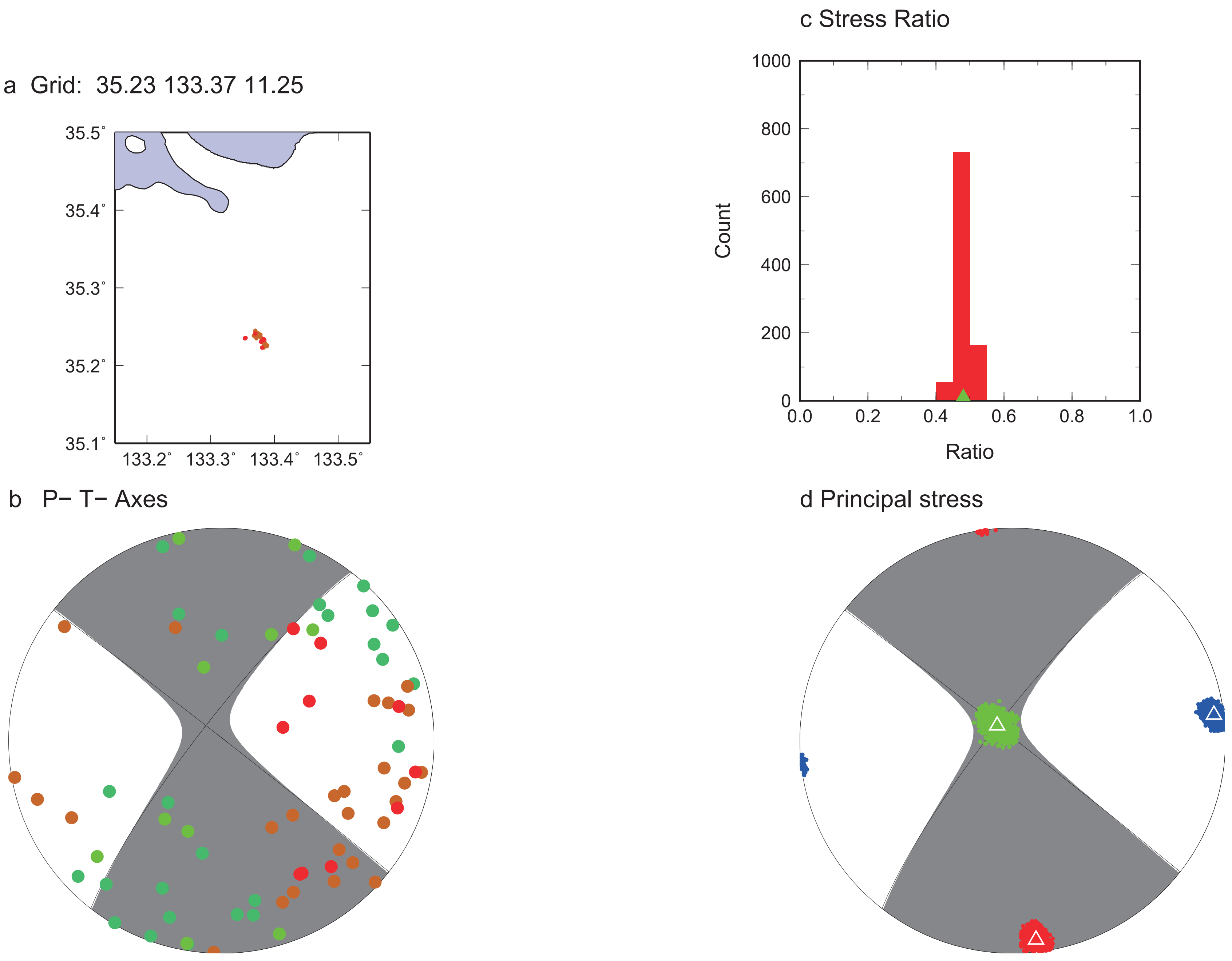
a Grid: 35.23 133.37 11.25
35.5˚
35.4˚
35.3˚
35.2˚
35.1˚
133.2˚
133.3˚
133.4˚
133.5˚
c Stress Ratio
1000
800
600
400
200
0
Count
0.0
0.2
0.4
0.6
0.8
1.0
Ratio
b P− T− Axes
d Principal stress

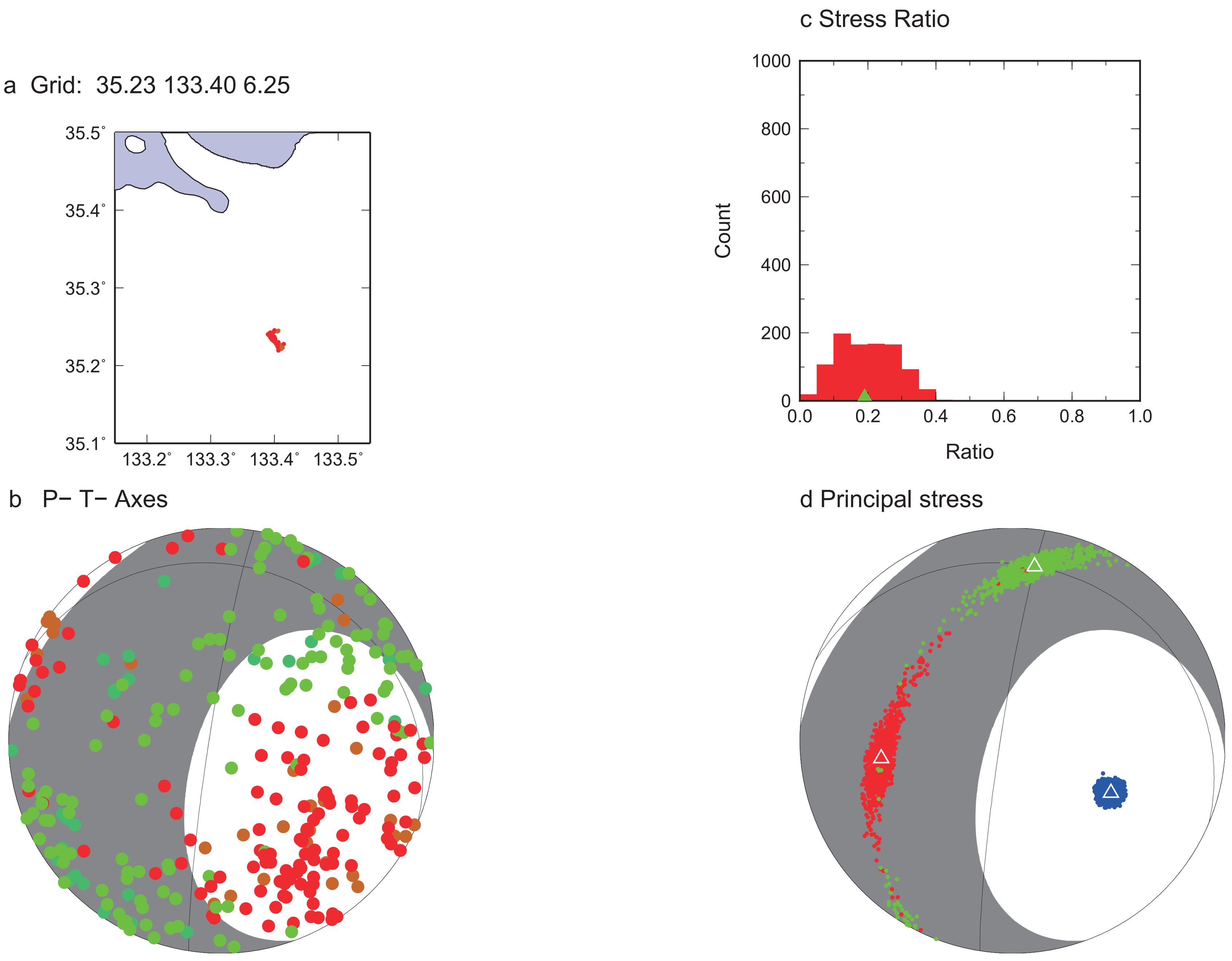
a Grid: 35.23 133.40 6.25
35.5˚
35.4˚
35.3˚
35.2˚
35.1˚
133.2˚ 133.3˚ 133.4˚ 133.5˚
b P− T− Axes
c Stress Ratio
Count
1000
800
600
400
200
0
0.0 0.2 0.4 0.6 0.8 1.0
Ratio
d Principal stress

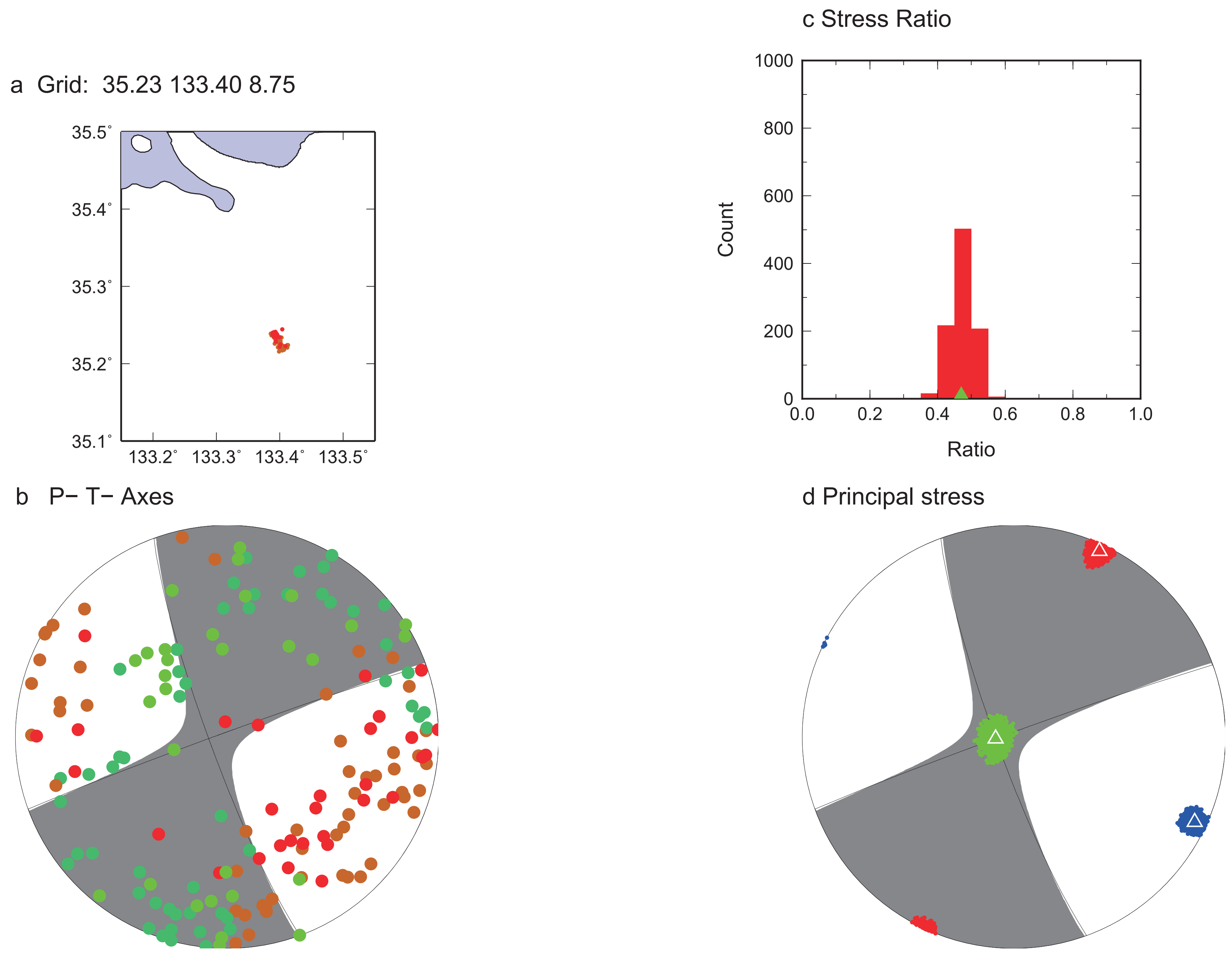
a Grid: 35.23 133.40 8.75
35.5˚
35.4˚
35.3˚
35.2˚
35.1˚
133.2˚
133.3˚
133.4˚
133.5˚
b P− T− Axes
c Stress Ratio
1000
800
600
400
200
0
Count
0.0
0.2
0.4
0.6
0.8
1.0
Ratio
d Principal stress

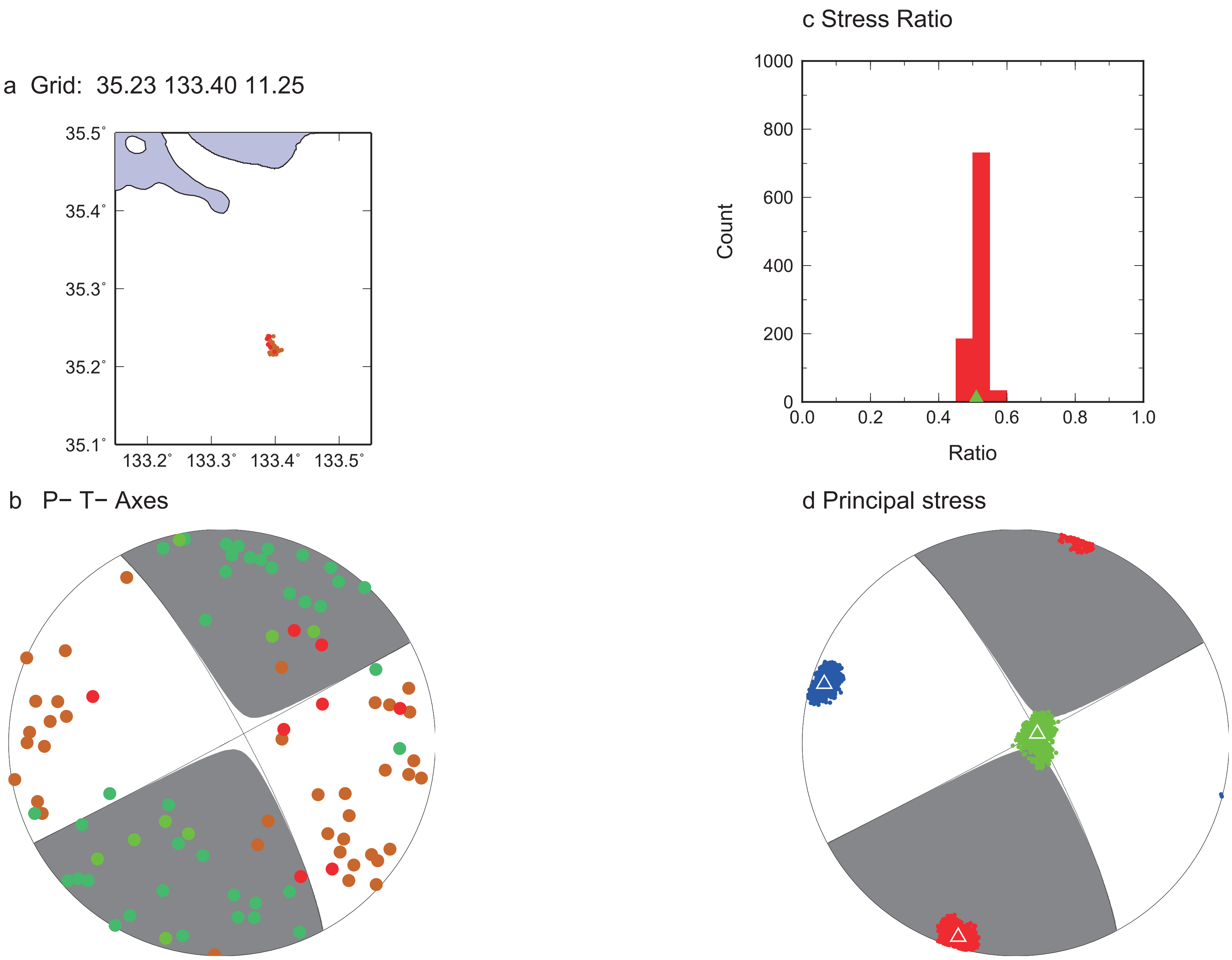

a Grid: 35.23 133.40 11.25
35.5˚
35.4˚
35.3˚
35.2˚
35.1˚
133.2˚
133.3˚
133.4˚
133.5˚
c Stress Ratio
Count
1000
800
600
400
200
0
0.0
0.2
0.4
0.6
0.8
1.0
Ratio
b P− T− Axes
d Principal stress

a Grid: 35.23 133.42 3.75

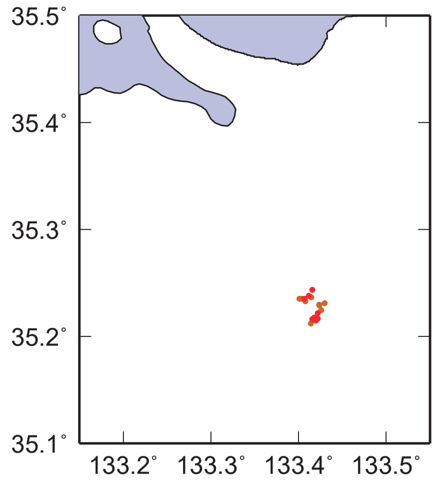

c Stress Ratio

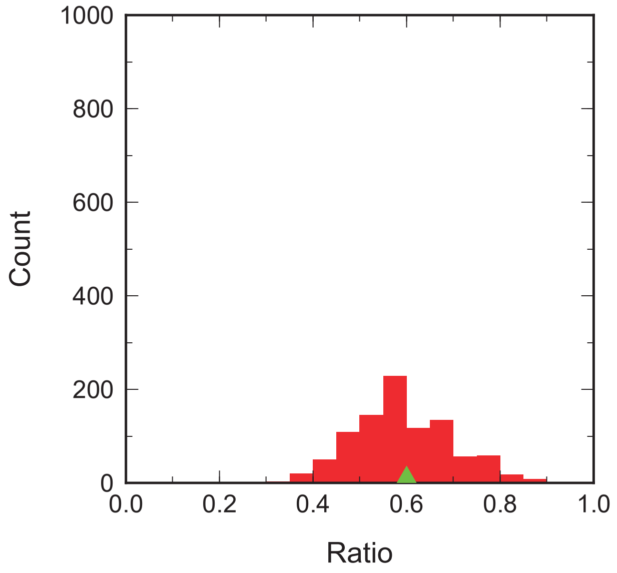

b P− T− Axes

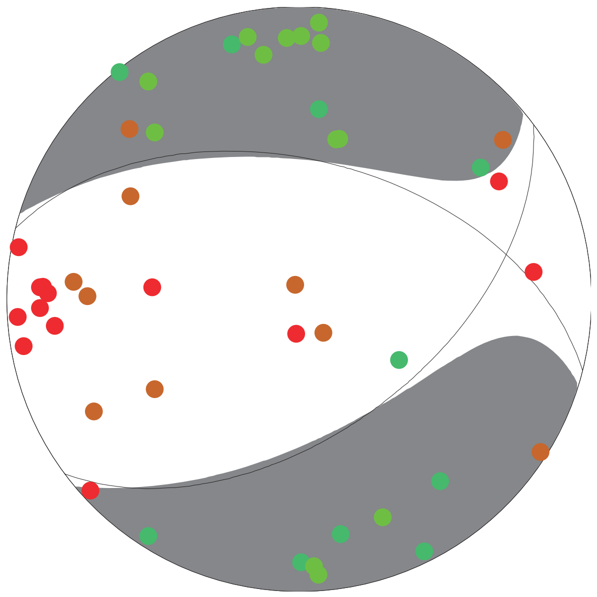

d Principal stress

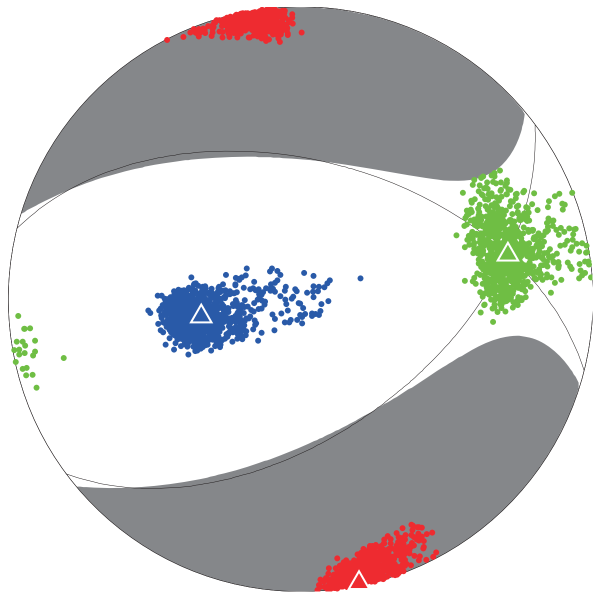

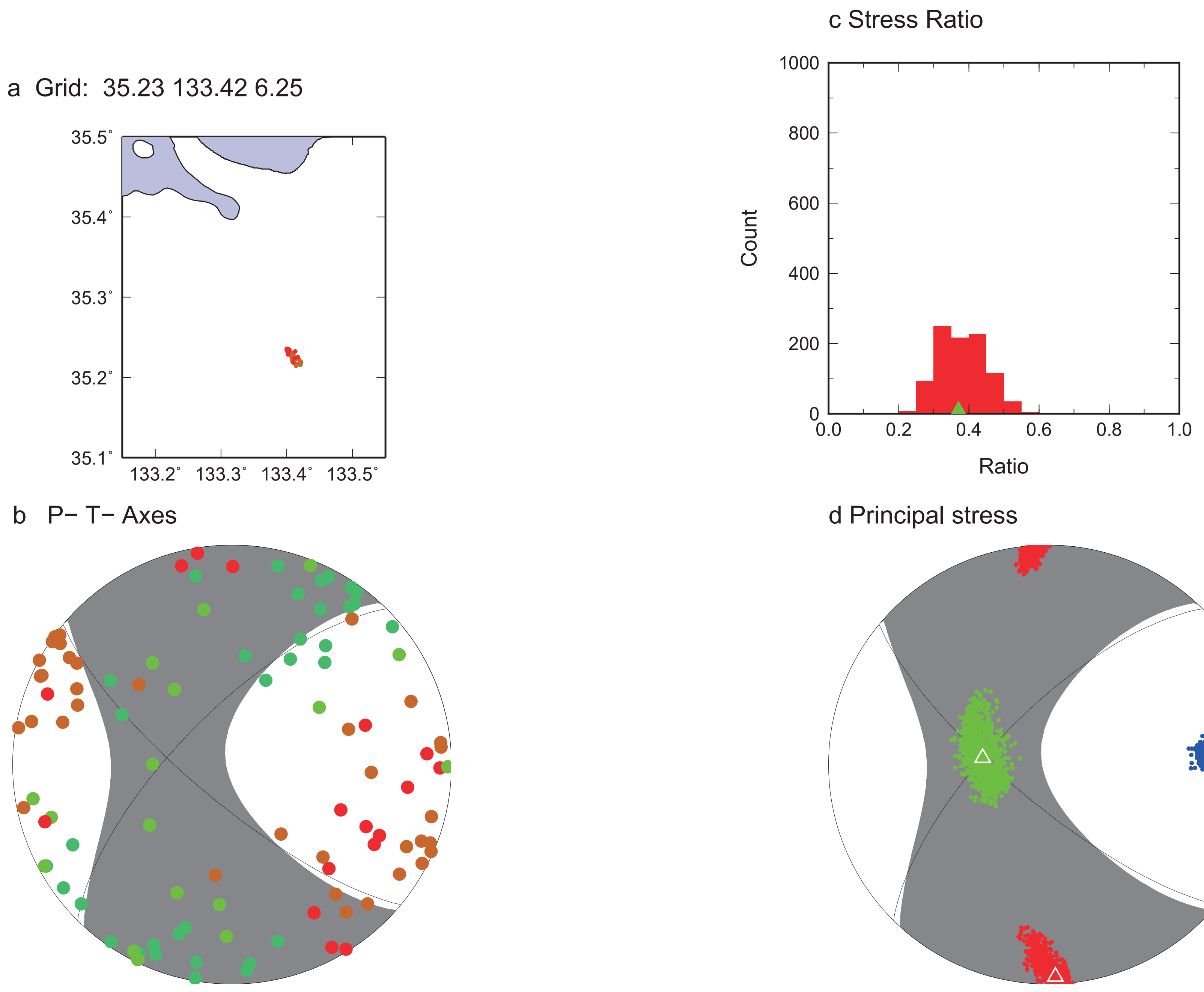
a Grid: 35.23 133.42 6.25
35.5˚
35.4˚
35.3˚
35.2˚
35.1˚
133.2˚
133.3˚
133.4˚
133.5˚
b P− T− Axes
c Stress Ratio
Count
1000
800
600
400
200
0
0.0
0.2
0.4
0.6
0.8
1.0
Ratio
d Principal stress

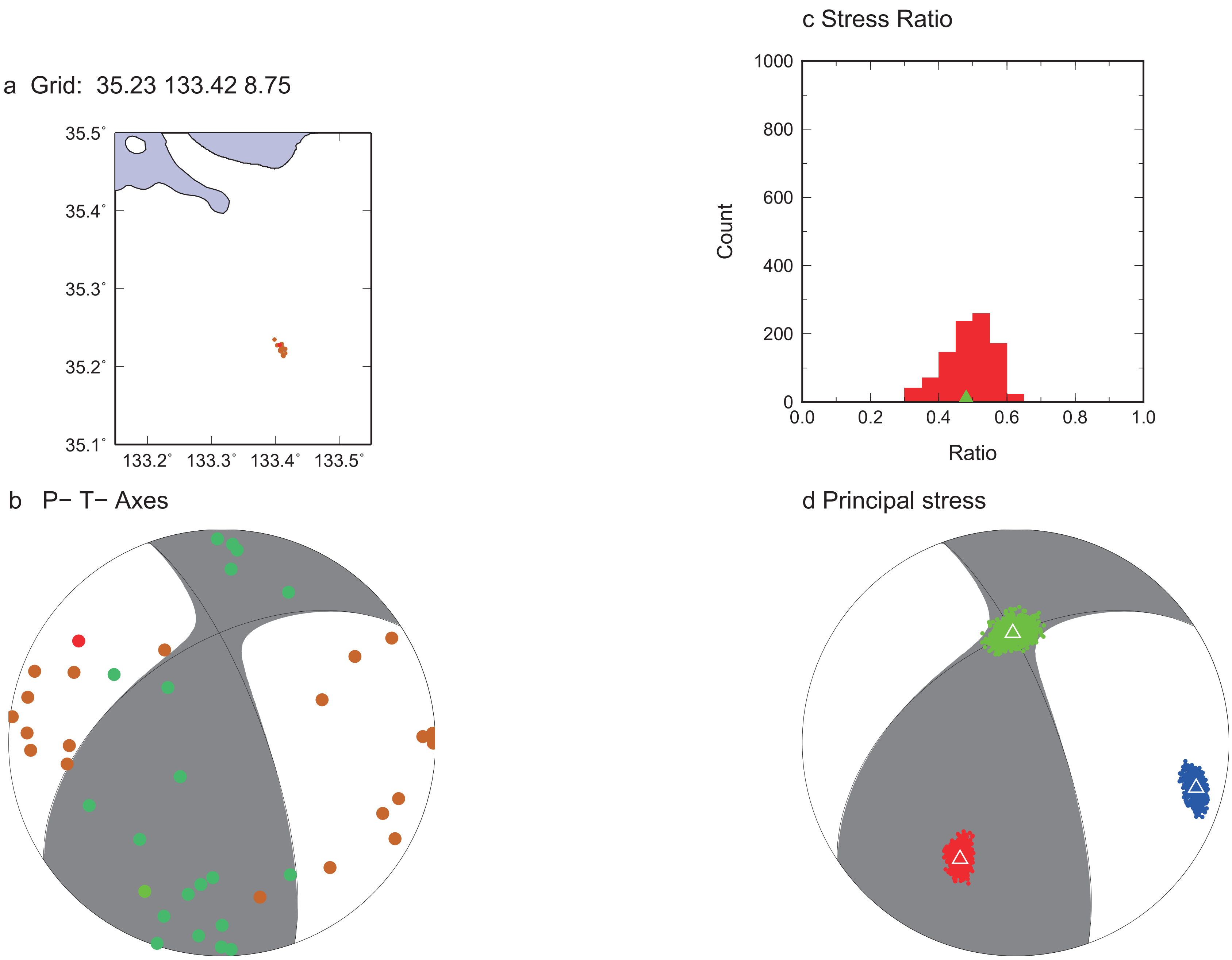
a Grid: 35.23 133.42 8.75
35.5˚
35.4˚
35.3˚
35.2˚
35.1˚
133.2˚
133.3˚
133.4˚
133.5˚
b P− T− Axes
c Stress Ratio
Count
1000
800
600
400
200
0
0.0
0.2
0.4
0.6
0.8
1.0
Ratio
d Principal stress

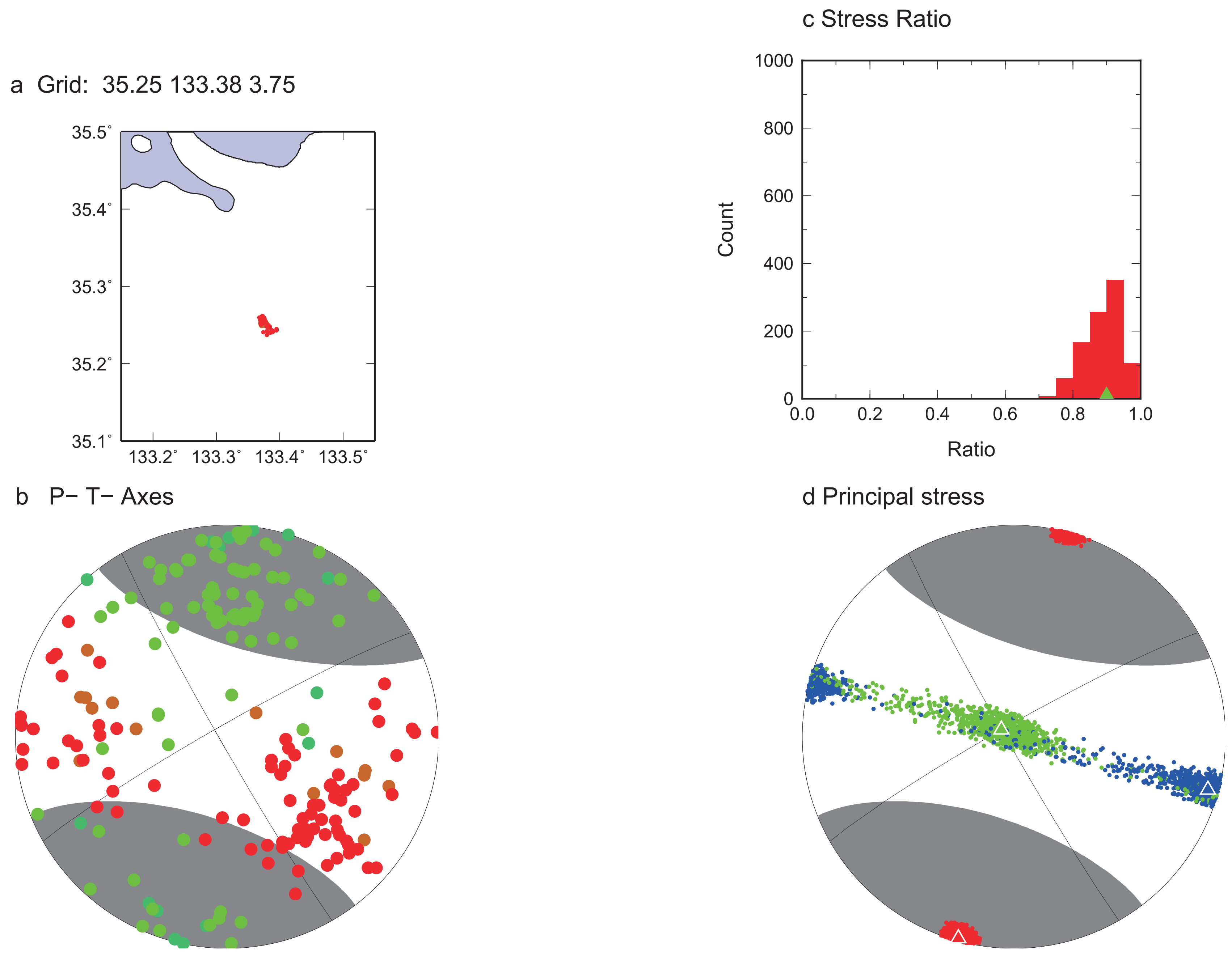

a Grid: 35.25 133.38 3.75
35.5˚
35.4˚
35.3˚
35.2˚
35.1˚
133.2˚ 133.3˚ 133.4˚ 133.5˚
b P− T− Axes
c Stress Ratio
Count
1000
800
600
400
200
0
0.0 0.2 0.4 0.6 0.8 1.0
Ratio
d Principal stress

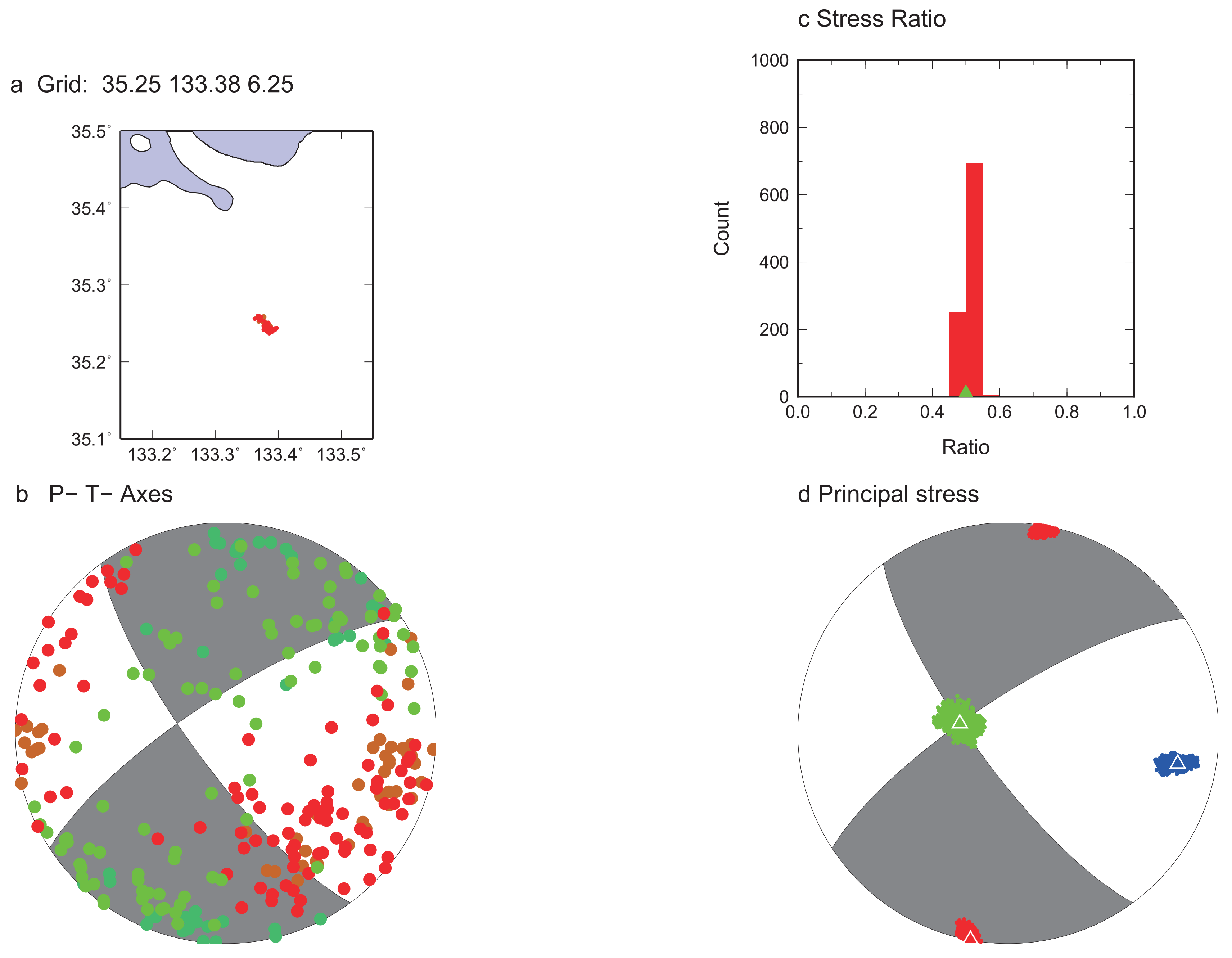
a Grid: 35.25 133.38 6.25
35.5˚
35.4˚
35.3˚
35.2˚
35.1˚
133.2˚ 133.3˚ 133.4˚ 133.5˚
b P– T– Axes
c Stress Ratio
Count
1000
800
600
400
200
0
0.0 0.2 0.4 0.6 0.8 1.0
Ratio
d Principal stress

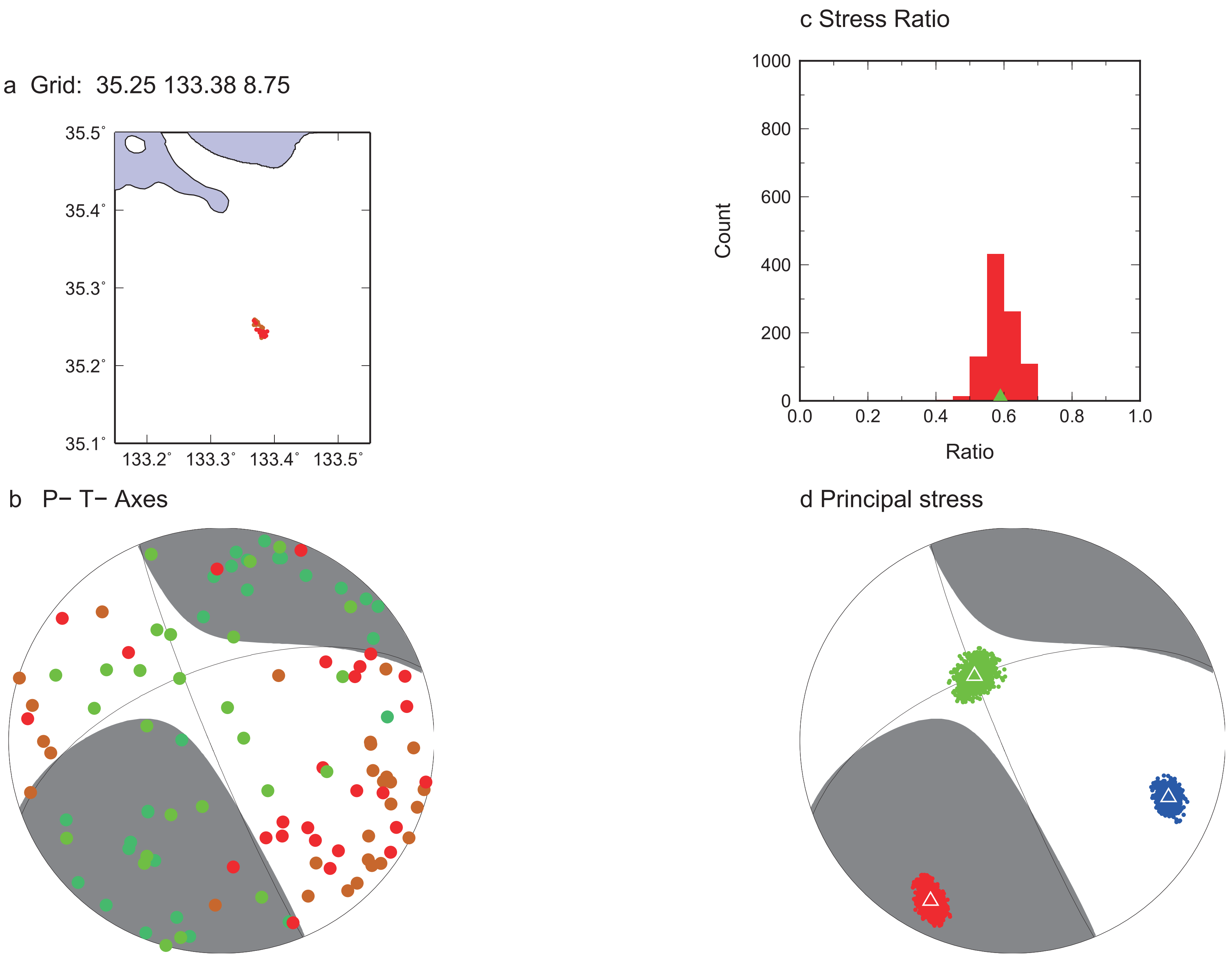
a Grid: 35.25 133.38 8.75
35.5˚
35.4˚
35.3˚
35.2˚
35.1˚
133.2˚ 133.3˚ 133.4˚ 133.5˚
b P− T− Axes
c Stress Ratio
Count
1000
800
600
400
200
0
0.0 0.2 0.4 0.6 0.8 1.0
Ratio
d Principal stress

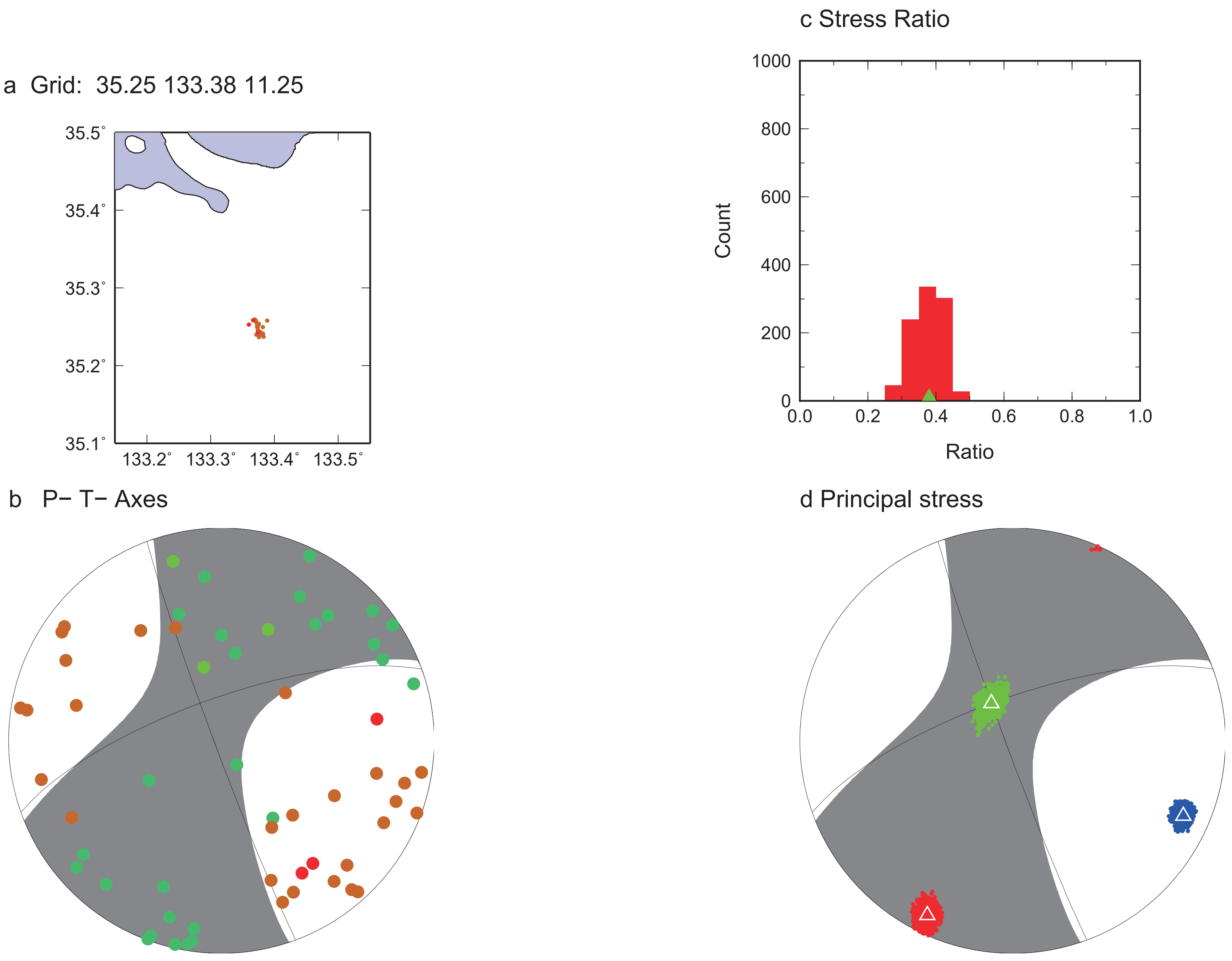
a Grid: 35.25 133.38 11.25
35.5˚
35.4˚
35.3˚
35.2˚
35.1˚
133.2˚ 133.3˚ 133.4˚ 133.5˚
b P– T– Axes
c Stress Ratio
Count
1000
800
600
400
200
0
0.0 0.2 0.4 0.6 0.8 1.0
Ratio
d Principal stress

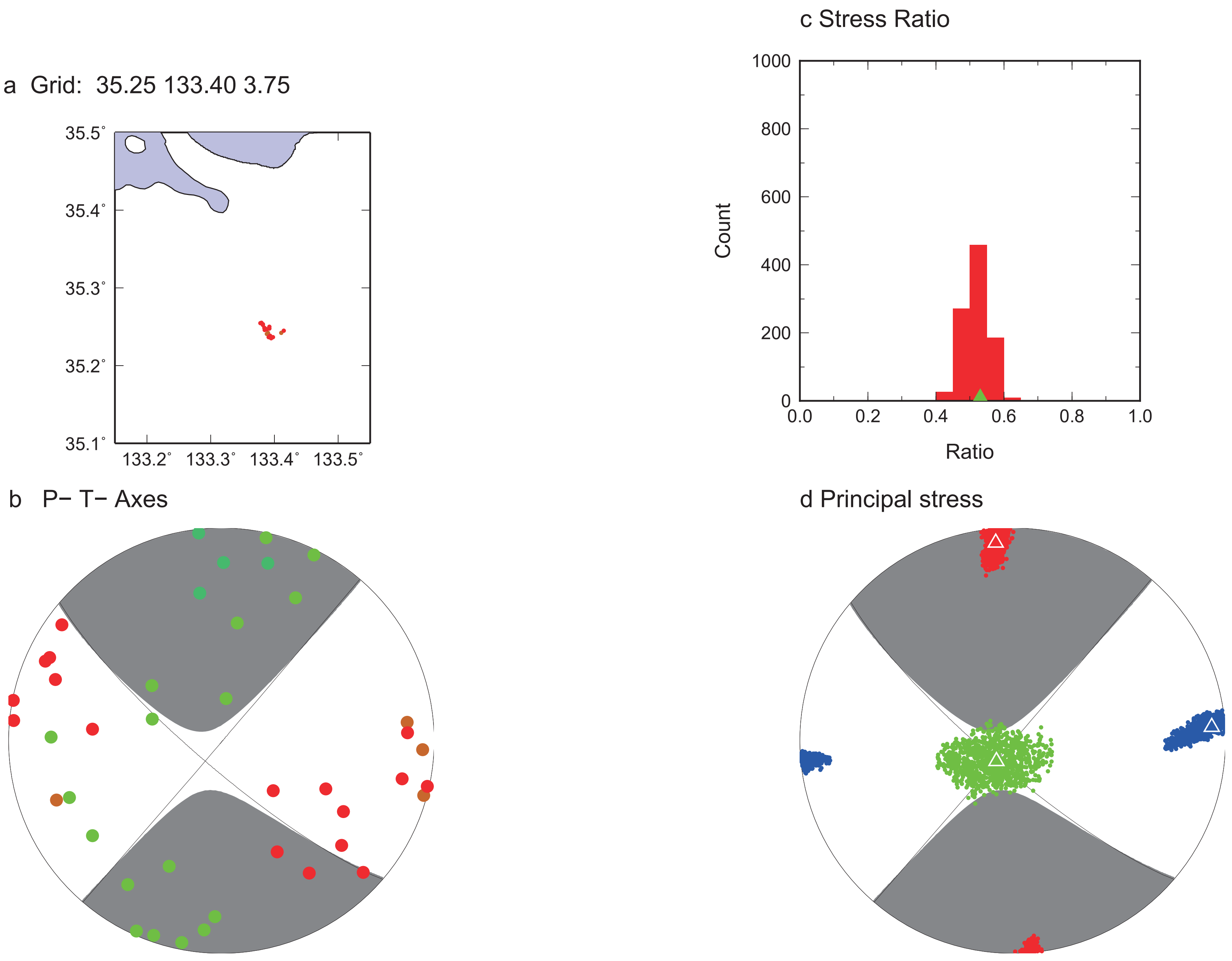
a Grid: 35.25 133.40 3.75
35.5˚
35.4˚
35.3˚
35.2˚
35.1˚
133.2˚
133.3˚
133.4˚
133.5˚
b P− T− Axes
c Stress Ratio
1000
800
600
400
200
0
Count
0.0
0.2
0.4
0.6
0.8
1.0
Ratio
d Principal stress

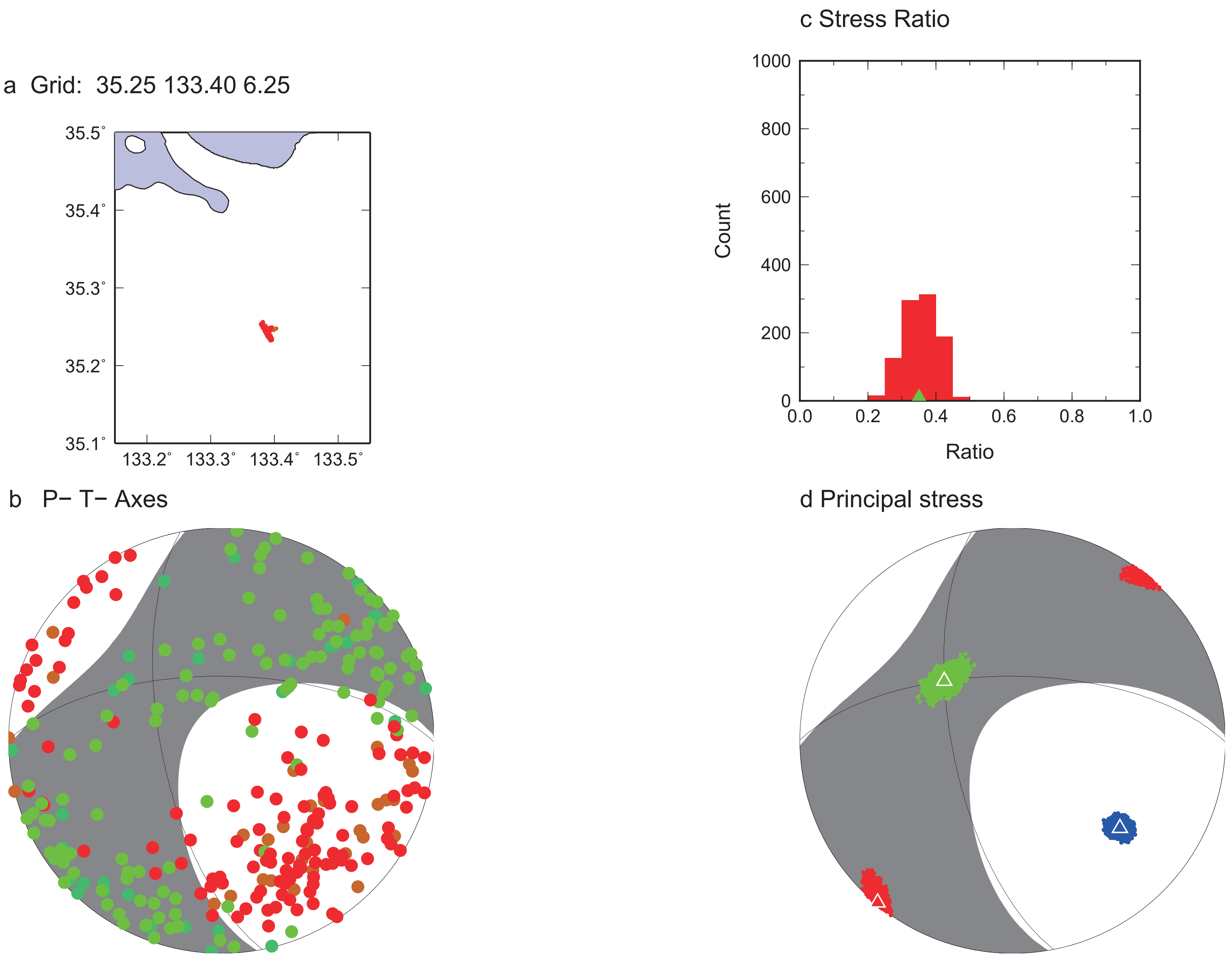
a Grid: 35.25 133.40 6.25
35.5˚
35.4˚
35.3˚
35.2˚
35.1˚
133.2˚ 133.3˚ 133.4˚ 133.5˚
b P− T− Axes
c Stress Ratio
Count
1000
800
600
400
200
0
0.0 0.2 0.4 0.6 0.8 1.0
Ratio
d Principal stress

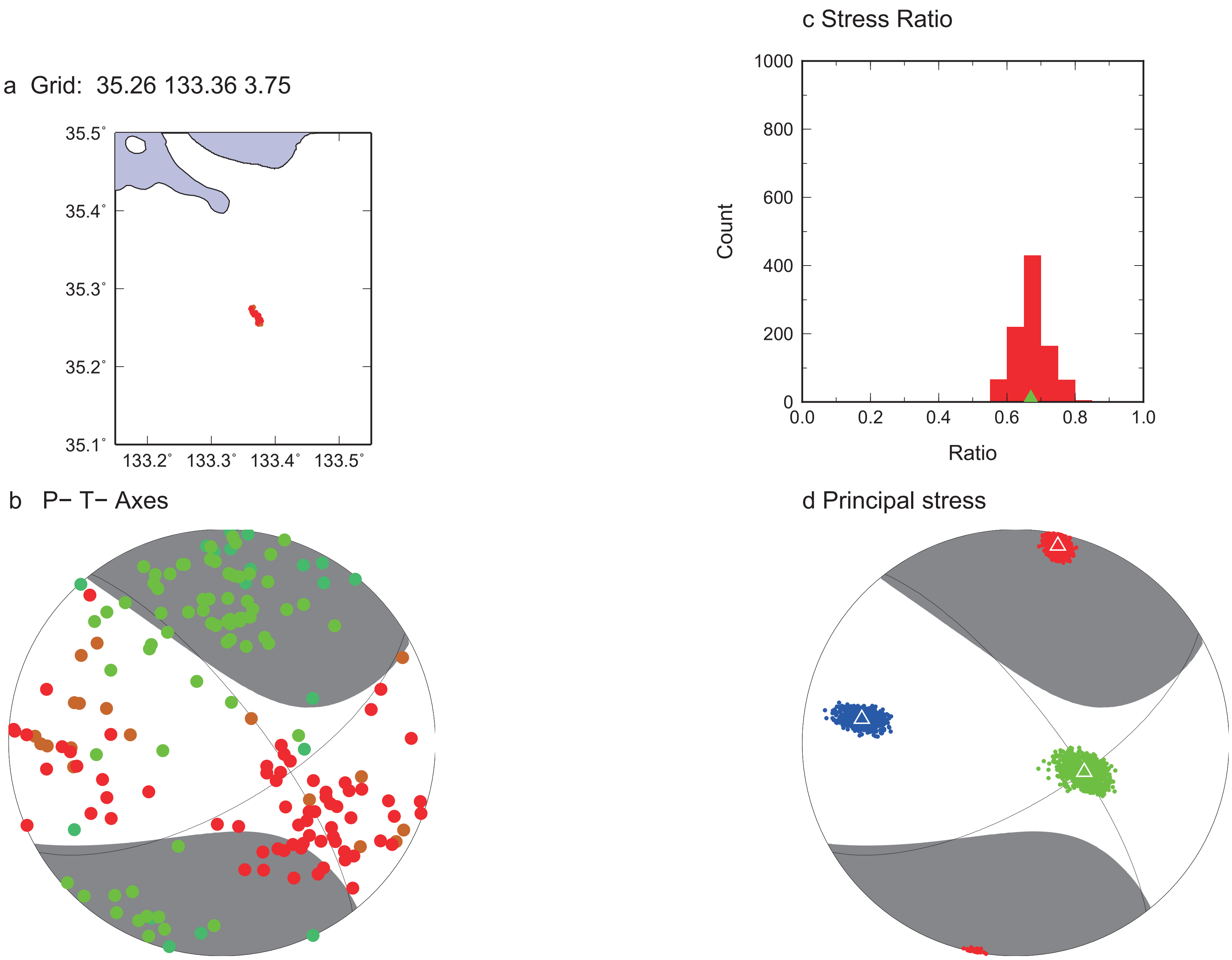

a Grid: 35.26 133.36 3.75
35.5°
35.4°
35.3°
35.2°
35.1°
133.2°
133.3°
133.4°
133.5°
b P– T– Axes
c Stress Ratio
1000
800
600
400
200
0
Count
0.0
0.2
0.4
0.6
0.8
1.0
Ratio
d Principal stress

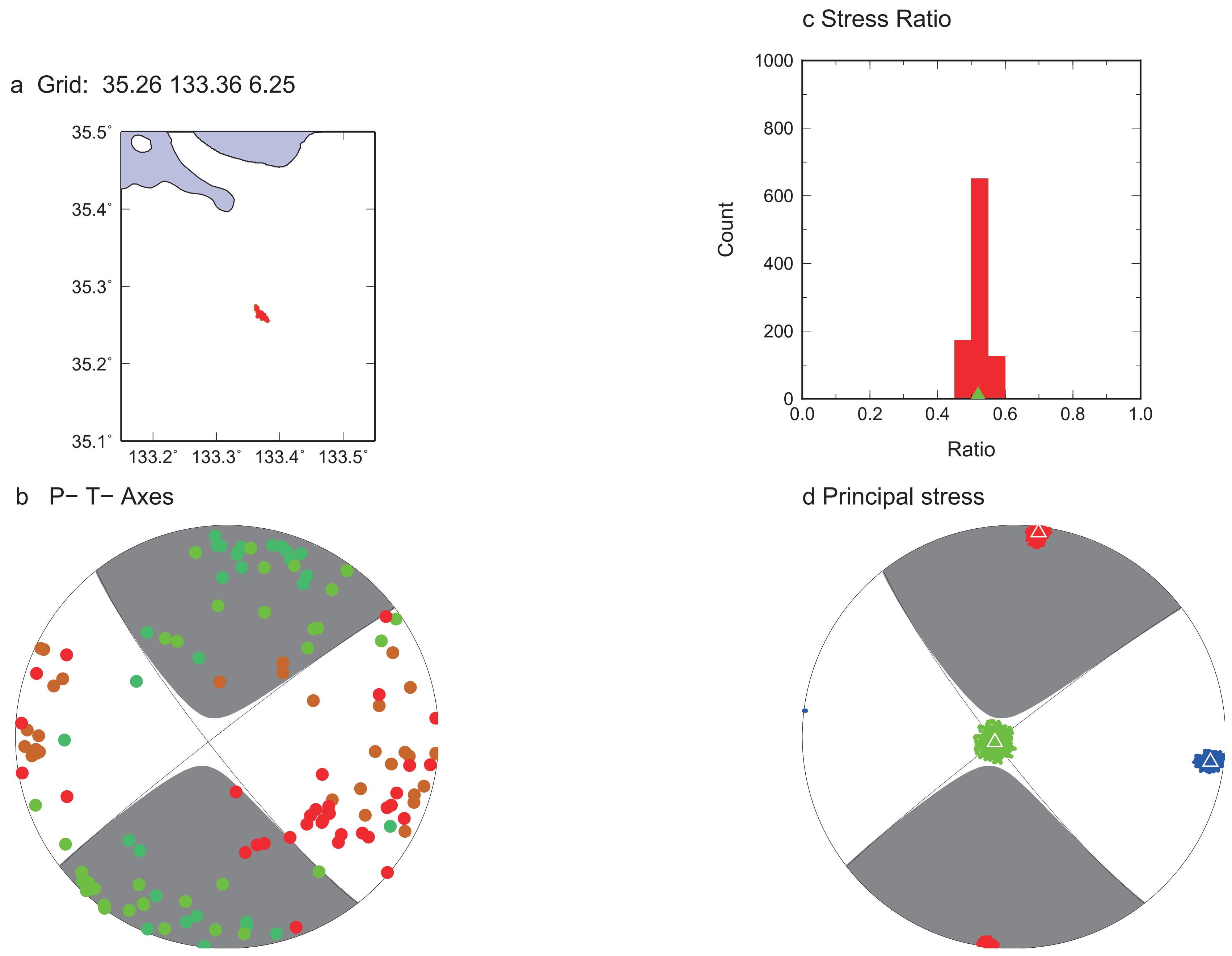
a Grid: 35.26 133.36 6.25
35.5˚
35.4˚
35.3˚
35.2˚
35.1˚
133.2˚
133.3˚
133.4˚
133.5˚
b P− T− Axes
c Stress Ratio
Count
1000
800
600
400
200
0
0.0
0.2
0.4
0.6
0.8
1.0
Ratio
d Principal stress

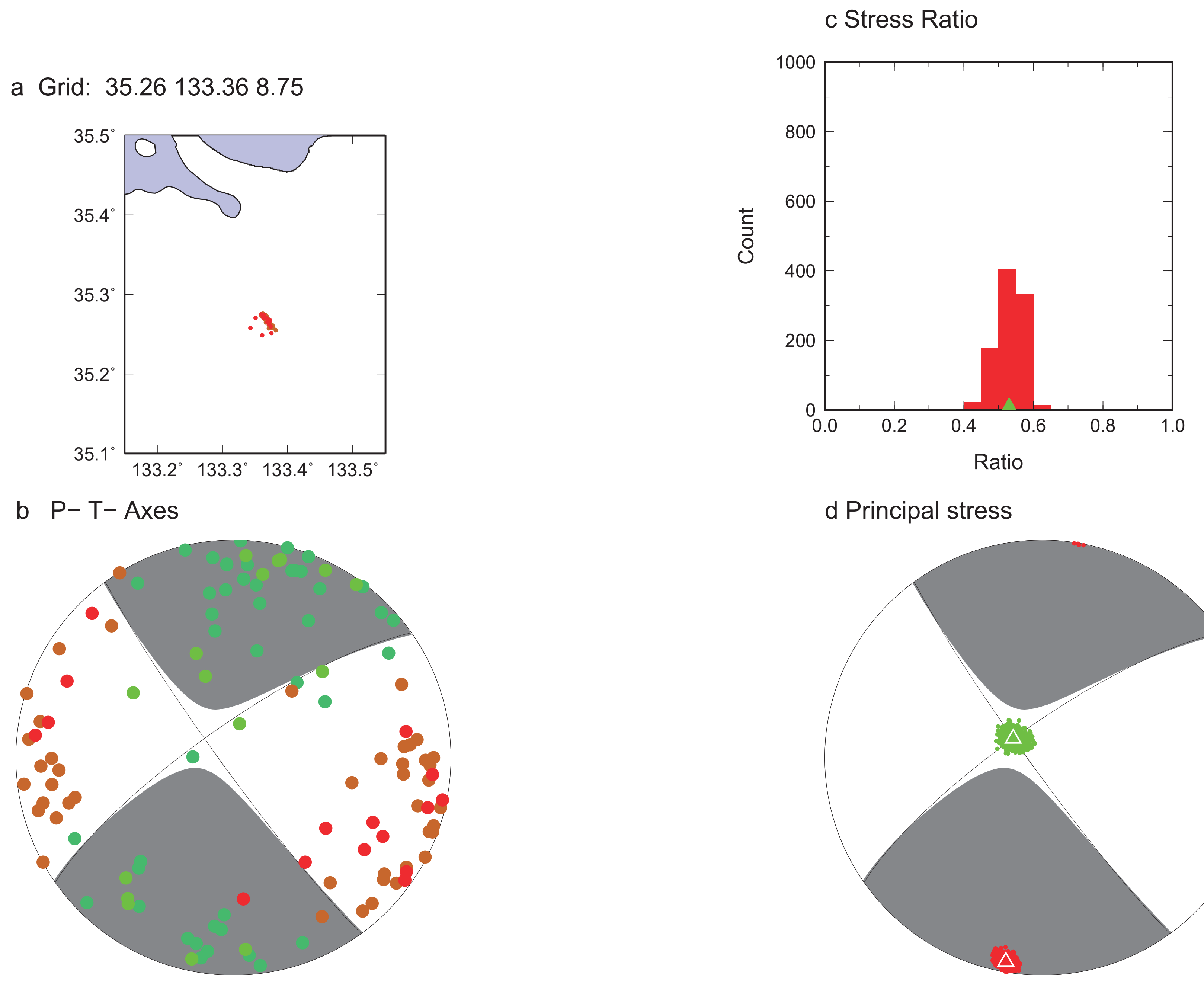
a Grid: 35.26 133.36 8.75
35.5˚
35.4˚
35.3˚
35.2˚
35.1˚
133.2˚ 133.3˚ 133.4˚ 133.5˚
c Stress Ratio
Count
1000
800
600
400
200
0
0.0 0.2 0.4 0.6 0.8 1.0
Ratio
b P− T− Axes
d Principal stress

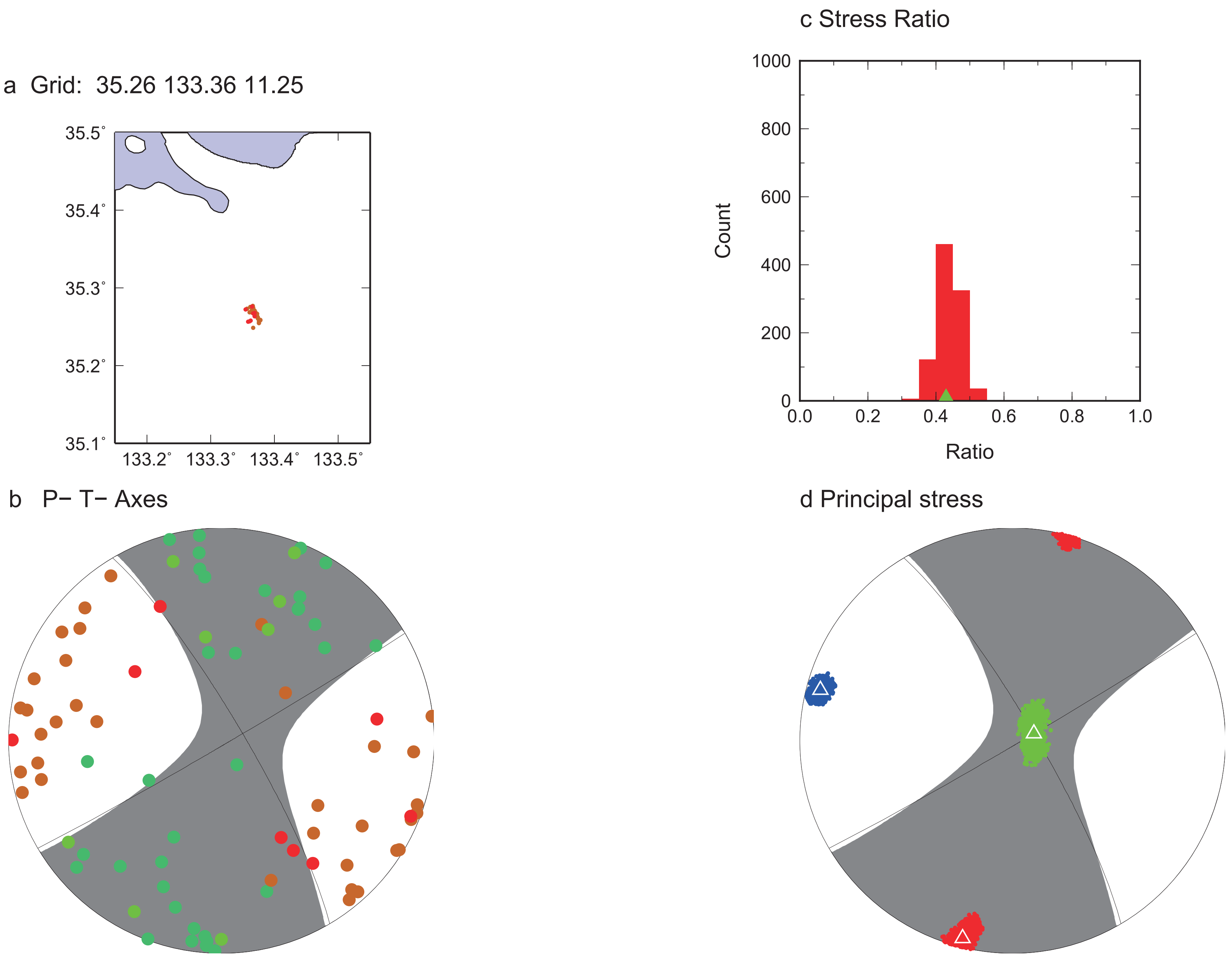
a Grid: 35.26 133.36 11.25
35.5˚
35.4˚
35.3˚
35.2˚
35.1˚
133.2˚
133.3˚
133.4˚
133.5˚
b P– T– Axes
c Stress Ratio
1000
800
600
400
200
0
Count
0.0
0.2
0.4
0.6
0.8
1.0
Ratio
d Principal stress

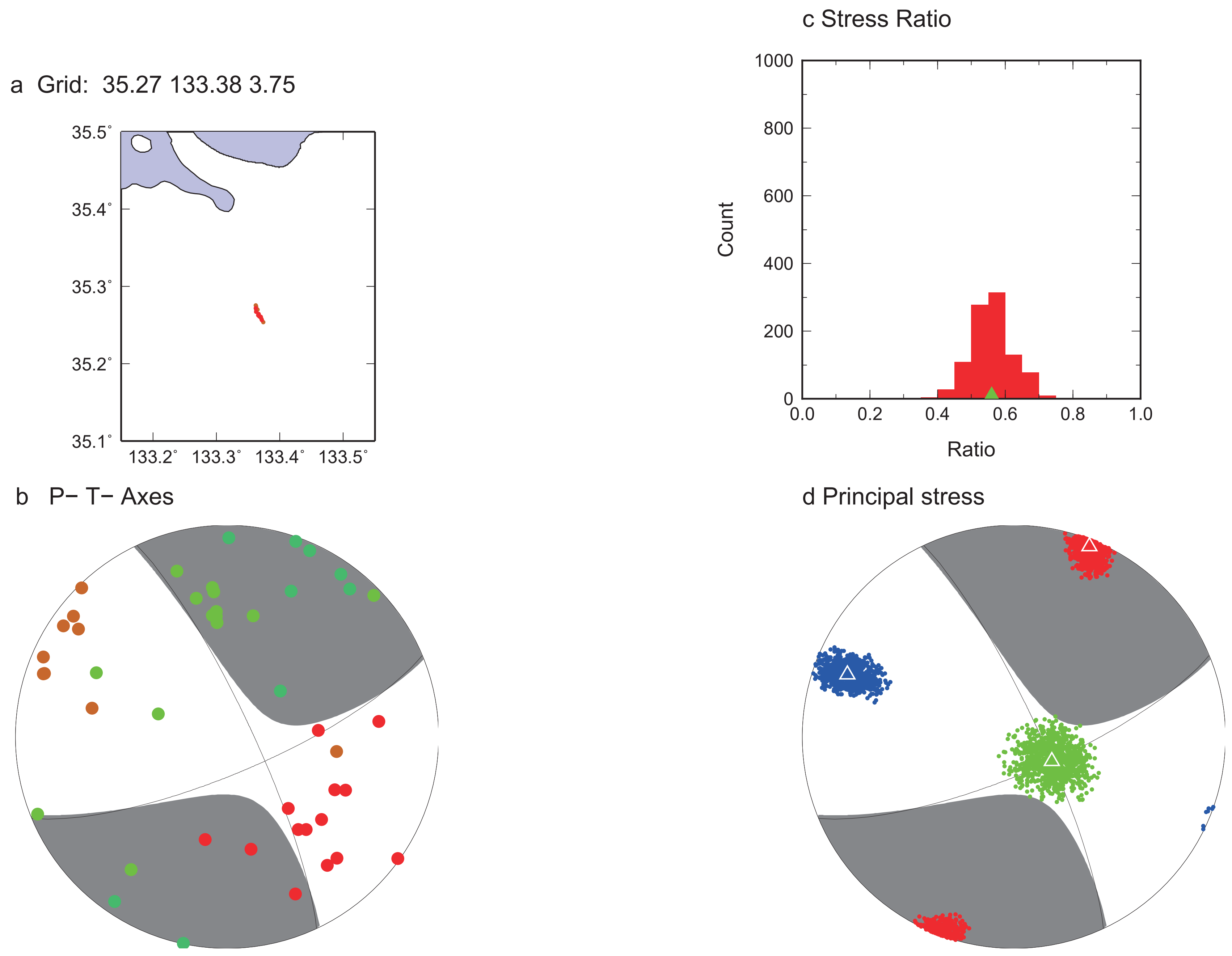
a Grid: 35.27 133.38 3.75
35.5˚
35.4˚
35.3˚
35.2˚
35.1˚
133.2˚ 133.3˚ 133.4˚ 133.5˚
c Stress Ratio
Count
1000
800
600
400
200
0
0.0 0.2 0.4 0.6 0.8 1.0
Ratio
b P− T− Axes
d Principal stress

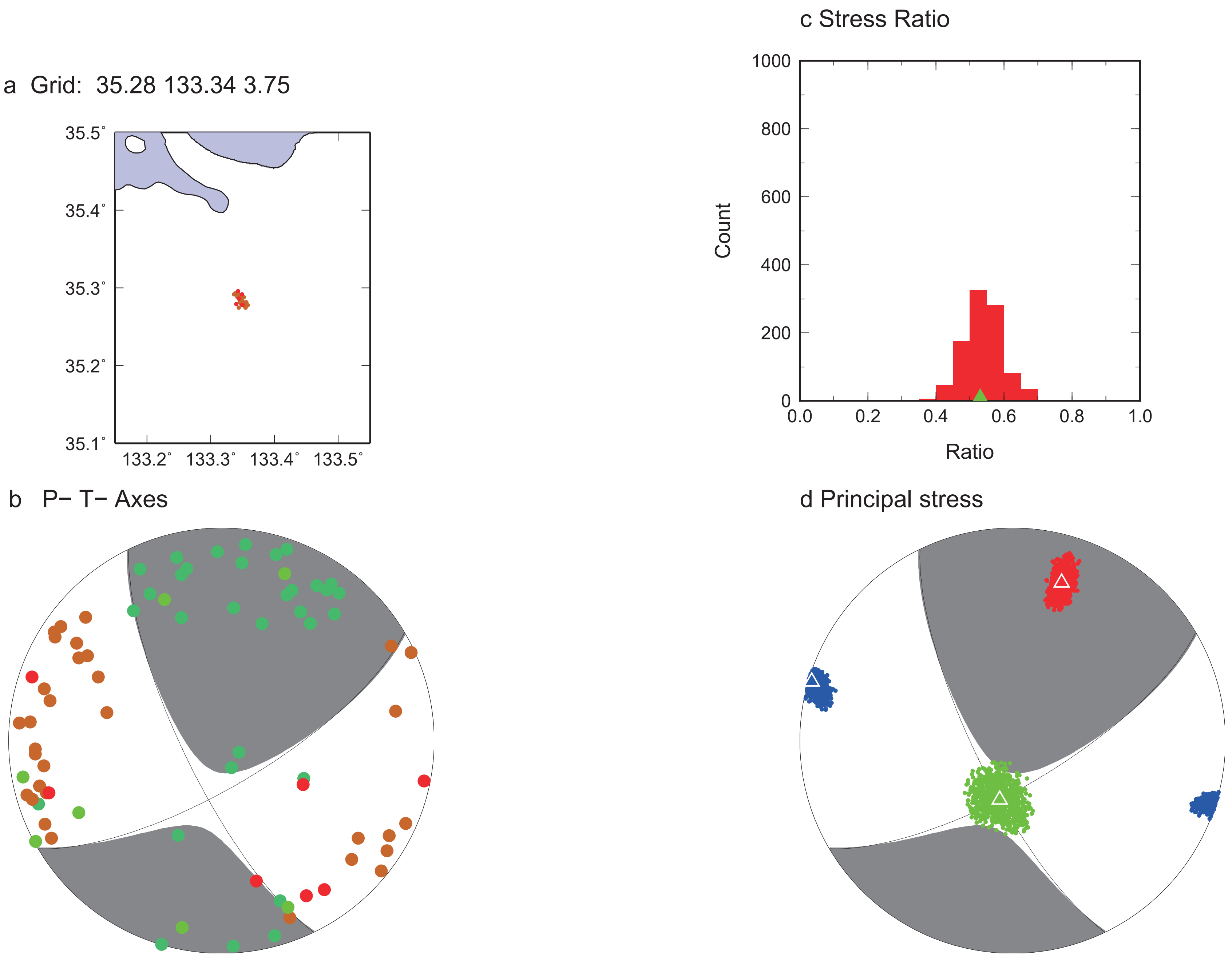
a Grid: 35.28 133.34 3.75
35.5˚
35.4˚
35.3˚
35.2˚
35.1˚
133.2˚ 133.3˚ 133.4˚ 133.5˚
c Stress Ratio
1000
800
600
400
200
0
Count
0.0 0.2 0.4 0.6 0.8 1.0
Ratio
b P− T− Axes
d Principal stress

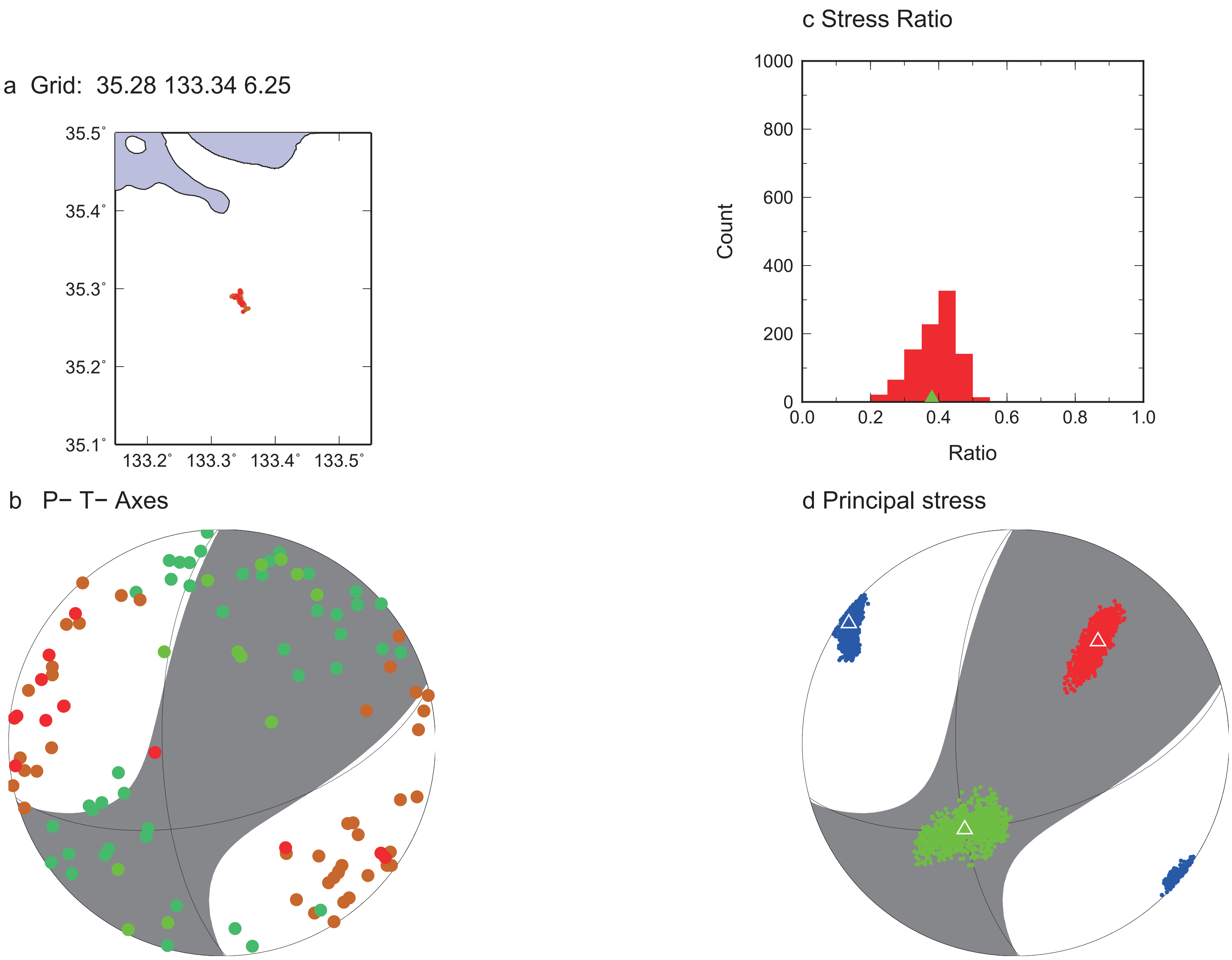
a Grid: 35.28 133.34 6.25
35.5˚
35.4˚
35.3˚
35.2˚
35.1˚
133.2˚
133.3˚
133.4˚
133.5˚
b P− T− Axes
c Stress Ratio
Count
1000
800
600
400
200
0
0.0
0.2
0.4
0.6
0.8
1.0
Ratio
d Principal stress

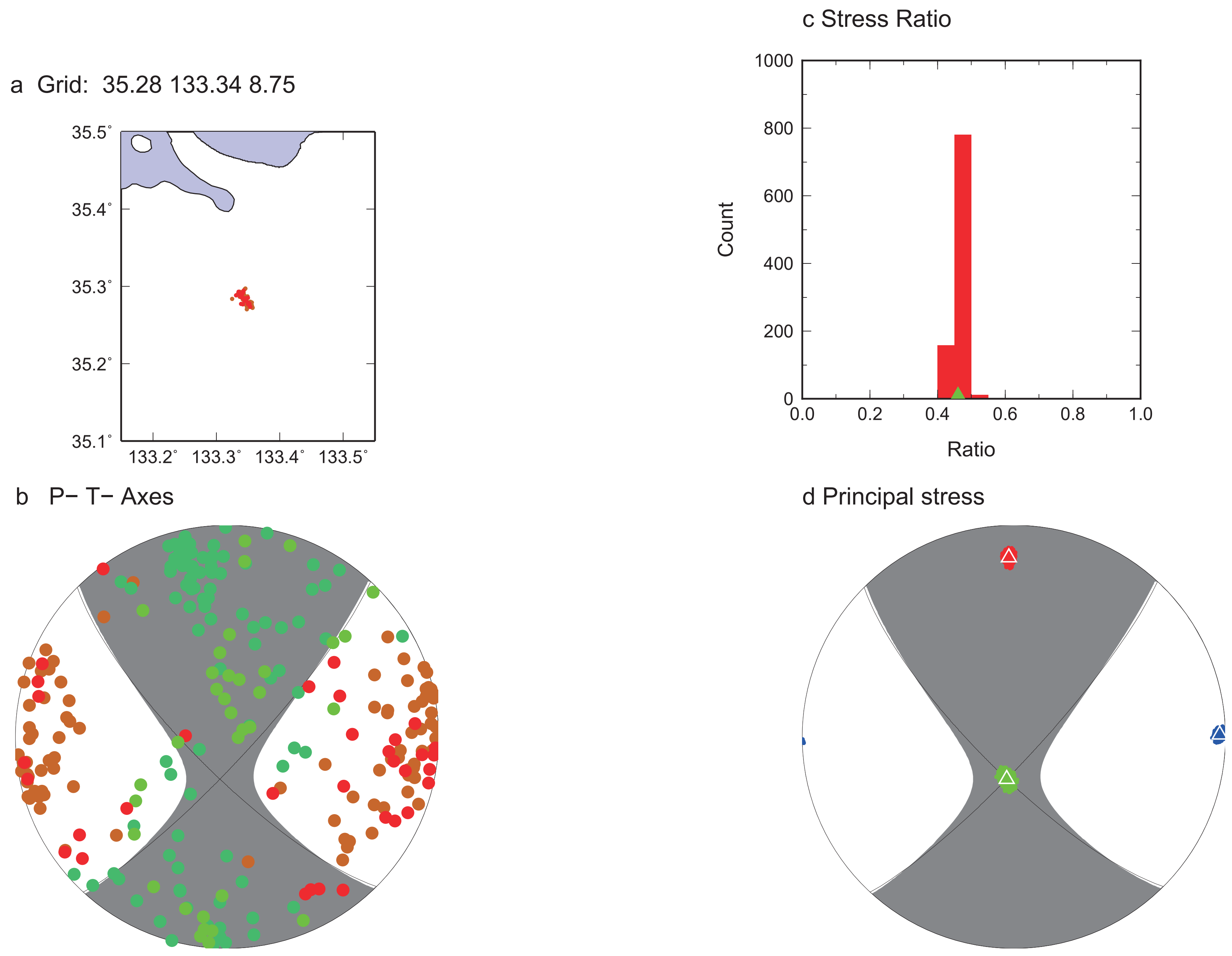
a Grid: 35.28 133.34 8.75
35.5˚
35.4˚
35.3˚
35.2˚
35.1˚
133.2˚ 133.3˚ 133.4˚ 133.5˚
b P− T− Axes
c Stress Ratio
Count
1000
800
600
400
200
0
0.0 0.2 0.4 0.6 0.8 1.0
Ratio
d Principal stress

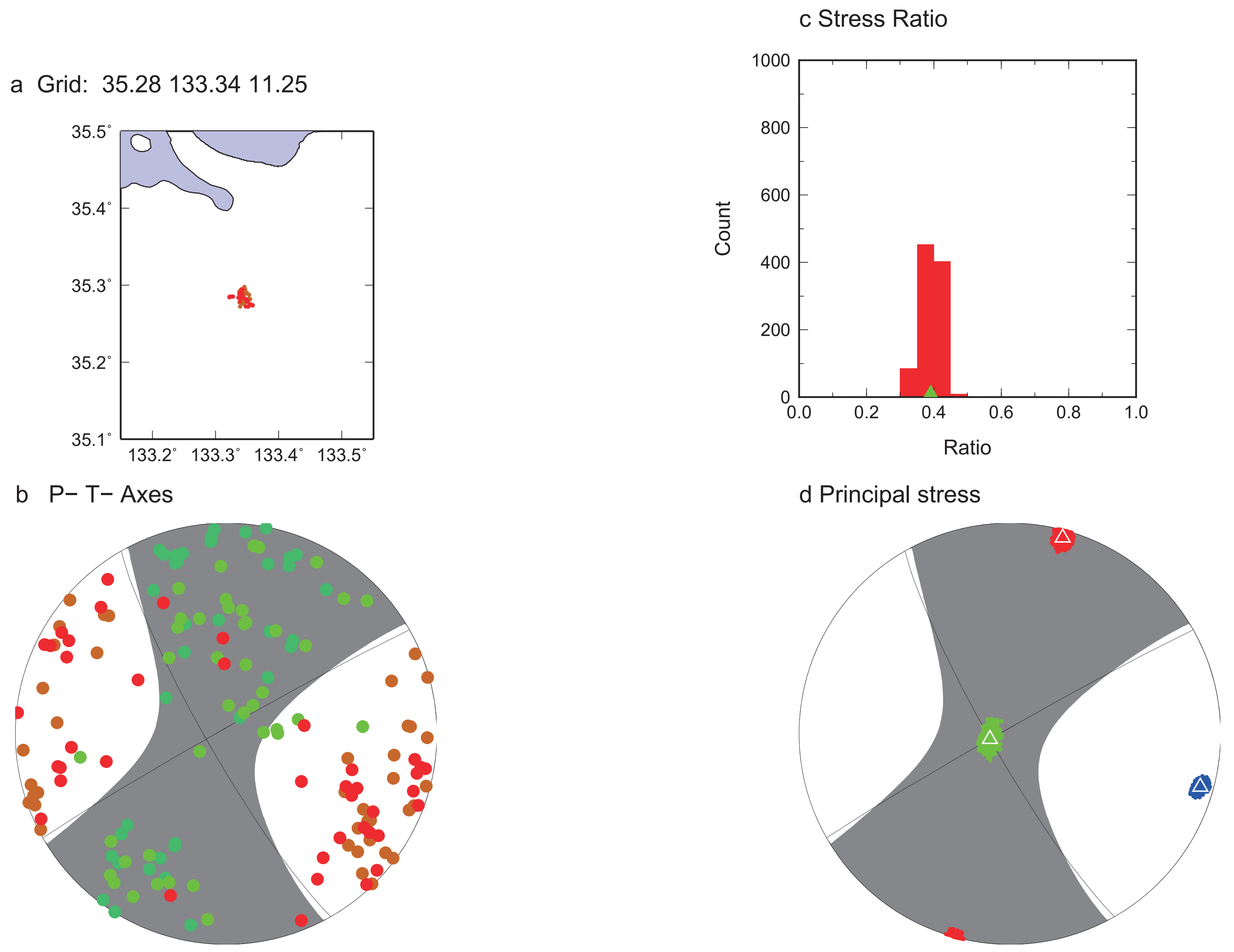

a Grid: 35.28 133.34 11.25
35.5˚
35.4˚
35.3˚
35.2˚
35.1˚
133.2˚
133.3˚
133.4˚
133.5˚
b P– T– Axes
c Stress Ratio
1000
800
600
400
200
0
Count
0.0
0.2
0.4
0.6
0.8
1.0
Ratio
d Principal stress

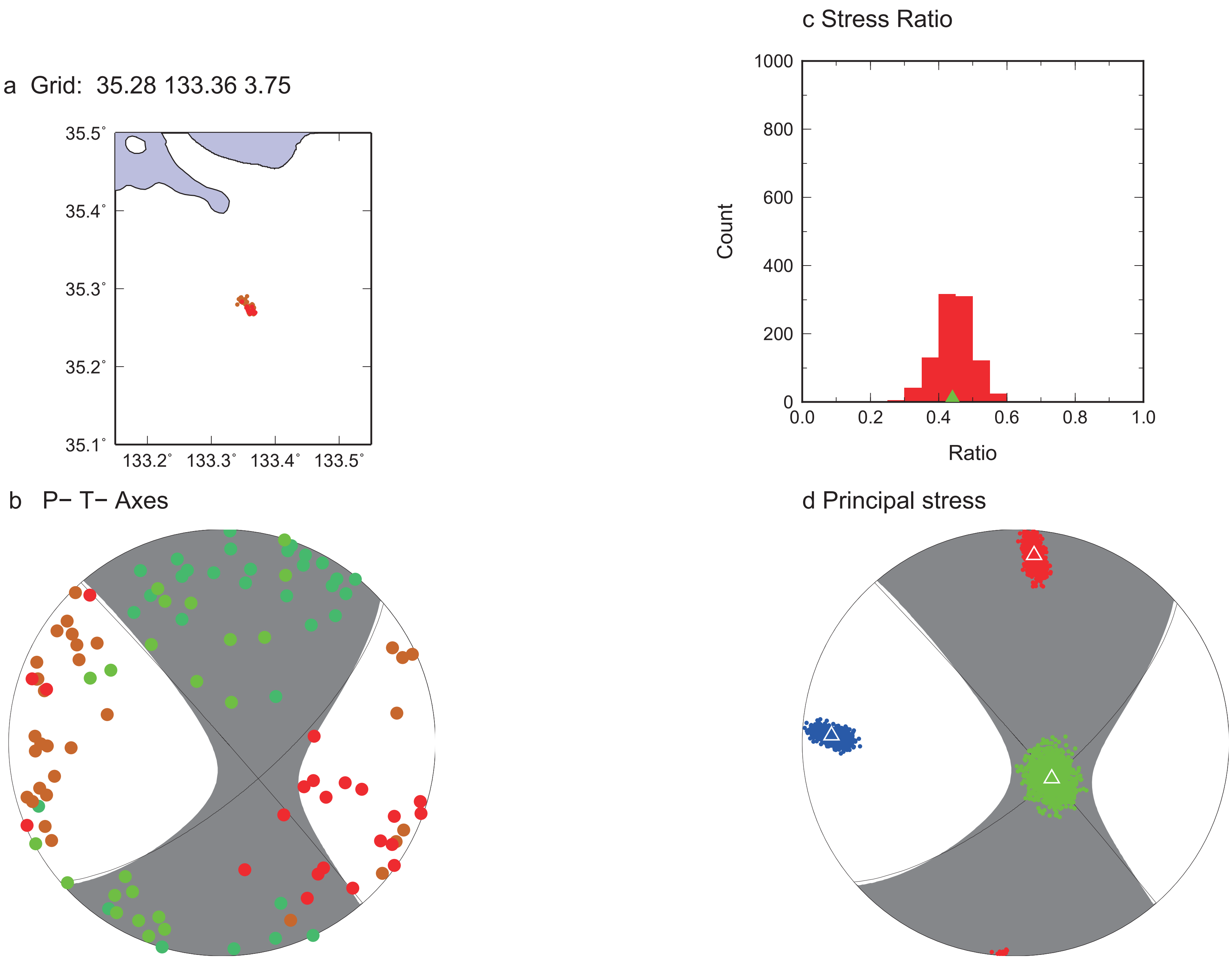

a Grid: 35.28 133.36 3.75
35.5˚
35.4˚
35.3˚
35.2˚
35.1˚
133.2˚
133.3˚
133.4˚
133.5˚
b P− T− Axes
c Stress Ratio
Count
1000
800
600
400
200
0
0.0
0.2
0.4
0.6
0.8
1.0
Ratio
d Principal stress

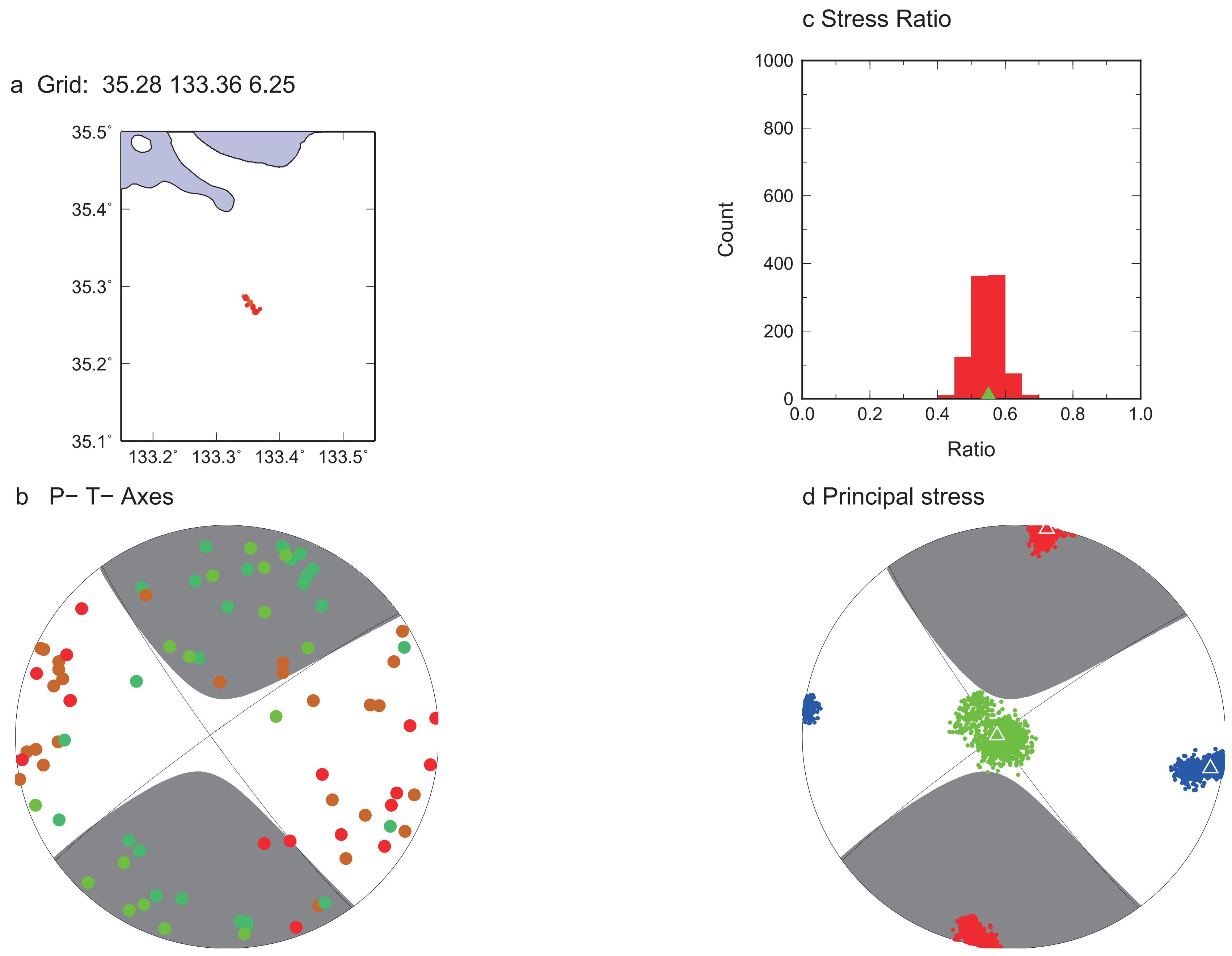
a Grid: 35.28 133.36 6.25
35.5˚
35.4˚
35.3˚
35.2˚
35.1˚
133.2˚ 133.3˚ 133.4˚ 133.5˚
c Stress Ratio
1000
800
600
400
200
0
Count
0.0 0.2 0.4 0.6 0.8 1.0
Ratio
b P− T− Axes
d Principal stress

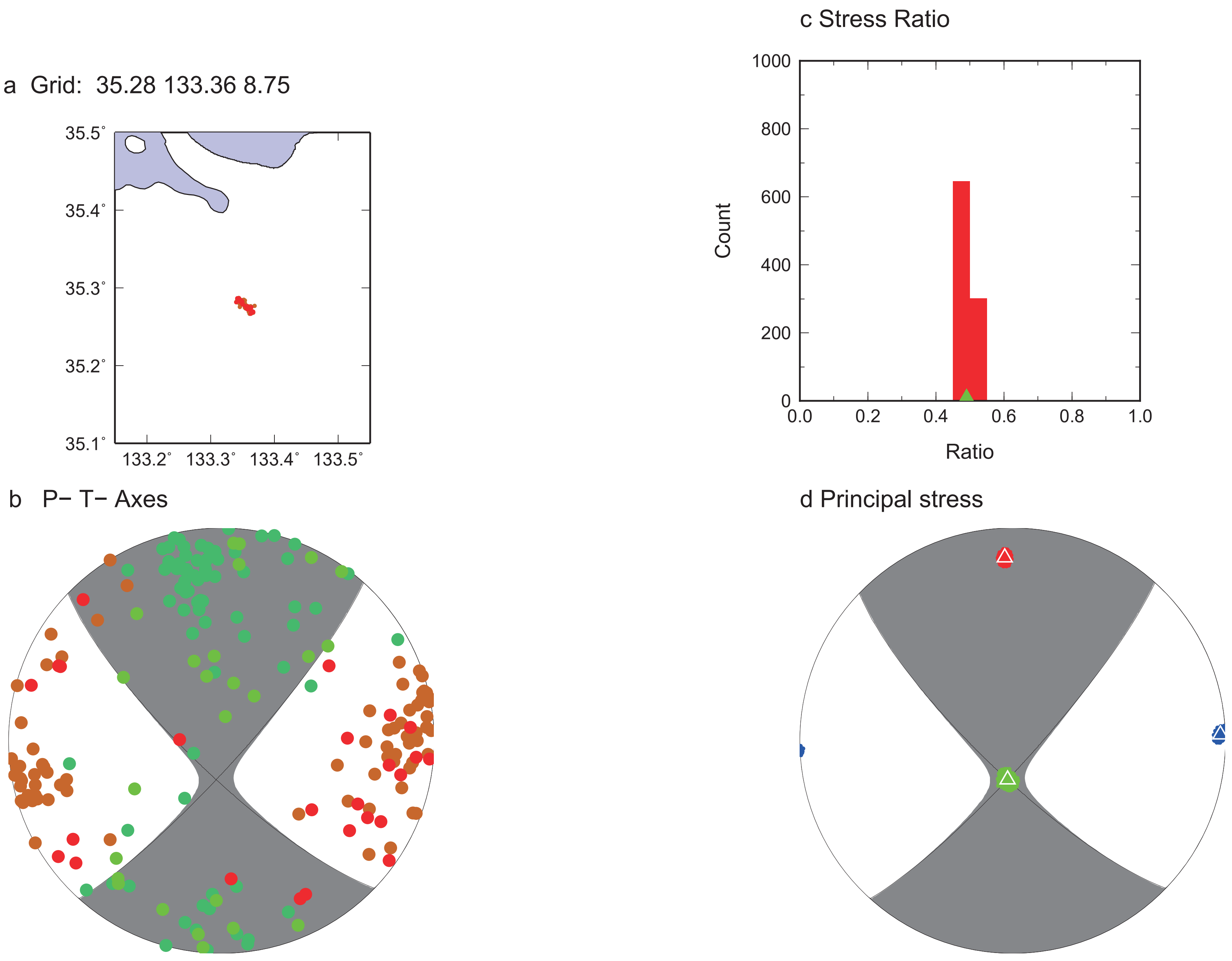
a Grid: 35.28 133.36 8.75
35.5˚
35.4˚
35.3˚
35.2˚
35.1˚
133.2˚
133.3˚
133.4˚
133.5˚
b P− T− Axes
c Stress Ratio
Count
1000
800
600
400
200
0
0.0
0.2
0.4
0.6
0.8
1.0
Ratio
d Principal stress

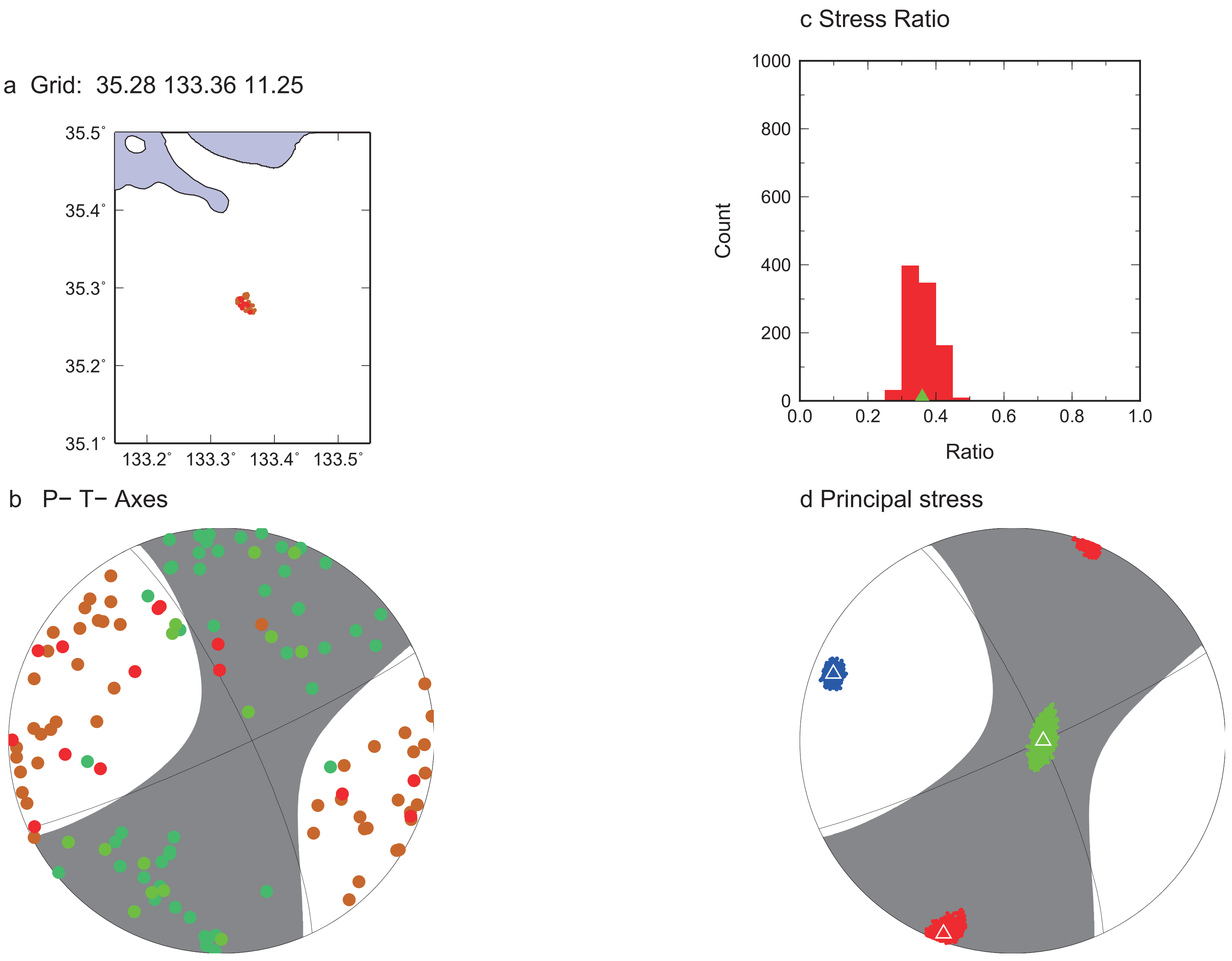
a Grid: 35.28 133.36 11.25
35.5˚
35.4˚
35.3˚
35.2˚
35.1˚
133.2˚ 133.3˚ 133.4˚ 133.5˚
c Stress Ratio
1000
800
600
400
200
0
Count
0.0 0.2 0.4 0.6 0.8 1.0
Ratio
b P– T– Axes
d Principal stress

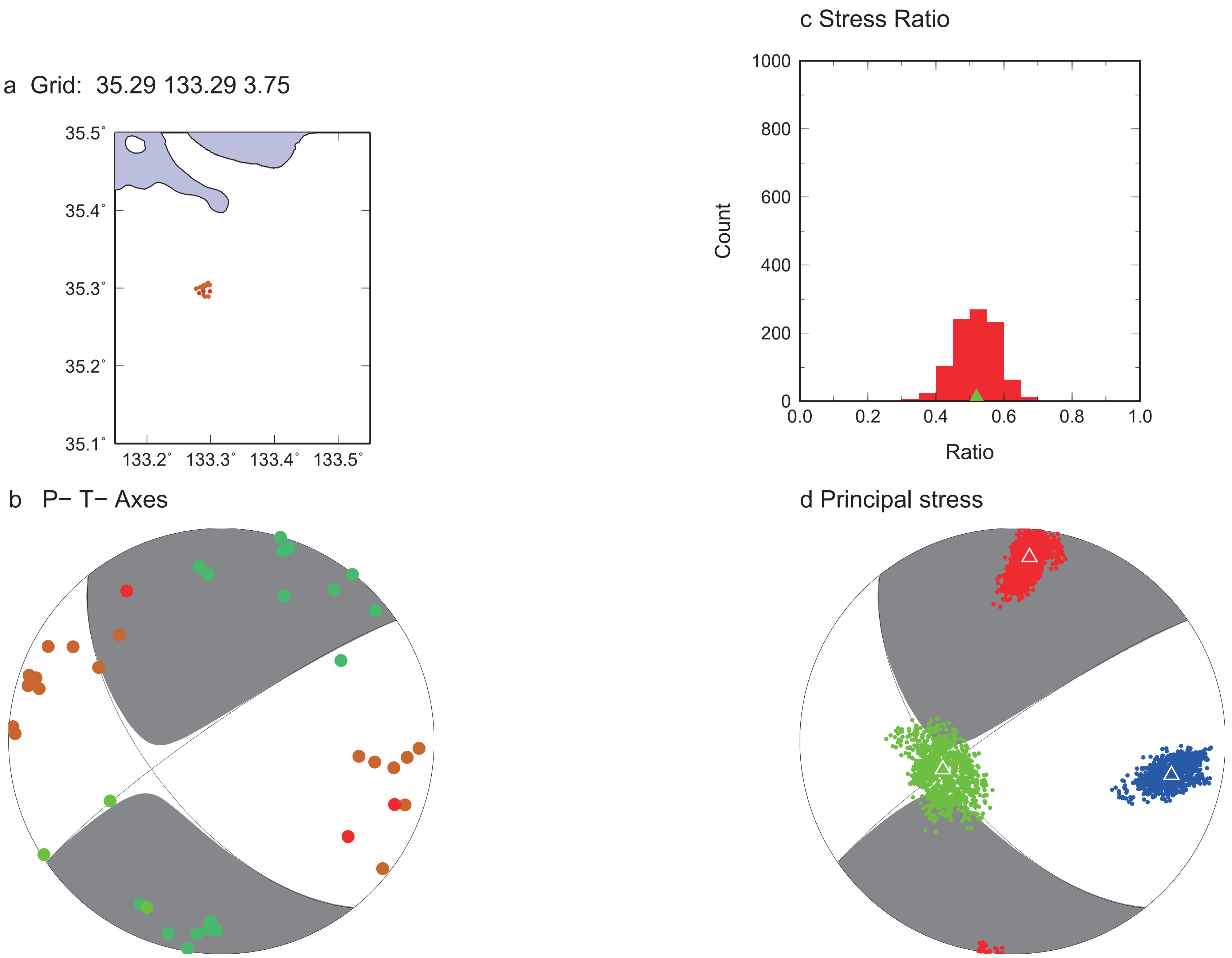

a Grid: 35.29 133.29 3.75
35.5˚
35.4˚
35.3˚
35.2˚
35.1˚
133.2˚
133.3˚
133.4˚
133.5˚
c Stress Ratio
Count
1000
800
600
400
200
0
0.0
0.2
0.4
0.6
0.8
1.0
Ratio
b P– T– Axes
d Principal stress

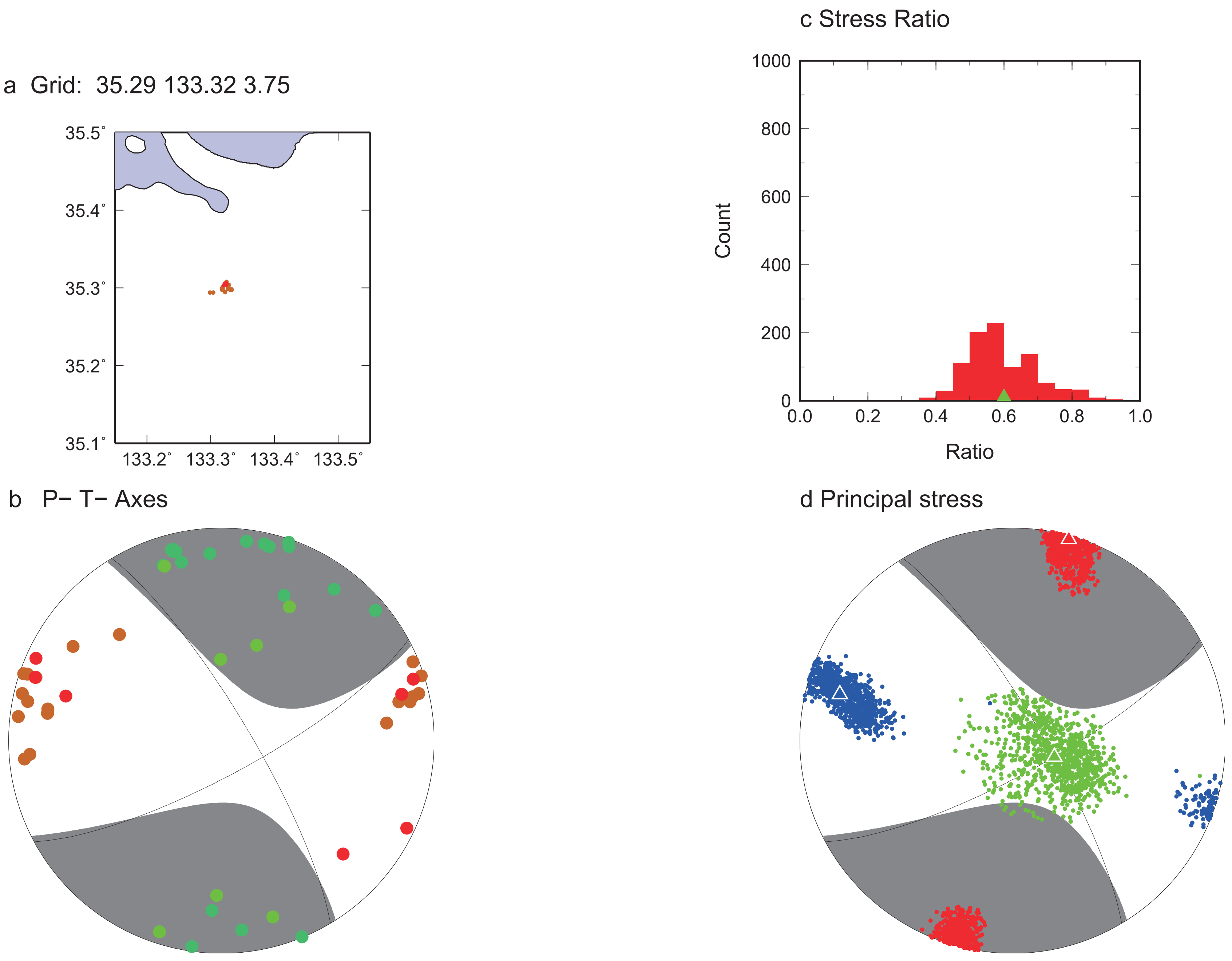
a Grid: 35.29 133.32 3.75
35.5˚
35.4˚
35.3˚
35.2˚
35.1˚
133.2˚ 133.3˚ 133.4˚ 133.5˚
b P− T− Axes
c Stress Ratio
Count
1000
800
600
400
200
0
0.0 0.2 0.4 0.6 0.8 1.0
Ratio
d Principal stress

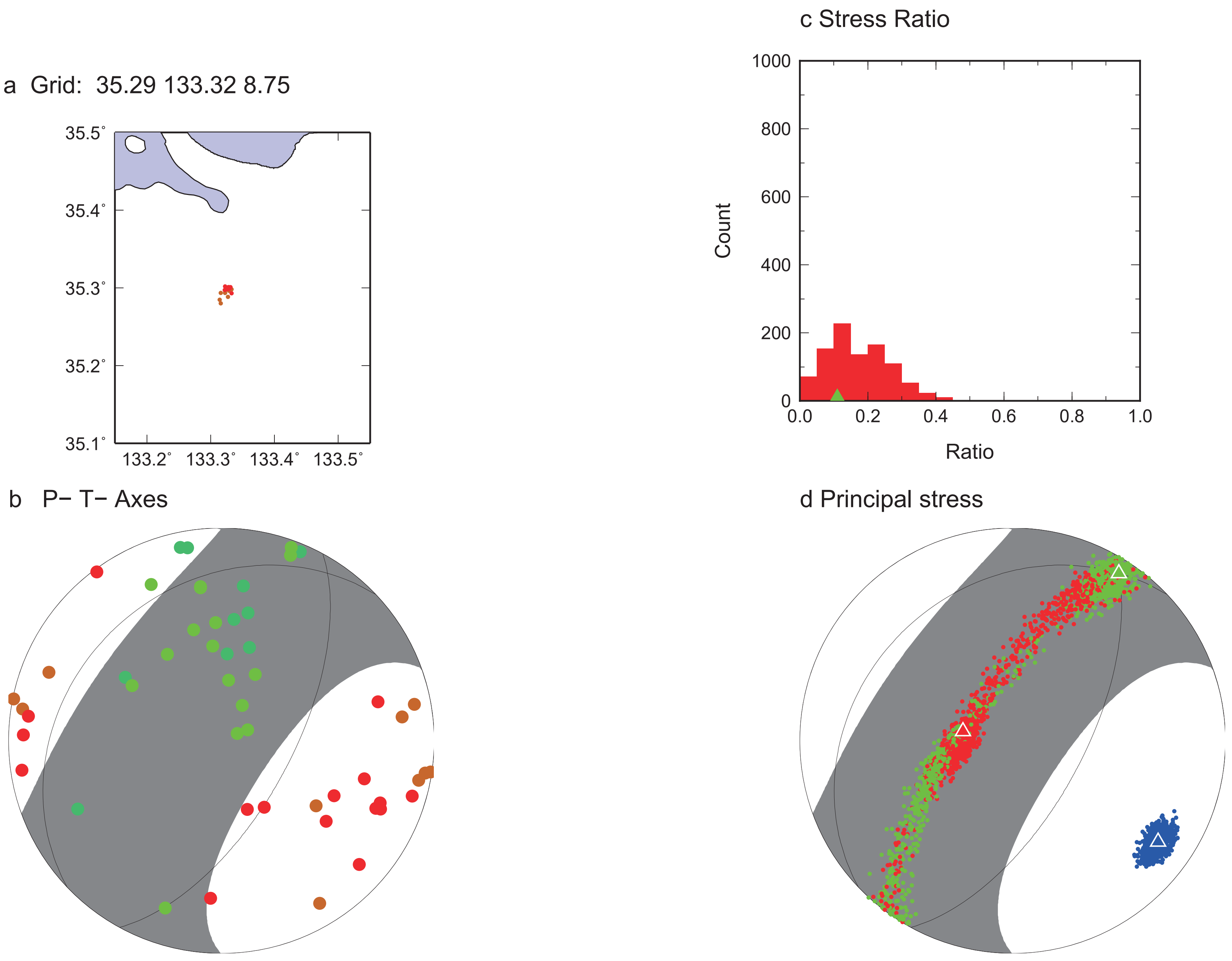

a Grid: 35.29 133.32 8.75
35.5˚
35.4˚
35.3˚
35.2˚
35.1˚
133.2˚
133.3˚
133.4˚
133.5˚
b P– T– Axes
c Stress Ratio
1000
800
600
400
200
0
Count
0.0
0.2
0.4
0.6
0.8
1.0
Ratio
d Principal stress

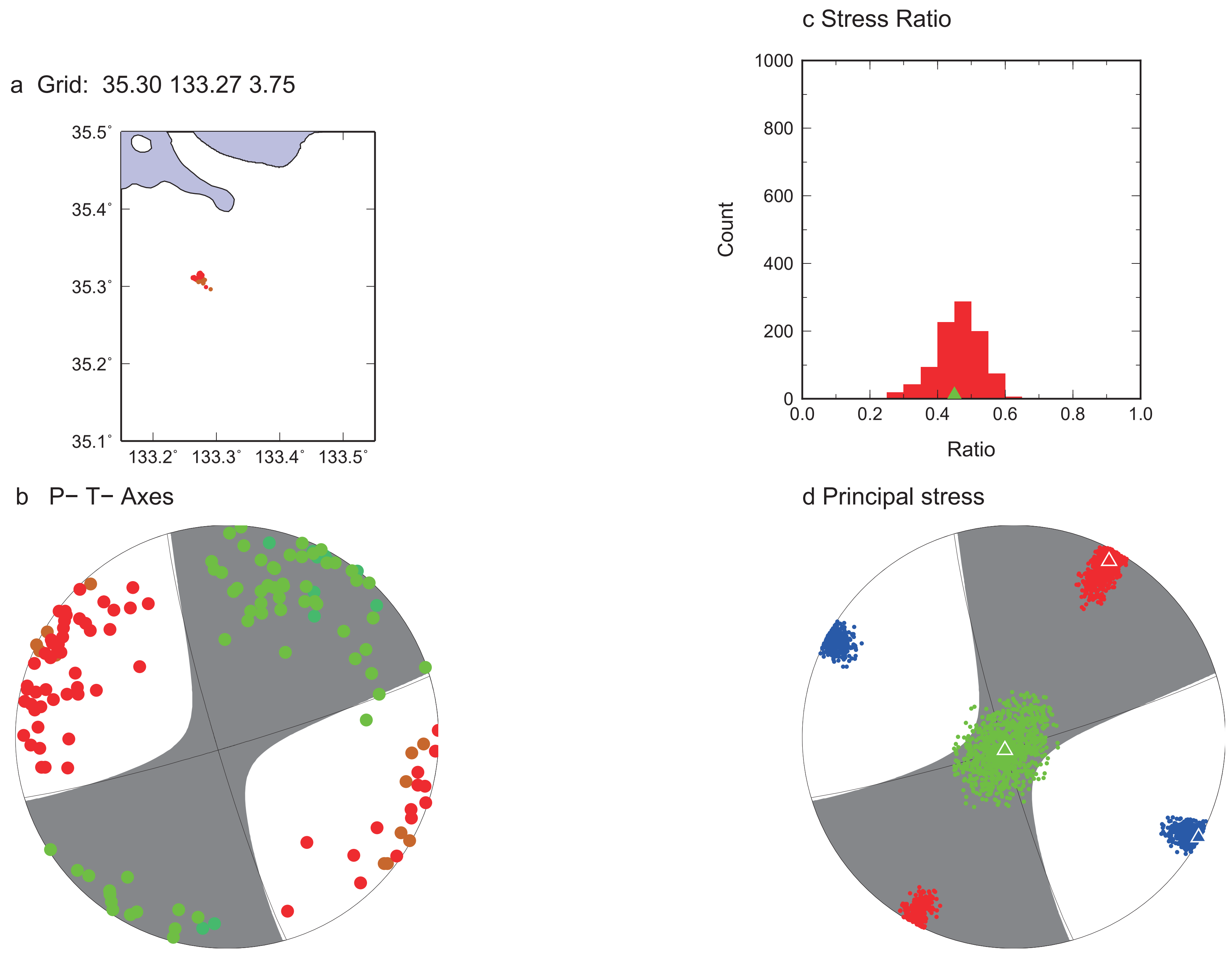
a Grid: 35.30 133.27 3.75
35.5˚
35.4˚
35.3˚
35.2˚
35.1˚
133.2˚
133.3˚
133.4˚
133.5˚
b P– T– Axes
c Stress Ratio
1000
800
600
400
200
0
Count
0.0
0.2
0.4
0.6
0.8
1.0
Ratio
d Principal stress

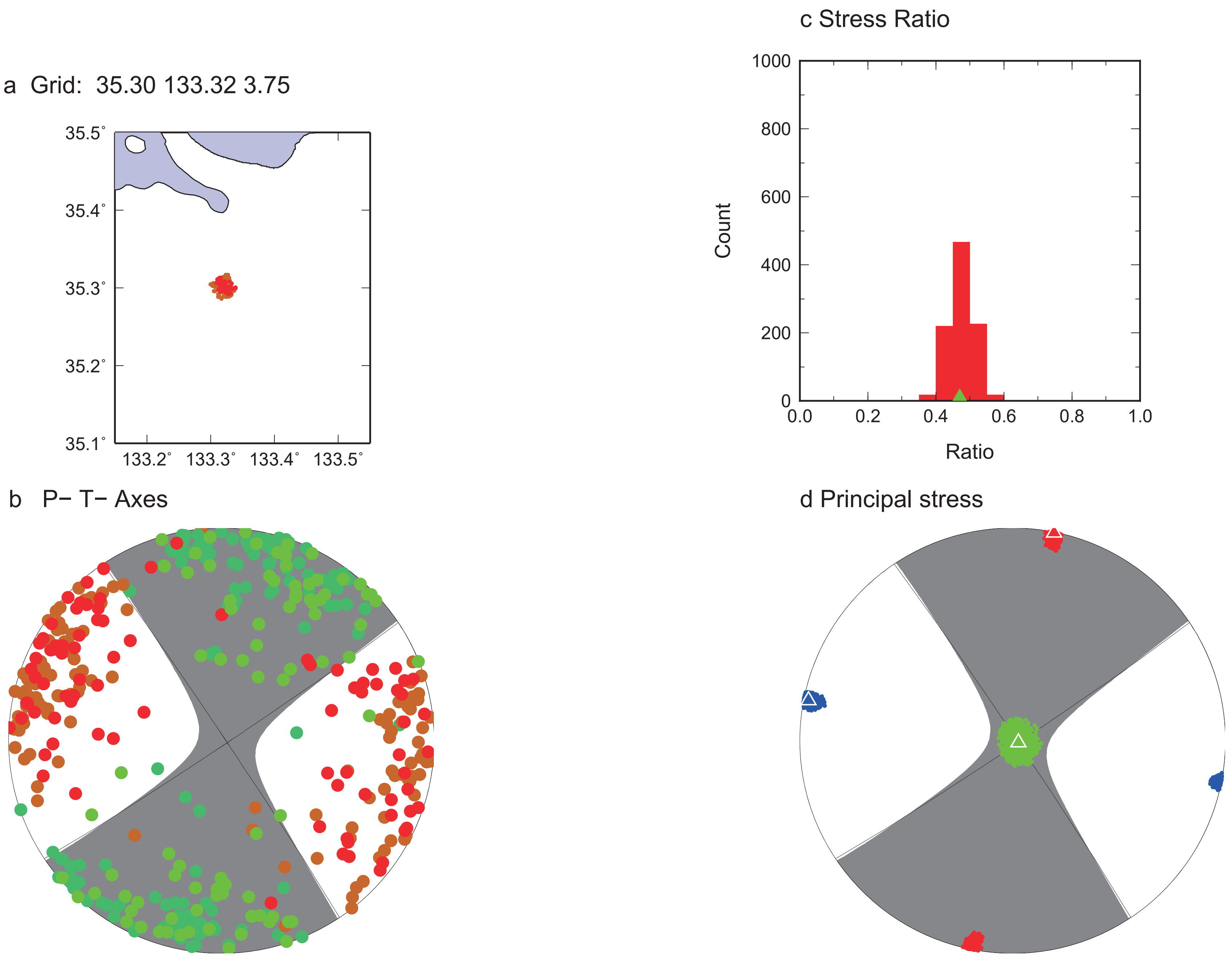
a Grid: 35.30 133.32 3.75
35.5˚
35.4˚
35.3˚
35.2˚
35.1˚
133.2˚
133.3˚
133.4˚
133.5˚
b P− T− Axes
c Stress Ratio
1000
800
600
400
200
0
Count
0.0
0.2
0.4
0.6
0.8
1.0
Ratio
d Principal stress

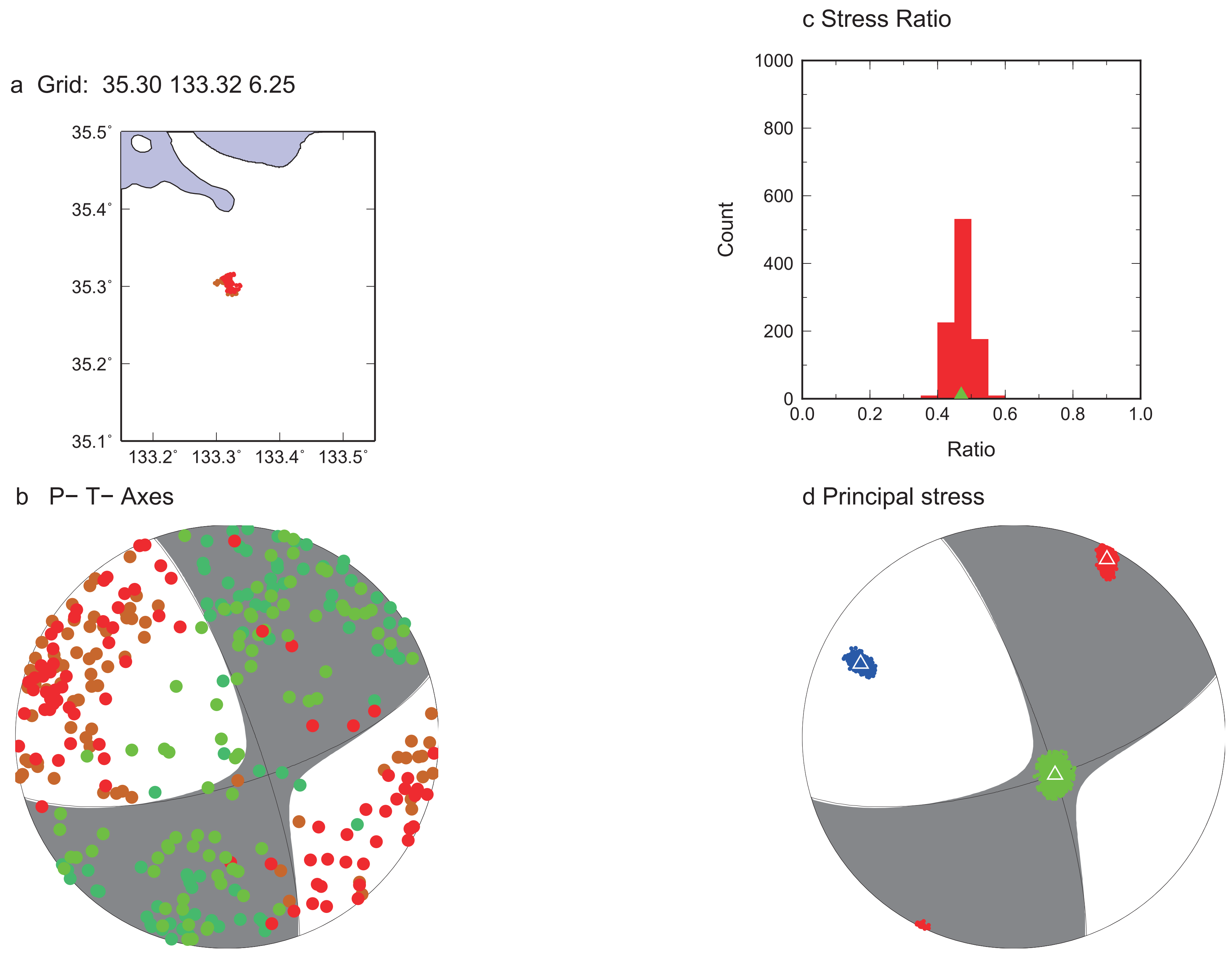
a Grid: 35.30 133.32 6.25
35.5˚
35.4˚
35.3˚
35.2˚
35.1˚
133.2˚
133.3˚
133.4˚
133.5˚
b P− T− Axes
c Stress Ratio
1000
800
600
400
200
0
Count
0.0
0.2
0.4
0.6
0.8
1.0
Ratio
d Principal stress

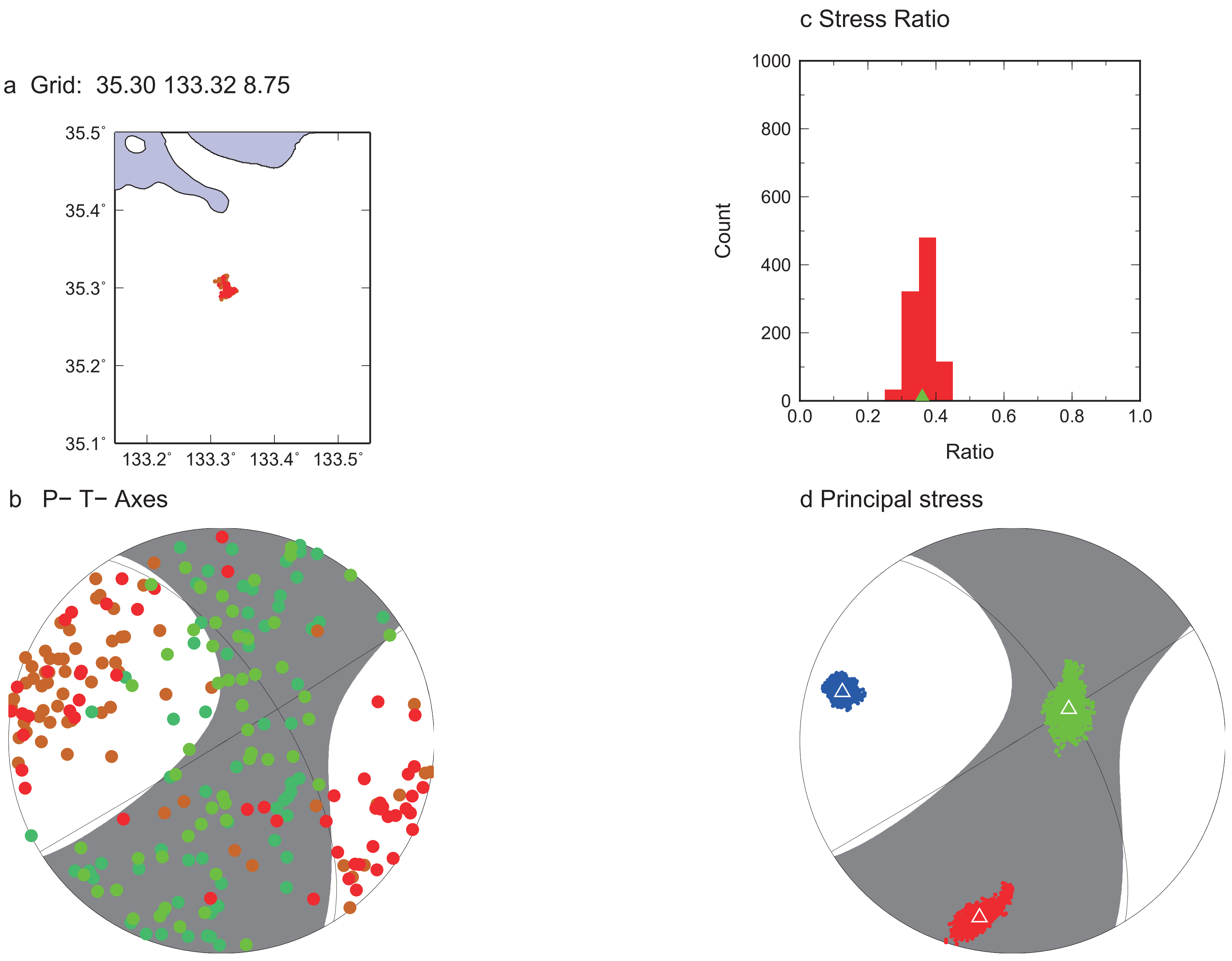
a Grid: 35.30 133.32 8.75
35.5˚
35.4˚
35.3˚
35.2˚
35.1˚
133.2˚
133.3˚
133.4˚
133.5˚
b P− T− Axes
c Stress Ratio
1000
800
600
400
200
0
Count
0.0
0.2
0.4
0.6
0.8
1.0
Ratio
d Principal stress

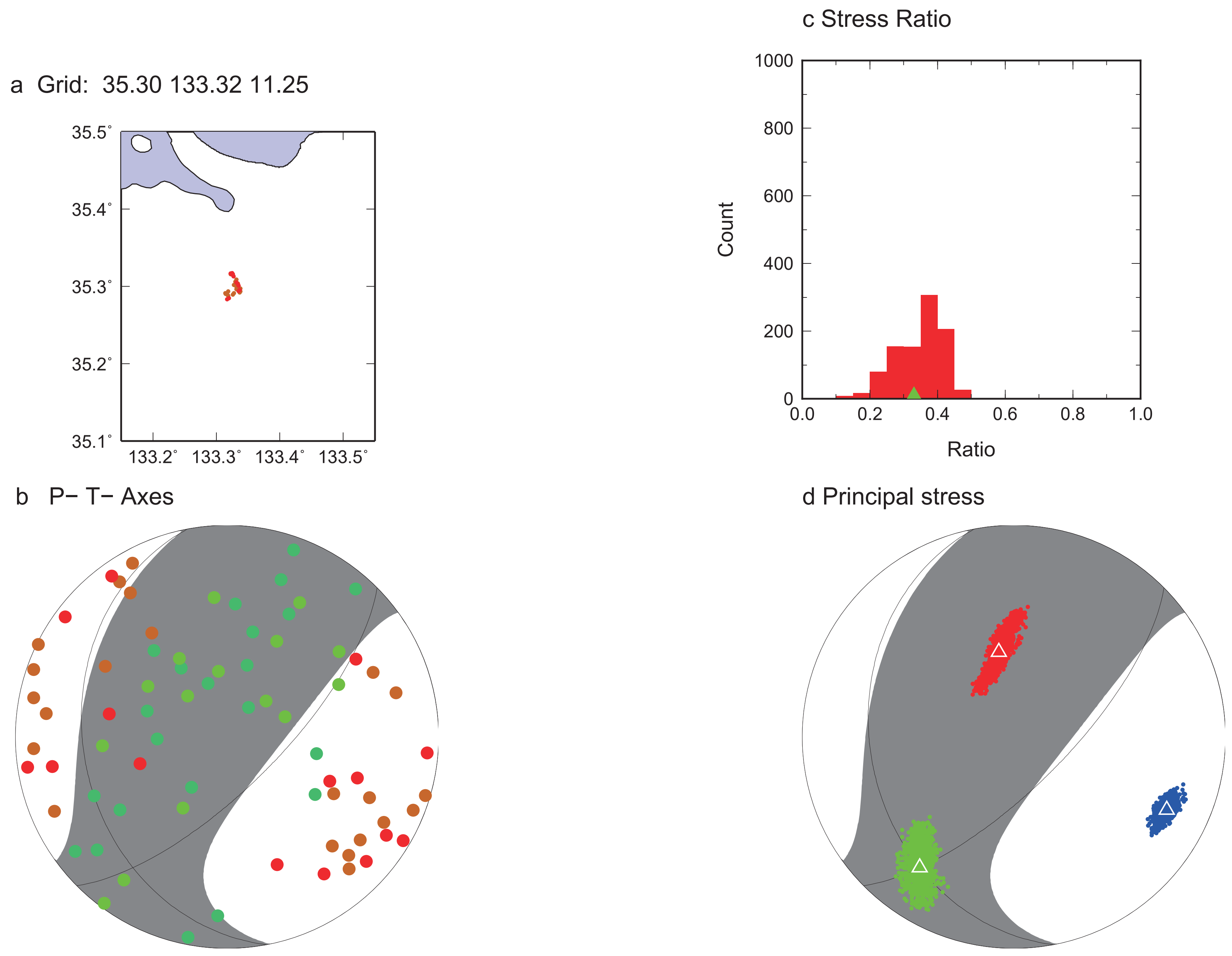
a Grid: 35.30 133.32 11.25
35.5˚
35.4˚
35.3˚
35.2˚
35.1˚
133.2˚
133.3˚
133.4˚
133.5˚
c Stress Ratio
1000
800
600
400
200
0
Count
0.0
0.2
0.4
0.6
0.8
1.0
Ratio
b P− T− Axes
d Principal stress

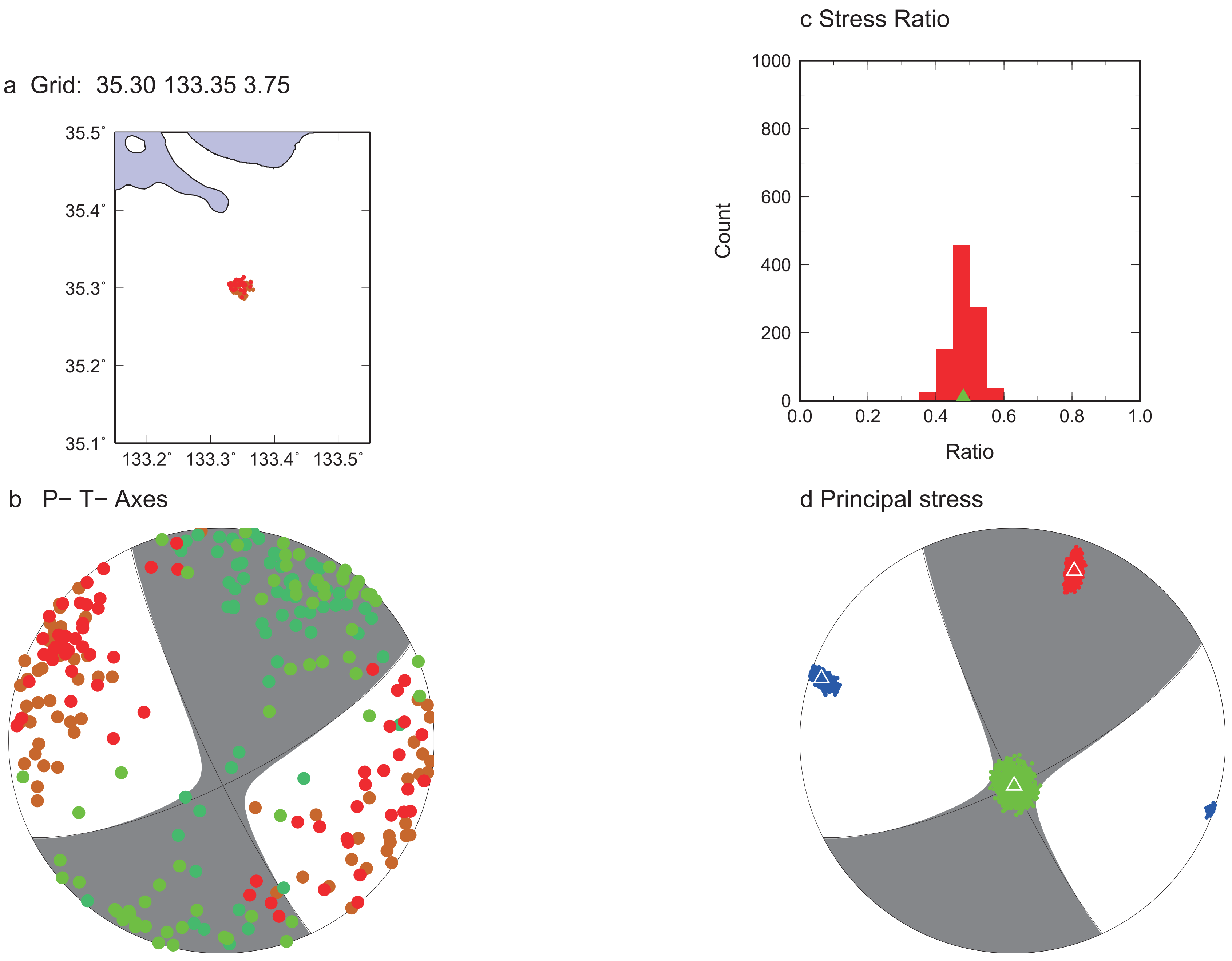

a Grid: 35.30 133.35 3.75
35.5˚
35.4˚
35.3˚
35.2˚
35.1˚
133.2˚
133.3˚
133.4˚
133.5˚
b P– T– Axes
c Stress Ratio
1000
800
600
400
200
0
Count
0.0
0.2
0.4
0.6
0.8
1.0
Ratio
d Principal stress

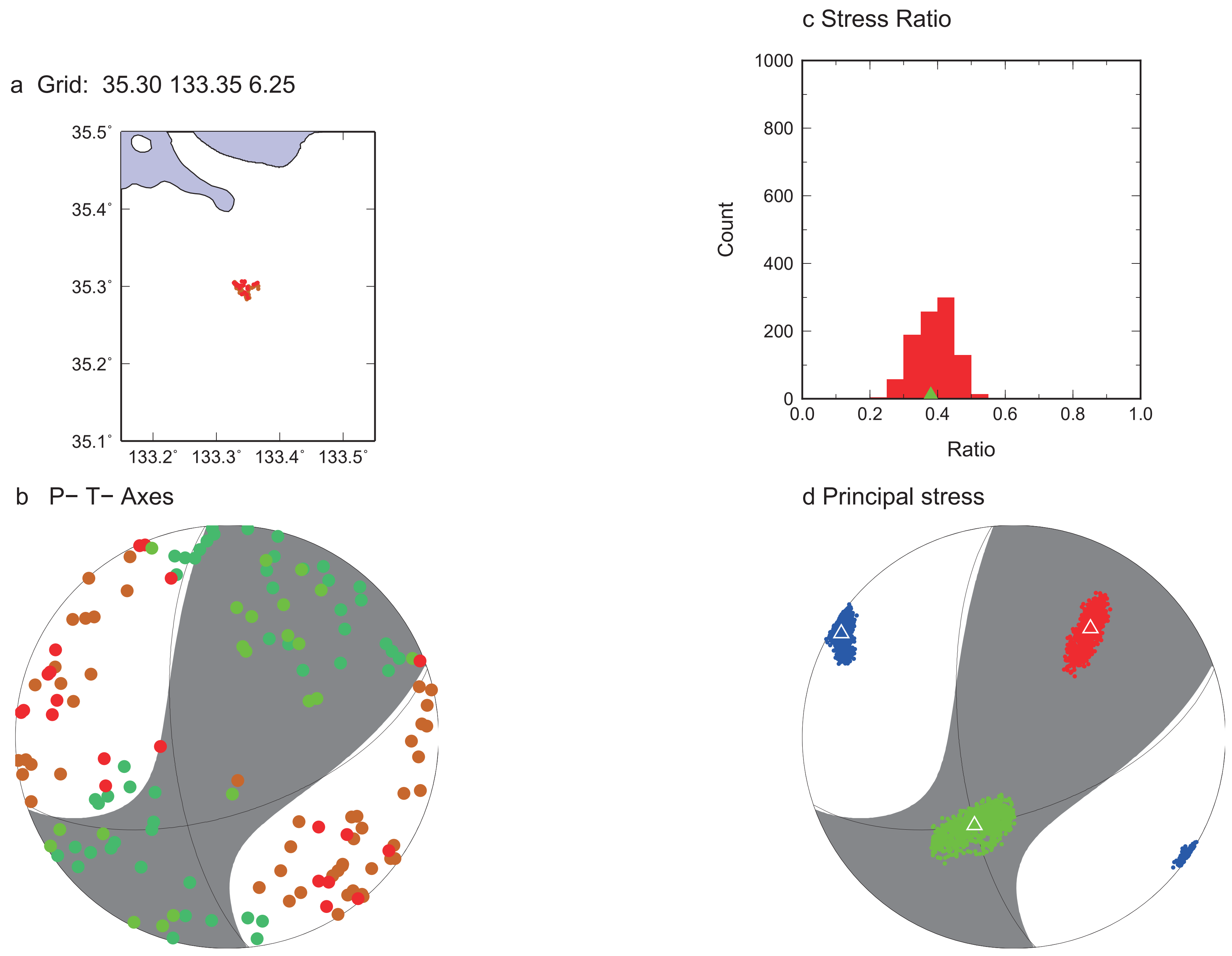
a Grid: 35.30 133.35 6.25
35.5˚
35.4˚
35.3˚
35.2˚
35.1˚
133.2˚
133.3˚
133.4˚
133.5˚
c Stress Ratio
Count
1000
800
600
400
200
0
0.0
0.2
0.4
0.6
0.8
1.0
Ratio
b P− T− Axes
d Principal stress

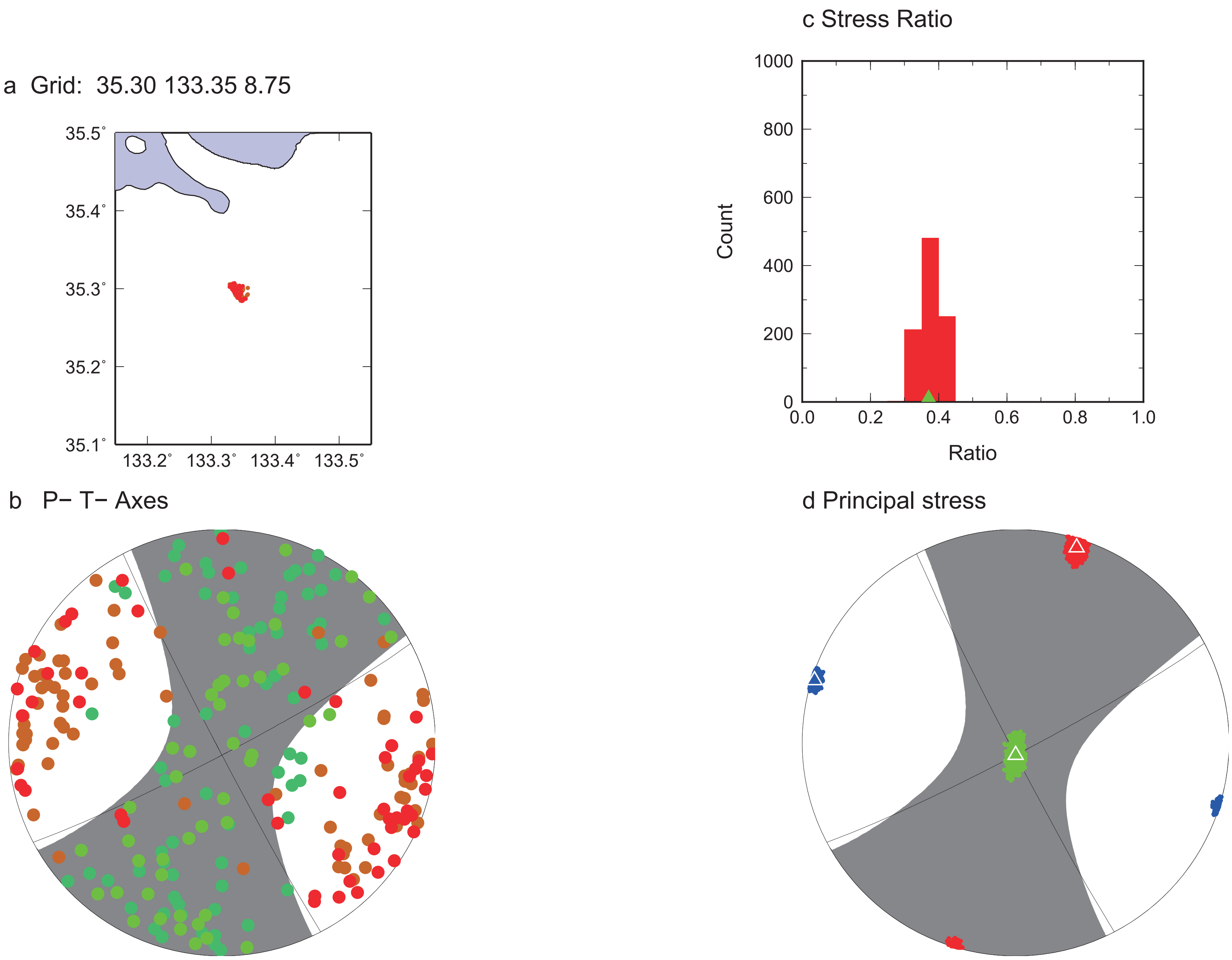

a Grid: 35.30 133.35 8.75
35.5˚
35.4˚
35.3˚
35.2˚
35.1˚
133.2˚
133.3˚
133.4˚
133.5˚
b P− T− Axes
c Stress Ratio
1000
800
600
400
200
0
Count
0.0
0.2
0.4
0.6
0.8
1.0
Ratio
d Principal stress

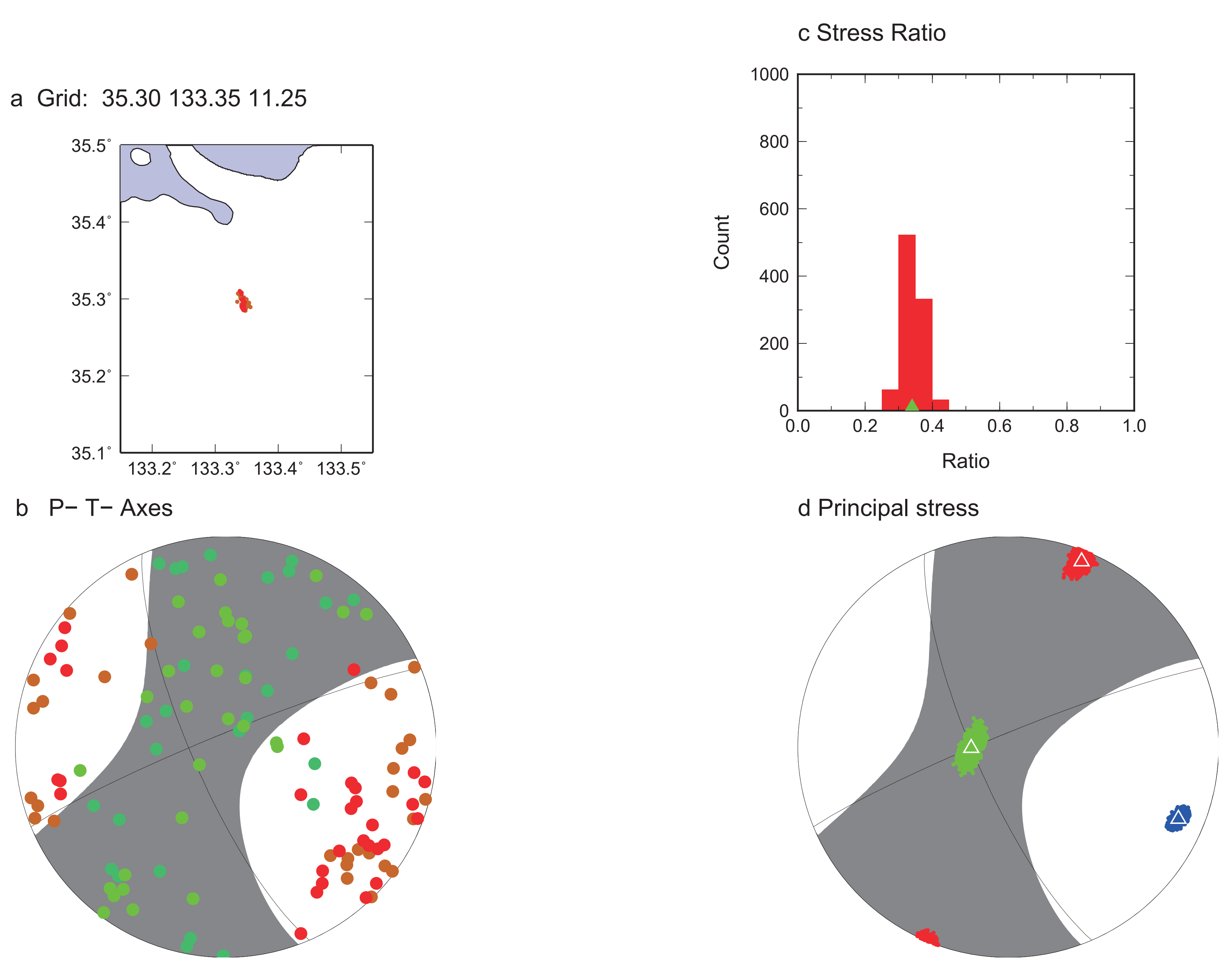
a Grid: 35.30 133.35 11.25
35.5˚
35.4˚
35.3˚
35.2˚
35.1˚
133.2˚
133.3˚
133.4˚
133.5˚
b P− T− Axes
c Stress Ratio
Count
1000
800
600
400
200
0
0.0
0.2
0.4
0.6
0.8
1.0
Ratio
d Principal stress

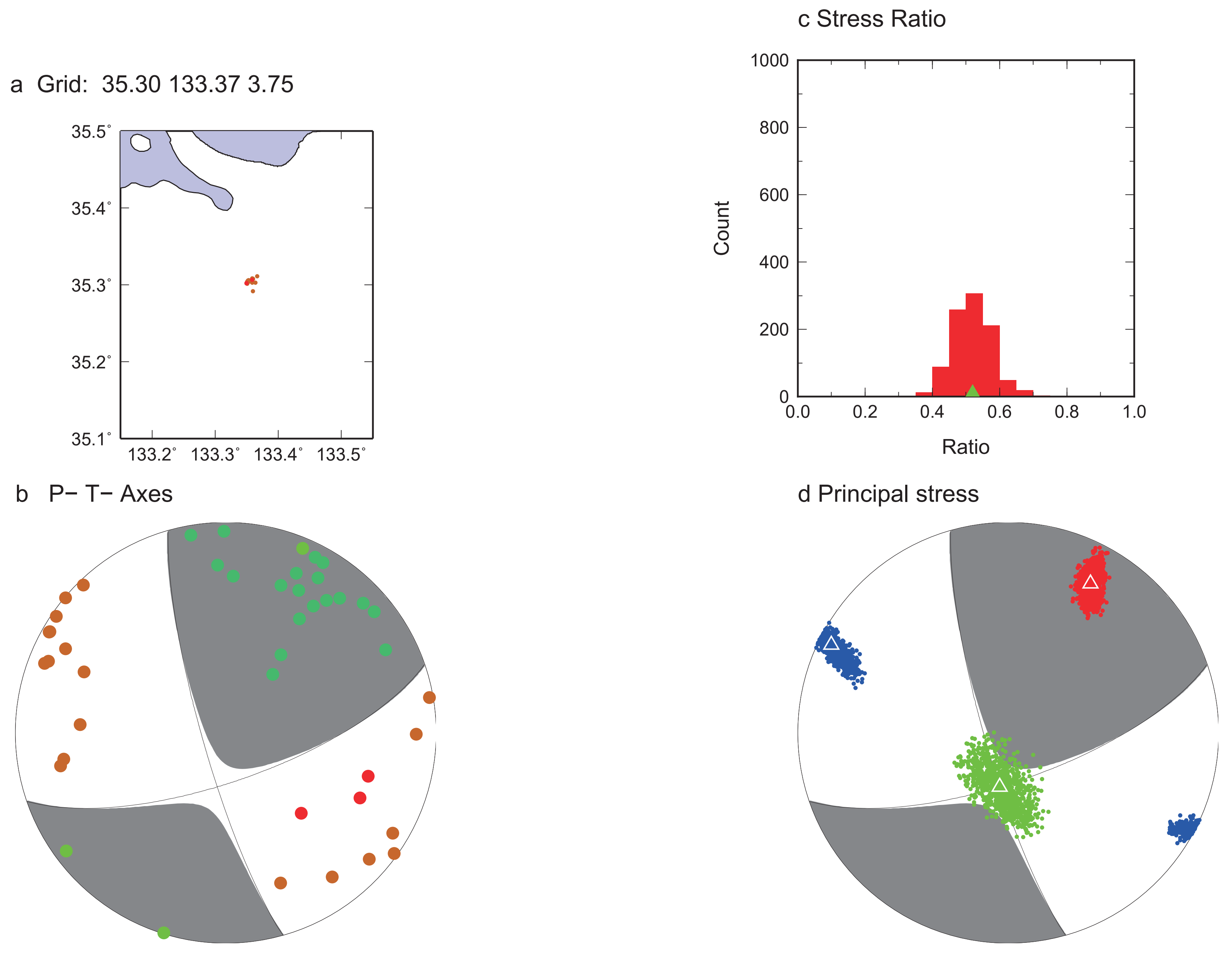
a Grid: 35.30 133.37 3.75
35.5˚
35.4˚
35.3˚
35.2˚
35.1˚
133.2˚ 133.3˚ 133.4˚ 133.5˚
b P− T− Axes
c Stress Ratio
1000
800
600
400
200
0
Count
0.0 0.2 0.4 0.6 0.8 1.0
Ratio
d Principal stress

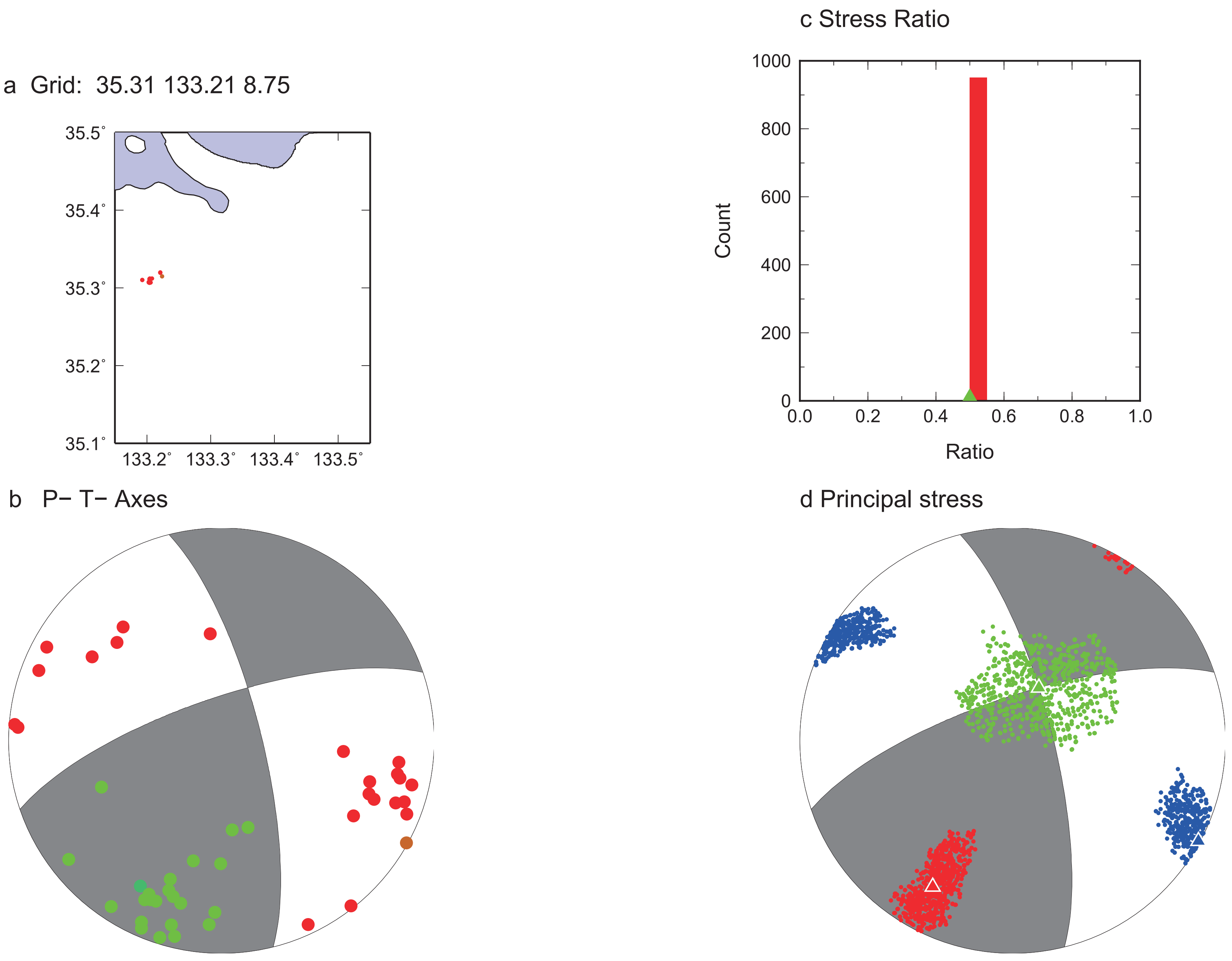

a Grid: 35.31 133.21 8.75
35.5˚
35.4˚
35.3˚
35.2˚
35.1˚
133.2˚
133.3˚
133.4˚
133.5˚
b P− T− Axes
c Stress Ratio
Count
1000
800
600
400
200
0
0.0
0.2
0.4
0.6
0.8
1.0
Ratio
d Principal stress

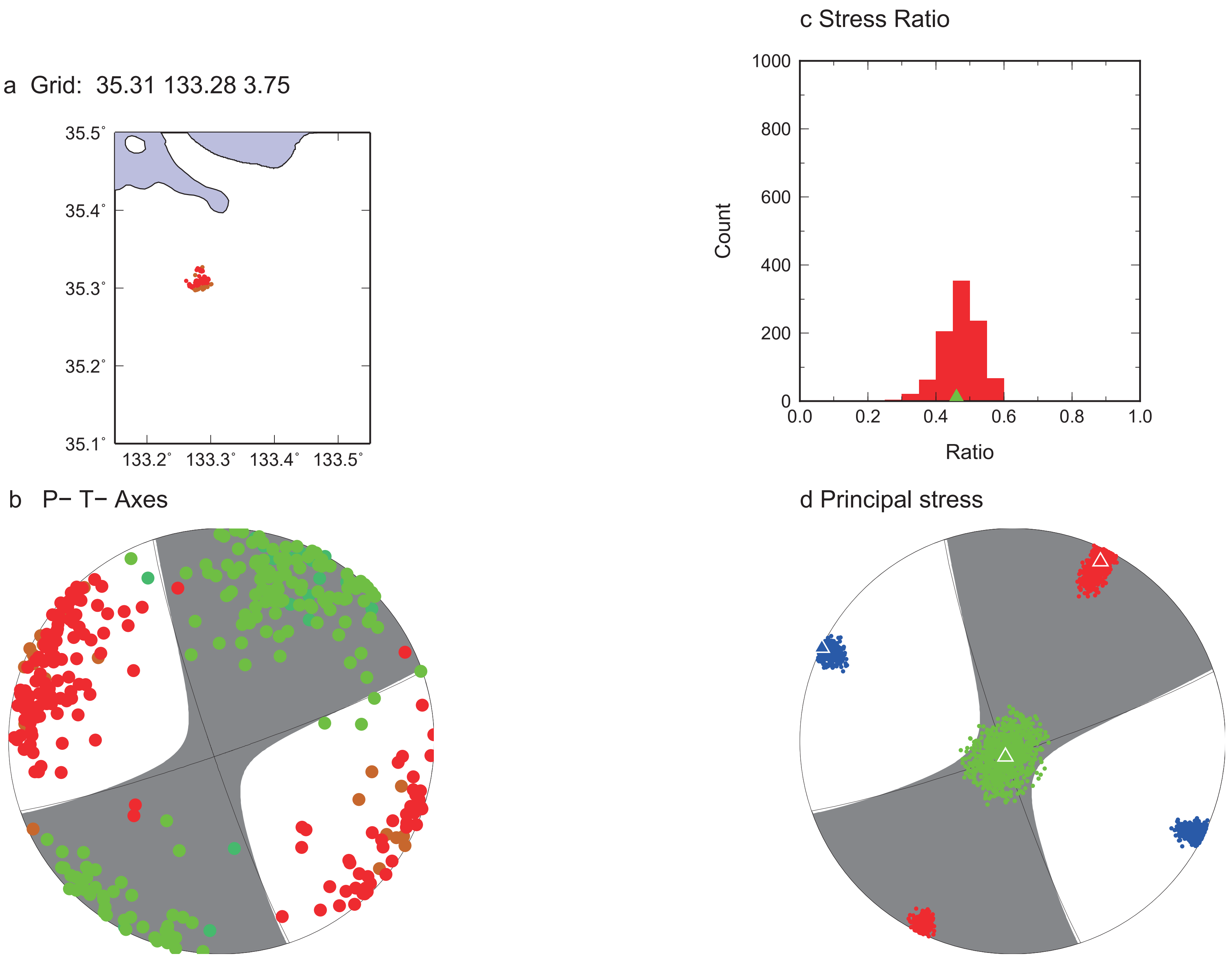
a Grid: 35.31 133.28 3.75
35.5˚
35.4˚
35.3˚
35.2˚
35.1˚
133.2˚
133.3˚
133.4˚
133.5˚
b P− T− Axes
c Stress Ratio
1000
800
600
400
200
0
Count
0.0
0.2
0.4
0.6
0.8
1.0
Ratio
d Principal stress

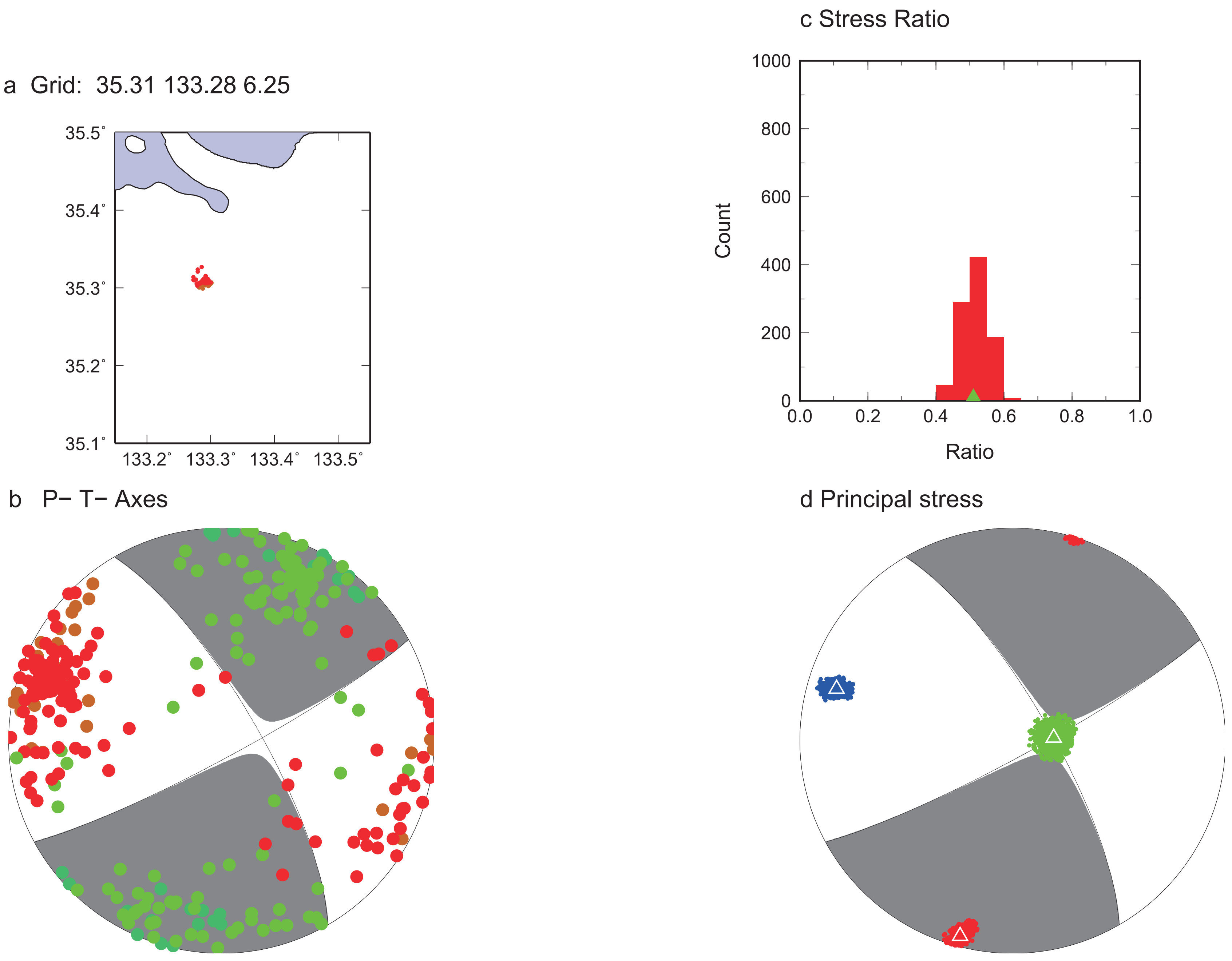
a Grid: 35.31 133.28 6.25
35.5˚
35.4˚
35.3˚
35.2˚
35.1˚
133.2˚
133.3˚
133.4˚
133.5˚
b P− T− Axes
c Stress Ratio
Count
1000
800
600
400
200
0
0.0
0.2
0.4
0.6
0.8
1.0
Ratio
d Principal stress

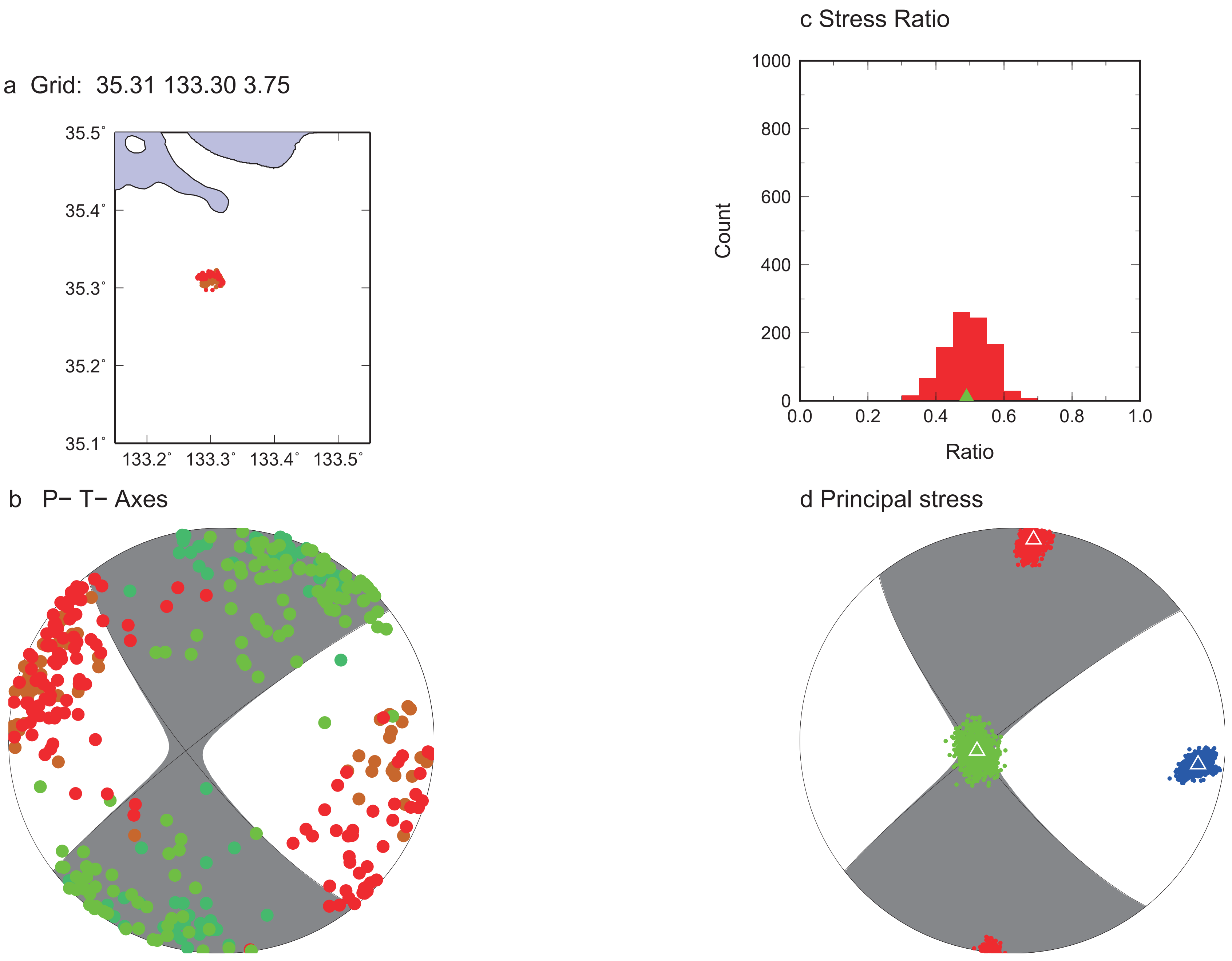
a Grid: 35.31 133.30 3.75
35.5˚
35.4˚
35.3˚
35.2˚
35.1˚
133.2˚
133.3˚
133.4˚
133.5˚
b P− T− Axes
c Stress Ratio
Count
1000
800
600
400
200
0
0.0
0.2
0.4
0.6
0.8
1.0
Ratio
d Principal stress

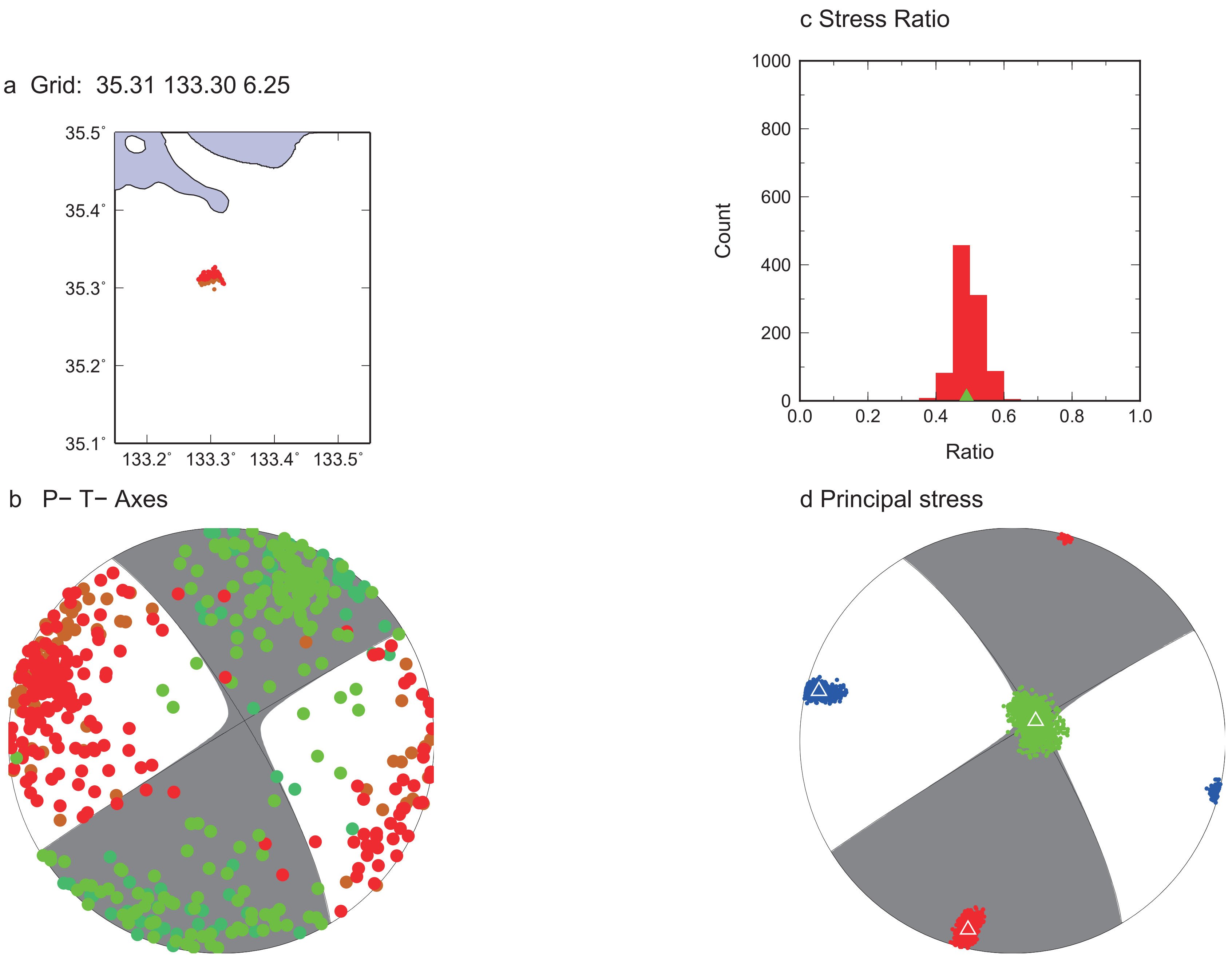
a Grid: 35.31 133.30 6.25
35.5˚
35.4˚
35.3˚
35.2˚
35.1˚
133.2˚
133.3˚
133.4˚
133.5˚
b P− T− Axes
c Stress Ratio
1000
800
600
400
200
0
Count
0.0
0.2
0.4
0.6
0.8
1.0
Ratio
d Principal stress

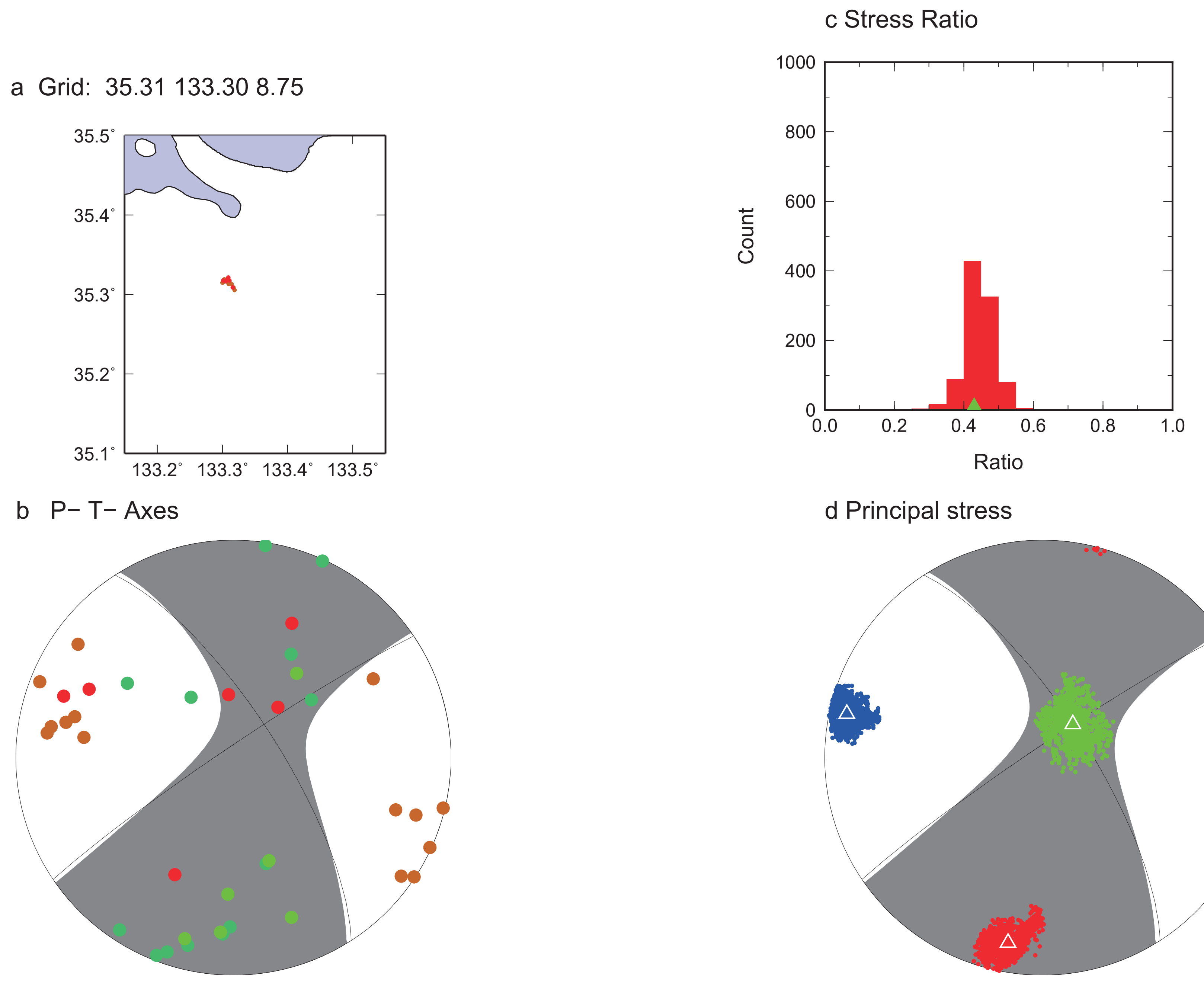
a Grid: 35.31 133.30 8.75
35.5˚
35.4˚
35.3˚
35.2˚
35.1˚
133.2˚
133.3˚
133.4˚
133.5˚
b P− T− Axes
c Stress Ratio
Count
1000
800
600
400
200
0
0.0
0.2
0.4
0.6
0.8
1.0
Ratio
d Principal stress

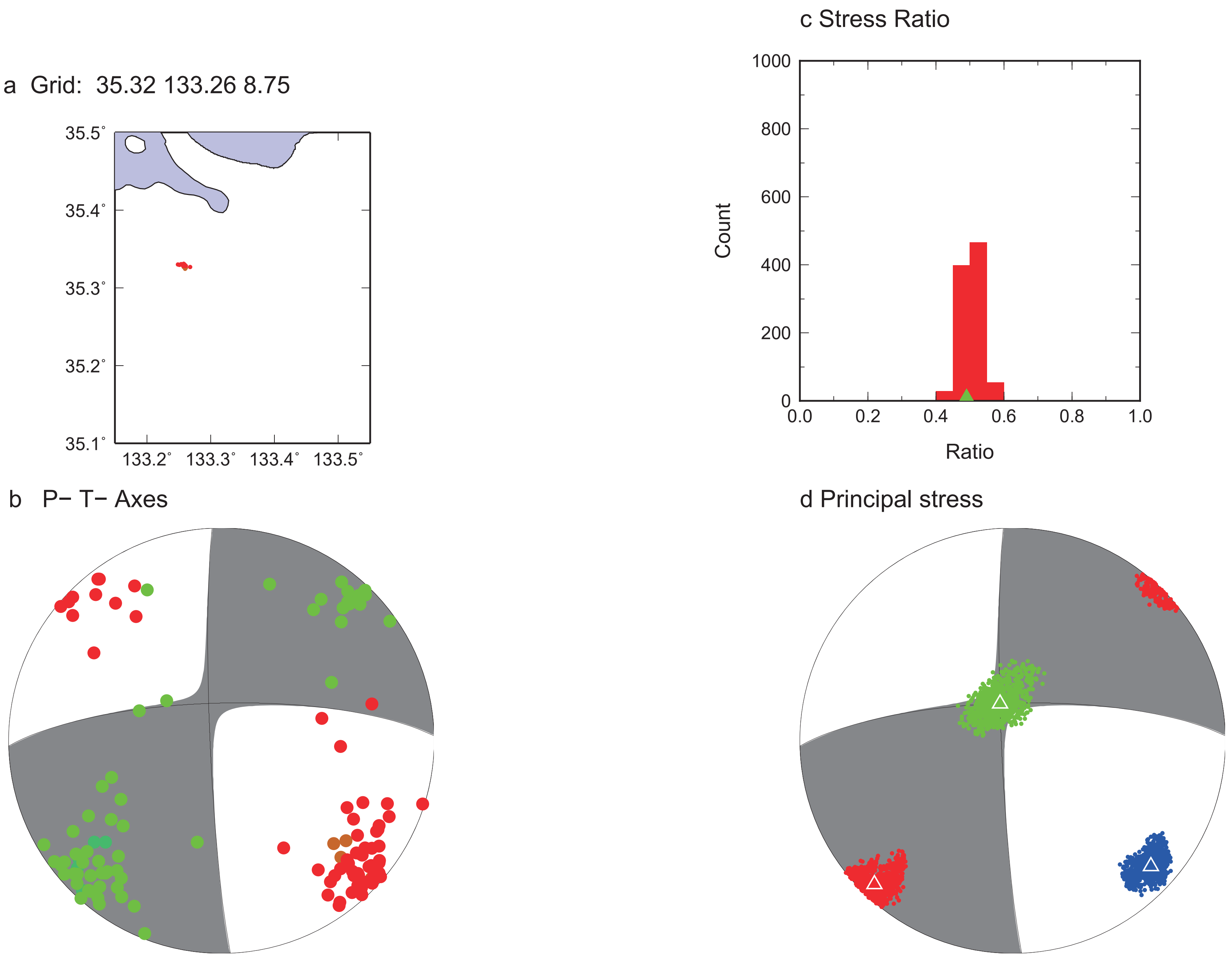
a Grid: 35.32 133.26 8.75
35.5˚
35.4˚
35.3˚
35.2˚
35.1˚
133.2˚
133.3˚
133.4˚
133.5˚
b P− T− Axes
c Stress Ratio
Count
1000
800
600
400
200
0
0.0
0.2
0.4
0.6
0.8
1.0
Ratio
d Principal stress

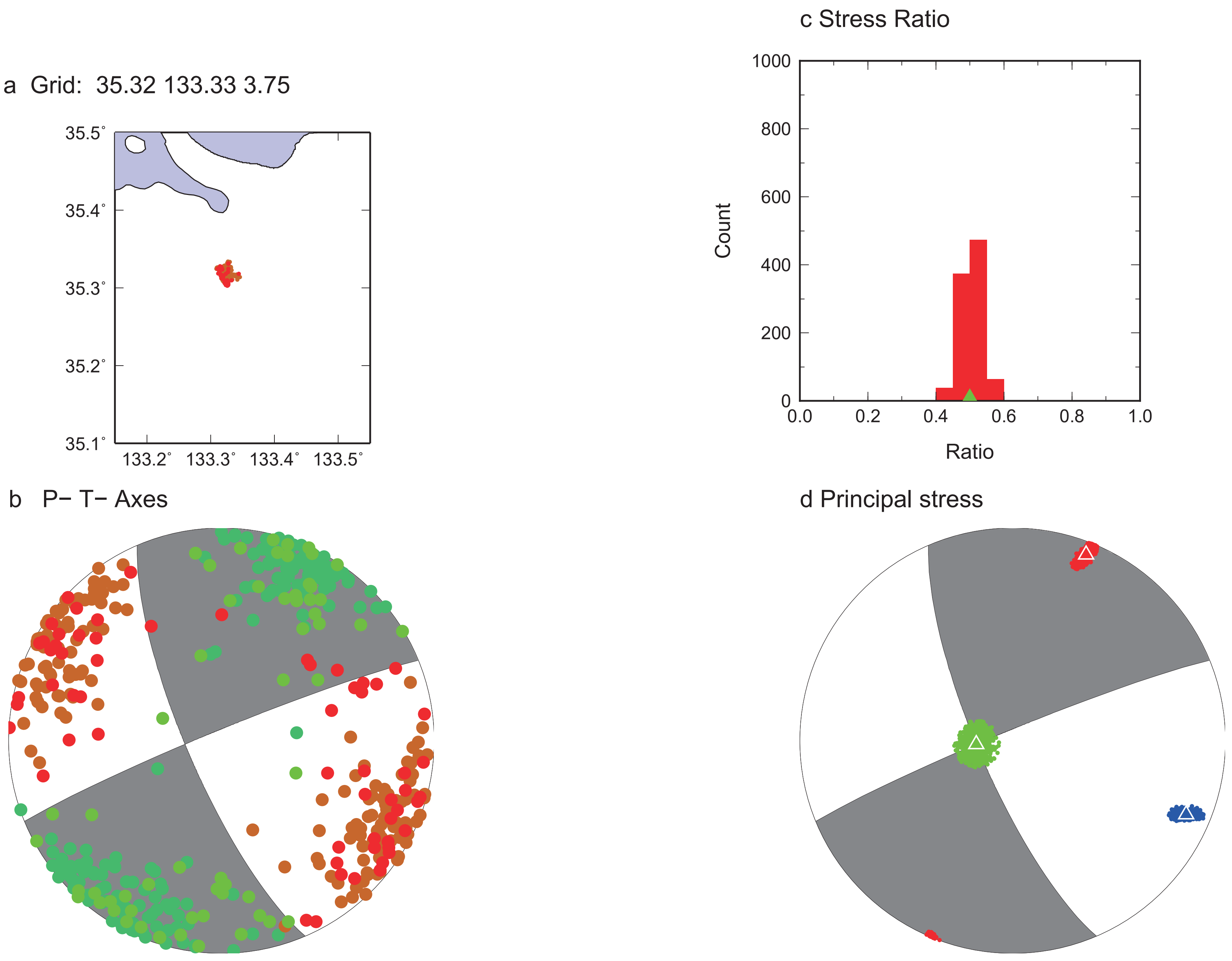
a Grid: 35.32 133.33 3.75
35.5°
35.4°
35.3°
35.2°
35.1°
133.2°
133.3°
133.4°
133.5°
b P– T– Axes
c Stress Ratio
Count
1000
800
600
400
200
0
0.0
0.2
0.4
0.6
0.8
1.0
Ratio
d Principal stress

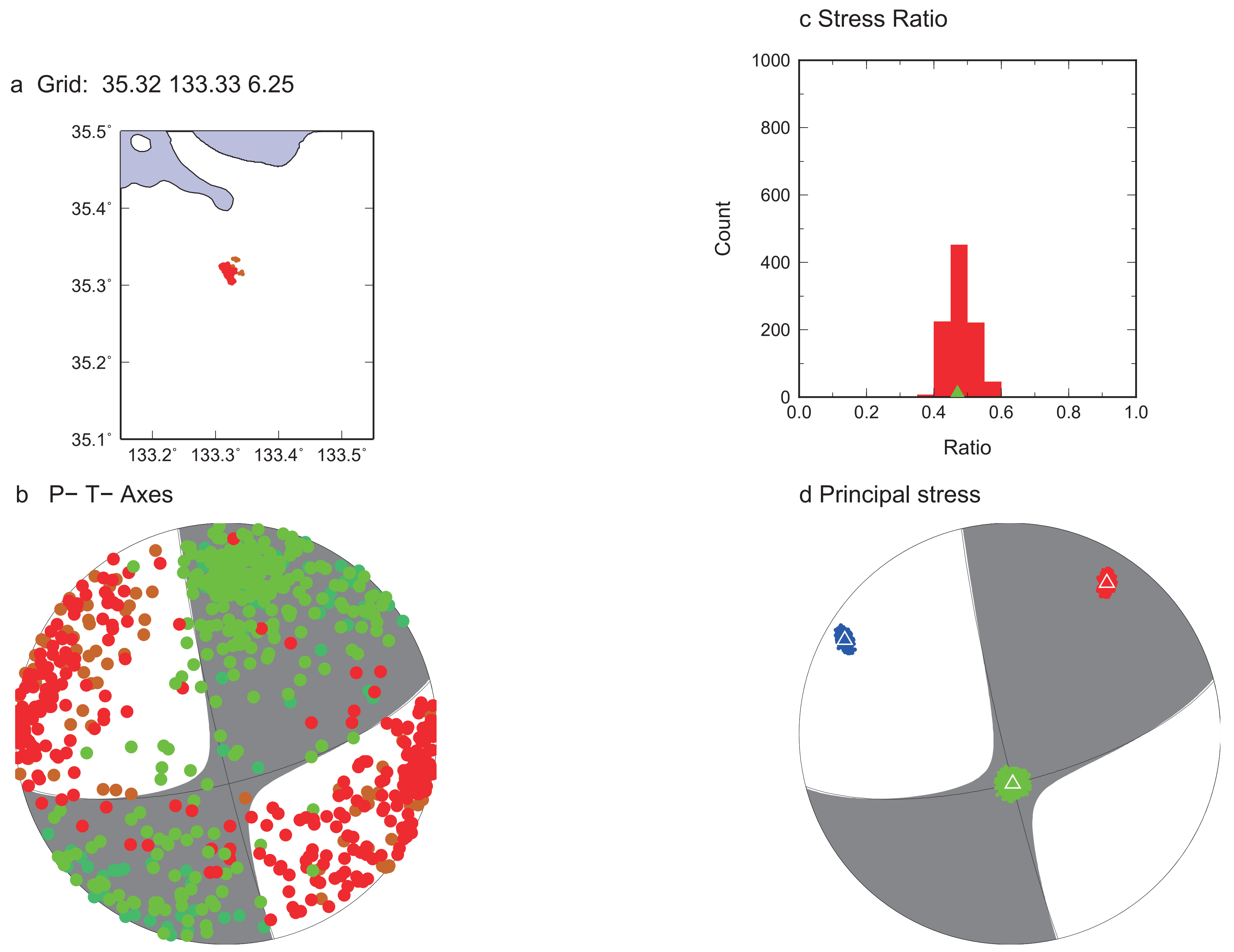
a Grid: 35.32 133.33 6.25
35.5˚
35.4˚
35.3˚
35.2˚
35.1˚
133.2˚ 133.3˚ 133.4˚ 133.5˚
b P– T– Axes
c Stress Ratio
1000
800
600
400
200
0
Count
0.0 0.2 0.4 0.6 0.8 1.0
Ratio
d Principal stress

a Grid: 35.32 133.33 8.75

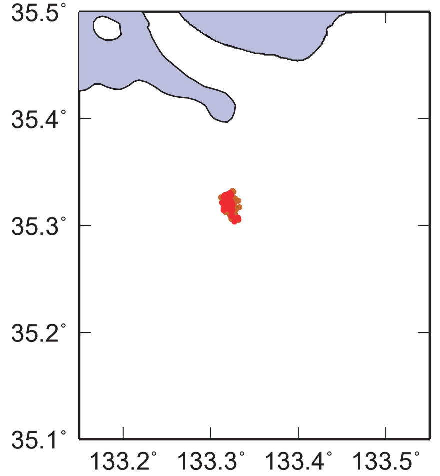

c Stress Ratio

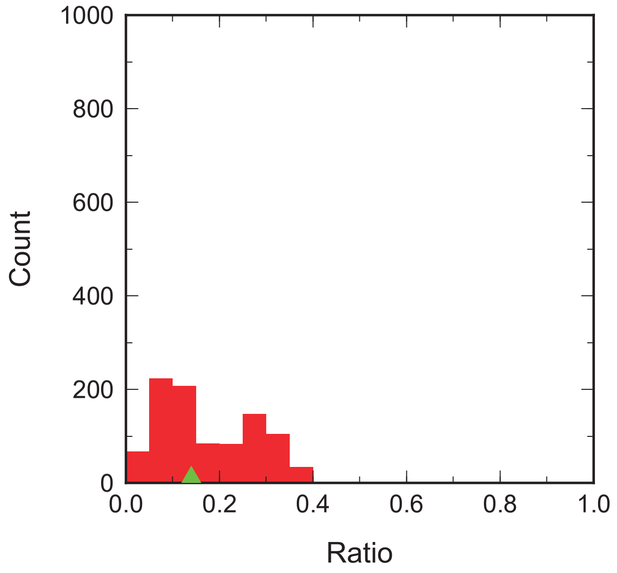

b P− T− Axes

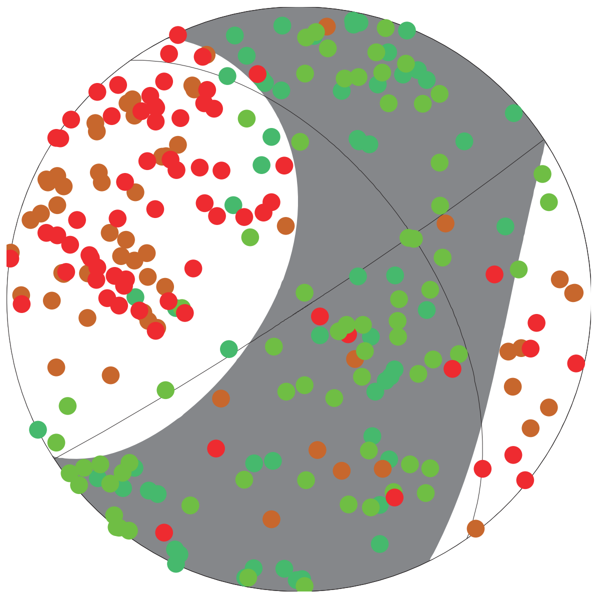

d Principal stress

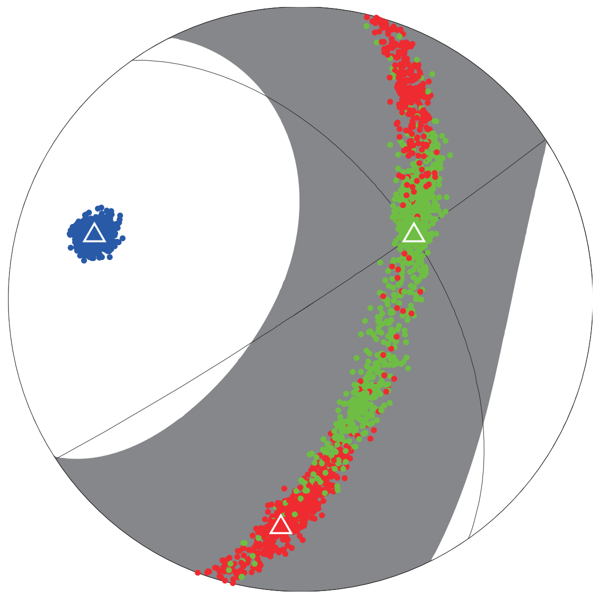

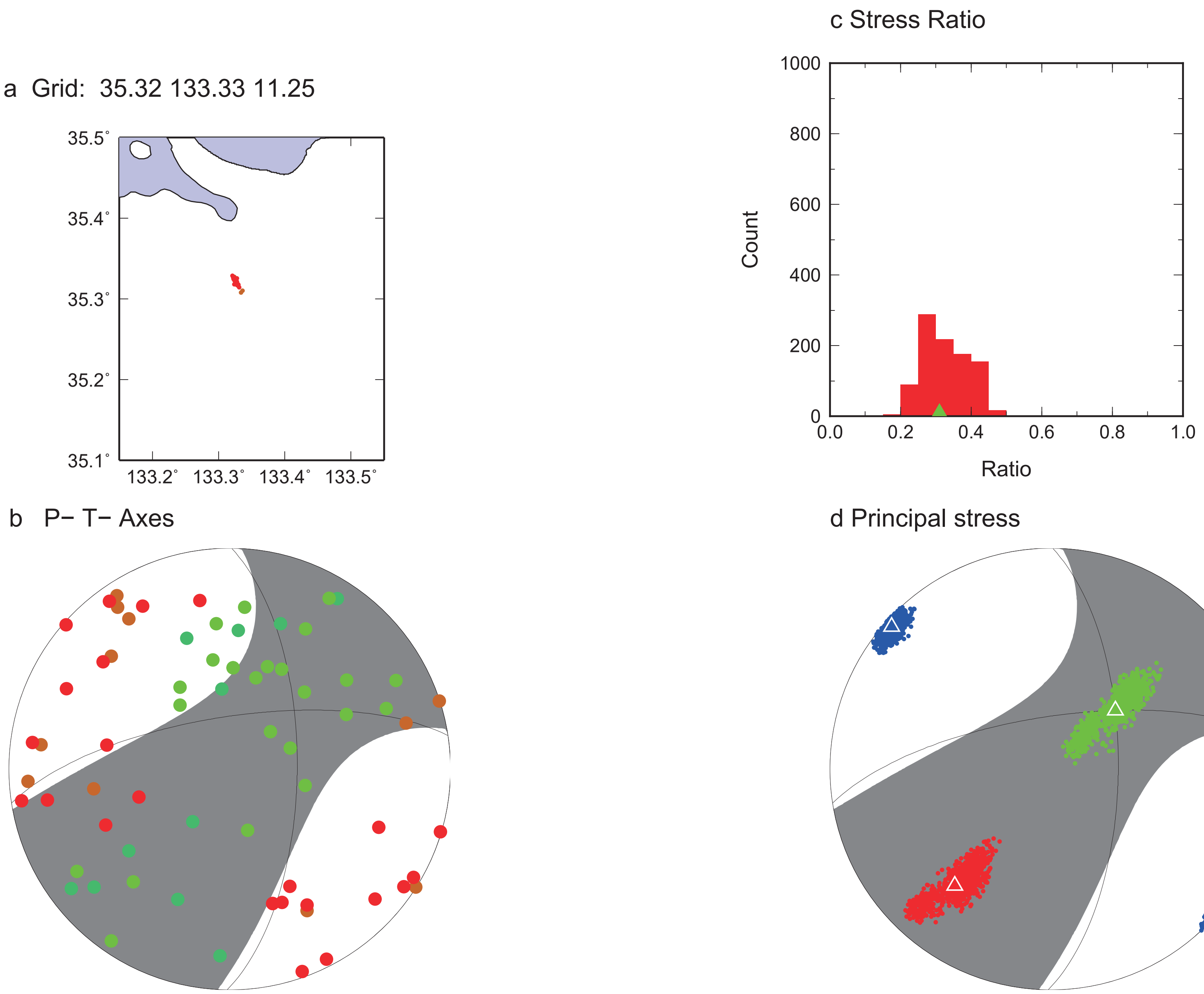
a Grid: 35.32 133.33 11.25
35.5˚
35.4˚
35.3˚
35.2˚
35.1˚
133.2˚
133.3˚
133.4˚
133.5˚
b P− T− Axes
c Stress Ratio
Count
1000
800
600
400
200
0
0.0
0.2
0.4
0.6
0.8
1.0
Ratio
d Principal stress

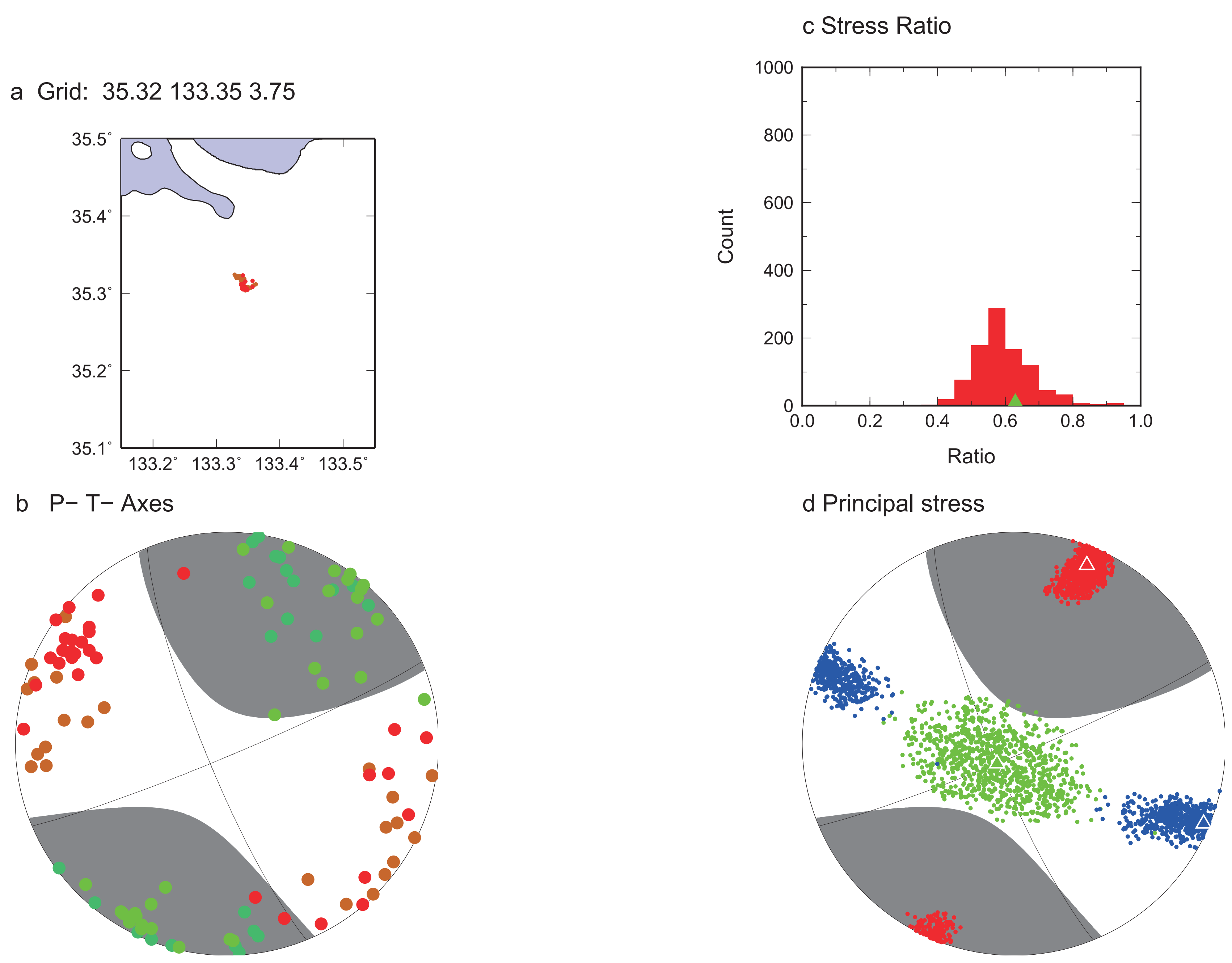
a Grid: 35.32 133.35 3.75
35.5˚
35.4˚
35.3˚
35.2˚
35.1˚
133.2˚ 133.3˚ 133.4˚ 133.5˚
b P– T– Axes
c Stress Ratio
1000
800
600
400
200
0
Count
0.0 0.2 0.4 0.6 0.8 1.0
Ratio
d Principal stress

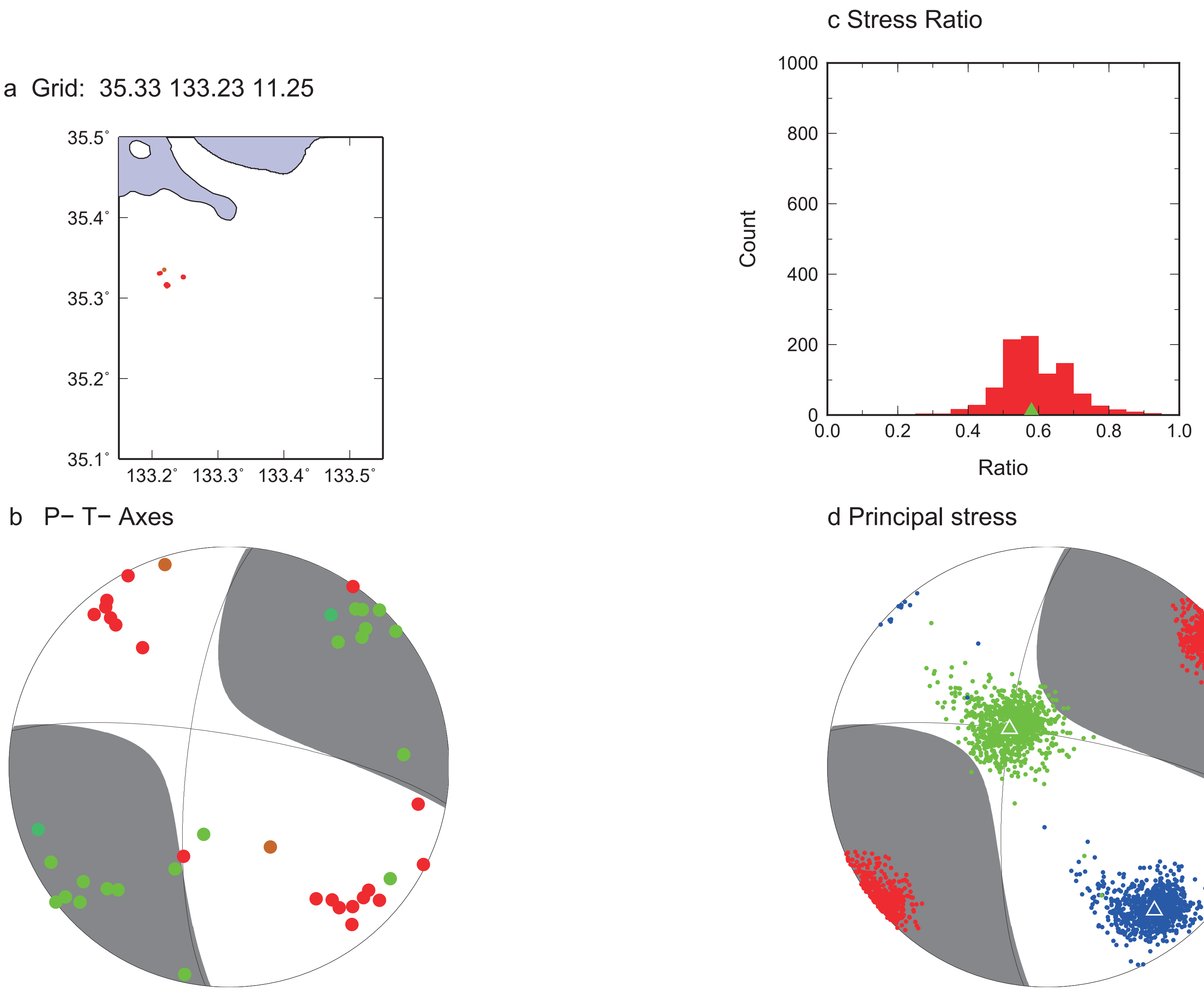

a Grid: 35.33 133.23 11.25
35.5˚
35.4˚
35.3˚
35.2˚
35.1˚
133.2˚
133.3˚
133.4˚
133.5˚
b P− T− Axes
c Stress Ratio
1000
800
600
400
200
0
Count
0.0
0.2
0.4
0.6
0.8
1.0
Ratio
d Principal stress

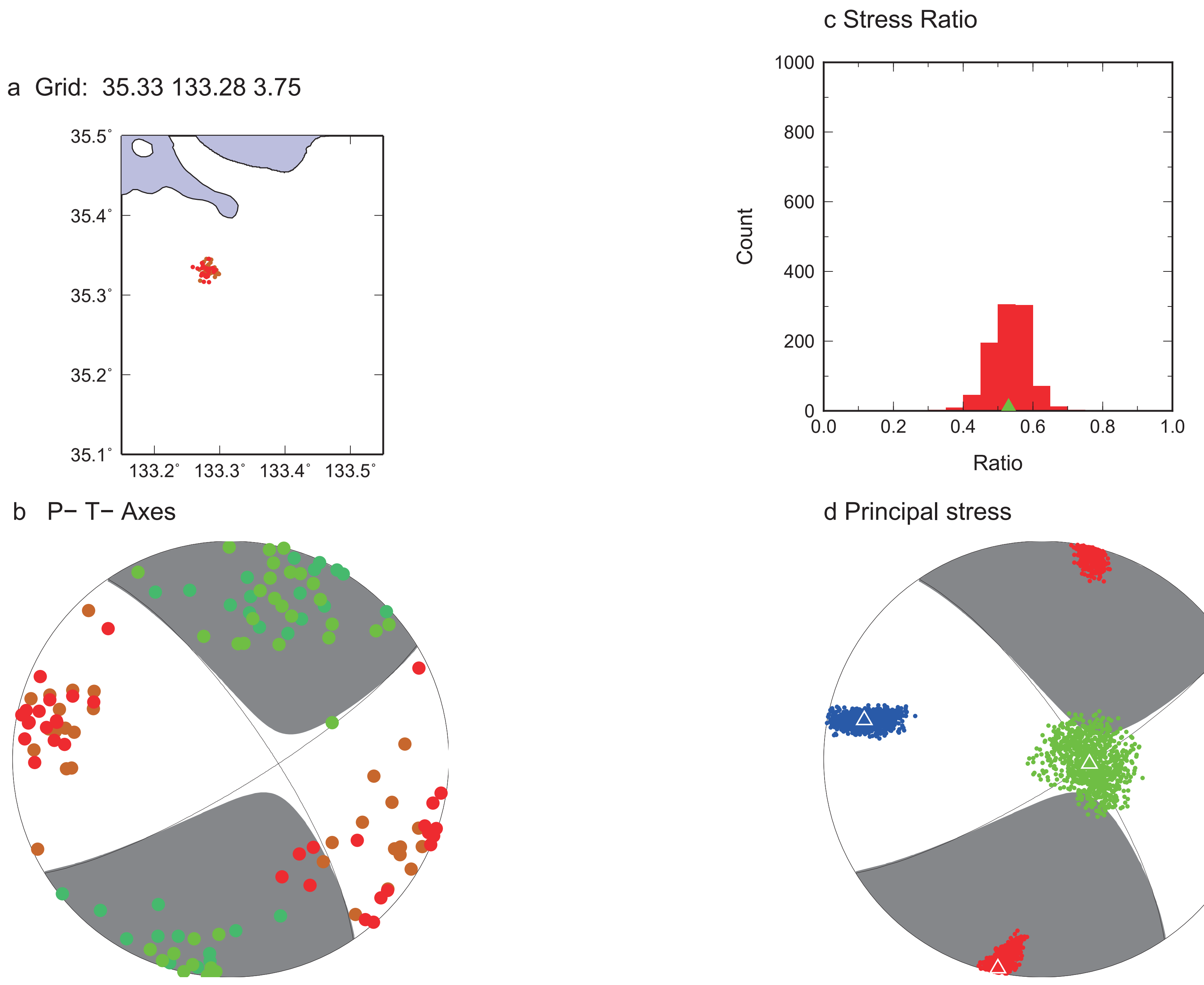
a Grid: 35.33 133.28 3.75
35.5˚
35.4˚
35.3˚
35.2˚
35.1˚
133.2˚
133.3˚
133.4˚
133.5˚
c Stress Ratio
1000
800
600
400
200
0
Count
0.0
0.2
0.4
0.6
0.8
1.0
Ratio
b P− T− Axes
d Principal stress

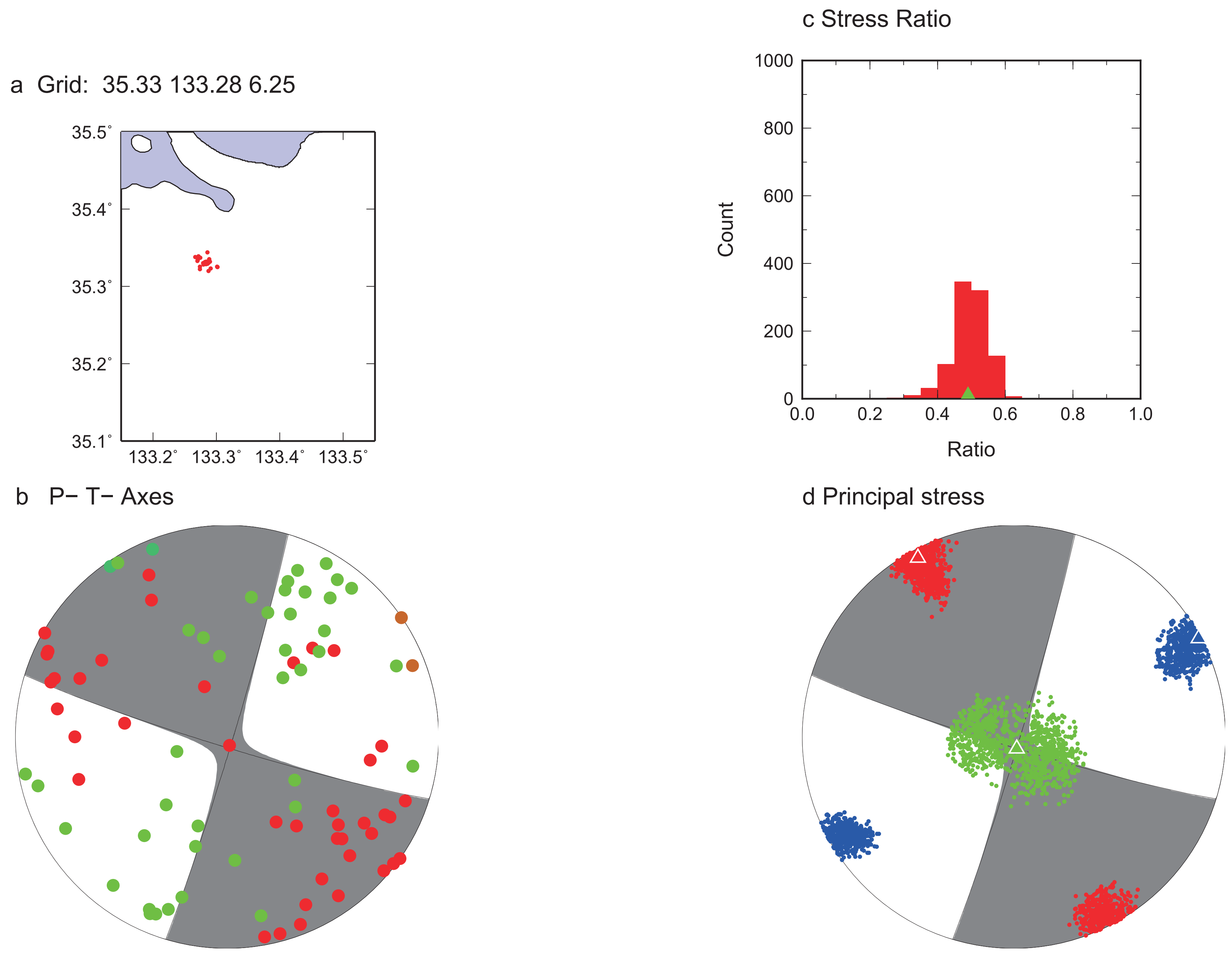
a Grid: 35.33 133.28 6.25
35.5˚
35.4˚
35.3˚
35.2˚
35.1˚
133.2˚ 133.3˚ 133.4˚ 133.5˚
c Stress Ratio
1000
800
600
400
200
0
Count
0.0 0.2 0.4 0.6 0.8 1.0
Ratio
b P− T− Axes
d Principal stress

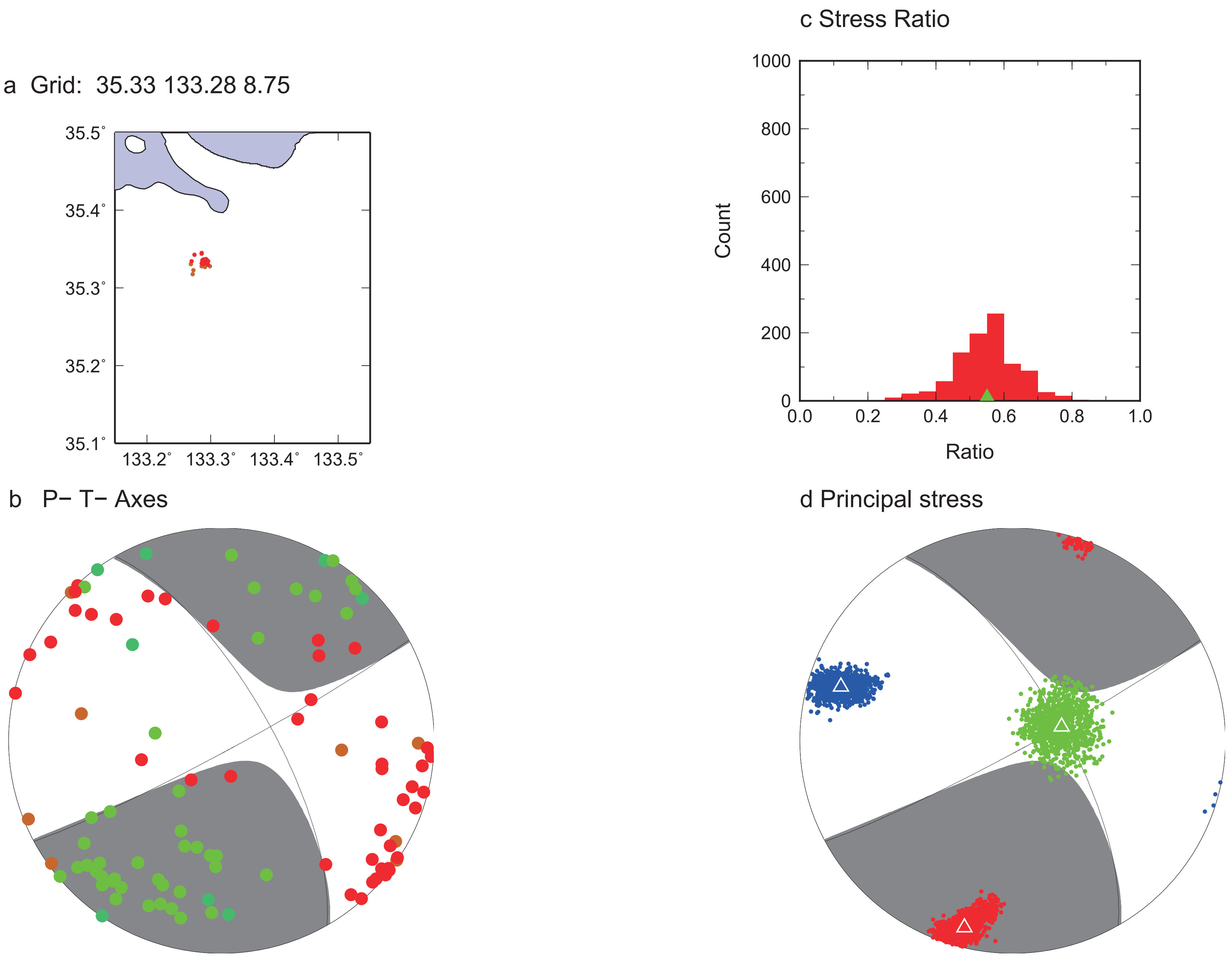
a Grid: 35.33 133.28 8.75
35.5˚
35.4˚
35.3˚
35.2˚
35.1˚
133.2˚
133.3˚
133.4˚
133.5˚
b P− T− Axes
c Stress Ratio
Count
1000
800
600
400
200
0
0.0
0.2
0.4
0.6
0.8
1.0
Ratio
d Principal stress

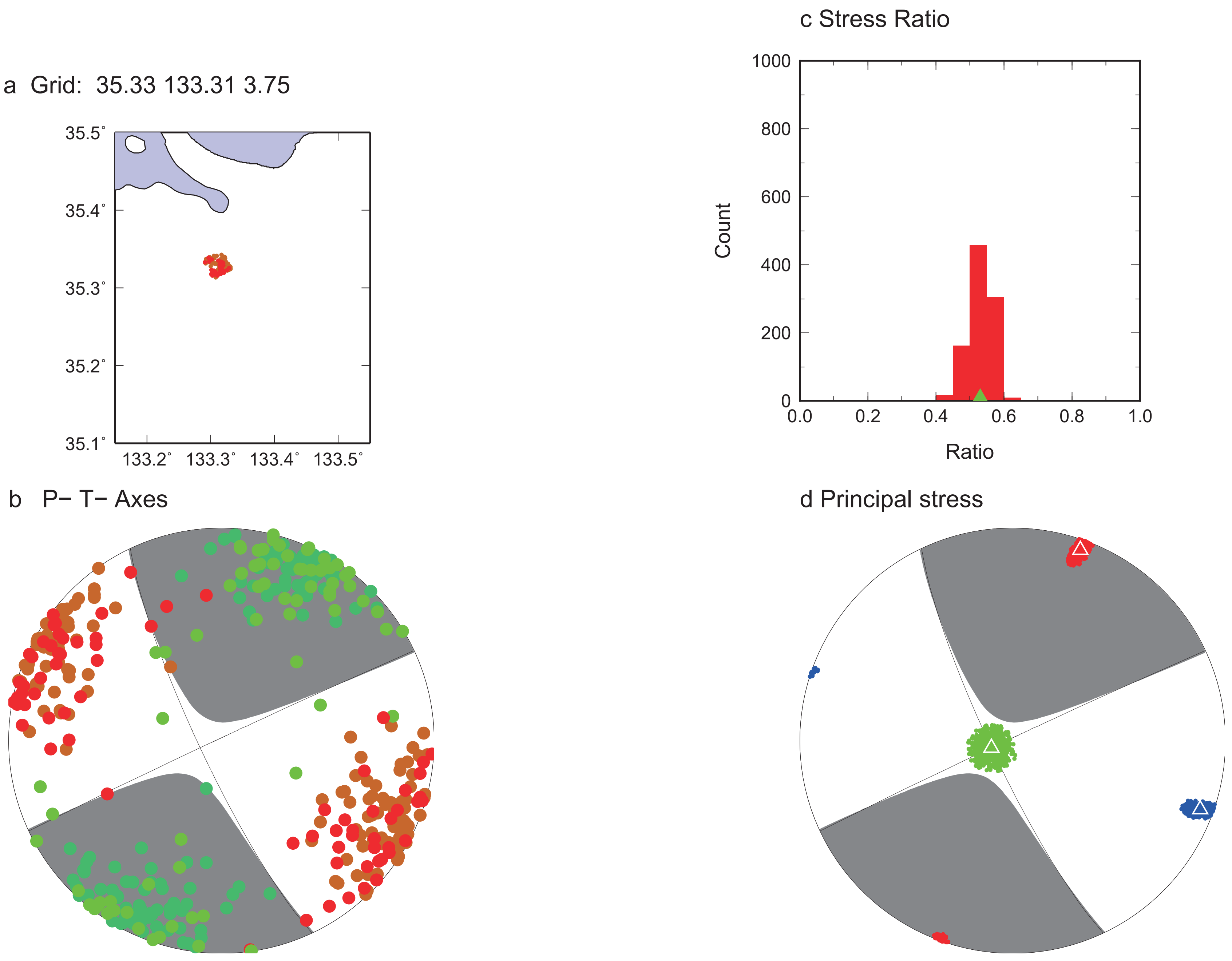
a Grid: 35.33 133.31 3.75
35.5°
35.4°
35.3°
35.2°
35.1°
133.2°
133.3°
133.4°
133.5°
b P− T− Axes
c Stress Ratio
1000
800
600
400
200
0
Count
0.0
0.2
0.4
0.6
0.8
1.0
Ratio
d Principal stress

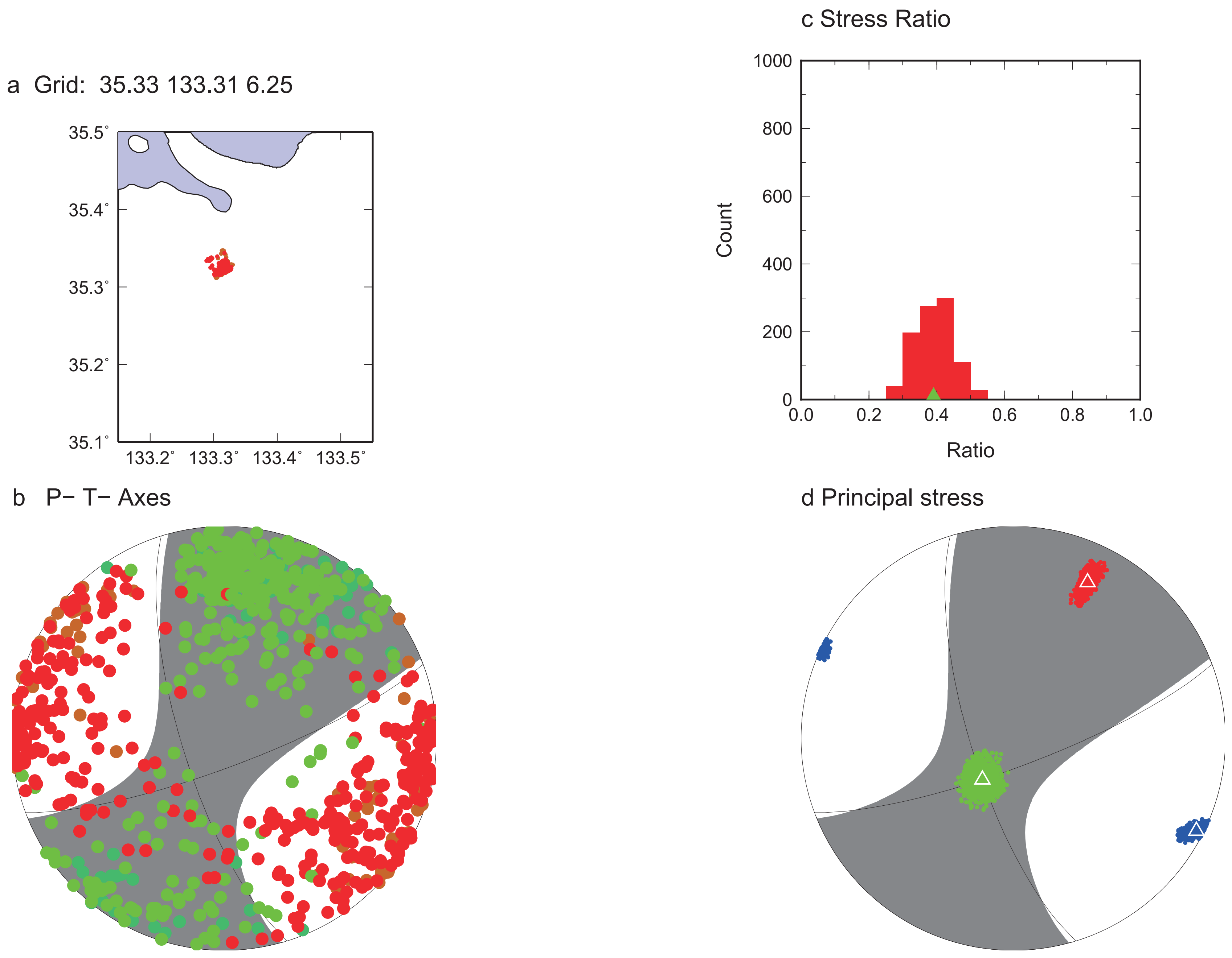
a Grid: 35.33 133.31 6.25
35.5˚
35.4˚
35.3˚
35.2˚
35.1˚
133.2˚
133.3˚
133.4˚
133.5˚
b P− T− Axes
c Stress Ratio
Count
1000
800
600
400
200
0
0.0
0.2
0.4
0.6
0.8
1.0
Ratio
d Principal stress

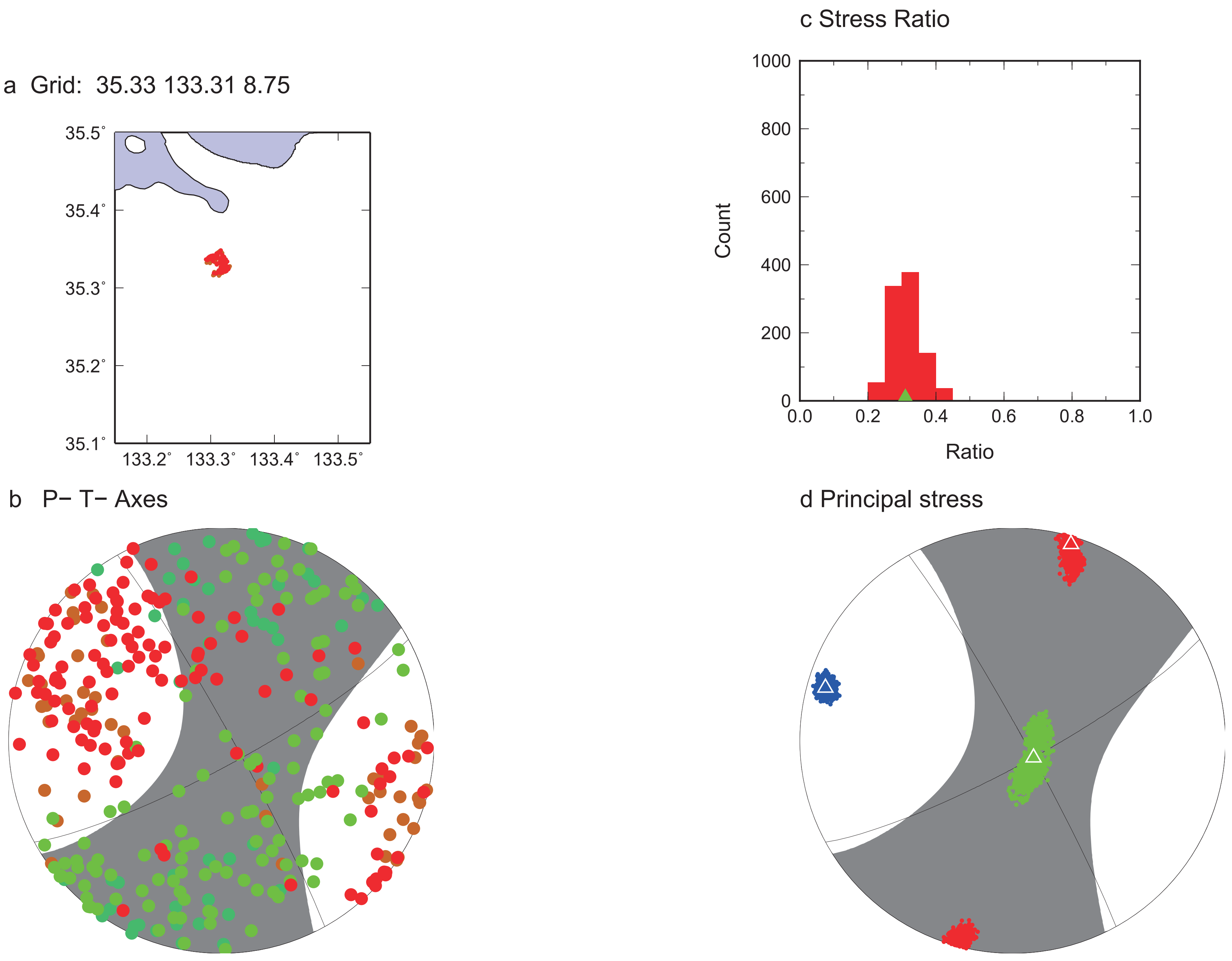
a Grid: 35.33 133.31 8.75
35.5˚
35.4˚
35.3˚
35.2˚
35.1˚
133.2˚
133.3˚
133.4˚
133.5˚
b P– T– Axes
c Stress Ratio
1000
800
600
400
200
0
Count
0.0
0.2
0.4
0.6
0.8
1.0
Ratio
d Principal stress

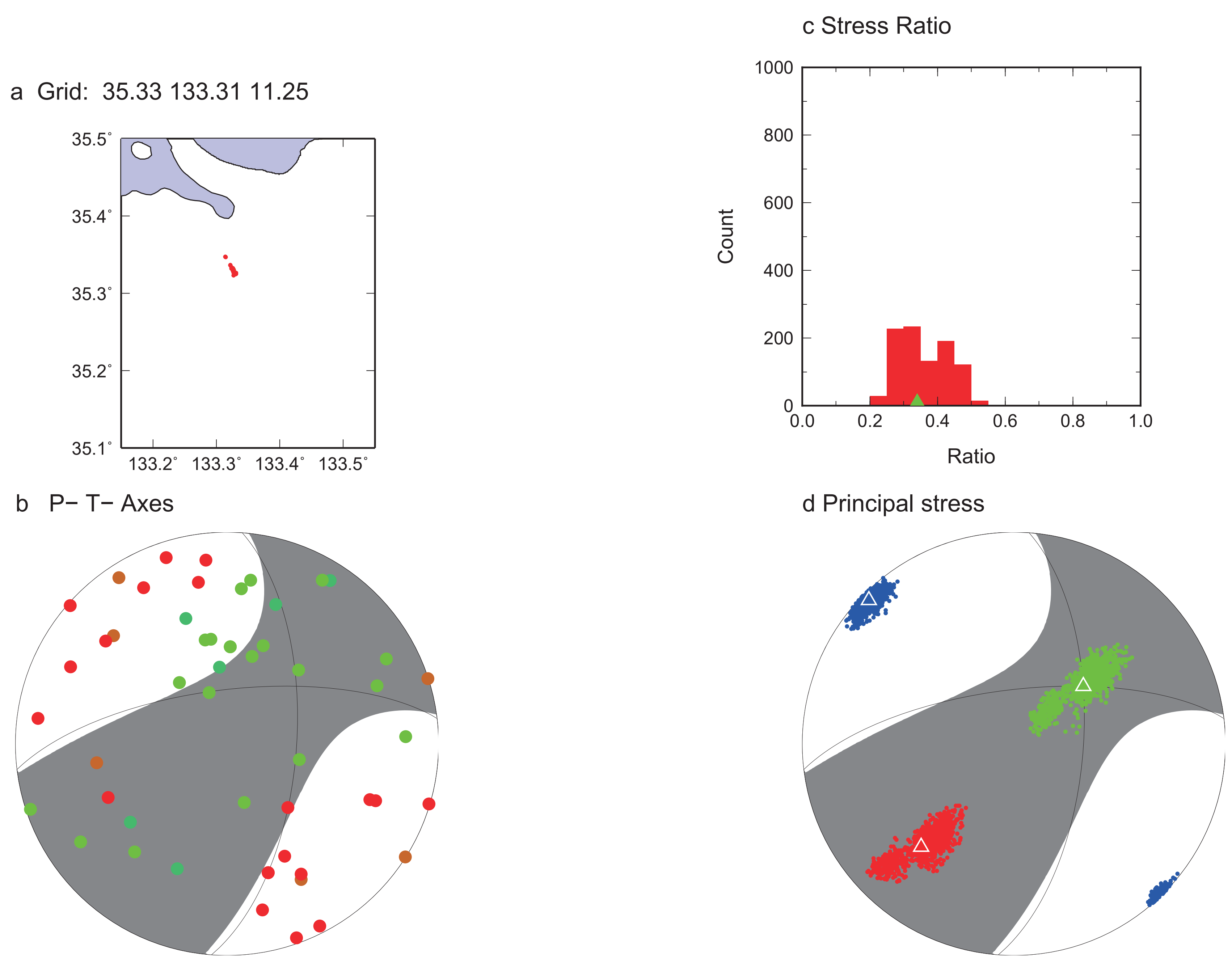
a Grid: 35.33 133.31 11.25
35.5°
35.4°
35.3°
35.2°
35.1°
133.2°
133.3°
133.4°
133.5°
c Stress Ratio
1000
800
600
400
200
0
Count
0.0
0.2
0.4
0.6
0.8
1.0
Ratio
b P− T− Axes
d Principal stress

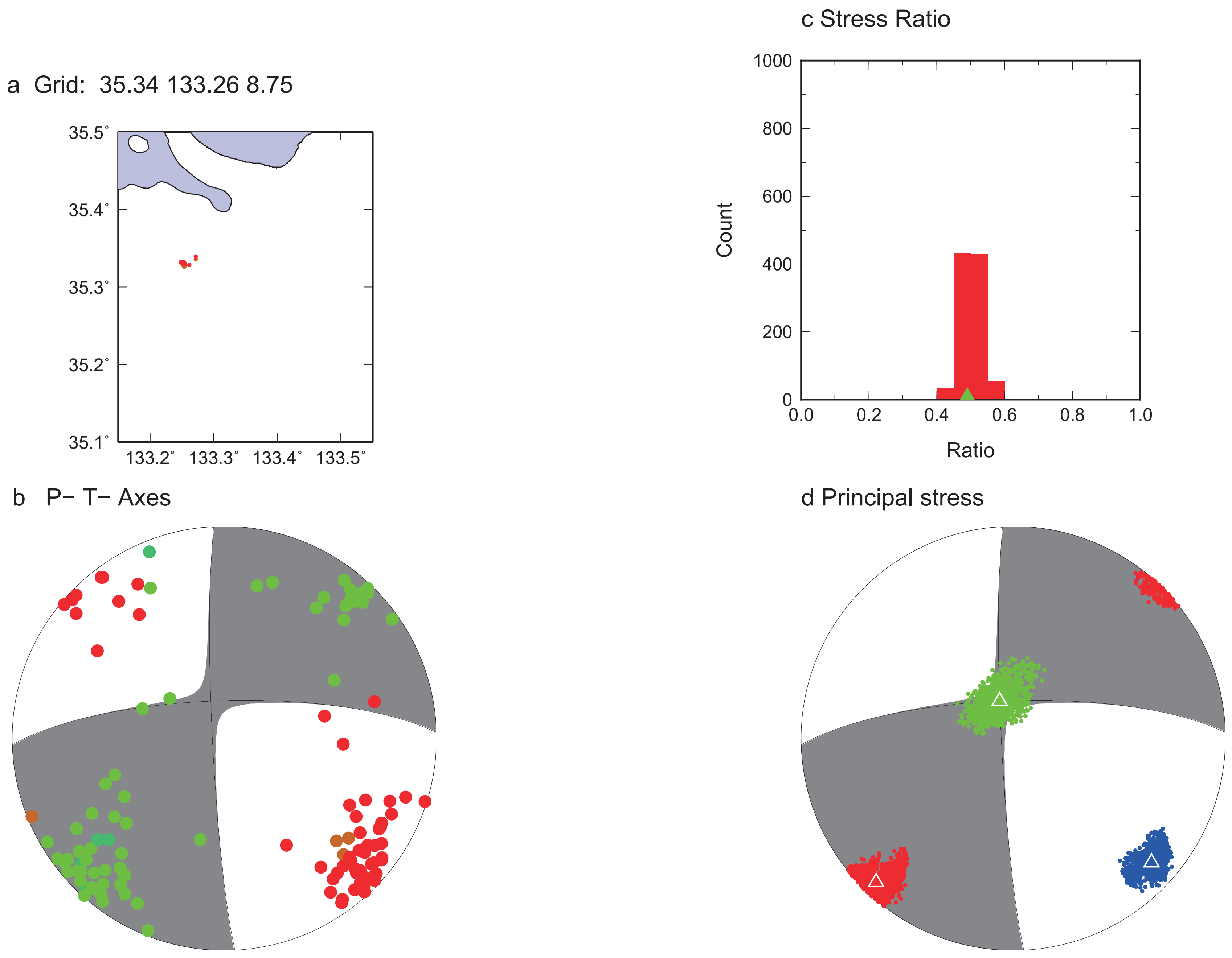

a Grid: 35.34 133.26 8.75
35.5˚
35.4˚
35.3˚
35.2˚
35.1˚
133.2˚
133.3˚
133.4˚
133.5˚
b P− T− Axes
c Stress Ratio
1000
800
600
400
200
0
Count
0.0
0.2
0.4
0.6
0.8
1.0
Ratio
d Principal stress

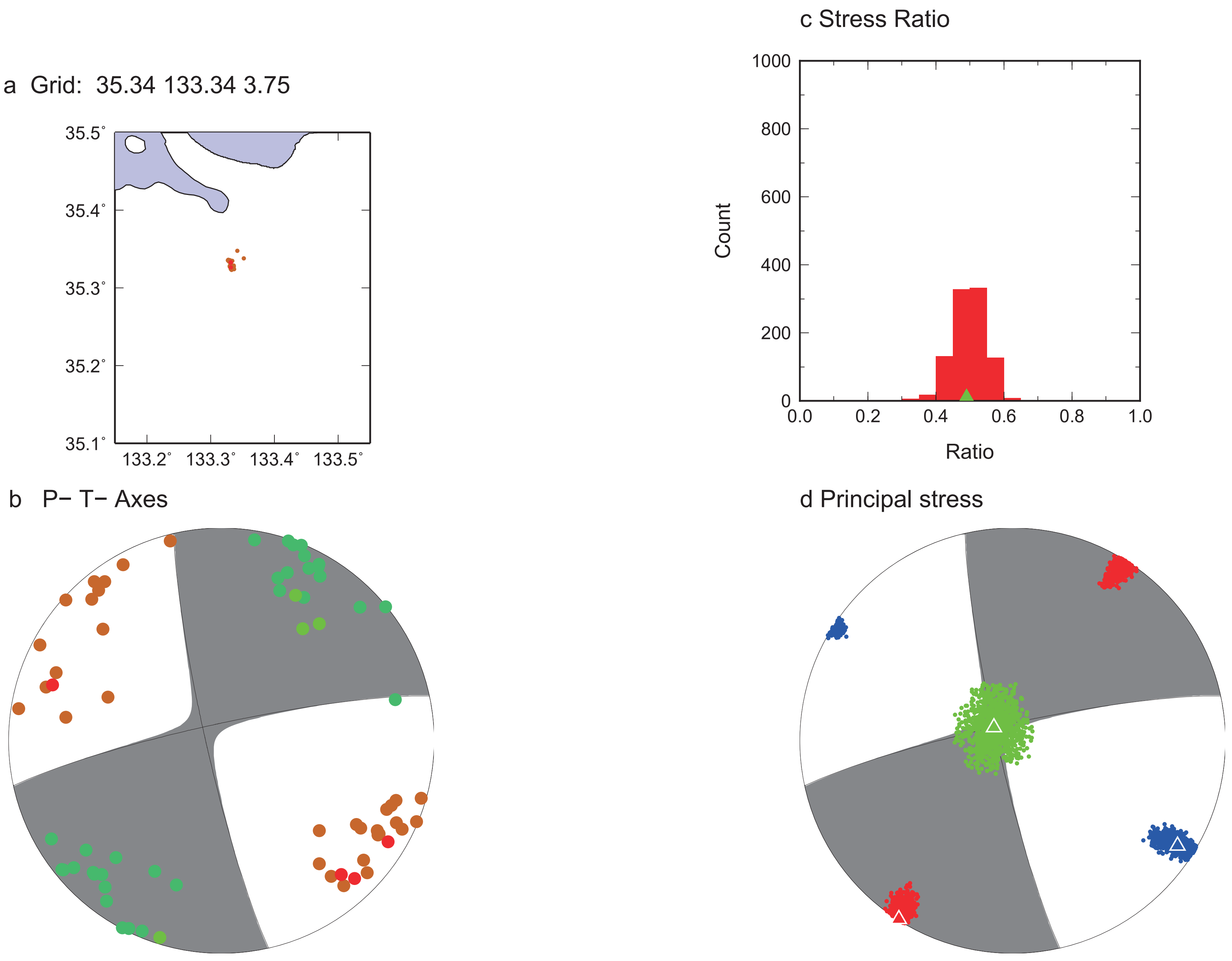
a Grid: 35.34 133.34 3.75
35.5˚
35.4˚
35.3˚
35.2˚
35.1˚
133.2˚ 133.3˚ 133.4˚ 133.5˚
b P− T− Axes
c Stress Ratio
1000
800
600
400
200
0
Count
0.0 0.2 0.4 0.6 0.8 1.0
Ratio
d Principal stress

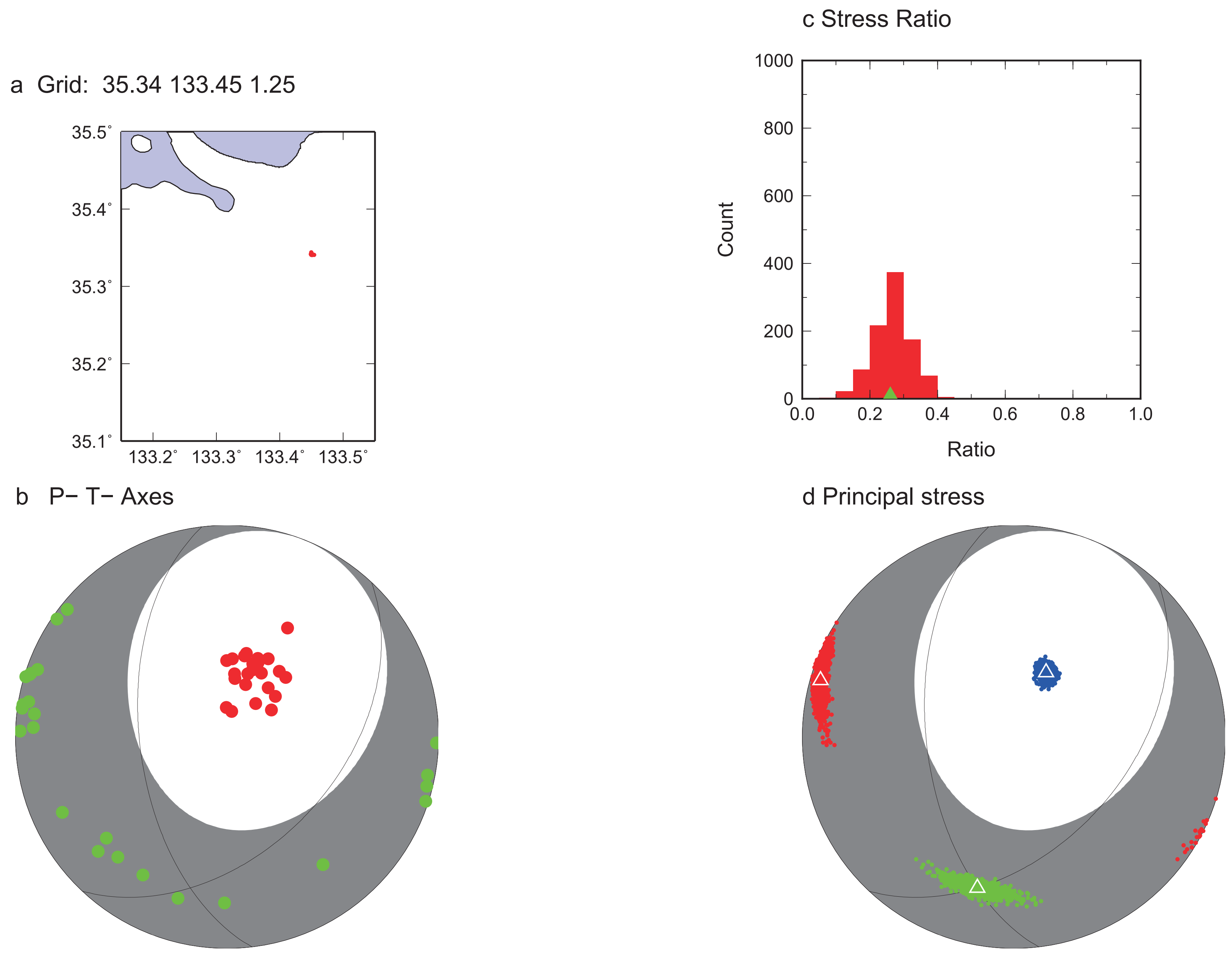
a Grid: 35.34 133.45 1.25
35.5°
35.4°
35.3°
35.2°
35.1°
133.2°
133.3°
133.4°
133.5°
c Stress Ratio
Count
1000
800
600
400
200
0
0.0
0.2
0.4
0.6
0.8
1.0
Ratio
b P– T– Axes
d Principal stress

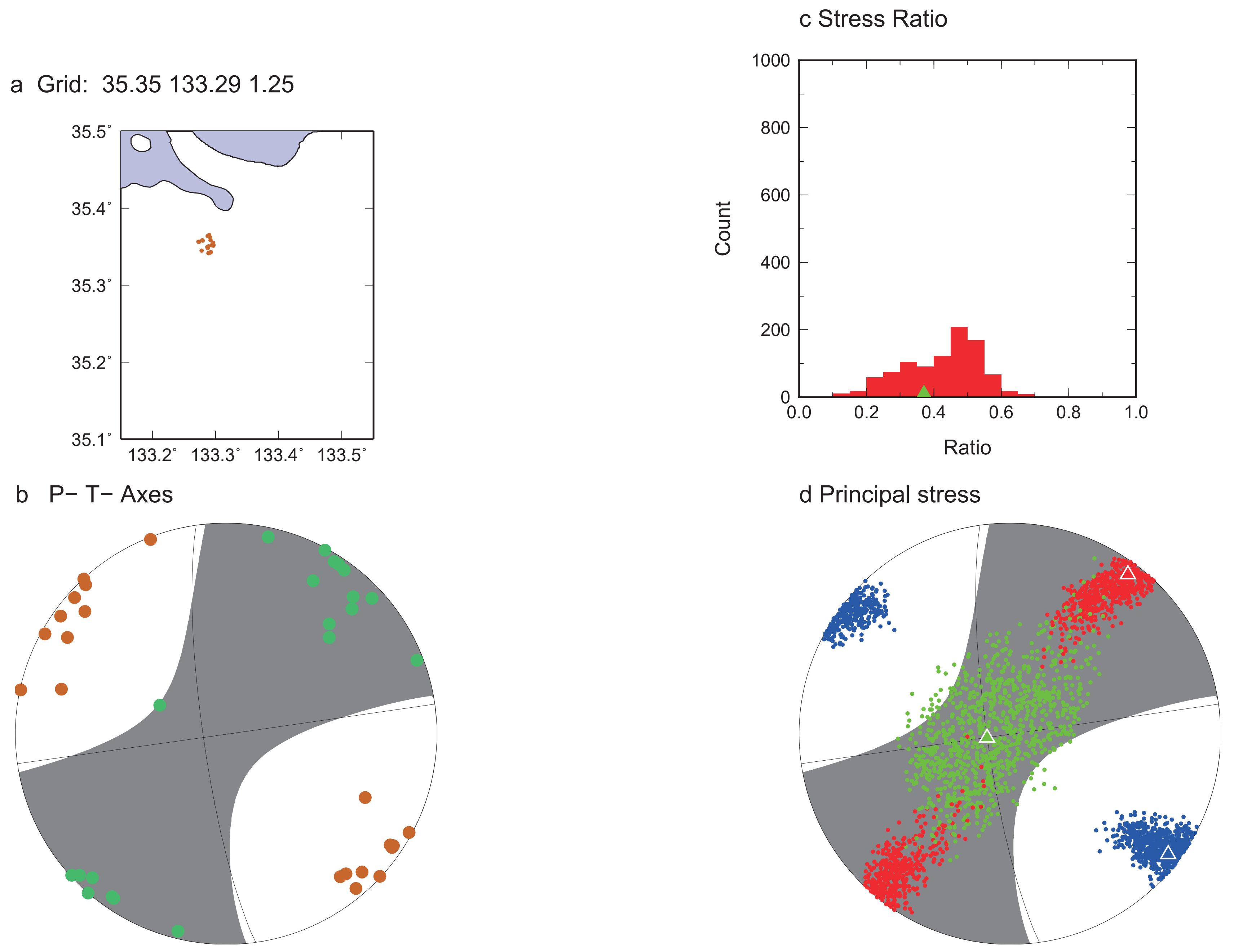

a Grid: 35.35 133.29 1.25
35.5°
35.4°
35.3°
35.2°
35.1°
133.2°
133.3°
133.4°
133.5°
b P− T− Axes
c Stress Ratio
Count
1000
800
600
400
200
0
0.0
0.2
0.4
0.6
0.8
1.0
Ratio
d Principal stress

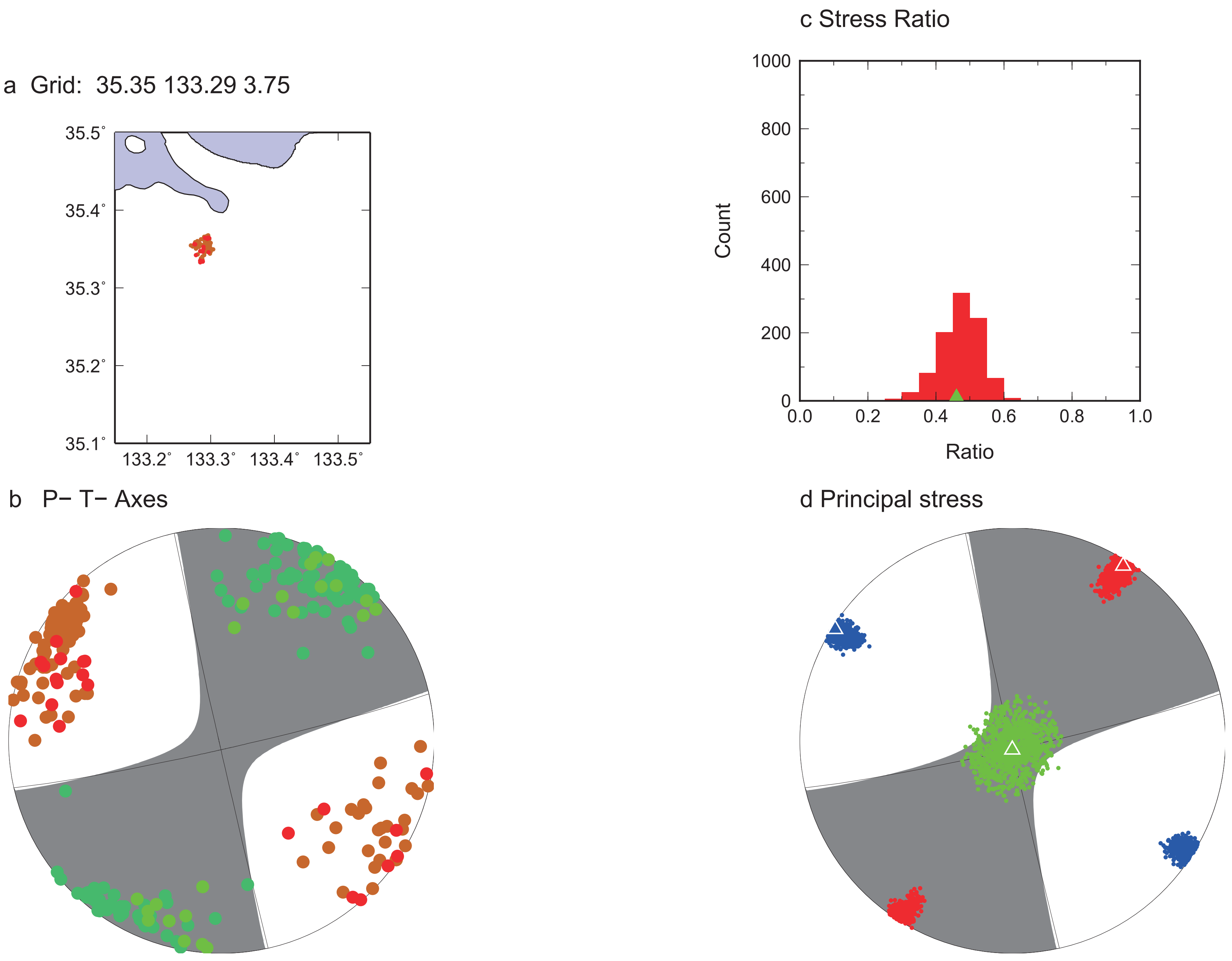

a Grid: 35.35 133.29 3.75
35.5˚
35.4˚
35.3˚
35.2˚
35.1˚
133.2˚
133.3˚
133.4˚
133.5˚
b P– T– Axes
c Stress Ratio
Count
1000
800
600
400
200
0
0.0
0.2
0.4
0.6
0.8
1.0
Ratio
d Principal stress

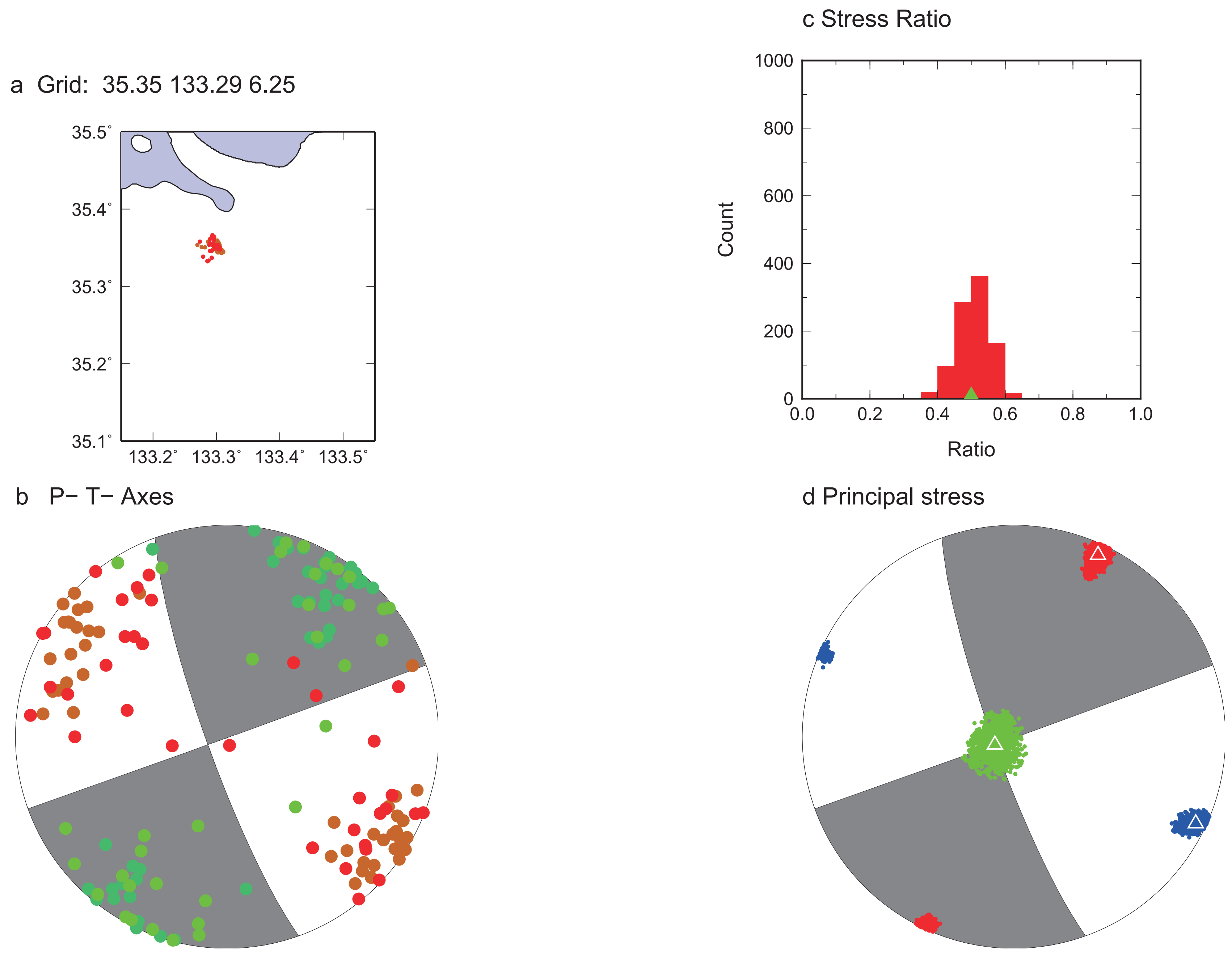

a Grid: 35.35 133.29 6.25
35.5˚
35.4˚
35.3˚
35.2˚
35.1˚
133.2˚
133.3˚
133.4˚
133.5˚
b P− T− Axes
c Stress Ratio
1000
800
600
400
200
0
Count
0.0
0.2
0.4
0.6
0.8
1.0
Ratio
d Principal stress

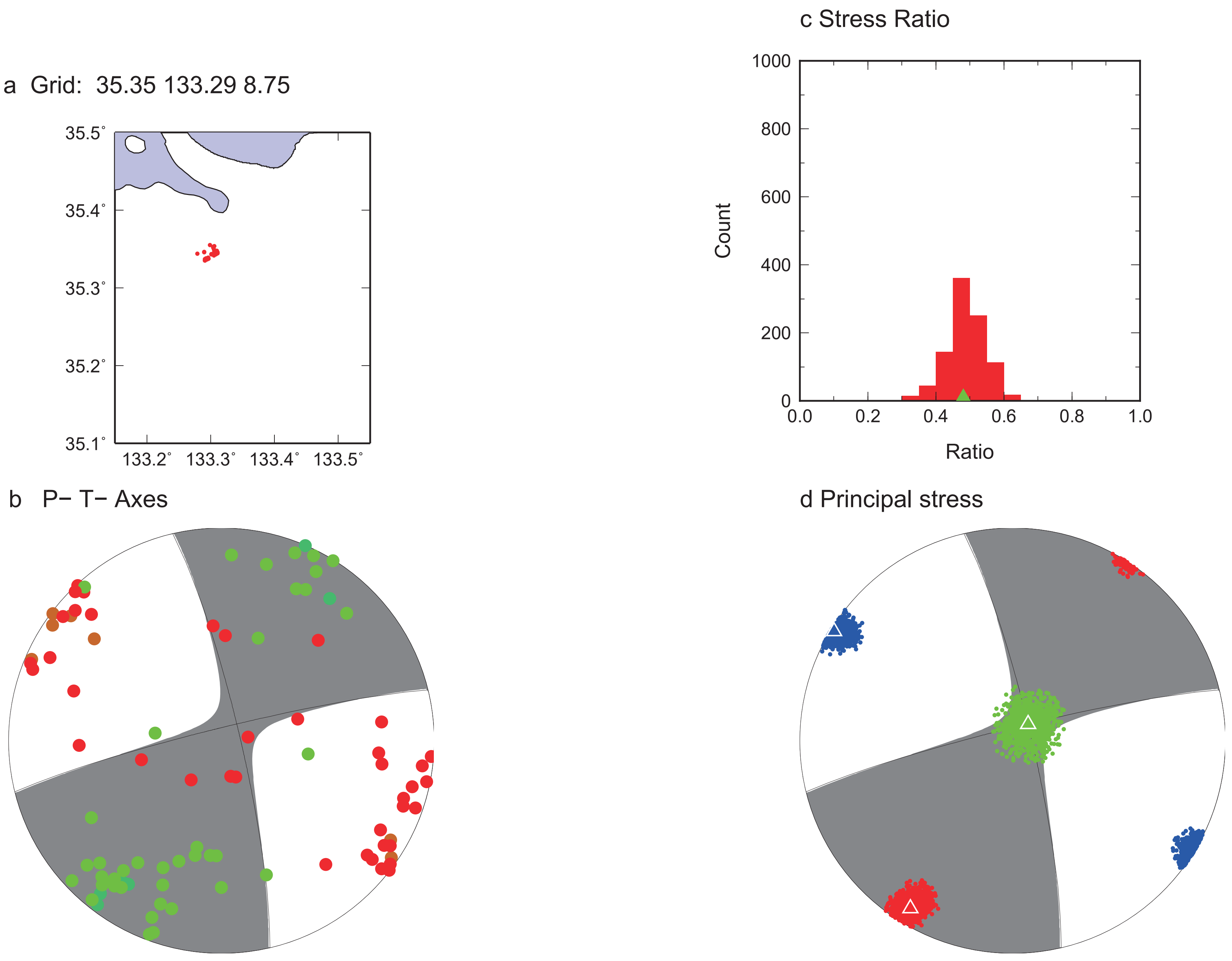
a Grid: 35.35 133.29 8.75
35.5˚
35.4˚
35.3˚
35.2˚
35.1˚
133.2˚
133.3˚
133.4˚
133.5˚
b P− T− Axes
c Stress Ratio
1000
800
600
400
200
0
Count
0.0
0.2
0.4
0.6
0.8
1.0
Ratio
d Principal stress

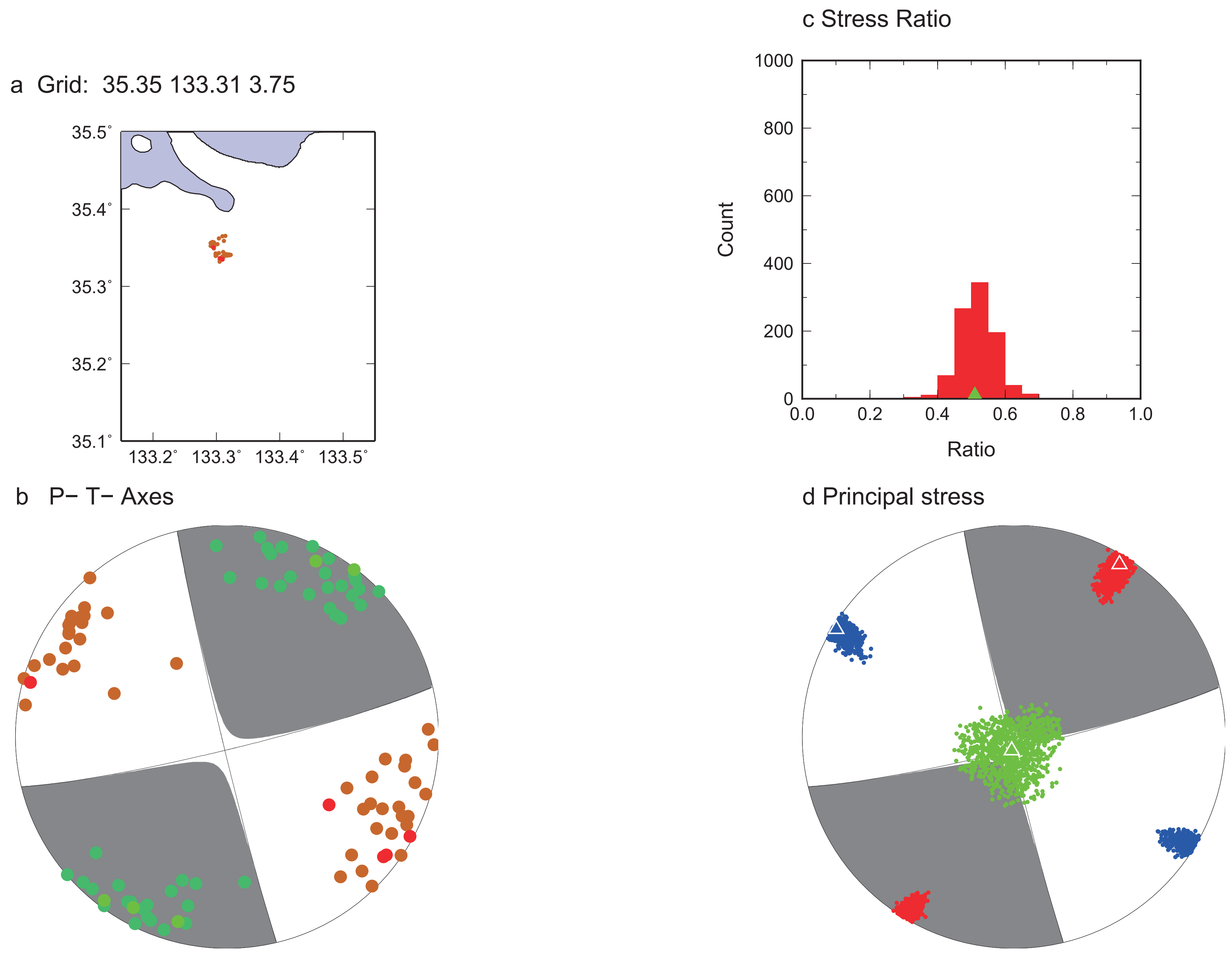
a Grid: 35.35 133.31 3.75
35.5˚
35.4˚
35.3˚
35.2˚
35.1˚
133.2˚ 133.3˚ 133.4˚ 133.5˚
c Stress Ratio
Count
1000
800
600
400
200
0
0.0 0.2 0.4 0.6 0.8 1.0
Ratio
b P− T− Axes
d Principal stress

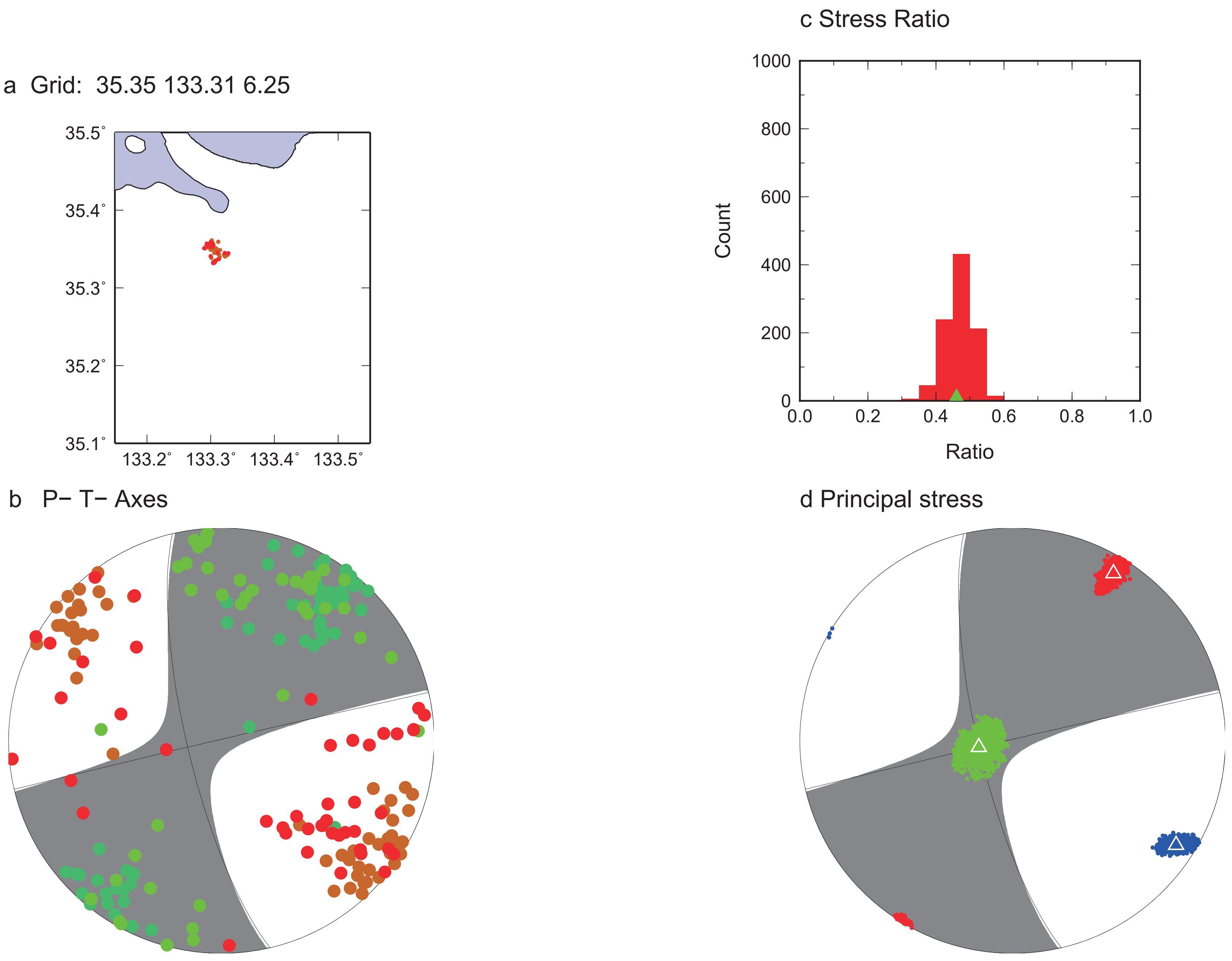

a Grid: 35.35 133.31 6.25
35.5˚
35.4˚
35.3˚
35.2˚
35.1˚
133.2˚ 133.3˚ 133.4˚ 133.5˚
b P− T− Axes
c Stress Ratio
Count
1000
800
600
400
200
0
0.0 0.2 0.4 0.6 0.8 1.0
Ratio
d Principal stress

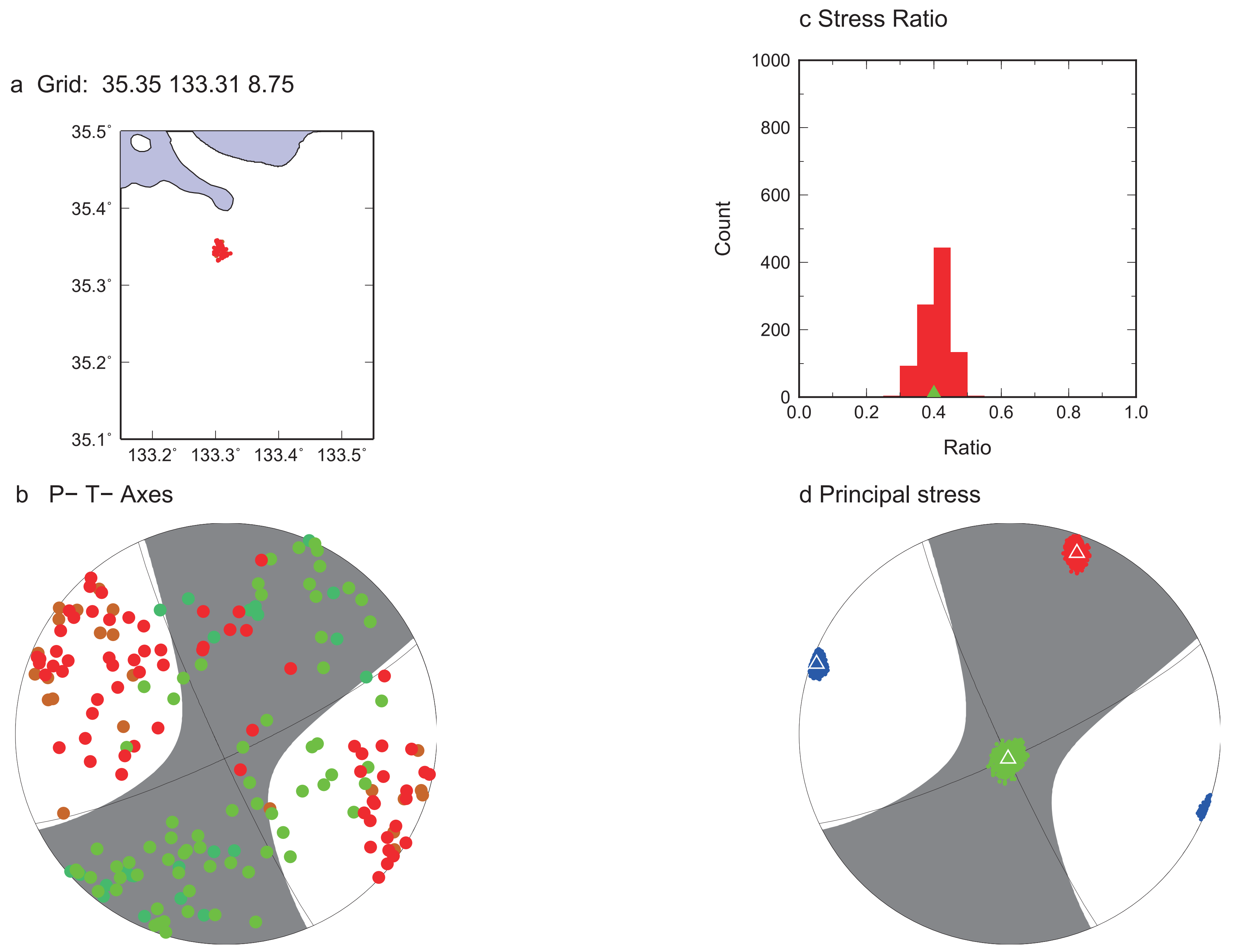
a Grid: 35.35 133.31 8.75
35.5˚
35.4˚
35.3˚
35.2˚
35.1˚
133.2˚ 133.3˚ 133.4˚ 133.5˚
b P− T− Axes
c Stress Ratio
1000
800
600
400
200
0
Count
0.0 0.2 0.4 0.6 0.8 1.0
Ratio
d Principal stress

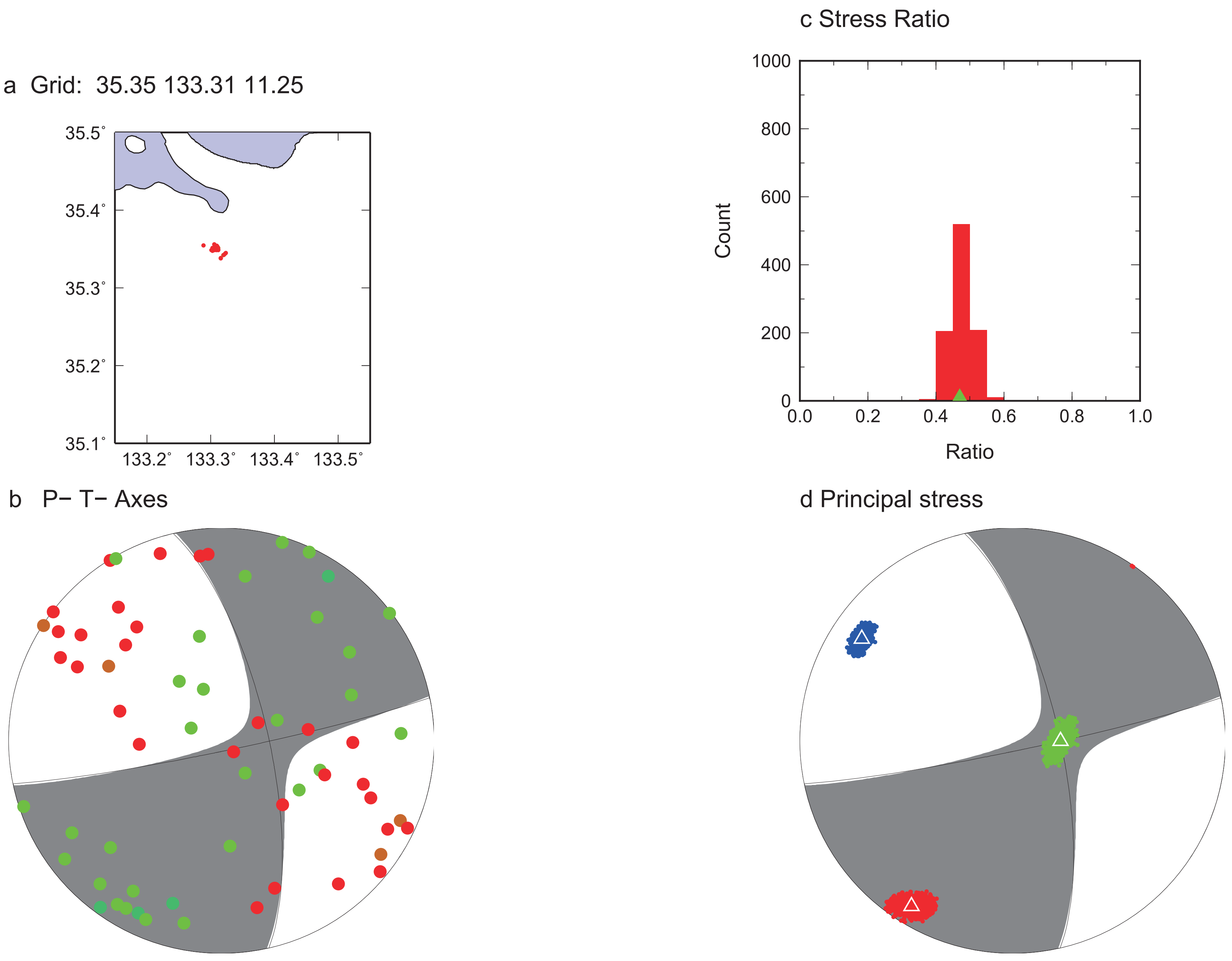
a Grid: 35.35 133.31 11.25
35.5˚
35.4˚
35.3˚
35.2˚
35.1˚
133.2˚ 133.3˚ 133.4˚ 133.5˚
b P− T− Axes
c Stress Ratio
Count
1000
800
600
400
200
0
0.0 0.2 0.4 0.6 0.8 1.0
Ratio
d Principal stress

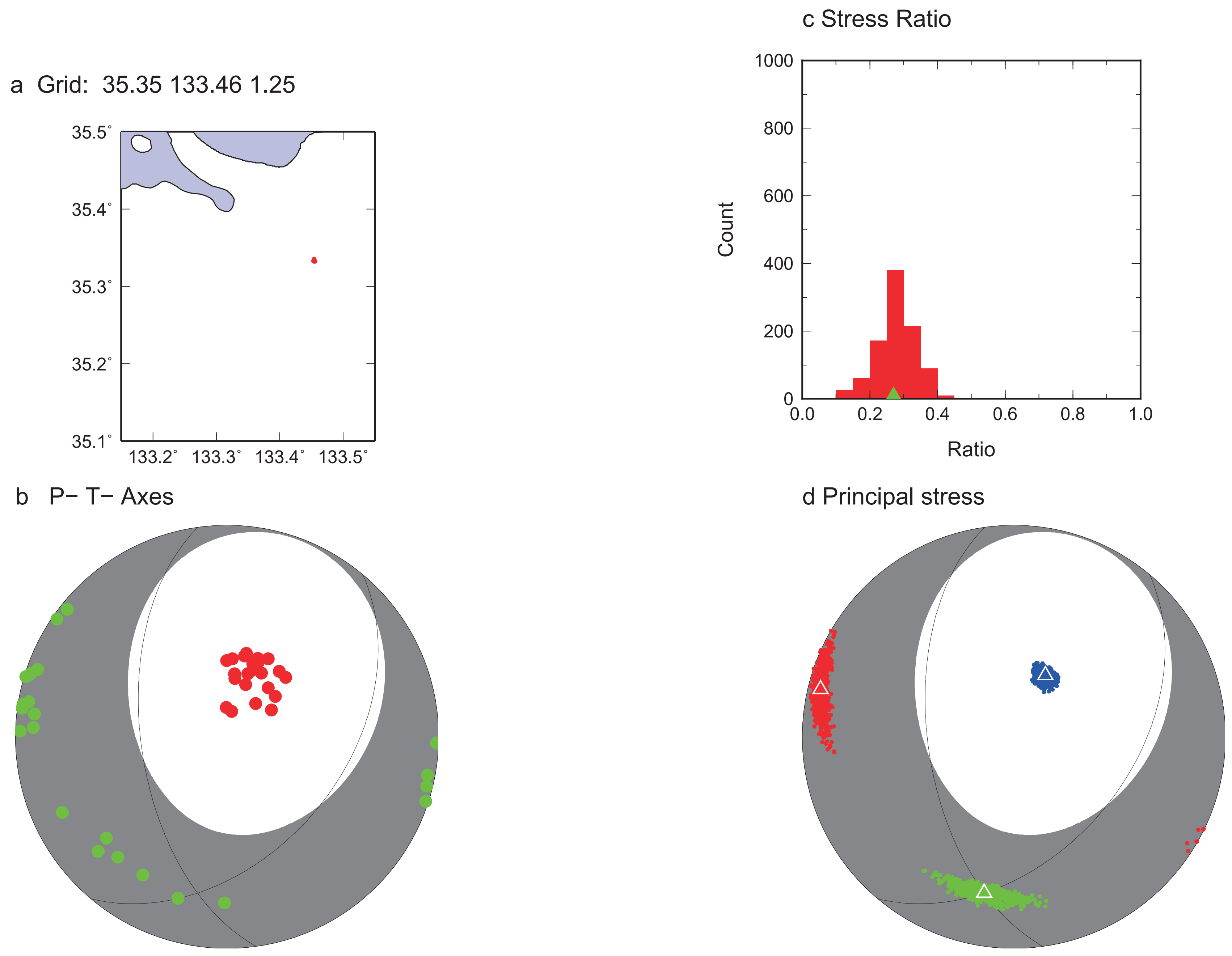
a Grid: 35.35 133.46 1.25
35.5°
35.4°
35.3°
35.2°
35.1°
133.2°
133.3°
133.4°
133.5°
b P− T− Axes
c Stress Ratio
Count
1000
800
600
400
200
0
0.0
0.2
0.4
0.6
0.8
1.0
Ratio
d Principal stress

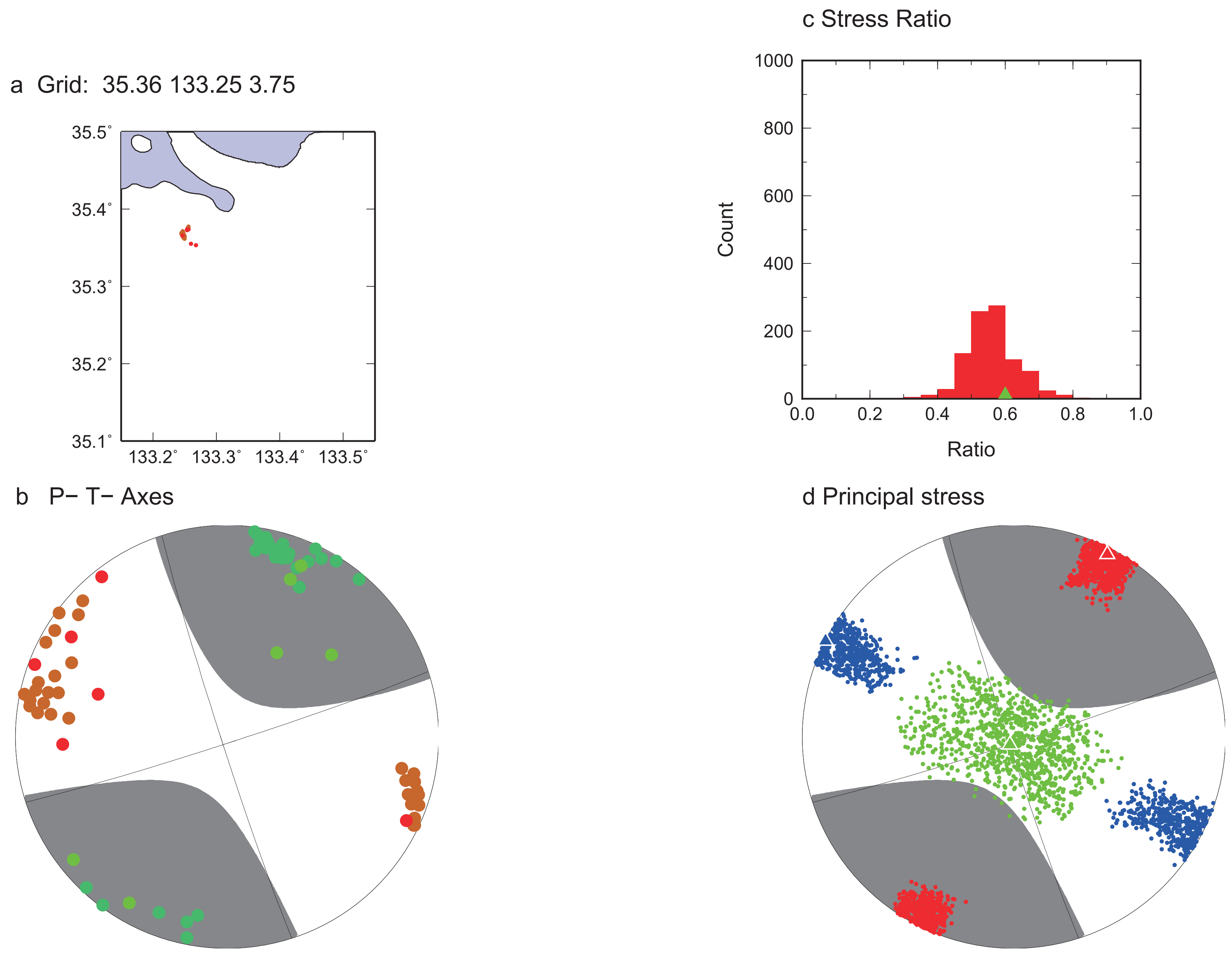
a Grid: 35.36 133.25 3.75
35.5˚
35.4˚
35.3˚
35.2˚
35.1˚
133.2˚ 133.3˚ 133.4˚ 133.5˚
b P− T− Axes
c Stress Ratio
Count
1000
800
600
400
200
0
0.0 0.2 0.4 0.6 0.8 1.0
Ratio
d Principal stress

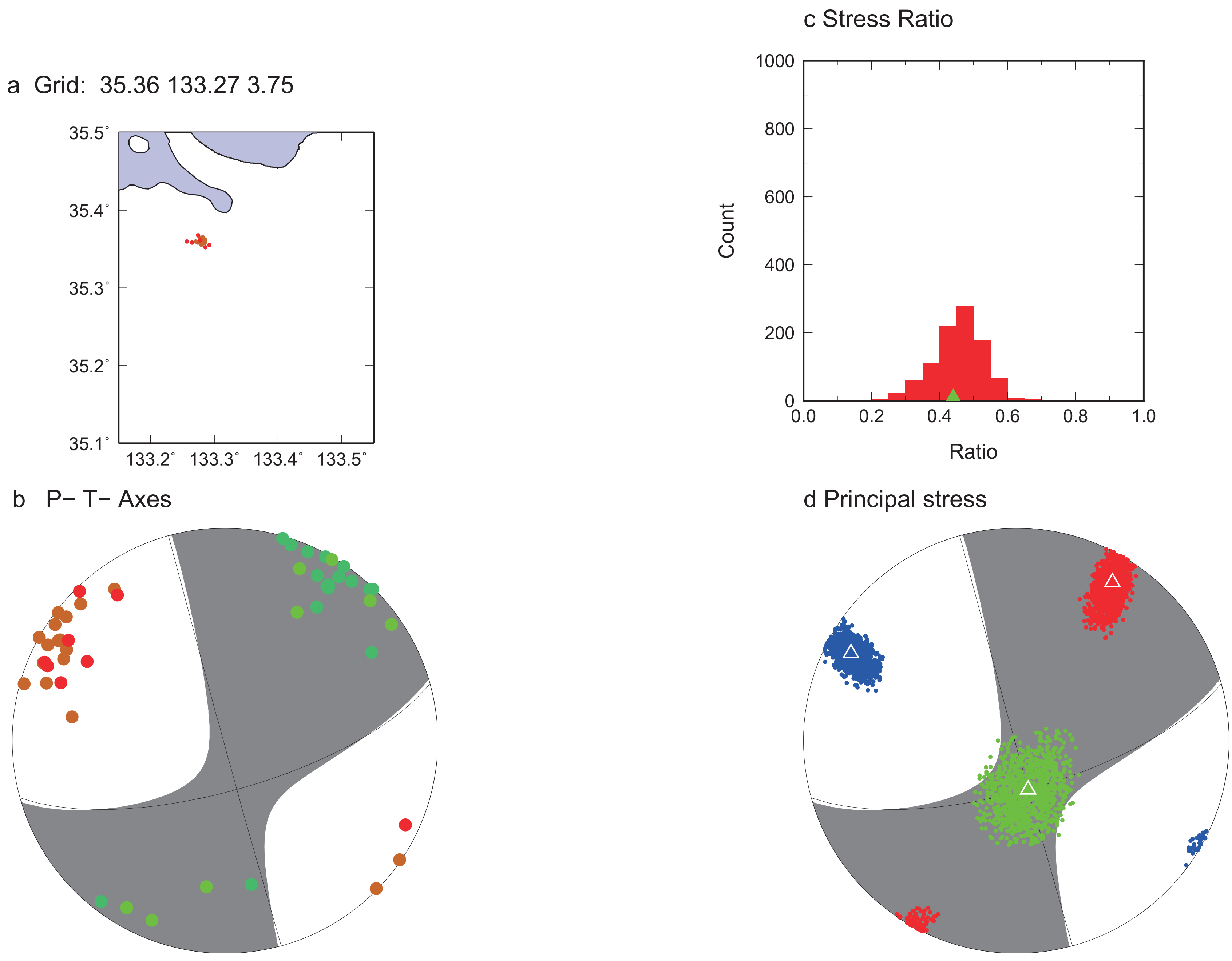

a Grid: 35.36 133.27 3.75
35.5˚
35.4˚
35.3˚
35.2˚
35.1˚
133.2˚
133.3˚
133.4˚
133.5˚
c Stress Ratio
Count
1000
800
600
400
200
0
0.0
0.2
0.4
0.6
0.8
1.0
Ratio
b P− T− Axes
d Principal stress

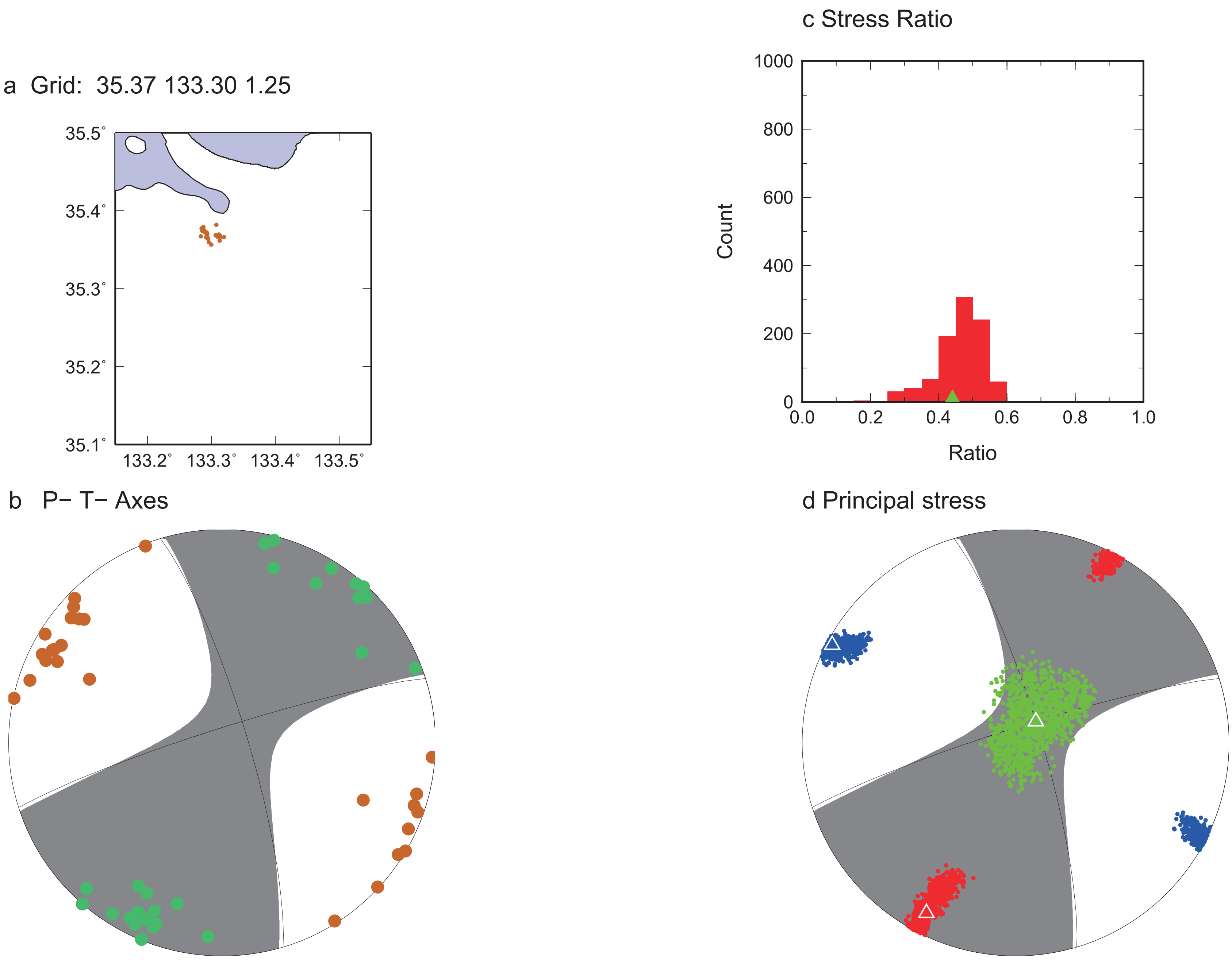
a Grid: 35.37 133.30 1.25
35.5˚
35.4˚
35.3˚
35.2˚
35.1˚
133.2˚ 133.3˚ 133.4˚ 133.5˚
b P− T− Axes
c Stress Ratio
Count
1000
800
600
400
200
0
0.0 0.2 0.4 0.6 0.8 1.0
Ratio
d Principal stress

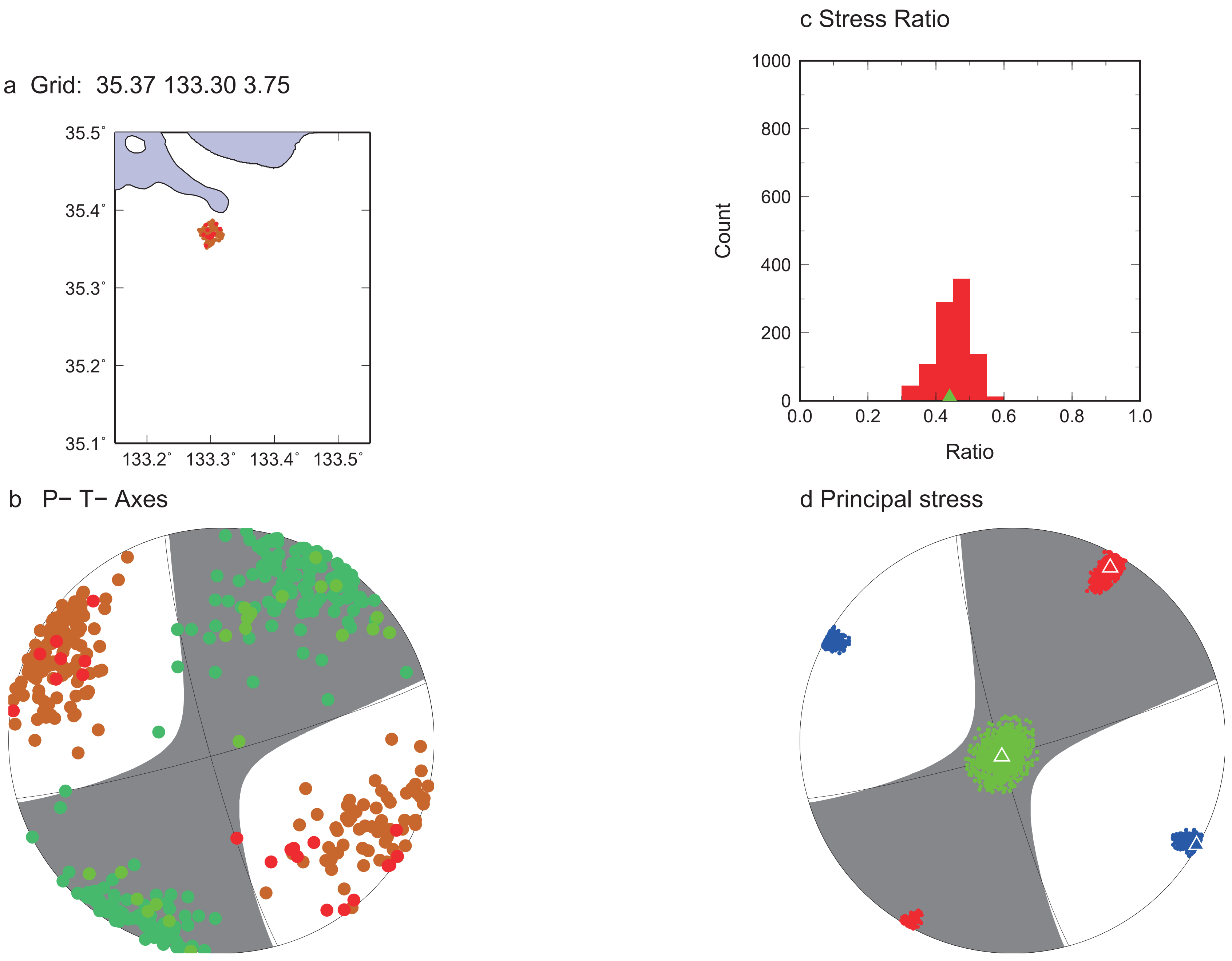

a Grid: 35.37 133.30 3.75
35.5˚
35.4˚
35.3˚
35.2˚
35.1˚
133.2˚
133.3˚
133.4˚
133.5˚
b P– T– Axes
c Stress Ratio
1000
800
600
400
200
0
Count
0.0
0.2
0.4
0.6
0.8
1.0
Ratio
d Principal stress

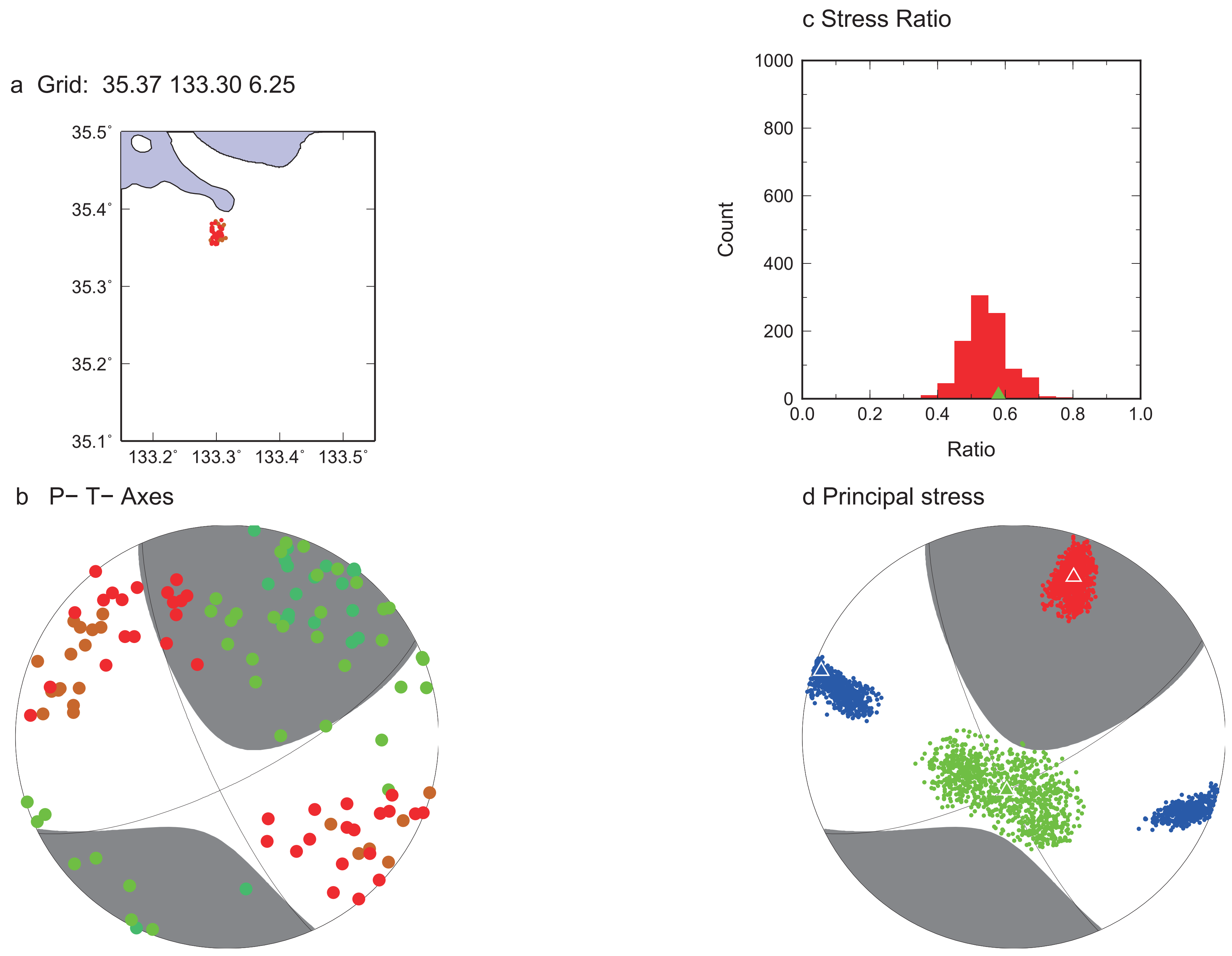

a Grid: 35.37 133.30 6.25
35.5˚
35.4˚
35.3˚
35.2˚
35.1˚
133.2˚
133.3˚
133.4˚
133.5˚
b P– T– Axes
c Stress Ratio
Count
1000
800
600
400
200
0
0.0
0.2
0.4
0.6
0.8
1.0
Ratio
d Principal stress

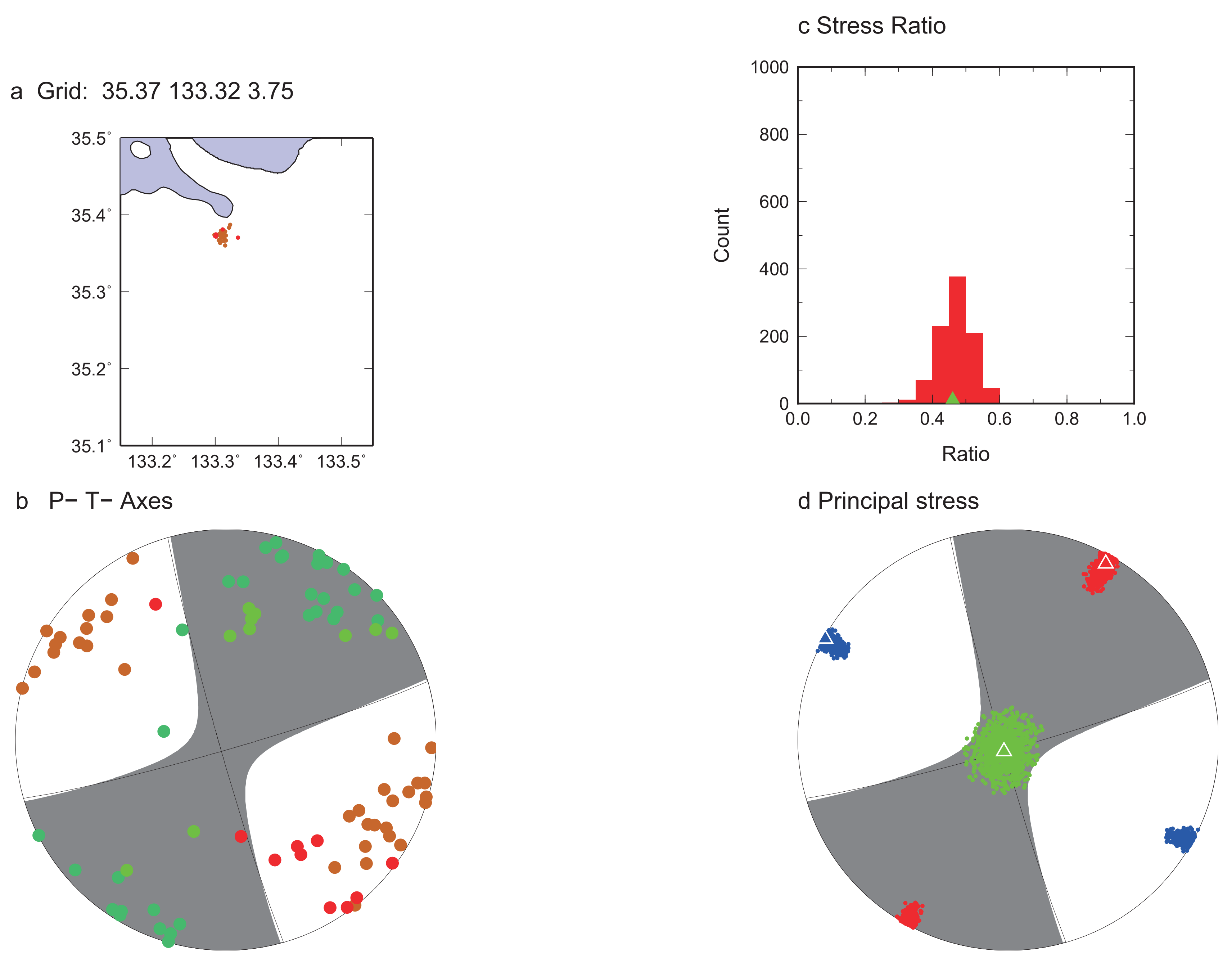
a Grid: 35.37 133.32 3.75
35.5˚
35.4˚
35.3˚
35.2˚
35.1˚
133.2˚ 133.3˚ 133.4˚ 133.5˚
b P− T− Axes
c Stress Ratio
1000
800
600
400
200
0
Count
0.0 0.2 0.4 0.6 0.8 1.0
Ratio
d Principal stress

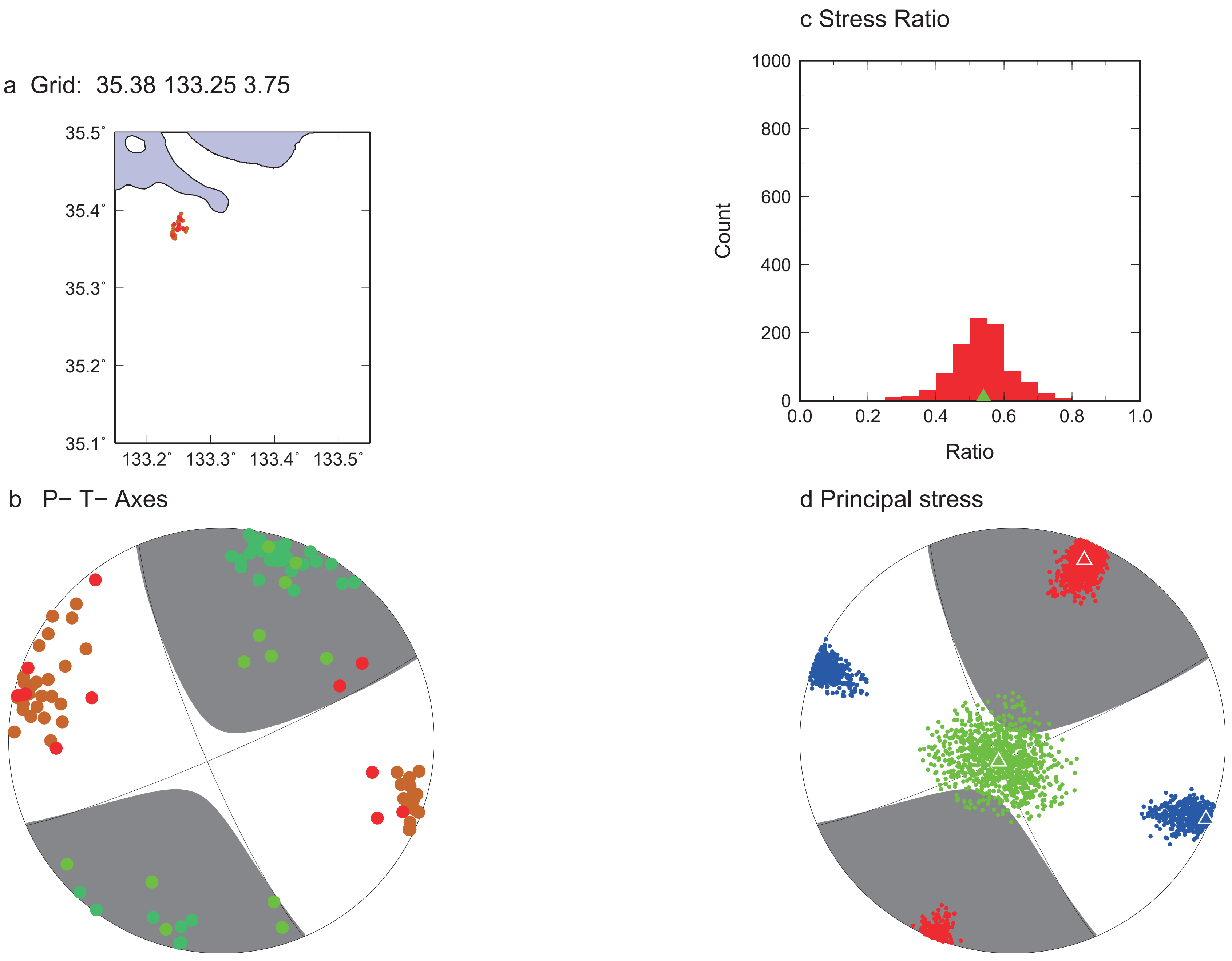
a Grid: 35.38 133.25 3.75
35.5˚
35.4˚
35.3˚
35.2˚
35.1˚
133.2˚ 133.3˚ 133.4˚ 133.5˚
b P− T− Axes
c Stress Ratio
Count
1000
800
600
400
200
0
0.0 0.2 0.4 0.6 0.8 1.0
Ratio
d Principal stress

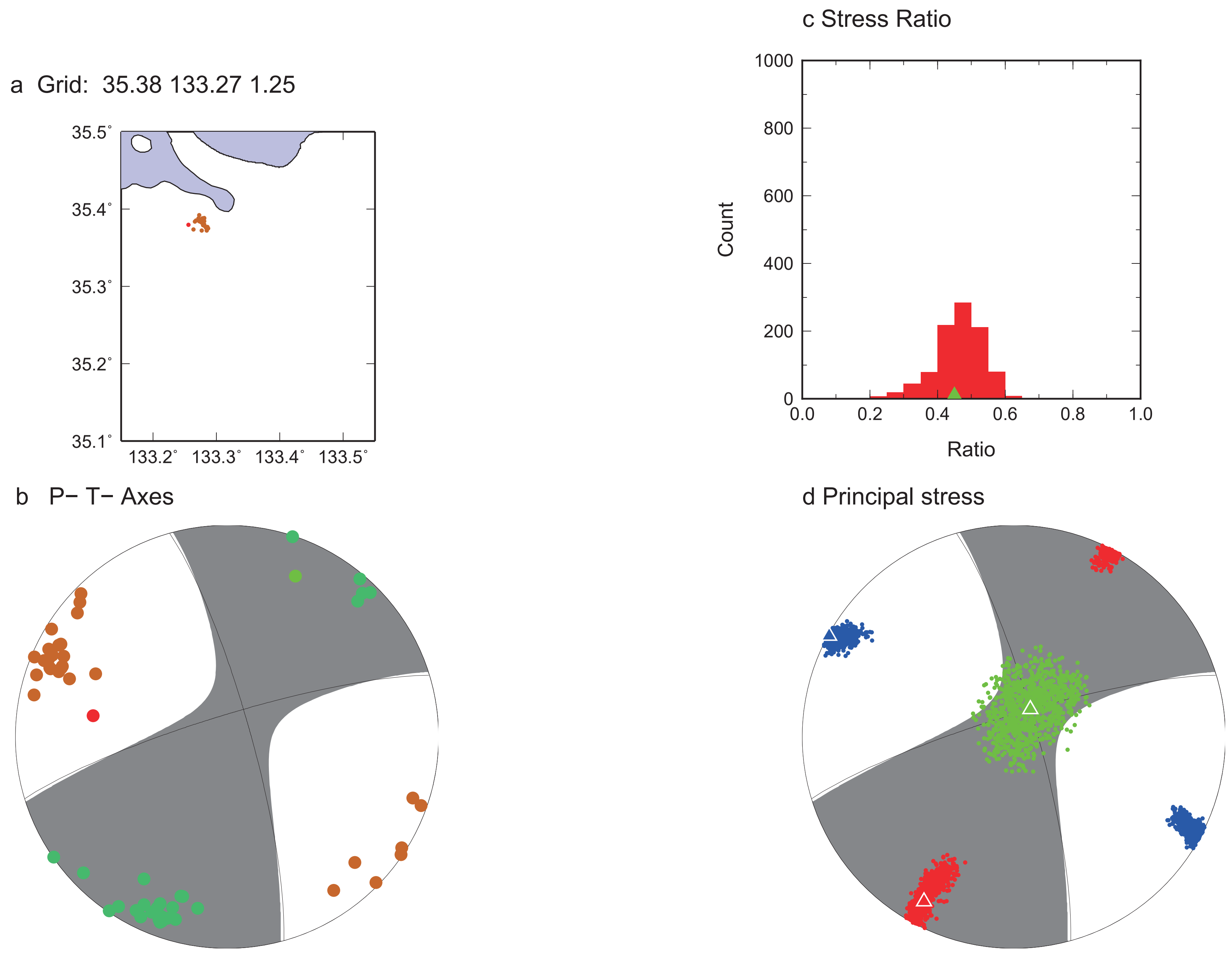
a Grid: 35.38 133.27 1.25
35.5°
35.4°
35.3°
35.2°
35.1°
133.2°
133.3°
133.4°
133.5°
b P– T– Axes
c Stress Ratio
Count
1000
800
600
400
200
0
0.0
0.2
0.4
0.6
0.8
1.0
Ratio
d Principal stress

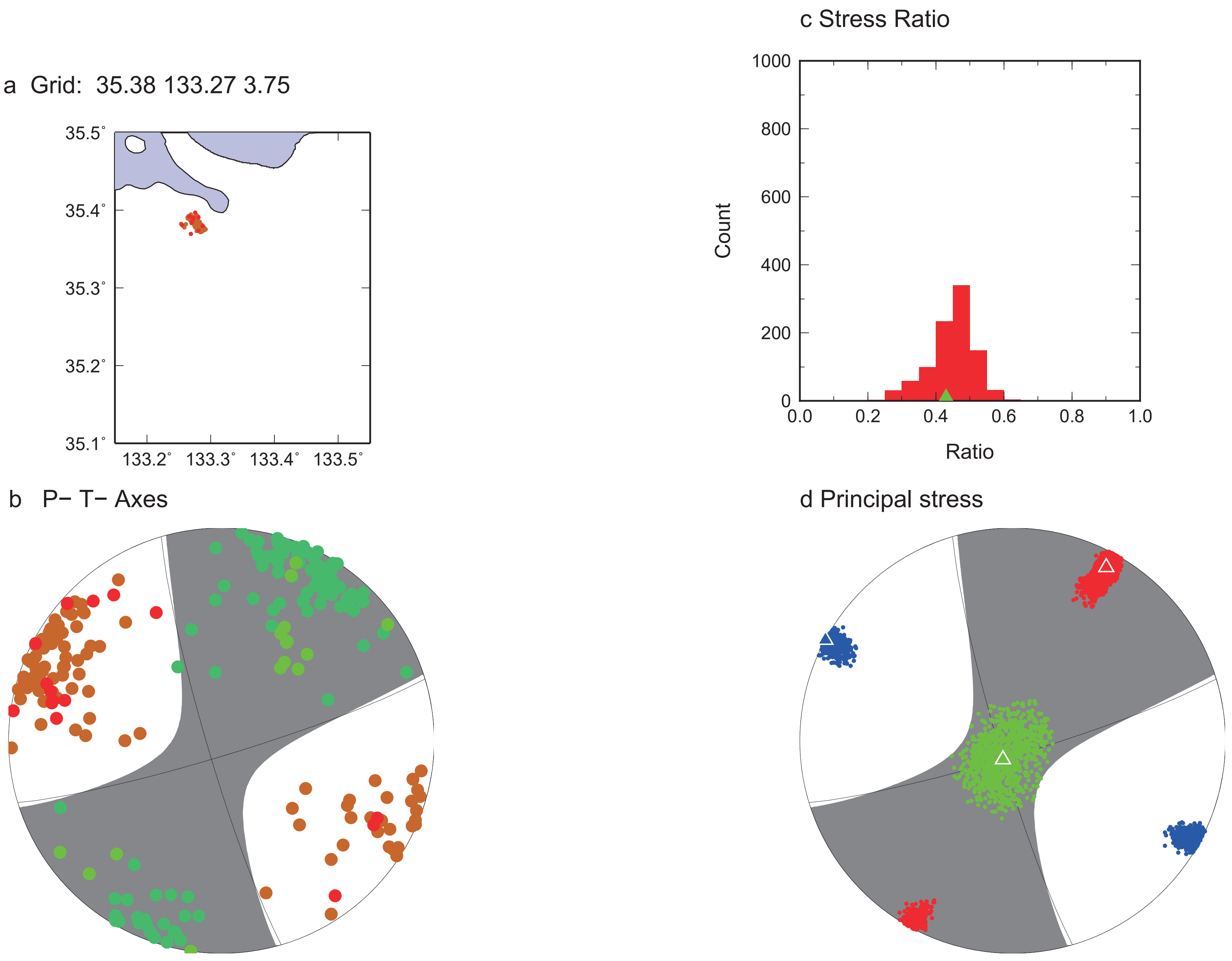
a Grid: 35.38 133.27 3.75
35.5˚
35.4˚
35.3˚
35.2˚
35.1˚
133.2˚
133.3˚
133.4˚
133.5˚
b P− T− Axes
c Stress Ratio
1000
800
600
400
200
0
Count
0.0
0.2
0.4
0.6
0.8
1.0
Ratio
d Principal stress

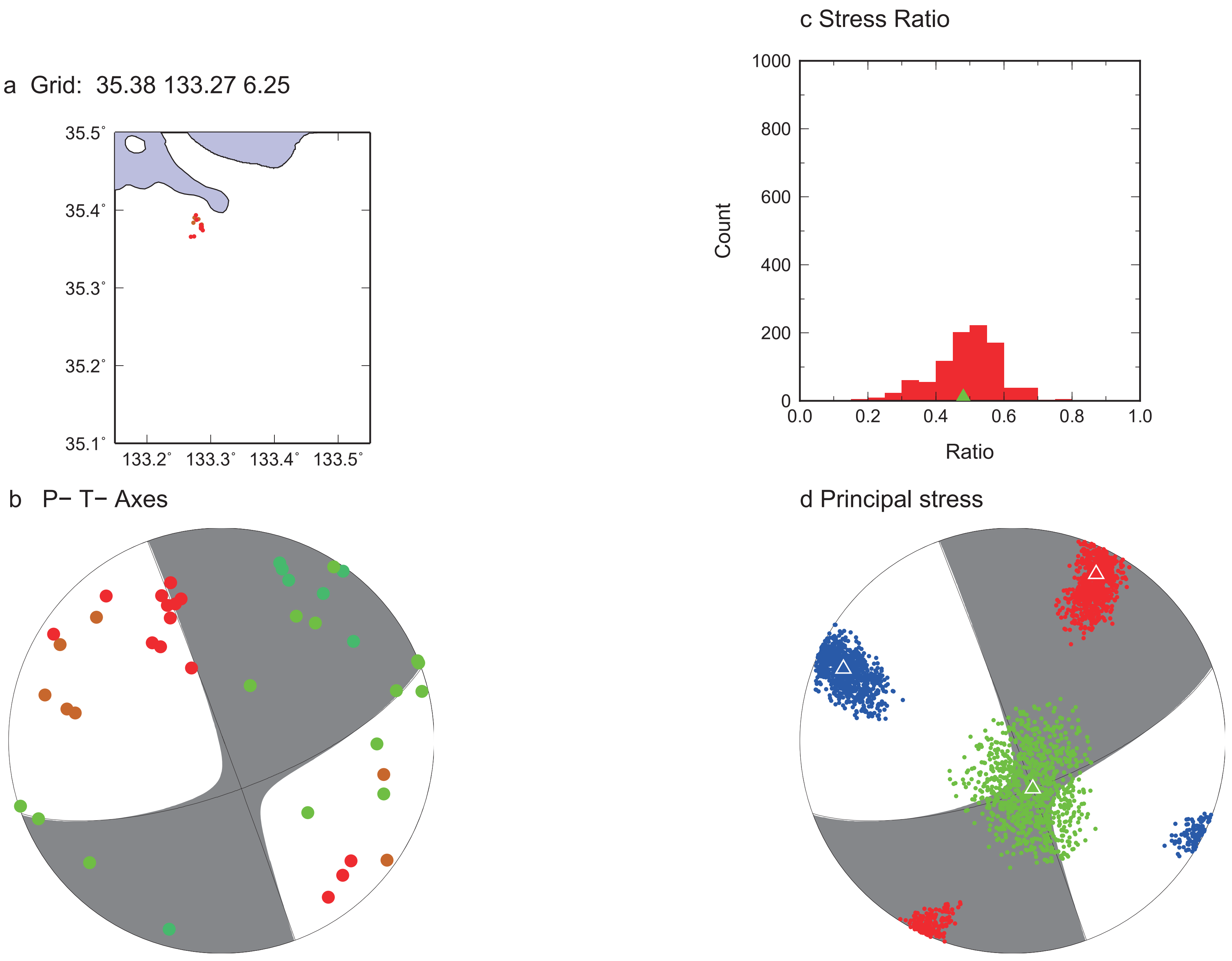
a Grid: 35.38 133.27 6.25
35.5˚
35.4˚
35.3˚
35.2˚
35.1˚
133.2˚
133.3˚
133.4˚
133.5˚
b P− T− Axes
c Stress Ratio
1000
800
600
400
200
0
Count
0.0
0.2
0.4
0.6
0.8
1.0
Ratio
d Principal stress

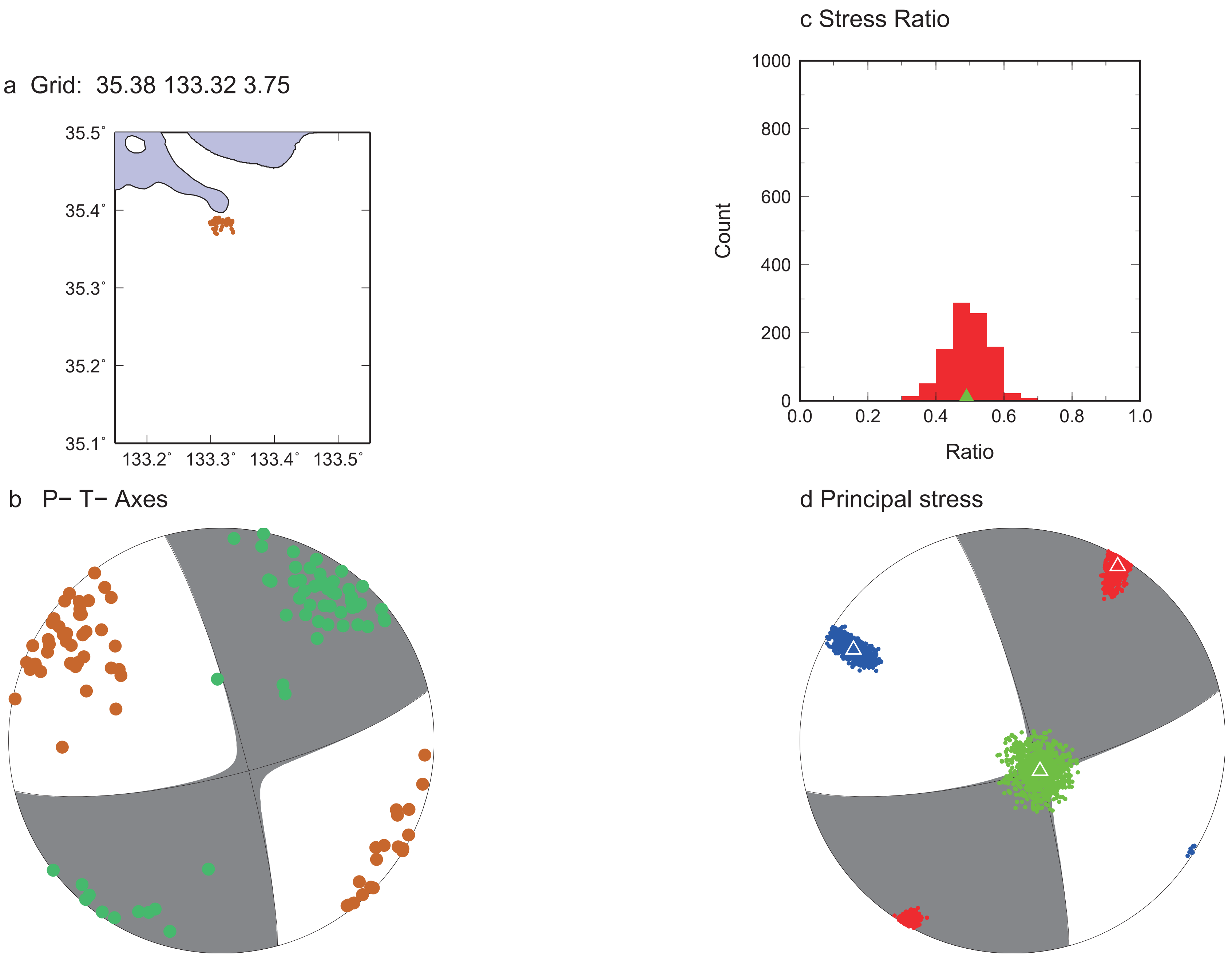

a Grid: 35.38 133.32 3.75
35.5˚
35.4˚
35.3˚
35.2˚
35.1˚
133.2˚
133.3˚
133.4˚
133.5˚
b P− T− Axes
c Stress Ratio
Count
1000
800
600
400
200
0
0.0
0.2
0.4
0.6
0.8
1.0
Ratio
d Principal stress

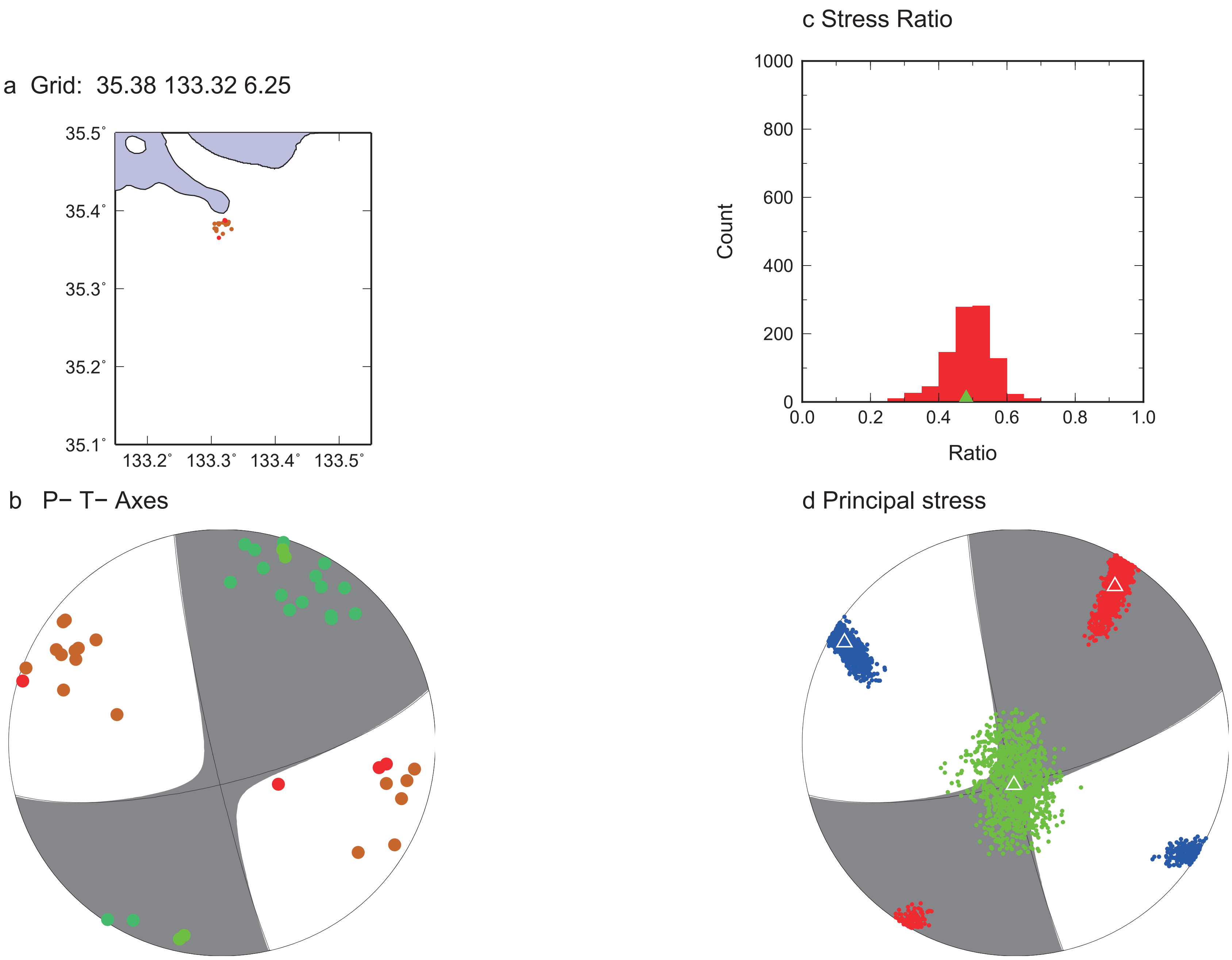
a Grid: 35.38 133.32 6.25
35.5˚
35.4˚
35.3˚
35.2˚
35.1˚
133.2˚
133.3˚
133.4˚
133.5˚
c Stress Ratio
1000
800
600
400
200
0
Count
0.0
0.2
0.4
0.6
0.8
1.0
Ratio
b P− T− Axes
d Principal stress

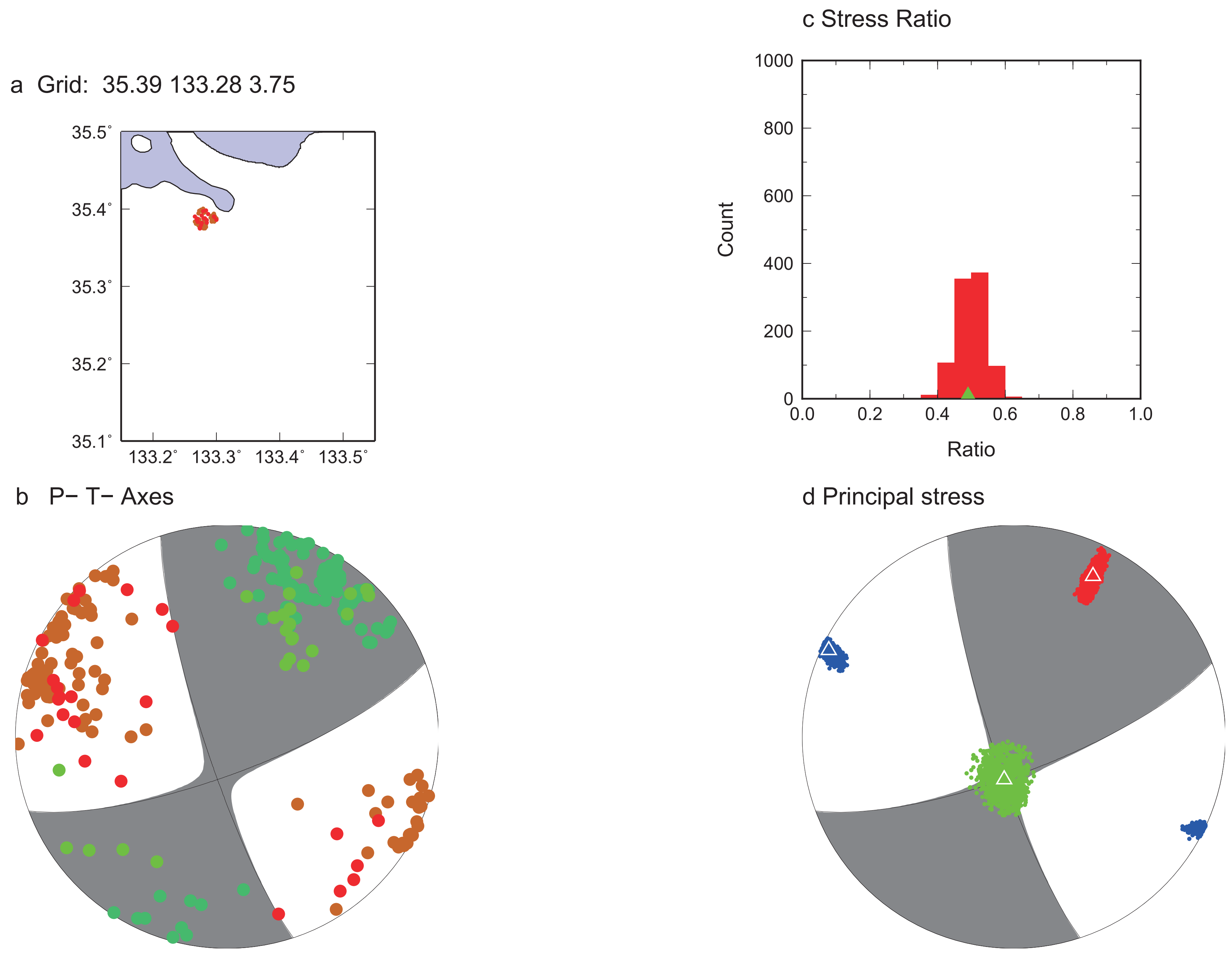

a Grid: 35.39 133.28 3.75
35.5˚
35.4˚
35.3˚
35.2˚
35.1˚
133.2˚ 133.3˚ 133.4˚ 133.5˚
b P− T− Axes
c Stress Ratio
Count
1000
800
600
400
200
0
0.0 0.2 0.4 0.6 0.8 1.0
Ratio
d Principal stress

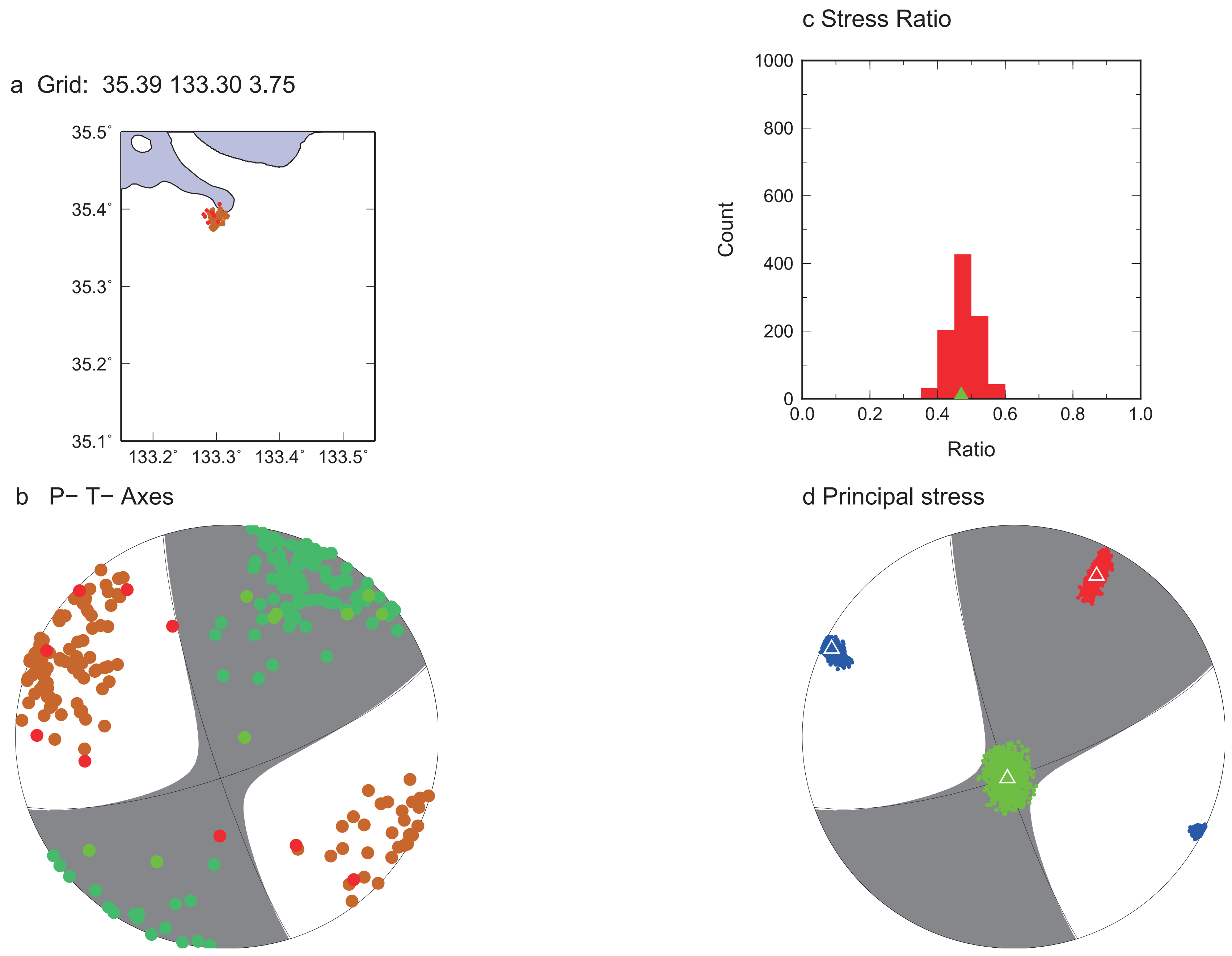
a Grid: 35.39 133.30 3.75
35.5˚
35.4˚
35.3˚
35.2˚
35.1˚
133.2˚ 133.3˚ 133.4˚ 133.5˚
b P− T− Axes
c Stress Ratio
Count
1000
800
600
400
200
0
0.0 0.2 0.4 0.6 0.8 1.0
Ratio
d Principal stress

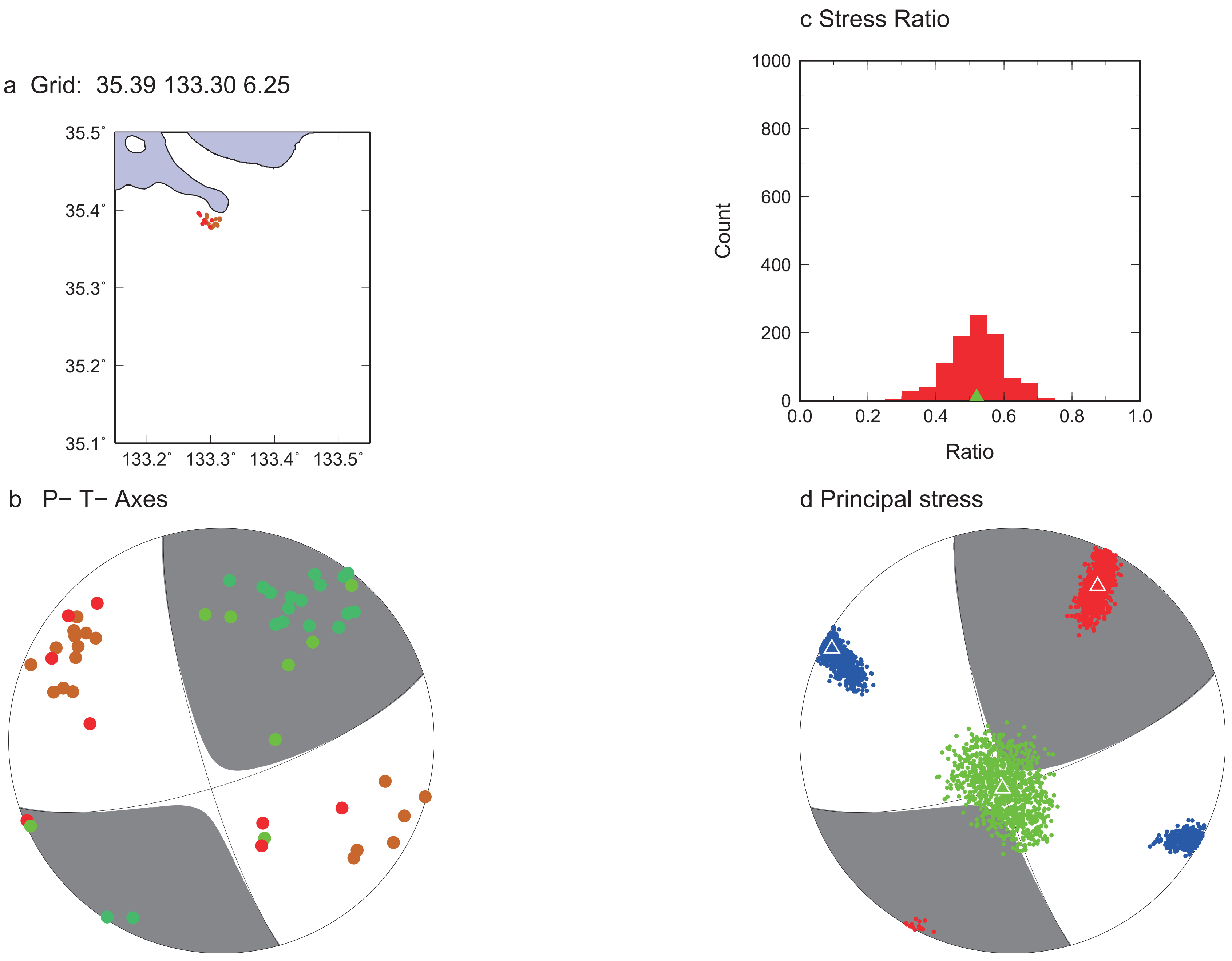
a Grid: 35.39 133.30 6.25
35.5°
35.4°
35.3°
35.2°
35.1°
133.2°
133.3°
133.4°
133.5°
c Stress Ratio
1000
800
600
400
200
0
Count
0.0
0.2
0.4
0.6
0.8
1.0
Ratio
b P– T– Axes
d Principal stress

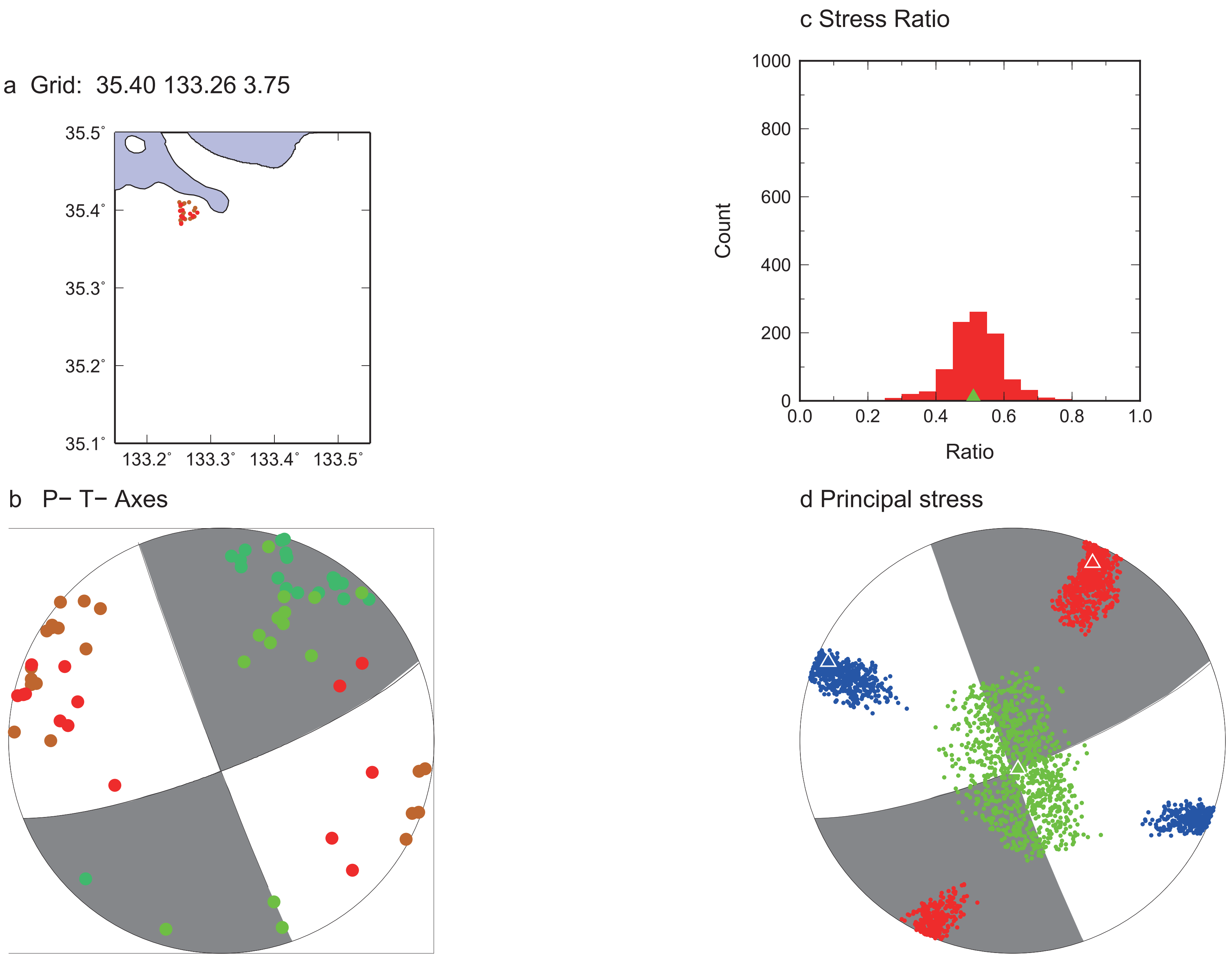
a Grid: 35.40 133.26 3.75
35.5˚
35.4˚
35.3˚
35.2˚
35.1˚
133.2˚
133.3˚
133.4˚
133.5˚
b P− T− Axes
c Stress Ratio
1000
800
600
400
200
0
Count
0.0
0.2
0.4
0.6
0.8
1.0
Ratio
d Principal stress

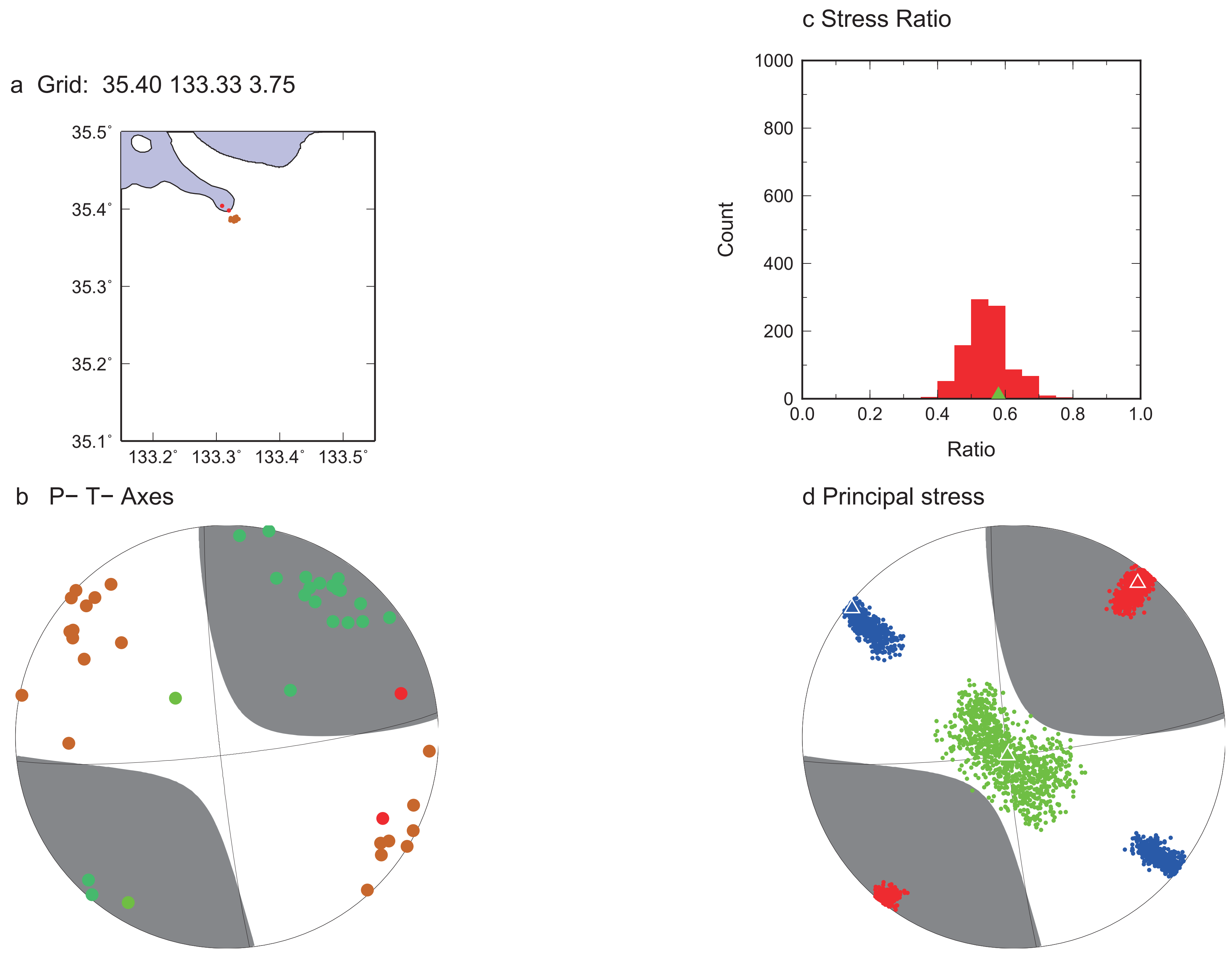

a Grid: 35.40 133.33 3.75
35.5˚
35.4˚
35.3˚
35.2˚
35.1˚
133.2˚
133.3˚
133.4˚
133.5˚
c Stress Ratio
Count
1000
800
600
400
200
0
0.0
0.2
0.4
0.6
0.8
1.0
Ratio
b P− T− Axes
d Principal stress

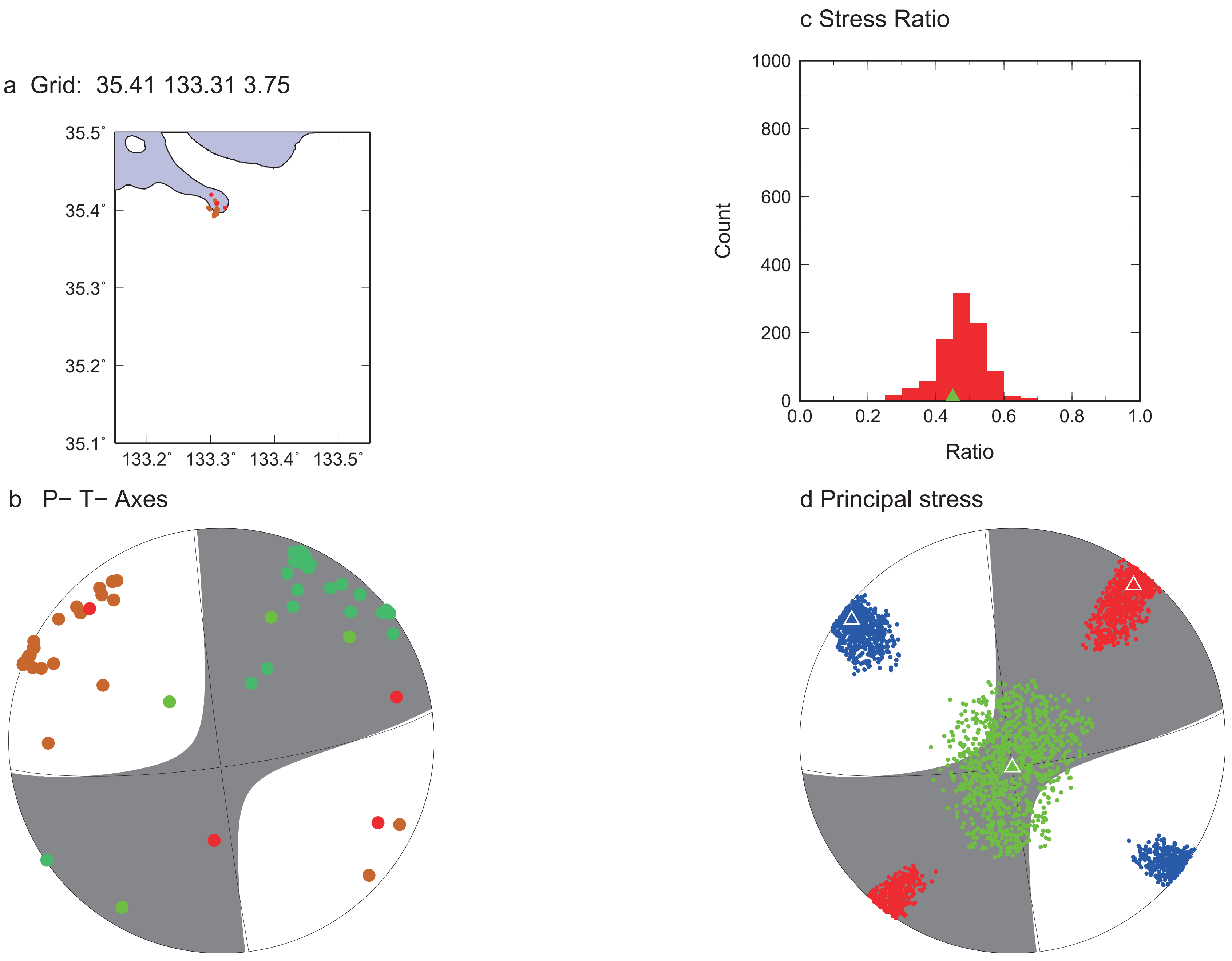
a Grid: 35.41 133.31 3.75
35.5˚
35.4˚
35.3˚
35.2˚
35.1˚
133.2˚
133.3˚
133.4˚
133.5˚
b P– T– Axes
c Stress Ratio
1000
800
600
400
200
0
Count
0.0
0.2
0.4
0.6
0.8
1.0
Ratio
d Principal stress
